# The Synthesis, Characterization, Cytotoxic Activity Assessment and Structure-Activity Relationship of 4-Aryl-6-(2,5-dichlorothiophen-3-yl)-2-methoxypyridine-3-carbonitriles

**DOI:** 10.3390/molecules24224072

**Published:** 2019-11-10

**Authors:** Mahmoud Al-Refai, Mohammad M. Ibrahim, Mohamad Nurul Azmi, Hasnah Osman, Mohamad Hafizi Abu Bakar, Armin Geyer

**Affiliations:** 1 Department of Chemistry, Faculty of Science, Al al-Bayt University, P.O. BOX 130040, Al-Mafraq 25113, Jordan; mohammadibrahim@aabu.edu.jo; 2 School of Chemical Sciences, Universiti Sains Malaysia, Minden 11800, Pulau Pinang, Malaysia; mnazmi@usm.my (M.N.A.); ohasnah@usm.my (H.O.); 3 Bioprocess Technology Division, School of Industrial Technology, Universiti Sains Malaysia, Minden 11800, Pulau Pinang, Malaysia; mhafizi88@usm.my; 4 Faculty of Chemistry, Philipps University Marburg, Hans-Meerwein-Straße 4, 35032 Marburg, Germany; geyer@staff.uni-marburg.de

**Keywords:** carbonitrile, methoxypyridine, thiophene, malononitrile, cytotoxicity, liver cancer, prostate cancer, breast cancer

## Abstract

A series of 2-methoxypyridine-3-carbonitrile (**5a**–**i**)-bearing aryl substituents were successfully synthesized in good yields by the condensation of chalcones (**4a**–**i**) with malononitrile in basic medium. The condensation process, in most cases, offers a route to a variety of methoxypyridine derivatives (**6a**–**g**) as side products in poor yields. All new compounds were fully characterized using different spectroscopic methods. Mass ESI-HMRS measurements were also performed. Furthermore, these compounds were screened for their in vitro cytotoxicity activities against three cancer cell lines; namely, those of the liver (line HepG2), prostate (line DU145) and breast (line MBA-MB-231). The cytotoxicity assessment revealed that compounds **5d**, **5g**, **5h** and **5i** exhibit promising antiproliferative effects (IC_50_ 1–5 µM) against those three cancer cell lines.

## 1. Introduction

**Figure 1:**
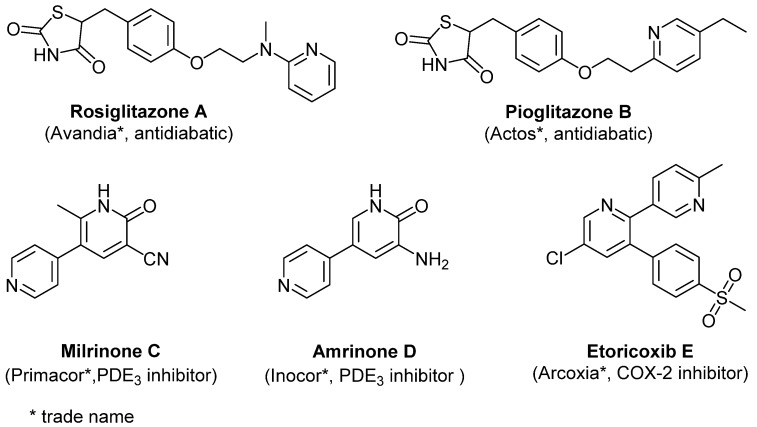
Clinically-used pyridine-containing compounds.

**Scheme 1:**
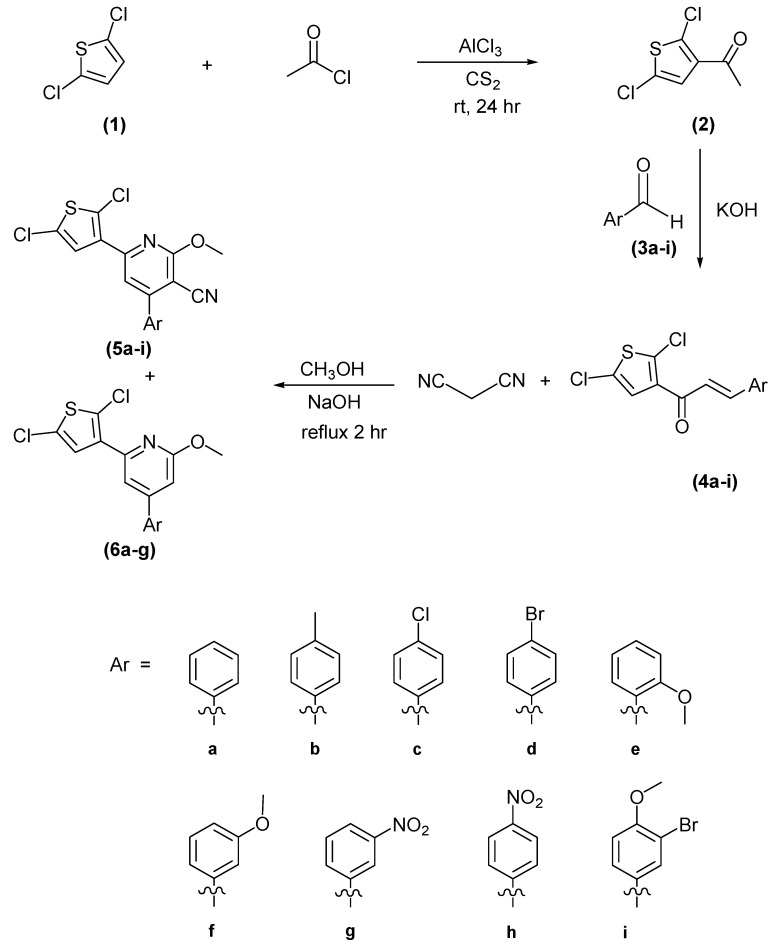
Synthesis of pyridine derivatives (**5** and **6**).

Pyridine is an important skeletal substituent and valuable organic moiety in many biologically-active and clinically-used compounds [1,2,3]. Such compounds display a wide spectrum of pharmacological activities: antimicrobial [4], antioxidant [5], HIV inhibitors [6], antimalarial [7], anticancer [8], and many others [9]. Pyridine is also used in many clinically-used agents; namely, rosiglitazone **A** [1,2,3], pioglitazone **B** [1,2,3], milrinone **C** [1,2,3], amrinone **D** [3] and etoricoxib **E** [4,10] (Figure 1). Pyridine-3-carbonitriles, in particular, have been found to possess many biological activities and many optical and electrical qualities [11]. Furthermore, they are important precursors for synthetic manipulations and have been commonly used as precursors and key intermediates in organic synthesis [12,13]. In recent years, we reported the synthesis of different pyridine derivatives bearing carbonitrile and 2,5-dichlorothiophene substituents [14,15,16,17,18,19]. In continuation of our ongoing work, herein, we present the synthesis and characterization of new pyridine-3-carbonitrile derivatives (Scheme 1). The new derivatives were screened for their cytotoxic activity against three cancer cell lines, namely, those of the liver (line HepG2), prostate (line DU145) and breast (line MBA-MB-231) are discussed.

## 2. Results and Discussion

### 2.1. Synthesis and Characterization

The derivative 3-acetyl-2,5-dichlorothiophene, **2**, was prepared following a published procedure [20] by Friedel–Crafts acylation of 2,5-dichlorthiophene, **1**, in carbon disulfide (CS_2_). The condensation of 3-acetyl-2,5-dichloro-thiophene (**2)** with different aromatic aldehydes, **3a**–**i**, in the presence of potassium hydroxide, afforded chalcones **4a**–**i** (Scheme 1). The targeted pyridine-3-carbonitrile derivatives, **5a**–**i** were obtained by the reaction of chalcones, **4a**–**i**, with malononitrile using methanolic solution of sodium hydroxide in good yields. In some cases, decyanation of pyridine-3-carbonitrile derivatives, **5a**–**i**, provided the pyridine compounds **6a**–**g** in poor yields; see Scheme 1. Spectroscopic data (^1^H-NMR, ^13^C-NMR, COSY, HSQC and HMBC) and mass ESI-HMRS measurements support the proposed structures for compounds **5a**–**i** and **6a**–**g**.

**Scheme 2:**
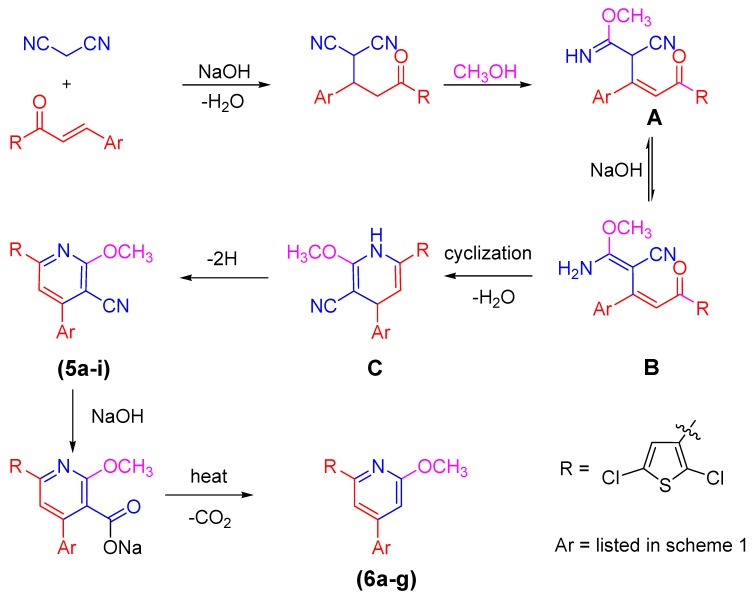
Proposed mechanism for the formation of pyridine derivatives (**5** and **6**).

The formation of 2-methoxypyridine-3-carbonitrile products **5a**–**i**, Scheme 2, was assumed to take place via conjugated addition of the malononitrile anion to the β-carbon of the corresponding chalcone, followed by methanol attack to C≡N group, leading to the imine A which tautomerizes to amine derivative B. The intermolecular dehydrative cyclization of amine B furnished the dihydropyridine C which oxidizes (aromatization) to the desired **5a**–**i** product (Scheme 2) [21,22,23,24]. The decyanation of **5a**–**i** is believed to proceed through the hydrolysis of the nitrile derivative to the corresponding carboxylic acid followed by the decarboxylation to form pyridines **6a**–**g** (Scheme 2).

All new compounds were characterized by ^1^H and ^13^C-NMR, and 2D-NMR (See Supplementary Materials). In the ^1^H-NMR spectra of compounds **5a**–**i**, the methoxy protons (OCH_3_-2) appeared at *δ* 4.16–4.20. The singlet signals which resonated at 7.58–7.67 and 7.40–7.43 were assigned to pyridine (H-5) and thiophene (H-4′) protons, respectively. This was supported by HMBC and COSY correlations. In addition, the ^1^H-NMR spectra of compounds **6a**–**g** exhibit two singlets at 7.41–7.43 and 4.04–4.08 attributed to H-4′ of thiophene and the methoxy protons (OCH_3–_2), respectively. The two mutually-coupled doublet signals at *δ* 7.66–7.72 and 6.89–6.97, respectively, could be assigned to H-5 and H-3 protons, respectively, based on their coupling constants (*J* = 1.2–1.7 Hz), and the chemical shifts of carbons to which they are attached (C-5, *δ* 113.9–117.0; C-3, *δ* 107.3–110.3).

The ^13^C-NMR spectra of compounds **5a**–**i** and **6a**–**g** are very similar, with one significant exception that the chemical shifts of C-3 in the series **5a**–**i**, appeared at 95.9 ppm instead of the 107.3 ppm for compounds **6a**–**g**. Finally, all structural assignments are well-matched with the high-resolution HSESIMS data and further confirmed by a combination of COSY, HSQC and HMBC experiments (See Supplementary Materials). A complete analysis of all NMR data is listed in the experimental section.

### 2.2. Cytotoxicity Properties

All compounds tested, **5a**–**i** and **6a**–**g**, were subjected to different concentrations for cytotoxicity screening against three human cancer cell lines, namely, HepG2 (liver), DU145 (prostate) and MBA-MB-231 (breast) along with standard 5-fluorouracil. The cytotoxicity scanning results showed variable degrees of toxicity on all the cancer cell lines (Table 1).

In general, the cytotoxicity results, based on IC_50_ values for the compounds we tested, displayed considerable effects (<100 µM) against all cancer cell lines, but **5a**, **5b** and **5c** did not (>100 µM). Among compounds tested, **5i** exhibited the most promising anticancer activity with a considerably broad spectrum of cytotoxic activity against all three human cancer cell lines. In tissue-specific toxicity, compounds **5d**, **5h** and **5i** showed very strong cytotoxic activity against HepG2 cell lines, all having IC_50_ values of 1.53 µM; comparatively, 5-fluorouracil had an IC_50_ value of 1.65 µM. Both compounds **5h** and **5i** demonstrated potent cytotoxic effects on DU145 cell lines with respect to the cytotoxicity of the 5-fluorouracil. Compound **5i** was the most potent cytotoxic agent against MBA-MB-231 cell lines, with an IC_50_ value of 1.38 µM for each.

On the other hand, compounds **6a**–**g** displayed mild to moderate cytotoxic activity with IC_50_ values in the range of 10.34–64.59 µM against all of the cancer cell lines. In addition, the selectivity indexes of compounds were determined in this study. All compounds were tested on normal human fibroblast cell lines. The IC_50_ values of compounds **5d**, **5g**, **5h** and **5i** were found to be high in normal human fibroblast cell lines (Table 1), suggesting their high selectivity for cancer cells over normal cells. These results may provide some important justification for further development as anticancer agents.

### 2.3. Structure-Activity Relationship (SAR)

The pyridine-3-carbonitrile derivatives (**5a**–**i**) showed more potent inhibition between 1 and 5 µM than the pyridine derivatives (**6a**–**g**) (Table 1). To explore the structure–activity relationship of pyridine-3-carbonitriles (**5**), the aromatic substituents in **5a** were initially replaced with 4-methylbenzene (**5b**), 4-chlorobenzene (**5c**), 4-bromobenzene (**5d**), 2-methoxybenzene (**5e**), 3-methoxybenzene (**5f**), 3-nitrobenzene (**5g**), 4-nitrobenzene (**5h**) and 3-bromo-4-methoxybenzene (**5i**) groups. 

The activity was increased in the **5d** derivative compared to the chloro-**5c** one. This leads to the conclusion that relatively larger substituents are valuable for good activity. Introducing a methoxy group at position 2 of the phenyl ring of **5f**, increases the activity. Therefore, the presence of a hydrophilic substituent on the phenyl ring will increase the activity. To explore the influence of electron withdrawing groups, a nitro group was introduced at positions 3 and 4 for **5g** and **5h**, respectively. Surprisingly, these two compounds showed very potent inhibitions against cell lines between 1 and 5 µM. Consequently, the nitro group can be considered an interesting moiety. In addition, compound **5i** with bromo and methoxy substitutions exhibited a similar activity to **5h**.

**Figure 2:**
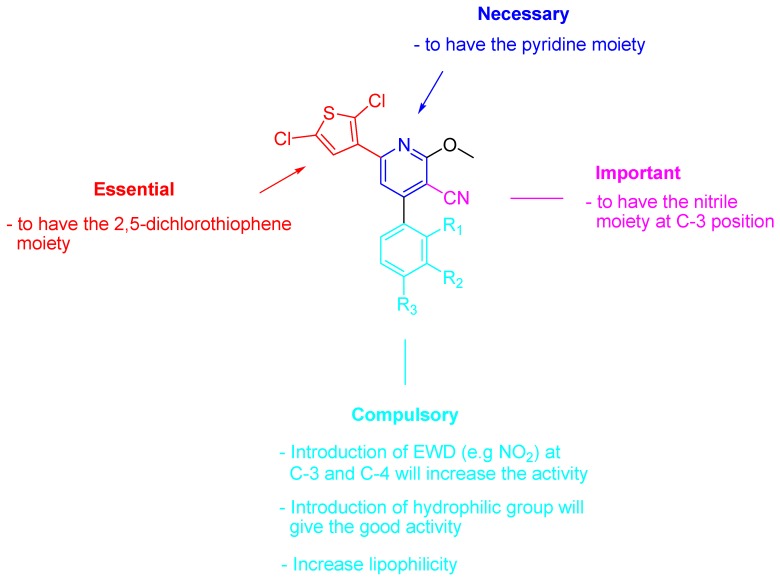
Chemical structure of the pyridine-3-carbonitriles with important sites for the structure–activity relationship (SAR).

In conclusion, the structural requirements of pyridine-3-carbonitrile derivatives to enhance their cytotoxic activity against tested cell lines are as follows: (a) the nitrile substituent at position 3 on the pyridine ring is essential for activity and as a basic pharmacophore; (b) the nitro, methoxy and chloro substituents on the benzene ring are useful for the enhancement of activity (Figure 2). Such substituents were selected to offer variable lipophilic, electronic and steric environments in order to influence the biological activity being targeted; said specificity is believed to be responsible for the biological significance of some anticancer agents [11].

## 3. Materials and Methods

### 3.1. Materials

The starting material, 3-acetyl-2,5-dichlorothiophene (**2**) was prepared as described in the previously-reported procedure [20]. The chalcone compounds, **4a**–**i**, were synthesized according to literature’s methods [25]. All reagents were purchased from Fluka and used as purchased. Solvents were dried and distilled according to standard protocols. 

### 3.2. Instrumentation

^1^H (500 MHz) and ^13^C (125 MHz) NMR spectra were recorded in CDCl_3_ used as an internal standard at 300 K on Bruker spectrometers (Bruker Biospin Mri GmbH, Ettlingen, Germany). Chemical shifts (*δ*) are given in parts per million (ppm) and were determined from the center of the respective coupling pattern (s: singlet; d: doublet; dd: doublet of d; t: triplet). ESI-HMRS measurements were performed on an LTQ-FT mass spectrometer (Thermo Fisher Scientific, Schwerte, Germany). All reactions were monitored using thin layer chromatography (TLC) coated with silica gel (60 F_254_, Merck, Darmstadt, Germany). Pure compounds were obtained by preparative TLC plates using a CHCl_3_/pentane (30:70) mixture as an eluent.

### 3.3. Synthesis

Synthesis of chalcones **4a**–**i**, went according to the reported procedure [19]. A solution of 3-acetyl-2,5-dichlorothiophene, **2**, in MeOH (10 mL) was added dropwise to an equimolar mixture of the corresponding aldehydes **3a**–**I** and KOH in MeOH (50 mL). After the addition was completed, the reaction mixture was stirred at RT for 10 h. The precipitate formed was filtered off, washed with MeOH and dried without any further purification.

#### 3.3.1. General procedure for the Synthesis 6-(2,5-dichlorothiophen-3-yl)-2-methoxy-4-(4-methoxyphenyl)pyridines (5a–i) and (6a–g)

The pyridines (**5a**–**i**) and (**6a**–**g**) were prepared according to the methods reported in [19,20,26,27]. A mixture of chalcone, **4**, (0.01 mol), malononitrile (0.66g, 0.01 mol) and KOH (0.56 g, 0.01 mol) in MeOH (50 mL) was refluxed for about 2 h. The reaction mixture was cooled, and then the solid obtained was filtered off, air-dried and purified using preparative TLC (20 × 20 cm) by CHCl_3_/pentane (70:30) as a mobile phase to give the desired product, Scheme 1.

#### 3.3.2. Characterization Data for Products

*6-(2,5-dichlorothiophen-3-yl)-2-methoxy-4-phenylpyridine-3-carbonitrile* (**5a**). White solid (1.30 g, 72% yield). M.p.: 156–157 °C. IR (cm^−1^) *ν* = 2219 (-CN, nitrile), 1689, 1535, 1451, 1440, 1355, 1224, 1112, 1014, 839, 763. ^1^H-NMR (CDCl_3_, 500 MHz): *δ* = 7.67 (m, 2H, H-2″, 6″), 7.65 (s, 1H, H-5), 7.56 (m, 3H, H-3″, 4″, 5″), 7.42 (s, 1H, H-4′), 4.17 (s, 3H, OCH_3_-2). ^13^C-NMR (CDCl_3_, 125 MHz) *δ* = 164.8 (C-2), 156.6 (C-4), 151.6 (C-6), 135.9 (C-1″), 135.6 (C-3′), 130.2 (C-4″), 129.1 (C-3″,5″), 128.4 (C-2″,6″), 127.5 (C-4′), 126.8 (C-5′), 126.4 (C-2′), 115.8 (C-5), 115.2 (CN-3), 93.7 (C-3), 54.9 (OCH_3_-2). (+)-ESIMS *m/z* 361([M + H]^+^, 74), 363 ([M + H + 2]^+^, 56), 356 ([M + H + 4]^+^, 14), 383 ([M + Na]^+^, 100), 385 ([M + Na + 2]^+^, 62), 387 ([M + Na + 4]^+^, 17). (+)-HRESIMS *m*/*z* 382.9781 [M + Na]^+^, 384.9752 [M + Na + 2]^+^ (calculated for C_17_H_10_Cl_2_N_2_OSNa, 382.9783).

*6-(2,5-dichlorothiophen-3-yl)-2-methoxy-4-p-tolylpyridine-3-carbonitrile* (**5b**). White solid (1.46 g, 78% yield). M.p.: 178–179 °C. IR (cm^−1^) *ν* = 2223 (-CN, nitrile), 1584, 1551, 1540, 1457, 1352, 1014, 807. ^1^H-NMR (CDCl_3_, 500 MHz): *δ* = 7.64 (s,1H, H-5), 7.58 (d, *J* = 8.3 Hz, 2H, H-2″,6″), 7.41 (s, 1H, H-4′), 7.36 (d, *J* = 8.3 Hz, 2H, H-3″, 5″), 4.16 (s, 3H, OCH_3_-2), 2.47 (s, 3H, CH_3_-4″). ^13^C-NMR (CDCl_3_, 125 MHz) *δ* = 164.8 (C-2), 156.6 (C-4), 151.5 (C-6), 140.6 (C-4″), 135.7 (C-3′), 133.1 (C-1″), 129.8 (C-3″,5″), 128.3 (C-2″,6″), 127.5 (C-4′), 126.8 (C-5′), 126.2 (C-2′), 115.7 (C-5), 115.4 (CN-3), 93.5 (C-3), 54.8 (OCH_3_-2), 21.4 (CH_3_-4″). (+)-ESIMS *m/z* 397 ([M + Na]^+^, 100), 399 ([M + Na + 2]^+^, 58), 401 ([M + Na + 4]^+^, 14). (+)-HRESIMS *m/z* 396.9936 [M + Na]^+^, 398.9907 [M + Na + 2]^+^, 400.9879 [M + Na + 4]^+^ (calculated for C_18_H_12_Cl_2_N_2_OSNa, 396.9940).

*4-(4-chlorophenyl)-6-(2,5-dichlorothiophen-3-yl)-2-methoxypyridine-3-carbonitrile* (**5c**). White solid (1.28 g, 65% yield). M.p.: 195–196 °C. IR (cm^−1^) *ν* = 2228 (-CN, nitrile), 1582, 1547, 1454, 1357, 1299, 1091, 1025, 1011, 826. ^1^H-NMR (CDCl_3_, 500 MHz): *δ* = 7.61 (d, *J* = 8.9 Hz, 2H, H-2″,6″), 7.62 (s,1H, H-5), 7.54 (d, *J* = 8.9 Hz, 2H, H-3″, 5″), 7.42 (s, 1H, H-4′), 4.18 (s, 3H, OCH_3_-2). ^13^C-NMR (CDCl_3_, 125 MHz) *δ* = 164.8 (C-2), 155.3 (C-4), 151.9 (C-6), 136.6 (C-4″), 135.5 (C-3′), 134.3 (C-1″), 129.8 (C-2″,6″), 129.4 (C-3″,5″), 127.4 (C-4′), 127.0 (C-5′), 126.5 (C-2′), 115.5 (C-5), 115.0 (CN-3), 93.5 (C-3), 55.0 (OCH_3_-2). (+)-ESIMS *m/z* 395([M + H]^+^, 16), 397 ([M + H+2]^+^, 15), 417 ([M + Na]^+^, 20), 419 ([M + Na + 2]^+^, 20). (+)-HRESIMS *m/z* 394.9578 [M + H]^+^, 396.9548 [M + H+2]^+^, 398.9519 [M + H + 4]^+^, 400.9487 [M + H + 6]^+^ (calculated for C_17_H_10_Cl_3_N_2_OS, 394.9574).

*4-(4-bromophenyl)-6-(2,5-dichlorothiophen-3-yl)-2-methoxypyridine-3-carbonitrile* (**5d**). White solid (1.47 g, 67% yield). M.p.: 214–215 °C. ^1^H-NMR (CDCl_3_, 500 MHz): *δ* = 7.71 (d, *J* = 8.6 Hz, 2H, H-3″, 5″), 7.62 (s,1H, H-5), 7.54 (d, *J* = 8.6 Hz, 2H, H-2″,6″), 7.41 (s, 1H, H-4′), 4.18 (s, 3H, OCH_3_-2). ^13^C-NMR (CDCl_3_, 125 MHz) *δ* =164.8 (C-2), 155.4 (C-4), 151.9 (C-6), 135.5 (C-3′), 134.8 (C-1″), 132.4 (C-3″,5″), 130.0 (C-2″,6″), 127.4 (C-4′), 127.0 (C-5′), 126.5 (C-2′), 125.1 (C-4″), 115.5 (C-5), 115.0 (CN-3), 93.5 (C-3), 55.0 (OCH_3_-2). (+)-ESIMS *m/z* 439 ([M + H]^+^, 63), 441 ([M + H+2]^+^, 100), 443 ([M + H + 4]^+^, 48). (+)-HRESIMS *m/z* 460.8890 [M + Na]^+^, 462.8864 [M + Na + 2]^+^, 464.8839 [M + Na + 4]^+^ (calculated for C_17_H_9_BrCl_2_N_2_OSNa, 460.8888).

*6-(2,5-dichlorothiophen-3-yl)-2-methoxy-4-(2-methoxyphenyl)pyridine-3-carbonitrile* (**5e**). White solid (1.56 g, 80% yield). M.p.: 149–150 °C. ^1^H-NMR (CDCl_3_, 500 MHz): *δ* = 7.61 (s,1H, H-5), 7.50 (m, 1H, H-4″), 7.41 (s, 1H, H-4′), 7.35 (dd, *J* = 8.3, 1.7 Hz, 1H, H-6″), 7.11 (m, 1H, H-5″), 7.09 (m, 1H, H-3″), 4.16 (s, 3H, OCH_3_-2), 3.91 (s, 3H, OCH_3_-2″). ^13^C-NMR (CDCl_3_, 125 MHz) *δ* = 164.1 (C-2), 156.3 (C-2″), 154.4 (C-4), 151.3 (C-6), 135.8 (C-3′), 131.5 (C-4″), 130.3 (C-6″), 127.5 (C-4′), 126.7 (C-5′), 126.1 (C-2′), 125.1 (C-1″), 121.0 (C-5″), 117.1 (C-5), 115.1 (CN-3), 111.6 (C-3″), 95.9 (C-3), 55.6 (OCH_3_-2″), 54.7 (OCH_3_-2). (+)-ESIMS *m/z* 391([M + H]^+^, 48), 393 ([M + H + 2]^+^, 17), 413 ([M + Na]^+^, 100), 415 ([M + Na + 2]^+^, 58), 417 ([M + Na + 4]^+^, 13). (+)-HRESIMS *m/z* 412.9896 [M + Na]^+^, 414.9867 [M + Na + 2]^+^, 416.9837 [M + Na + 4]^+^ (calculated for C_18_H_12_Cl_2_N_2_O_2_SNa, 412.9889).

*6-(2,5-dichlorothiophen-3-yl)-2-methoxy-4-(3-methoxyphenyl)pyridine-3-carbonitrile* (**5f**). White solid (1.54 g, 79% yield). M.p.: 179–180 °C. IR (cm^−1^) *ν* = 2223 (-CN, nitrile), 1601, 1540, 1511, 1376, 1350, 1257, 1169, 1022, 820. ^1^H-NMR (CDCl_3_, 500 MHz): *δ* = 7.64 (s,1H, H-5), 7.45 (t, *J* = 7.8 Hz, 1H, H-5″), 7.41 (s, 1H, H-4′), 7.22 (ddd, *J* = 7.6, 1.7, 0.9 Hz, 1H, H-6″), 7.18 (t, *J* = 2.4 Hz, 1H, H-2″), 7.07 (ddd, *J* = 8.3, 2.6, 0.9 Hz, 1H, H-4″), 4.16 (s, 3H, OCH_3_-2), 3.90 (s, 3H, OCH_3_-3″). ^13^C-NMR (CDCl_3_, 125 MHz) *δ* = 164.7 (C-2), 159.9 (C-3″), 156.5 (C-4), 151.6 (C-6), 137.2 (C-1″), 135.5 (C-3′), 130.2 (C-5″), 127.4 (C-4′), 126.8 (C-5′), 126.4 (C-2′), 120.7 (C-6″), 115.9 (C-4″),115.7 (C-5), 115.2 (CN-3), 113.9 (C-2″), 93.7 (C-3), 54.9 (OCH_3_-3″) 55.5 (OCH_3_-2). (+)-ESIMS *m/z* 391([M + H]^+^, 48), 393 ([M + H+2]^+^, 17), 413 ([M + Na]^+^, 100), 415 ([M + Na + 2]^+^, 58), 417 ([M + Na + 4]^+^, 13). (+)-HRESIMS *m/z* 412.9896 [M + Na]^+^, 414.9867 [M + Na + 2]^+^, 416.9837 [M + Na + 4]^+^ (calculated for C_18_H_12_Cl_2_N_2_O_2_SNa, 412.9889).

*6-(2,5-dichlorothiophen-3-yl)-2-methoxy-4-(3-nitrophenyl)pyridine-3-carbonitrile* (**5g**). Yellow solid (1.26 g, 62% yield). M.p.: 213–214 °C. IR (cm^−1^) *ν* = 2225 (-CN, nitrile), 1582, 1553, 1537, 1453, 1356, 1282, 1018, 837, 770. ^1^H-NMR (CDCl_3_, 500 MHz): *δ* = 8.48 (t, *J* = 2.02Hz, 1H, H-2″), 8.42 (ddd, *J* = 8.2, 2.3, 1.1Hz, 1H, H-4″), 8.03 (ddd, *J* = 7.7, 1.8, 1.0 Hz, 1H, H-6″), 7.78 (t, *J* = 8.1Hz, 1H, H-5″), 7.67 (s,1H, H-5), 7.43 (s, 1H, H-4′), 4.20 (s, 3H, OCH_3_-2). ^13^C-NMR (CDCl_3_, 125 MHz) *δ* =164.8 (C-2), 154.0 (C-4), 152.4 (C-6), 148.6 (C-3″), 137.5 (C-1″), 135.1 (C-3′), 134.3 (C-6″), 130.3 (C-5″), 127.3 (C-4′), 127.2 (C-5′), 127.0 (C-2′), 124.8 (C-4″),123.5 (C-2″), 115.4 (C-5), 114.5 (CN-3), 93.7 (C-3), 55.1 (OCH_3_-2). (+)-ESIMS *m/z* 428 ([M + Na]^+^, 100), 430 ([M + Na + 2]^+^, 66), 432 ([M + Na + 4]^+^, 14). (+)-HRESIMS *m/z* 427.9629 [M + Na]^+^, 429.9601 [M + Na + 2]^+^, 431.9574 [M + Na + 4]^+^ (calculated for C_17_H_9_Cl_2_N_3_O_3_SNa, 427.9634).

*6-(2,5-dichlorothiophen-3-yl)-2-methoxy-4-(4-nitrophenyl)pyridine-3-carbonitrile* (**5h**). Brown solid (1.15 g, 57% yield). M.p.: 204–205 °C. ^1^H-NMR (CDCl_3_, 500 MHz): *δ* = 8.43 (d, *J* = 8.3 Hz, 2H, H-3″, 5″), 7.84 (d, *J* = 8.3 Hz, 2H, H-2″,6″), 7.66 (s,1H, H-5), 7.44 (s, 1H, H-4′), 4.20 (s, 3H, OCH_3_-2). ^13^C-NMR (CDCl_3_, 125 MHz) *δ* = 164.8 (C-2), 154.1 (C-4), 152.4 (C-6), 148.8 (C-4″), 142.0 (C-1″), 135.2 (C-3′), 129.6 (C-2″,6″), 127.3 (C-4′), 127.2 (C-5′), 126.9 (C-2′), 124.3 (C-3″,5″), 115.3 (C-5), 114.5 (CN-3), 93.7 (C-3), 55.1 (OCH_3_-2). (+)-ESIMS *m/z* 428 ([M + Na]^+^, 100), 430 ([M + Na + 2]^+^, 66), 432 ([M + Na + 4]^+^, 14). (+)-HRESIMS *m/z* 427.9629 [M + Na]^+^, 429.9601 [M + Na + 2]^+^, 431.9574 [M + Na + 4]^+^ (calculated for C_17_H_9_Cl_2_N_3_O_3_SNa, 427.9634).

*4-(3-bromo-4-methoxyphenyl)-6-(2,5-dichlorothiophen-3-yl)-2-methoxypyridine-3-carbonitrile* (**5i**). Yellow solid (1.76 g, 75% yield). M.p.: 191–192 °C. ^1^H-NMR (CDCl_3_, 500 MHz): *δ* = 7.82 (d, *J* = 2.5 Hz, 1H, H-2″), 7.67 (dd, *J* = 8.6, 2.4 Hz, 1H, H-6″), 7.58 (s,1H, H-5), 7.40 (s, 1H, H-4′), 7.06 (d, *J* = 8.6 Hz, 1H, H-5″), 4.16 (s, 3H, OCH_3_-2), 3.99 (s, 3H, OCH_3_-4″). ^13^C-NMR (CDCl_3_, 125 MHz) *δ* =164.9 (C-2), 157.5 (C-4″), 154.7 (C-4), 151.8 (C-6), 135.5 (C-3′), 133.2 (C-2″), 129.4 (C-1″),129.0 (C-6″), 127.4 (C-4′), 126.9 (C-5′), 126.5 (C-2′), 115.4 (C-5), 115.2 (CN-3), 112.4 (C-3″),112.0 (C-5″), 93.3 (C-3), 56.5 (OCH_3_-4″), 54.9 (OCH_3_-2). (+)-ESIMS *m/z* 491 ([M + Na]^+^, 66), 493 ([M + Na + 2]^+^, 100), 495 ([M + Na + 4]^+^, 46). (+)-HRESIMS *m/z* 490.8995 [M + Na]^+^, 492.8971 [M + Na + 2]^+^, 494.8945 [M + Na + 4]^+^ (calculated for C_18_H_11_BrCl_2_N_2_O_2_SNa, 490.8994).

*2-(2,5-dichlorothiophen-3-yl)-6-methoxy-4-phenylpyridine* (**6a**). Pale yellow solid (0.25 g, 15% yield). M.p.: 127–128 °C. ^1^H-NMR (CDCl_3_, 500 MHz): *δ* = 7.72 (d, *J* = 1.5 Hz, 1H, H-5), 7.68 (m, 2H, H-2″, 6″), 7.50 (m, 3H, H-3″,4″, 5″), 7.43 (s, 1H, H-4′), 6.95 (d, *J* = 1.5 Hz, 1H, H-3), 4.06 (s, 3H, OCH_3_-2). ^13^C-NMR (CDCl_3_, 125 MHz) *δ* =164.3 (C-2), 151.9 (C-4), 149.0 (C-6), 138.4 (C-1″), 137.2 (C-3′), 129.1 (C-4″), 129.1 (C-3″, 5″), 127.9 (C-4′),127.1 (C-2″,6″), 126.1 (C-5′), 123.7 (C-2′), 114.4 (C-5), 107.7 (C-3), 53.7 (OCH_3_-2). (+)-ESIMS *m/z* 336 ([M + H]^+^, 100), 338 ([M + H + 2]^+^, 64), 340 ([M + H + 4]^+^, 13). (+)-HRESIMS *m/z* 336.0009 [M + H]^+^, 337.9980 [M + Na + 2]^+^ (calculated for C_16_H_12_Cl_2_NOS, 336.0011).

*2-(2,5-dichlorothiophen-3-yl)-6-methoxy-4-p-tolylpyridine* (**6b**). Pale yellow solid (0.32 g, 18% yield). M.p.: 150–151 °C. ^1^H-NMR (CDCl_3_, 500 MHz): *δ* = 7.69 (d, *J* = 1.2 Hz,1H, H-5), 7.57 (d, *J* = 7.9 Hz, 2H, H-2″,6″), 7.42 (s, 1H, H-4′), 7.31 (d, *J* = 7.9 Hz, 2H, H-3″,5″), 6.92 (d, *J* = 1.2 Hz, 1H, H-3), 4.04 (s, 3H, OCH_3_-2), 2.44 (s, 3H, CH_3_-4″). ^13^C-NMR (CDCl_3_, 125 MHz) *δ* =164.3 (C-2), 151.8 (C-4), 148.8 (C-6), 139.3 (C-4″), 137.2 (C-3′), 135.4 (C-1″), 129.8 (C-3″,5″), 127.9 (C-4′), 126.9 (C-2″,6″), 126.0 (C-5′), 123.6 (C-2′), 114.3 (C-5), 107.3 (C-3), 53.7 (OCH_3_-2), 21.3 (CH_3_-4″). (+)-ESIMS *m/z* 350 ([M + H]^+^, 100), 352 ([M + H+2]^+^, 70), 354 ([M + H+4]^+^, 15). (+)-HRESIMS *m/z* 350.0166 [M + H]^+^, 352.0137 [M + H+2]^+^ (calculated for C_17_H_14_Cl_2_NOS, 350.0168).

*4-(4-chlorophenyl)-2-(2,5-dichlorothiophen-3-yl)-6-methoxypyridine* (**6c**). Pale yellow solid (0.24 g, 13% yield). M.p.: 142–143 °C. IR (cm^−1^) *ν* = 1608, 1550, 1452, 1361, 1205, 1092, 1024, 1012, 814. ^1^H-NMR (CDCl_3_, 500 MHz): *δ* = 7.65 (d, *J* = 1.3 Hz, 1H, H-5), 7.59 (d, *J* = 8.6 Hz, 2H, H-2″,6″), 7.48 (d, *J* = 8.6 Hz, 2H, H-3″, 5″), 7.41 (s, 1H, H-4′), 6.89 (d, *J* = 1.3 Hz,1H, H-3), 4.04 (s, 3H, OCH_3_-2). ^13^C-NMR (CDCl_3_, 125 MHz) *δ* =164.3 (C-2), 150.6 (C-4), 149.1 (C-6), 137.0 (C-3′), 136.7 (C-1″), 135.3 (C-4″), 129.3 (C-3″,5″), 128.4 (C-2″,6″), 127.8 (C-4′), 126.1 (C-5′), 123.8 (C-2′), 114.0 (C-5), 107.5 (C-3), 53.7 (OCH_3_-2). (+)-ESIMS *m/z* 370 ([M + H]^+^, 100), 372 ([M + H + 2]^+^, 98), 374 ([M + H + 4]^+^, 55). (+)-HRESIMS *m/z* 369.9620 [M + H]^+^, 371.9591 [M + H + 2]^+^, 373.9562 [M + H + 4]^+^ (calculated for C_16_H_11_Cl_3_NOS, 369.9621).

*4-(4-bromophenyl)-2-(2,5-dichlorothiophen-3-yl)-6-methoxypyridine* (**6d**). Pale yellow solid (0.31 g, 15% yield). M.p.: 148–149 °C. IR (cm^−1^) *ν* = 1606, 1550, 1448, 1357, 1207, 1019, 1007, 809. ^1^H-NMR (CDCl_3_, 500 MHz): *δ* = 7.66 (d, *J* = 1.4 Hz, 1H, H-5), 7.64 (d, *J* = 8.5 Hz, 2H, H-3″, 5″), 7.53 (d, *J* = 8.5 Hz, 2H, H-2″,6″), 7.42 (s, 1H, H-4′), 6.89 (d, *J* = 1.4 Hz, 1H, H-3), 4.05 (s, 3H, OCH_3_-2). ^13^C-NMR (CDCl_3_, 125 MHz) *δ* = 164.3 (C-2), 150.6 (C-4), 149.2 (C-6), 137.3 (C-1″), 137.0 (C-3′), 132.3 (C-3″,5″), 128.6 (C-2″,6″), 127.8 (C-4′), 126.2 (C-5′), 123.8 (C-2′), 123.5 (C-4″), 114.0 (C-5), 107.5 (C-3), 53.7 (OCH_3_-2). (+)-ESIMS *m/z* 414 ([M + H]^+^, 60), 416 ([M + H+2]^+^, 100), 418 ([M + H+4]^+^, 45). (+)-HRESIMS *m/z* 413.9117 [M + H]^+^, 415.9094 [M + H+2]^+^, 417.9067 [M + H+4]^+^, 419.9042 [M + H+6]^+^ (calculated for C_16_H_11_BrCl_2_NOS, 413.9116).

*2-(2,5-dichlorothiophen-3-yl)-6-methoxy-4-(2-methoxyphenyl)pyridine* (**6e**). Pale yellow solid (0.39 g, 21% yield). M.p.: 135–136 °C. ^1^H-NMR (CDCl_3_, 500 MHz): *δ* = 7.40 (m, *J* = 8.3, 1.7 Hz, 1H, H-6″), 7.68 (d, *J* = 1.3 Hz, 1H, H-5), 7.44 (m, 1H, H-4″), 7.41 (s, 1H, H-4′), 7.09 (m, 1H, H-5″), 7.05 (dd, *J* = 8.3, 1.1 Hz, 1H, H-3″), 6.93 (d, *J* = 1.3 Hz,1H, H-3), 4.05 (s, 3H, OCH_3_-2), 3.88 (s, 3H, OCH_3_-2″). ^13^C-NMR (CDCl_3_, 125 MHz) *δ* = 163.7 (C-2), 156.6 (C-2″), 149.5 (C-4), 148.0 (C-6), 137.5 (C-3′), 130.4 (C-4″),130.1 (C-6″), 128.0 (C-4′), 127.8 (C-1″), 125.9 (C-5′), 123.3 (C-2′), 121.0 (C-5″), 117.0 (C-5), 111.5 (C-3″), 110.3 (C-3), 55.6 (OCH_3_-2″), 53.5 (OCH_3_-2). (+)-ESIMS *m/z* 366 ([M + H]^+^, 100), 368 ([M + H+2]^+^, 65), 370 ([M + H+4]^+^, 15). (+)-HRESIMS *m/z* 366.0117 [M + H]^+^, 368.0085 [M + H+2]^+^, 370.0057 [M + H+4]^+^ (calculated for C_17_H_14_Cl_2_NO_2_S, 366.0117).

*2-(2,5-dichlorothiophen-3-yl)-6-methoxy-4-(3-methoxyphenyl)pyridine* (**6f**). Pale yellow solid (0.33 g, 18% yield). M.p.: 156–157 °C. ^1^H-NMR (CDCl_3_, 500 MHz): *δ* = 7.70 (d, *J* = 1.7 Hz, 1H, H-5), 7.43 (t, *J* = 8.3 Hz, 1H, H-5″), 7.42 (s, 1H, H-4′), 7.26 (ddd, *J* = 7.7, 1.4, 0.9 Hz, 1H, H-6″), 7.19 (t, *J* = 1.9 Hz, 1H, H-2″), 7.02 (ddd, *J* = 8.3, 2.4, 0.8 Hz, 1H, H-4″), 6.94 (d, *J* = 1.7 Hz, 1H, H-3), 4.06 (s, 3H, OCH_3_-2), 3.91 (s, 3H, OCH_3_-3″). ^13^C-NMR (CDCl_3_, 125 MHz) *δ* = 164.2 (C-2), 160.1 (C-3″), 151.8 (C-4), 149.0 (C-6), 139.8 (C-1″), 137.2 (C-3′), 130.1 (C-5″), 127.9 (C-4′), 126.1 (C-5′), 123.7 (C-2′), 119.5 (C-6″), 114.4 (C-4″),114.4 (C-5),112.9 (C-2″), 107.7 (C-3), 55.4 (OCH_3_-3″) 53.7 (OCH_3_-2). (+)-ESIMS *m/z* 366 ([M + H]^+^, 100), 368 ([M + H+2]^+^, 65), 370 ([M + H+4]^+^, 15). (+)-RESIMS *m/z* 366.0117 [M + H]^+^, 368.0085 [M + H+2]^+^, 370.0057 [M + H+4]^+^ (calculated for C_17_H_14_Cl_2_NO_2_S, 366.0117).

*2-(2,5-dichlorothiophen-3-yl)-6-methoxy-4-(3-nitrophenyl)pyridine* (**6g**). Pale yellow solid (0.21 g, 11% yield). M.p.: 182–183 °C. IR (cm^−1^) *ν* = 1604, 1556, 1521, 1454, 1349, 1211, 1025, 834, 737. ^1^H-NMR (CDCl_3_, 500 MHz): *δ* = 8.45 (t, *J* = 1.8 Hz, 1H, H-2″), 8.34 (ddd, *J* = 8.1, 2.0, 1.3 Hz, 1H, H-4″), 7.99 (ddd, *J* = 7.3,1.8, 1.8 Hz, 1H, H-6″), 7.73 (t, *J* = 8.3 Hz, 1H, H-5″), 7.72 (d, *J* = 1.3 Hz, 1H, H-5), 7.44 (s, 1H, H-4′), 6.97 (d, *J* = 1.3 Hz, 1H, H-3), 4.08 (s, 3H, OCH_3_-2). ^13^C-NMR (CDCl_3_, 125 MHz) *δ* =164.5 (C-2), 149.7 (C-6), 149.3 (C-4), 148.9 (C-3″), 140.2 (C-1″), 136.8 (C-3′), 133.0 (C-6″), 130.2 (C-5″), 127.7 (C-4′), 126.4 (C-5′), 124.1 (C-2′), 123.8 (C-4″),122.1 (C-2″), 113.9 (C-5), 107.9 (C-3), 53.8 (OCH_3_-2). (+)-ESIMS *m/z* 381 ([M + H]^+^, 16), 383 ([M + H + 2]^+^, 10). (+)-HRESIMS *m/z* 380.9860 [M + H]^+^, 382.9831 [M + H+2]^+^ (calculated for C_16_H_11_Cl_2_N_2_O_3_S, 380.9862).

### 3.4. Cell Culture and Maintenance

Three different cancer cell lines; namely, DU145 derived from prostate cancer cells (American Type Culture Collection No. HTB-81), HepG2 derived from human liver cancer cells (American Type Culture Collection No. HB-8065), MBA-MB-231 derived from breast cancer cells (American Type Culture Collection No. HTB-26) and normal human fibroblasts (HSF1184) were grown and cultivated according to our previously described method [18]. Briefly, cells were seeded in Dulbecco′s modified Eagle’s media (DMEM) supplemented with 10% heat-inactivated fetal bovine serum (FBS), 100 IU/mL penicillin, 100 mg/mL streptomycin and 2 mM glutamine. The cells were maintained in a humidified atmosphere with 5% CO_2_ at 37 °C for 8 days, with subsequent media renewal. Then, cells were plated in a 96-well flat bottom plate at a concentration of 104 cells/well in fresh complete growth medium for 24 h and maintained in 37 °C in a 5% CO_2_-humidified incubator.

### 3.5. Cytotoxicity Assay

Cells in 96-well flat bottom plates were first serum-starved for 4 h by replacing cultured media with fresh medium (without serum). All synthesized compounds were properly dissolved in dimethyl sulfoxide (DMSO) to produce a stock concentration of 0.1 M. Cells were incubated with a range of different concentrations of the compounds we were testing (0–1000 μM) to give a final volume of 300 µL per well. In comparison, cells were incubated either alone with DMSO (negative control) or with 5-fluorouracil as a positive control. DMSO was used as a vehicle for the dissolution of the tested compounds and its final concentration in cultured media was less than 0.1%. Triplicate wells were designed for each individual dose. The compounds we synthesized were evaluated for in-vitro cytotoxicity properties via the standard 3-(4,5-dimethythiazol-2-yl)-2,5-diphenyl tetrazolium bromide (MTT) assay accordingly to our previously-reported work [2]. The normal human fibroblast line (HSF1184) was used as a standard reference for the measurement of selectivity indexes (SIs) relative to cancer cell lines. The IC_50_ values and SIs were calculated accordingly.

## 4. Conclusions

In conclusion, a series of 2-methoxypyridine-3-carbonitrile bearing aryl substituents were synthesized, in most cases in good yields, by the condensation of chalcones with malononitrile in basic medium. Although decarbonylation byproducts were observed in poor yields, they offer a route to a variety of methoxypyridine derivatives. All new compounds were fully characterized using different spectroscopic methods. Furthermore, these compounds were screened for their in-vitro cytotoxicity activities against three cancer cell lines; namely, those of the liver (line HepG2), prostate (line DU145) and breast (line MBA-MB-231). Although the IC_50_ values are not very impressive, their selectivity with respect to normal cells is, in some cases, very high. Remarkably, the decarbonylated substrates show less cytotoxicity on normal cells than in cancer cells. The cytotoxicity assessments revealed that compounds **5d**, **5g**, **5h** and **5i** exhibit promising antiproliferative effects (IC_50_ 1–5 µM) against tested cancer cell lines.

## Figures and Tables

**Table 1:** Cytotoxic activities and selectivity indexes (SIs) of 2-methoxypyridine derivatives.

Tested cpd.	IC_50_ Values (µM)						
	DU145	SI	HepG2	SI	MDA-MB-231	SI	HSF1184
**5a**	>100	ND	49.55 ± 0.51	1.66	21.15 ± 0.37	3.90	82.39 ± 0.04
**5b**	>100	ND	9.48 ± 0.63	12.08	>100	ND	114.54 ± 0.38
**5c**	42.36 ± 0.21	2.18	>100	ND	28.34 ± 0.42	3.26	92.38 ± 0.38
**5d**	3.63 ± 0.17	116.65	1.62 ± 0.15	261.39	2.59 ± 0.04	163.49	423.45 ± 0.49
**5f**	27.45 ± 0.63	3.48	18.34 ± 0.31	5.21	43.49 ± 0.06	2.20	95.49 ± 0.29
**5g**	4.97 ± 0.35	27.28	2.11 ± 0.26	64.26	1.93 ± 0.03	70.25	135.59 ± 0.55
**5h**	1.77 ± 0.38	105.93	1.53 ± 0.38	122.54	2.14 ± 0.06	87.61	187.49 ± 0.14
**5i**	1.82 ± 0.21	128.82	1.72 ± 0.42	136.31	1.38 ± 0.03	169.89	234.45 ± 0.27
**6a**	53.49 ± 0.02	1.67	32.31 ± 0.23	2.77	15.34 ± 0.41	5.82	89.34 ± 0.55
**6b**	64.59 ± 0.08	1.59	39.49 ± 0.58	2.59	52.39 ± 0.31	1.95	102.39 ± 0.30
**6c**	21.65 ± 0.12	5.19	11.28 ± 0.22	9.96	10.34 ± 0. 37	10.87	112.38 ± 0.47
**6d**	29.45 ± 0.88	3.14	64.59 ± 0.33	1.43	15.43 ± 0.48	6.00	92.55 ± 0.42
**6f**	18.45 ± 0.02	5.68	38.45 ± 0.62	2.73	64.39 ± 0.28	1.63	104.83 ± 0.71
**6g**	25.42 ± 0.38	3.33	39.43 ± 0.09	2.14	18.34 ± 0.34	4.61	84.56 ± 0.40
5-fluorouracil	1.92 ± 0.24	96.60	1.65 ± 0.09	112.41	2.21 ± 0.28	83.93	185.48 ± 0.48

## Supplementary Materials

NMR and Mass spectra for the compounds we prepared are available online.
