# Supplementary material for: The Synthesis, Characterization, Cytotoxic Activity Assessment and Structure-Activity Relationship of 4-Aryl-6-(2,5-dichlorothiophen-3-yl)-2-methoxypyridine-3-carbonitriles
Source: Molecules. 2019 Nov 10;24(22):4072. doi: 10.3390/molecules24224072 (PMC6891325; doi:10.3390/molecules24224072)
Supplement: Supplementary file 1 [file molecules-24-04072-s001.pdf]

Synthesis, Characterization, Cytotoxic Activity Assessment and Structure Activity Relationship of 4-Aryl-6-(2,5-dichlorothiophen-3-yl)-2-methoxypyridine-3-carbonitriles

Mahmoud Al-Refai[1*], Mohammad M. Ibrahim[1], Mohamad Nurul Azmi[2], Hasnah Osman[2], Mohamad Hafizi Abu Bakar[3], Armin Geyer[4].

1 *Department of Chemistry, Faculty of Science, Al al-Bayt University, P.O.BOX 130040, Al-Mafraq 25113, Jordan.*

2 *School of Chemical Sciences, Universiti Sains Malaysia, 11800 Minden, Pulau Pinang, Malaysia.*

3 *Bioprocess Technology Division, School of Industrial Technology, Universiti Sains Malaysia, 11800 Minden, Pulau Pinang, Malaysia.*

4 *Faculty of Chemistry, Philipps University Marburg, Hans-Meerwein-Straße 4, 35032 Marburg, Germany.*

*Corresponding author. Mahmoud Al-Refai, Department of Chemistry, Faculty of Science, University of Al al-Bayt, P.O.BOX 130040, Al-Mafraq 25113, Jordan. E-mail: mahmoud_alrefai@aabu.edu.jo

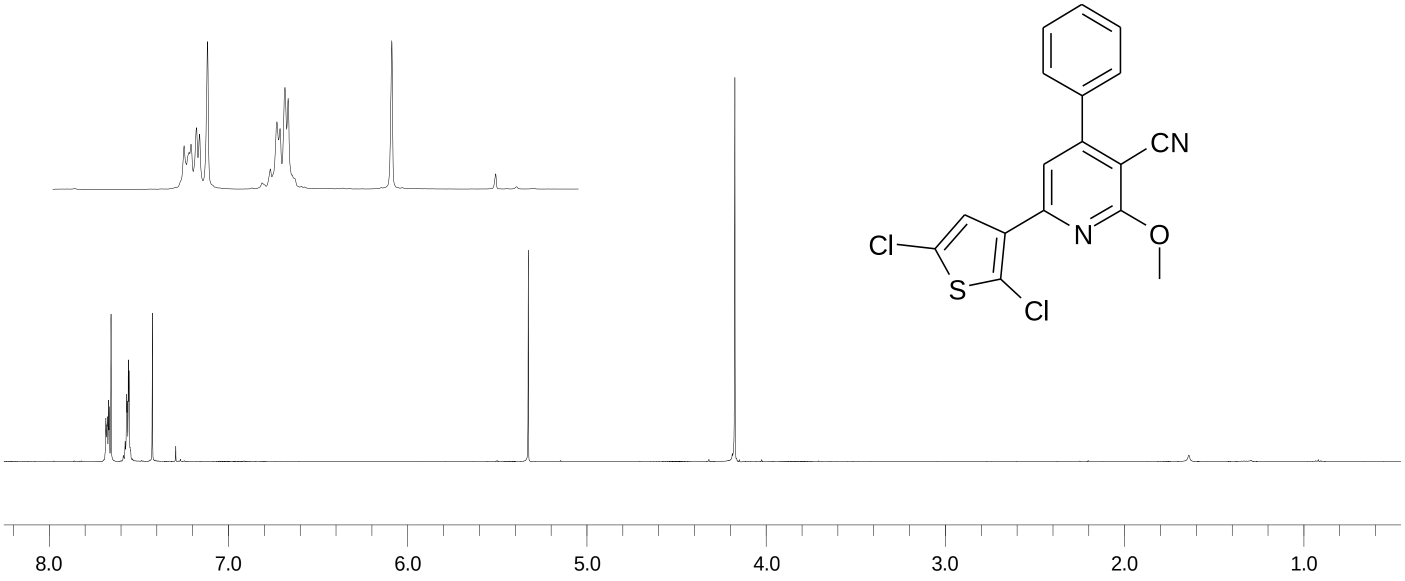

S 1: ^{1}H NMR spectrum ($CDCl_3$, 500 MHz) of 6-(2,5-dichlorothiophen-3-yl)-2-methoxy-4-phenylpyridine-3-carbonitrile (**5a**).

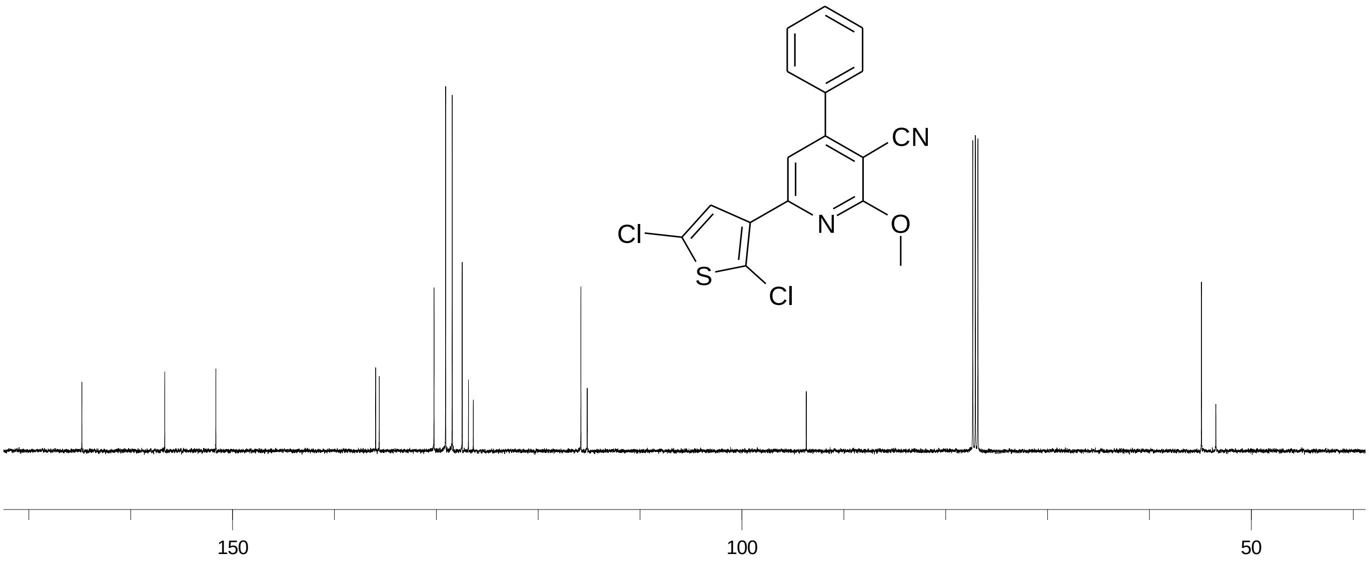

S 2: ^{13}C NMR spectrum ($CDCl_3$, 125MHz) of 6-(2,5-dichlorothiophen-3-yl)-2-methoxy-4-phenylpyridine-3-carbonitrile (**5a**).

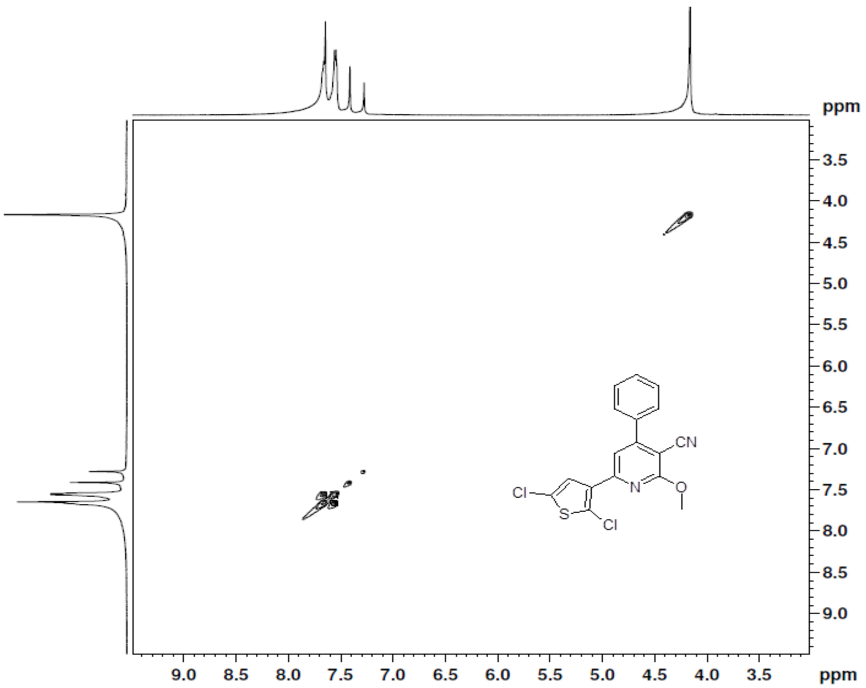

S 3 : H,H COSY spectrum ($CDCl_3$, 300 MHz) of 6-(2,5-dichlorothiophen-3-yl)-2-methoxy-4-phenylpyridine-3-carbonitrile (**5a**).

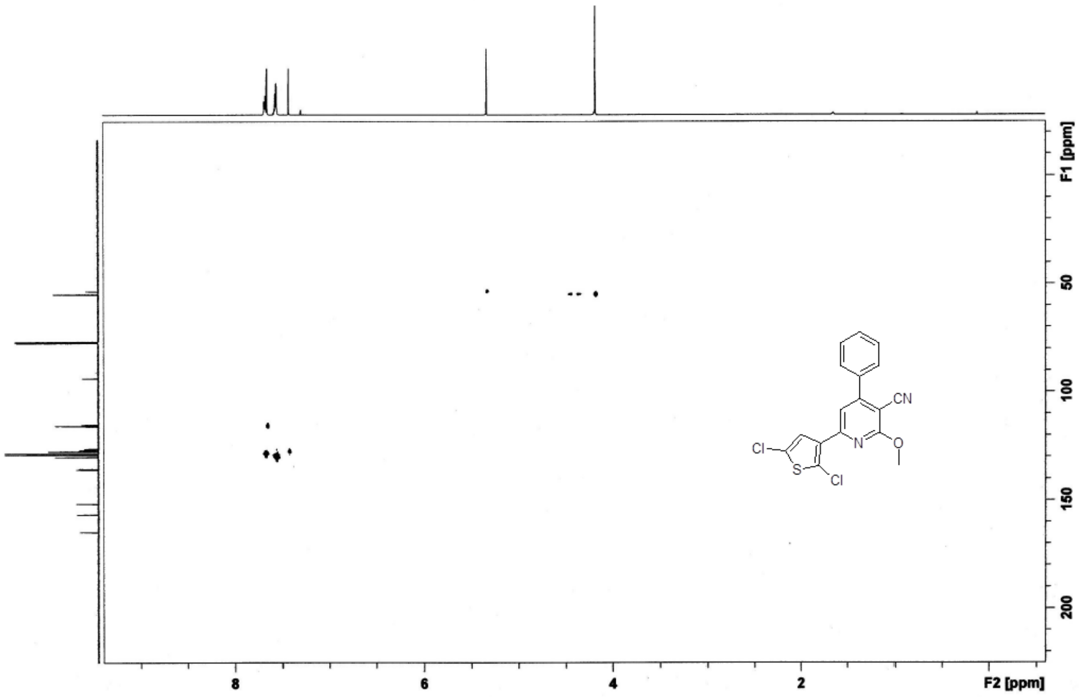

S 4: HSQC spectrum ($CDCl_3$, 500 MHz) of 6-(2,5-dichlorothiophen-3-yl)-2-methoxy-4-phenylpyridine-3-carbonitrile (**5a**).

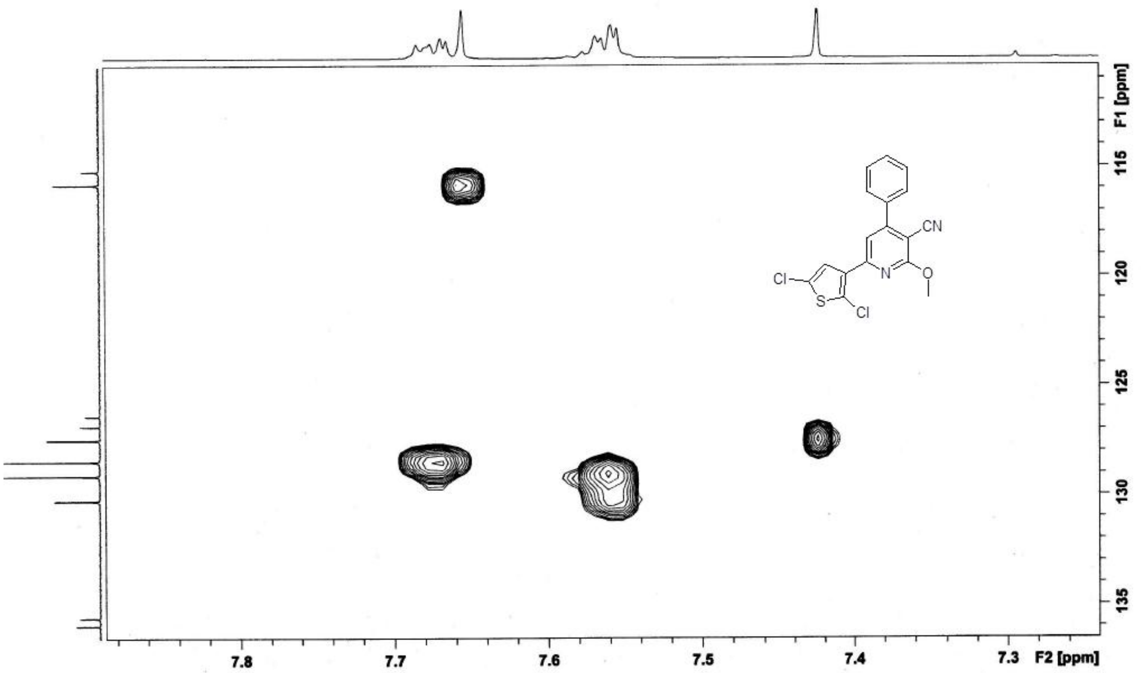

S 5: HSQC spectrum ($CDCl_3$, 500 MHz) of 6-(2,5-dichlorothiophen-3-yl)-2-methoxy-4-phenylpyridine-3-carbonitrile (**5a**).

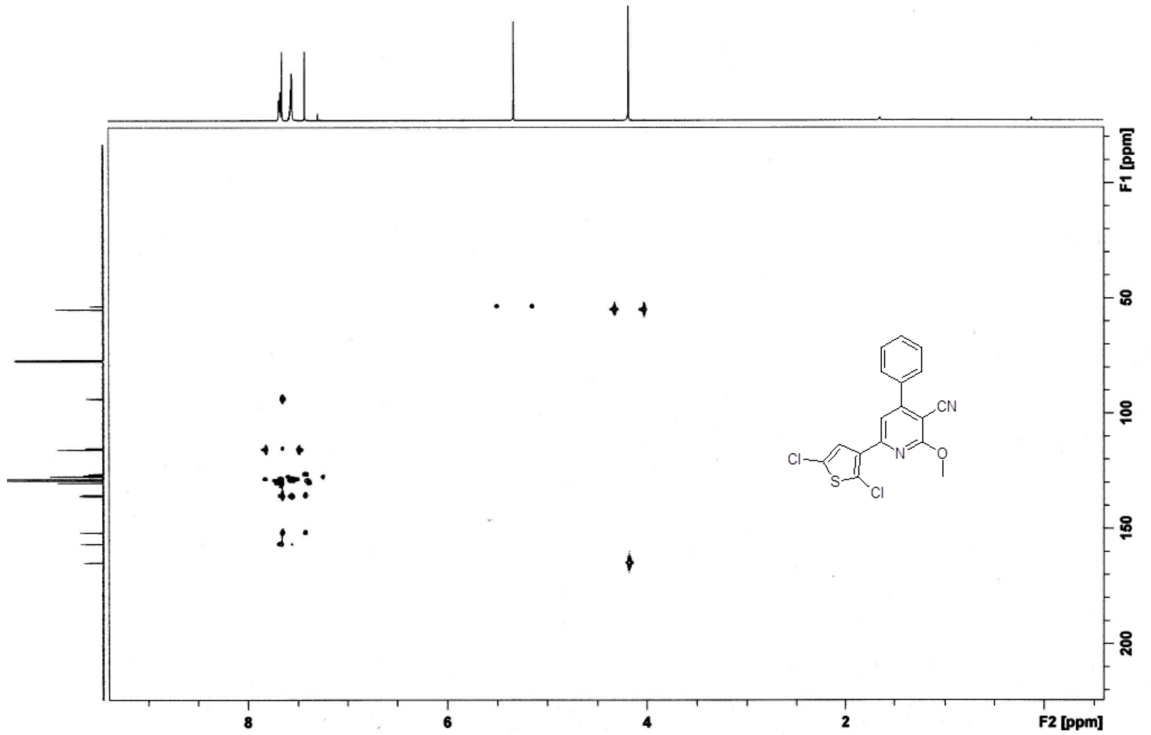

S 6: HMBC spectrum ($CDCl_3$, 500 MHz) of 6-(2,5-dichlorothiophen-3-yl)-2-methoxy-4-phenylpyridine-3-carbonitrile (**5a**).

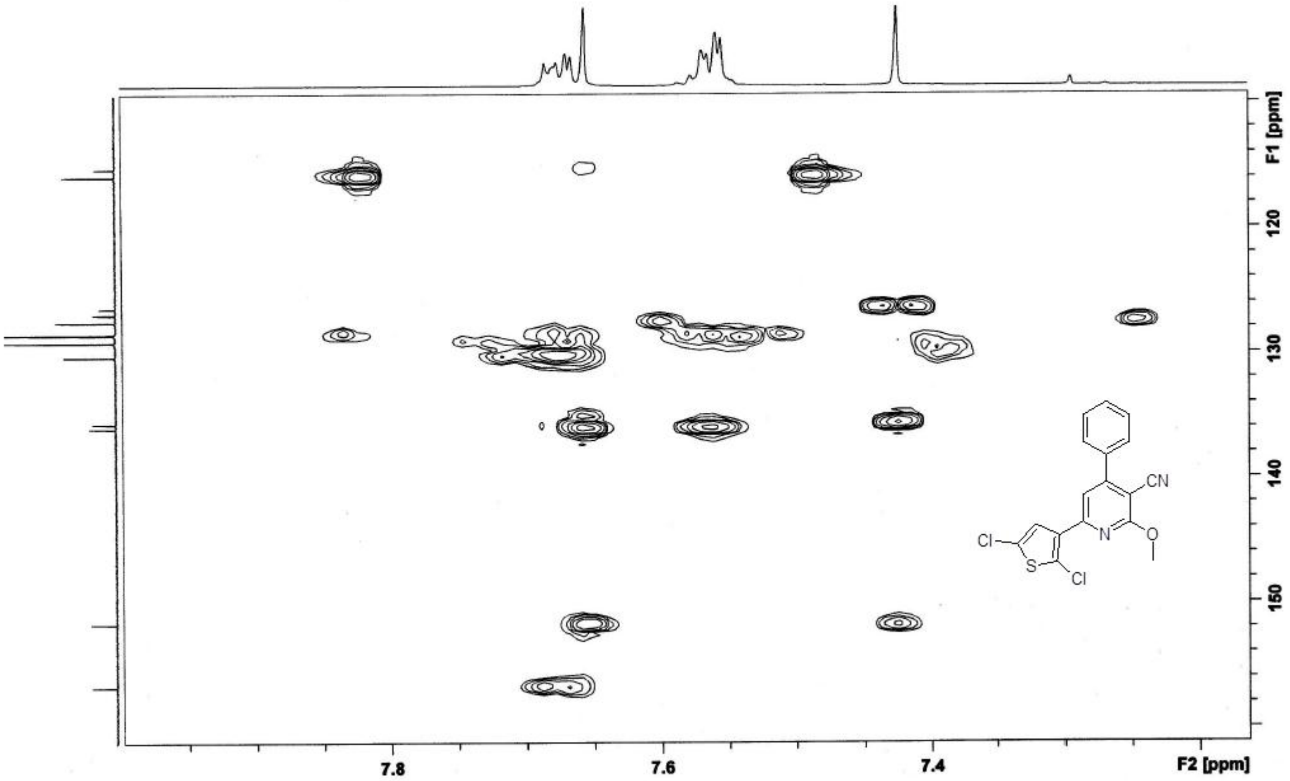

S 7: HMBC spectrum ($CDCl_3$, 500 MHz) of 6-(2,5-dichlorothiophen-3-yl)-2-methoxy-4-phenylpyridine-3-carbonitrile (**5a**).

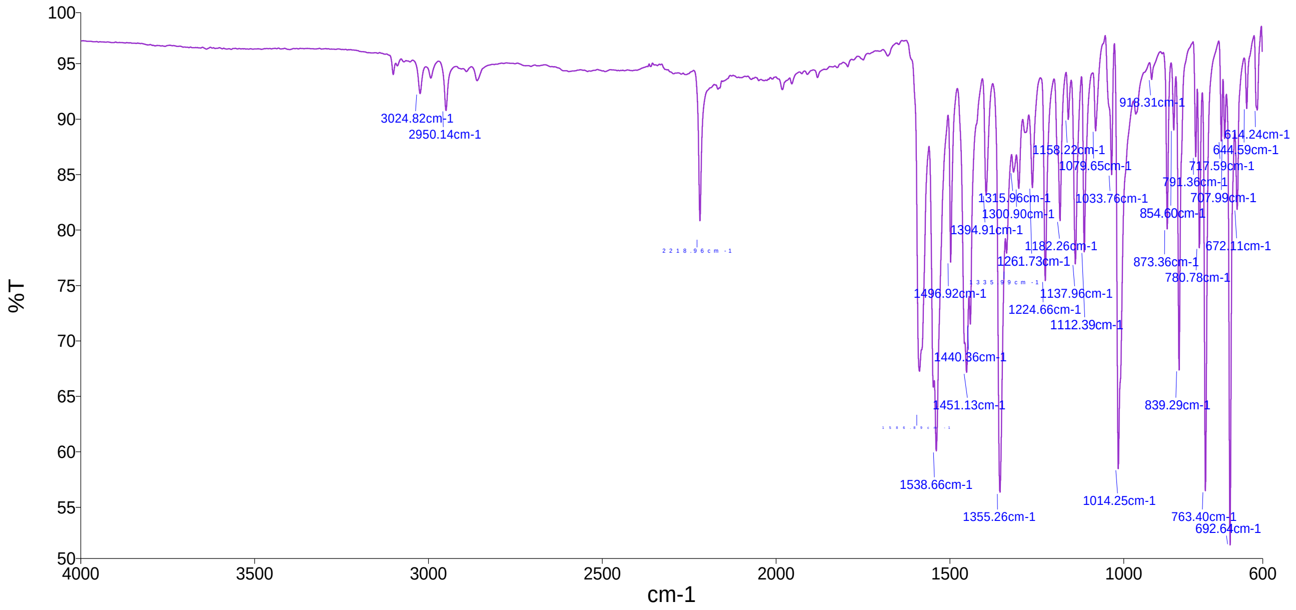

S 8: IR spectrum of 6-(2,5-dichlorothiophen-3-yl)-2-methoxy-4-phenylpyridine-3-carbonitrile (**5a**).

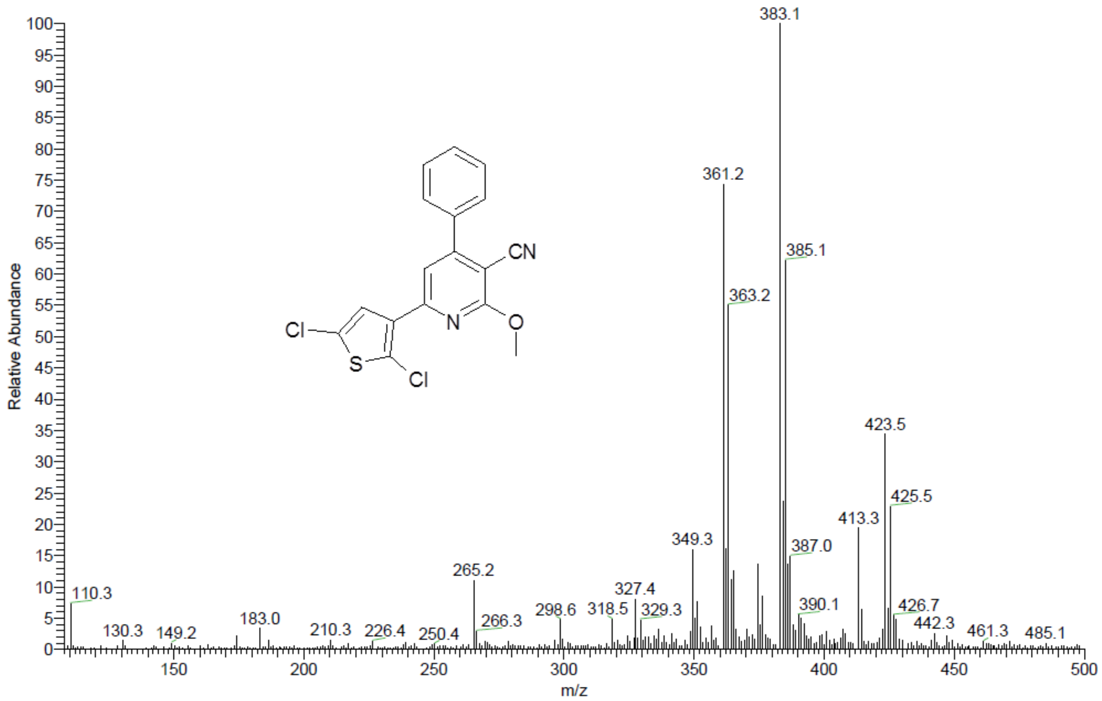

S 9: ESI mass spectrum of 6-(2,5-dichlorothiophen-3-yl)-2-methoxy-4-phenylpyridine-3-carbonitrile (**5a**).

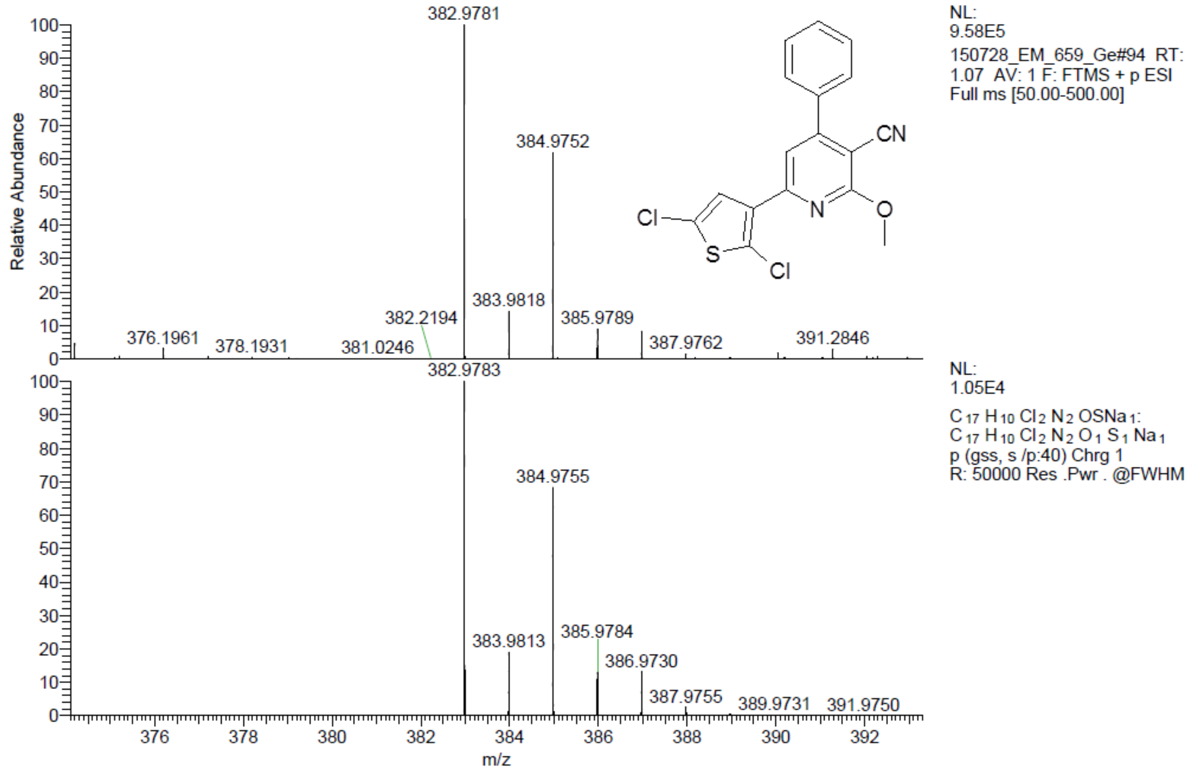

S 10: HRESI mass spectrum of 6-(2,5-dichlorothiophen-3-yl)-2-methoxy-4-phenylpyridine-3-carbonitrile (**5a**).

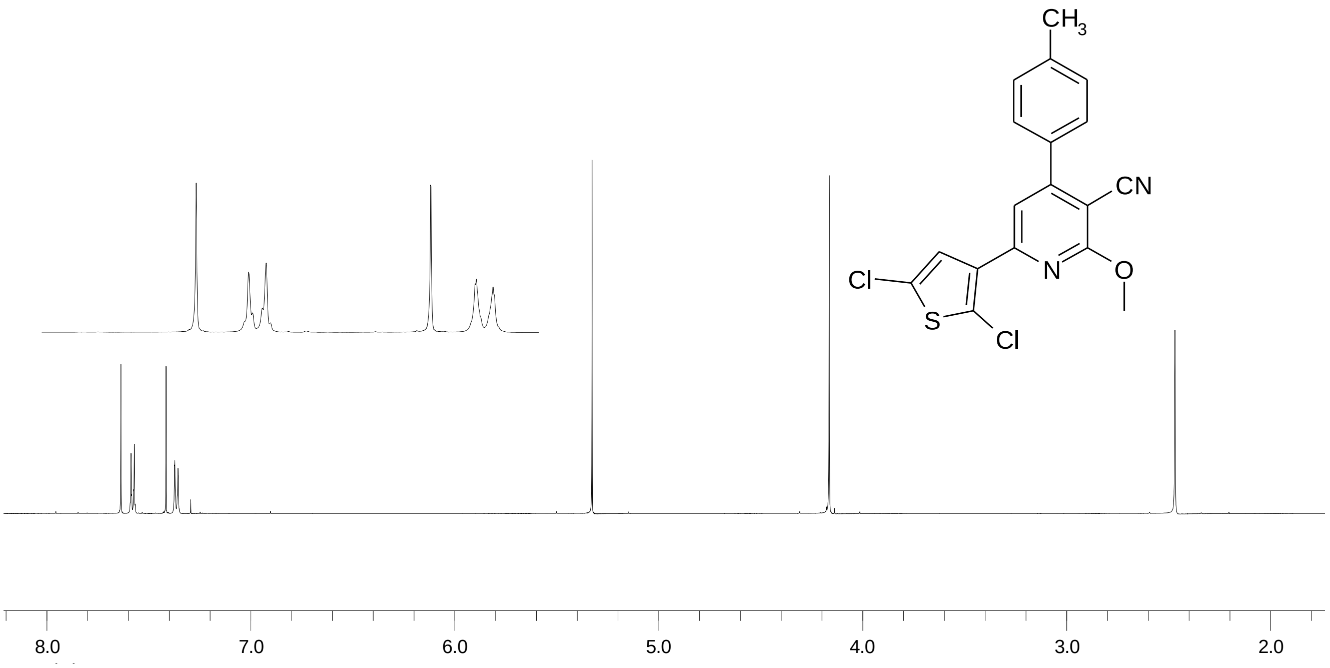

S 11: ^{1}H NMR spectrum ($CDCl_3$, 500 MHz) of 6-(2,5-dichlorothiophen-3-yl)-2-methoxy-4-*p*-tolylpyridine-3-carbonitrile (**5b**).

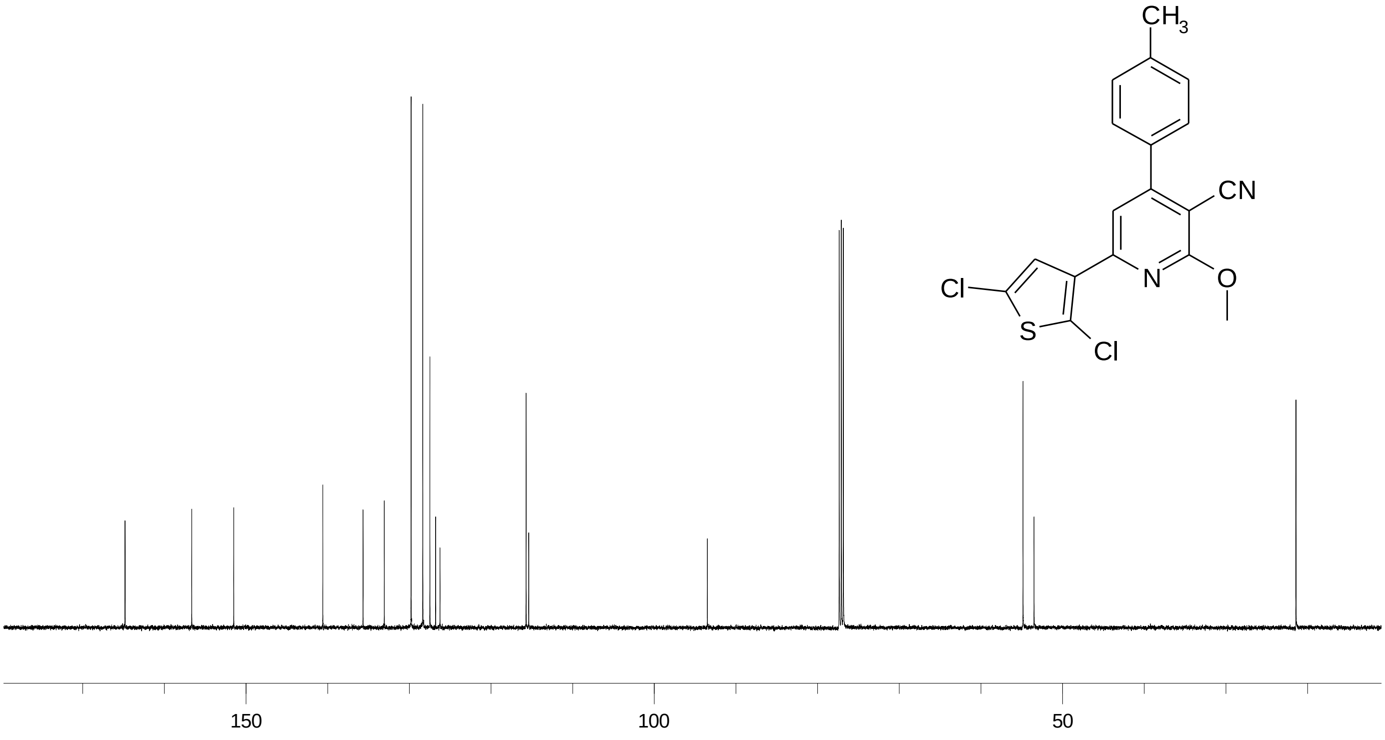

S 12: ^{13}C NMR spectrum ($CDCl_3$, 125MHz) of 6-(2,5-dichlorothiophen-3-yl)-2-methoxy-4-*p*-tolylpyridine-3-carbonitrile (**5b**).

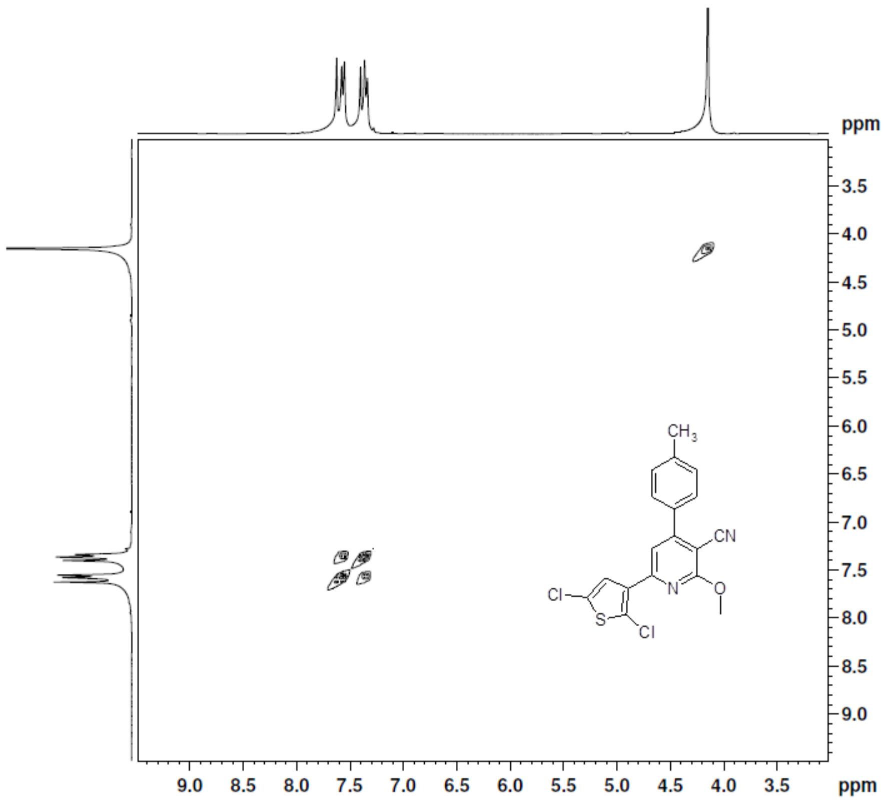

S 13: H,H COSY spectrum ($CDCl_3$, 300 MHz) of 6-(2,5-dichlorothiophen-3-yl)-2-methoxy-4-*p*-tolylpyridine-3-carbonitrile (**5b**).

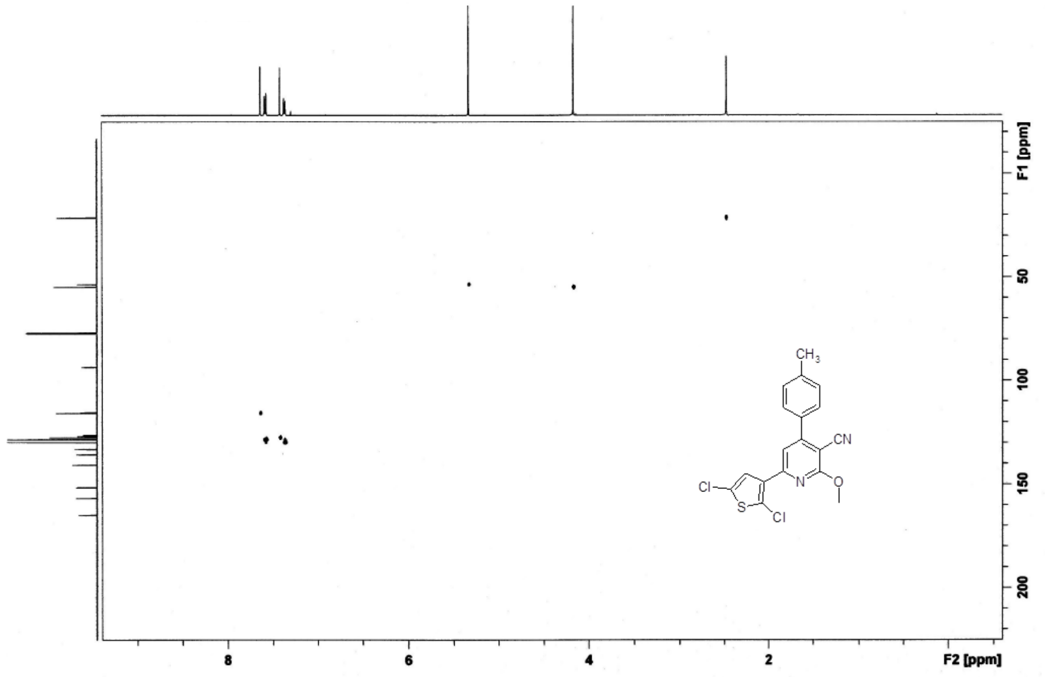

S 14: HSQC spectrum ($CDCl_3$, 500 MHz) of 6-(2,5-dichlorothiophen-3-yl)-2-methoxy-4-*p*-tolylpyridine-3-carbonitrile (**5b**).

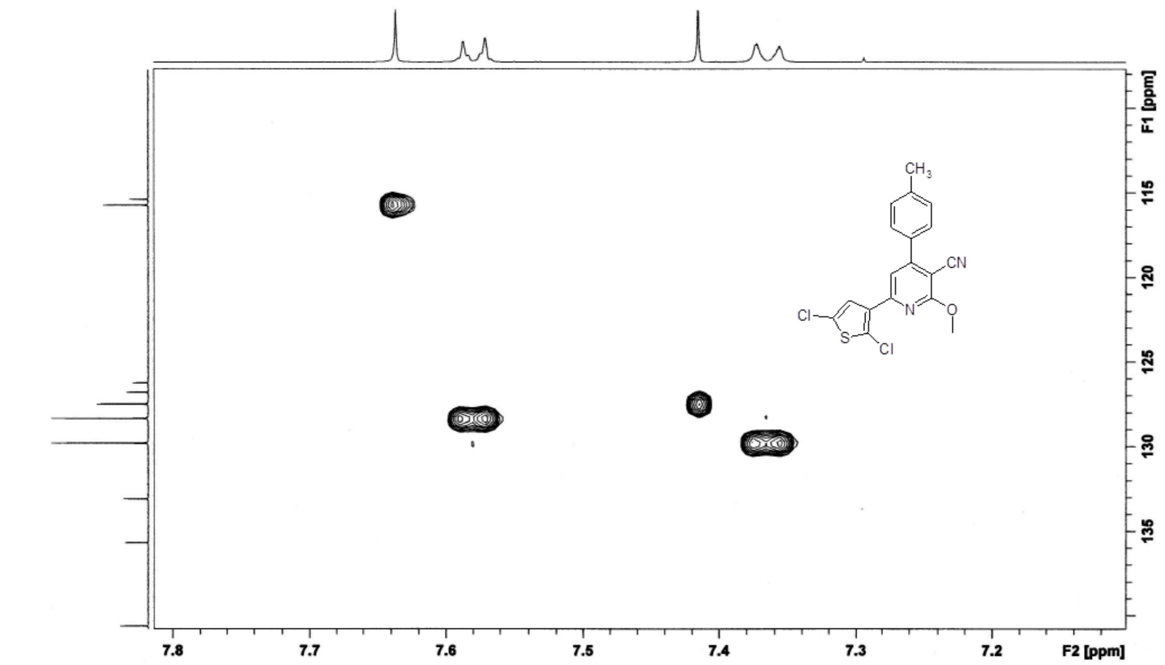

S 15: HSQC spectrum ($CDCl_3$, 500 MHz) of 6-(2,5-dichlorothiophen-3-yl)-2-methoxy-4-*p*-tolylpyridine-3-carbonitrile (**5b**).

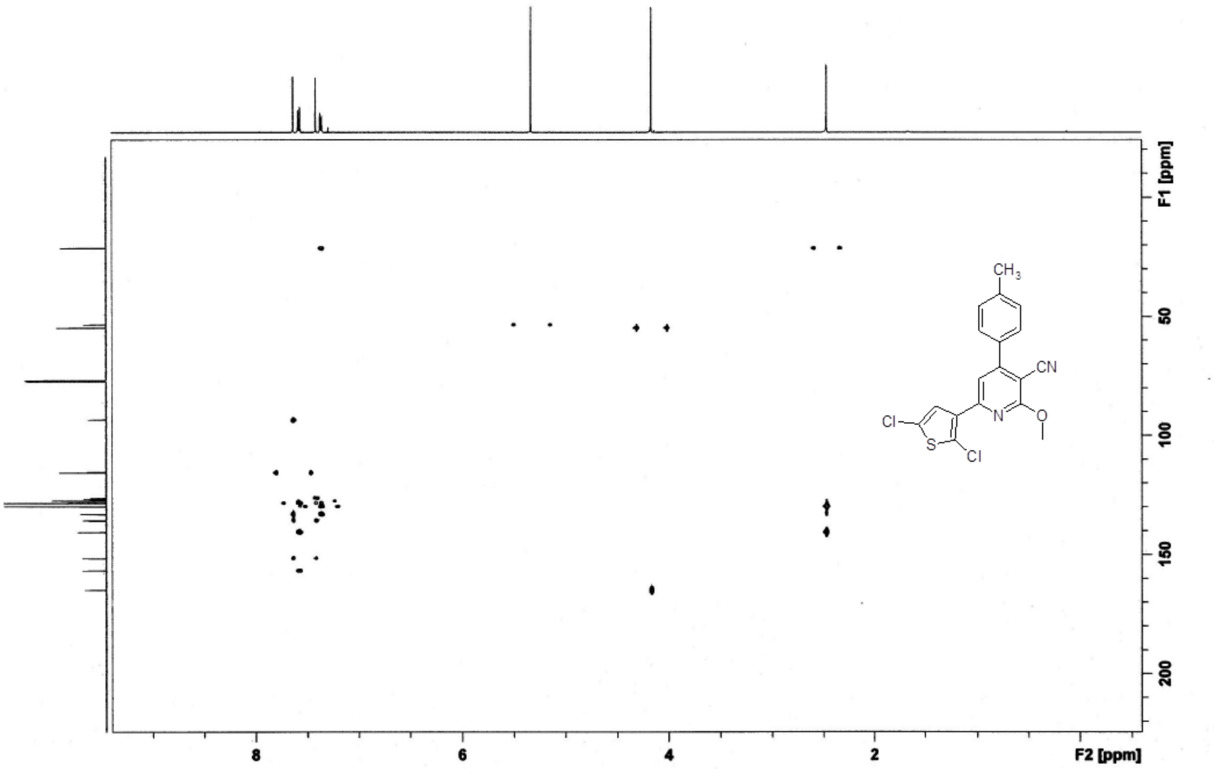

S 16: HMBC spectrum ($CDCl_3$, 500 MHz) of 6-(2,5-dichlorothiophen-3-yl)-2-methoxy-4-*p*-tolylpyridine-3-carbonitrile (**5b**).

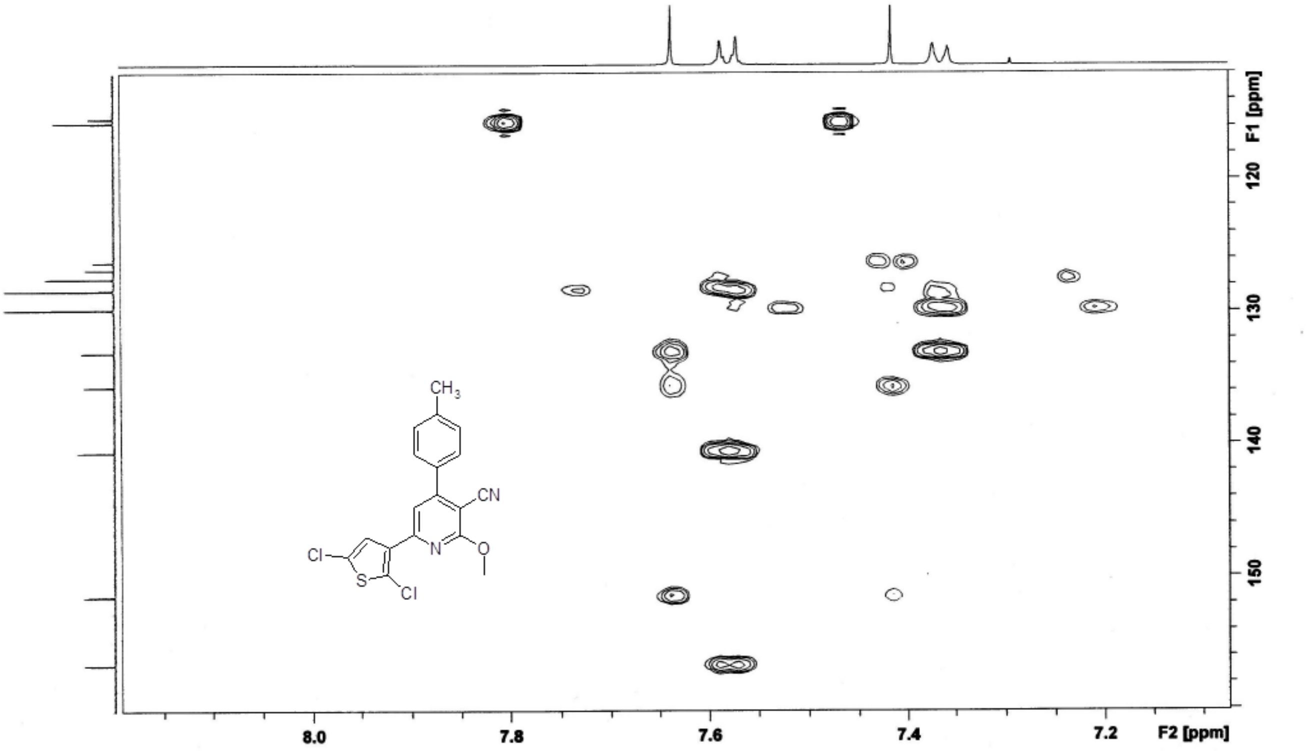

S 17: HMBC spectrum ($CDCl_3$, 500 MHz) of 6-(2,5-dichlorothiophen-3-yl)-2-methoxy-4-*p*-tolylpyridine-3-carbonitrile (**5b**).

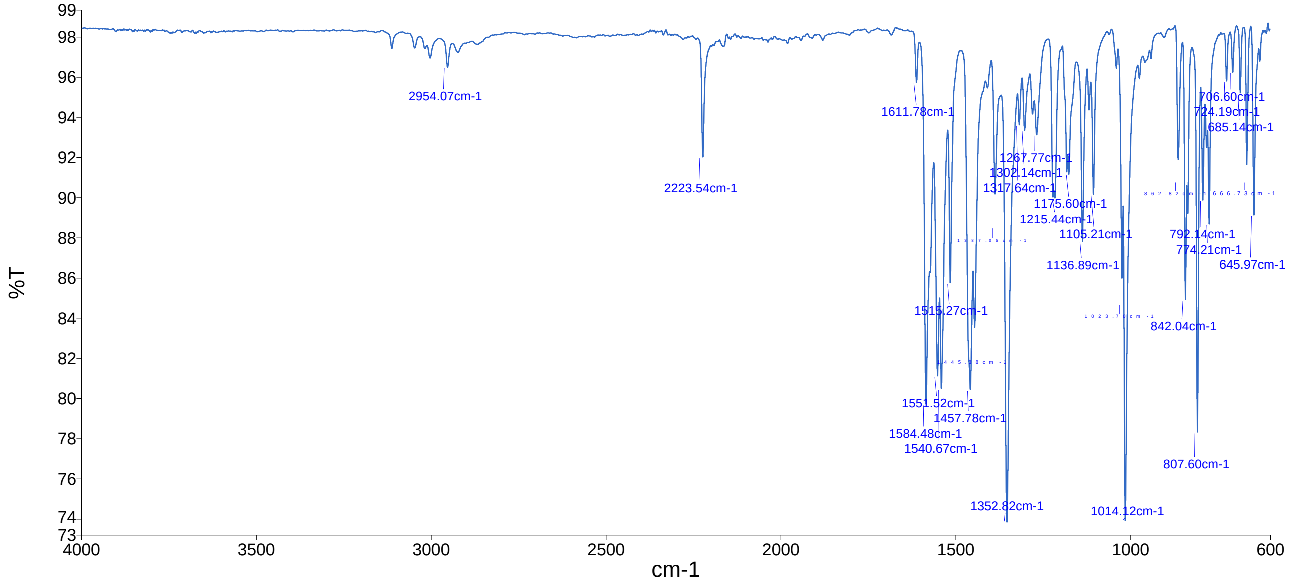

S 18: IR spectrum of 6-(2,5-dichlorothiophen-3-yl)-2-methoxy-4-*p*-tolylpyridine-3-carbonitrile (**5b**).

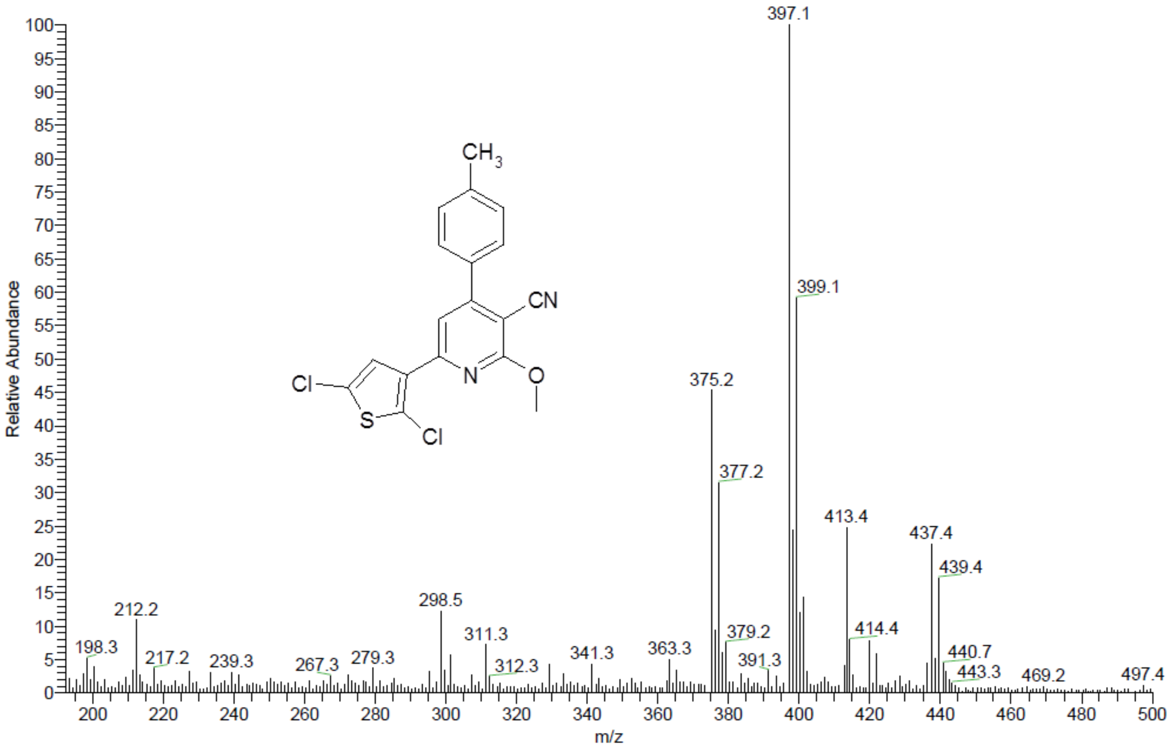

S 19: ESI mass spectrum of 6-(2,5-dichlorothiophen-3-yl)-2-methoxy-4-*p*-tolylpyridine-3-carbonitrile (**5b**).

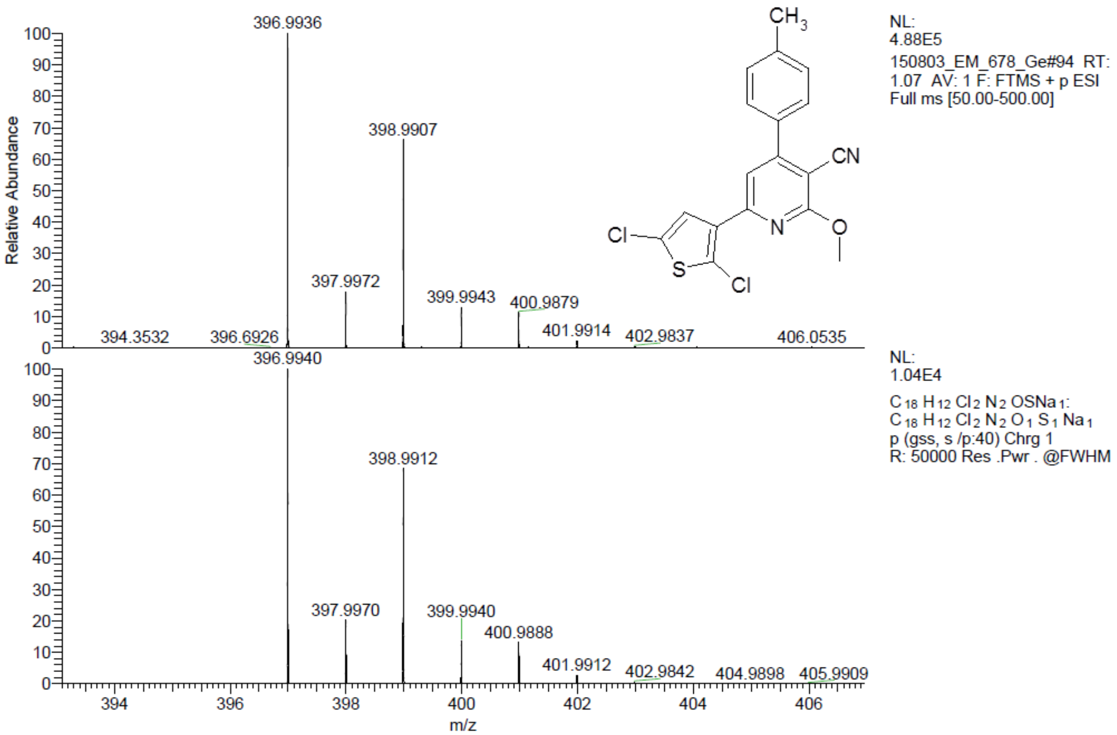

S 20: HRESI mass spectrum of 6-(2,5-dichlorothiophen-3-yl)-2-methoxy-4-*p*-tolylpyridine-3-carbonitrile (**5b**).

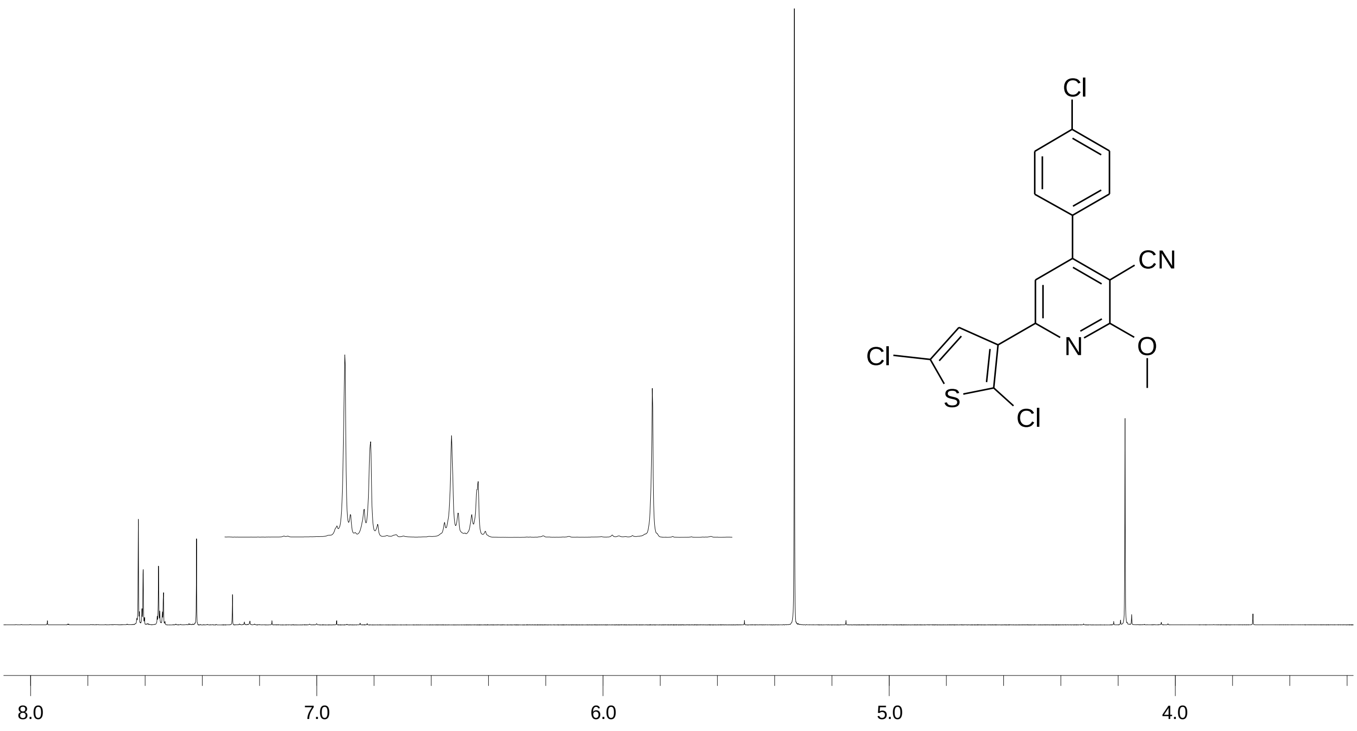

S 21: ^{1}H NMR spectrum ($CDCl_3$, 500 MHz) of 4-(4-chlorophenyl)-6-(2,5-dichlorothiophen-3-yl)-2-methoxypyridine-3-carbonitrile (**5c**).

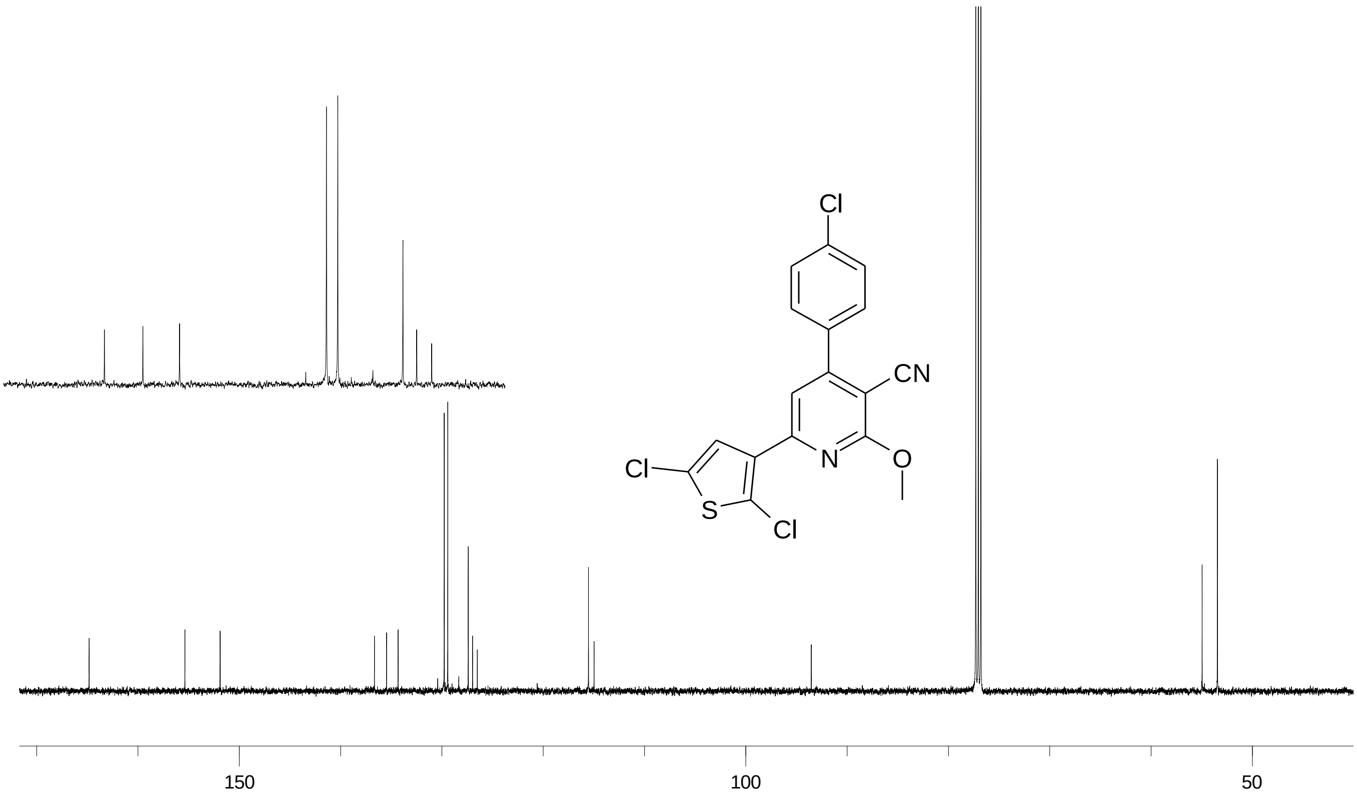

S 22: ^{13}C NMR spectrum ($CDCl_3$, 125MHz) of 4-(4-chlorophenyl)-6-(2,5-dichlorothiophen-3-yl)-2-methoxypyridine-3-carbonitrile (**5c**).

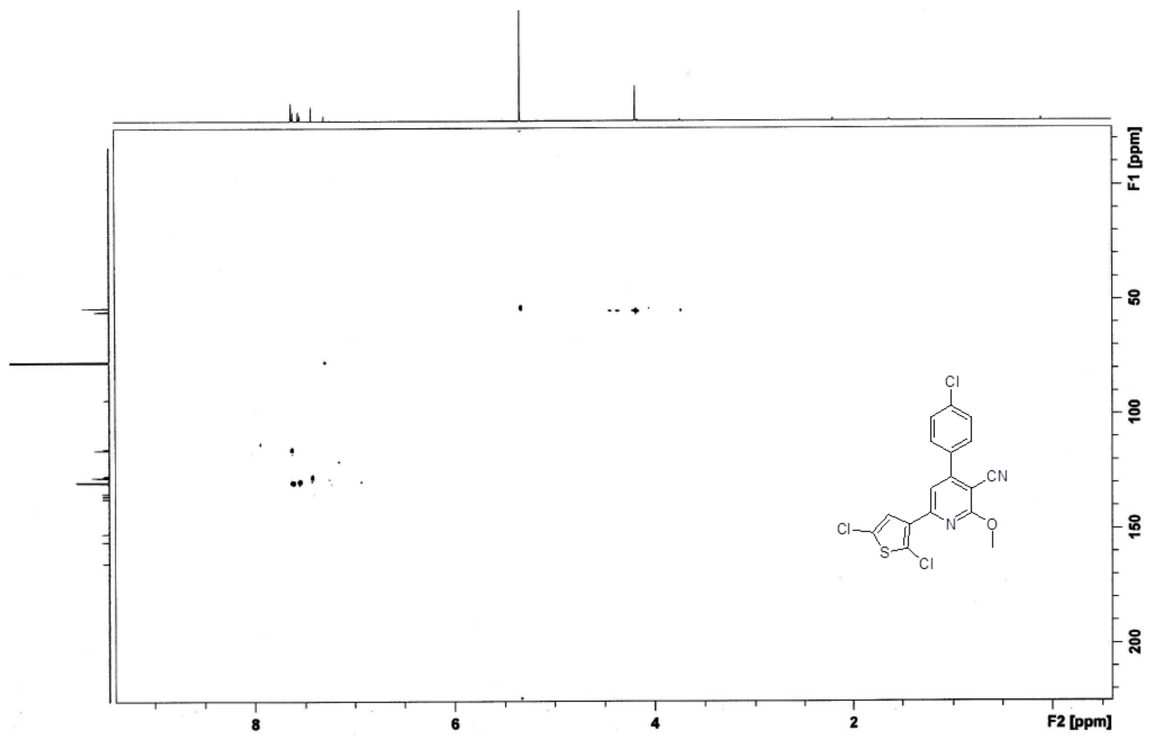

S 23: HSQC spectrum ($CDCl_3$, 500 MHz) of 4-(4-chlorophenyl)-6-(2,5-dichlorothiophen-3-yl)-2-methoxypyridine-3-carbonitrile (**5c**).

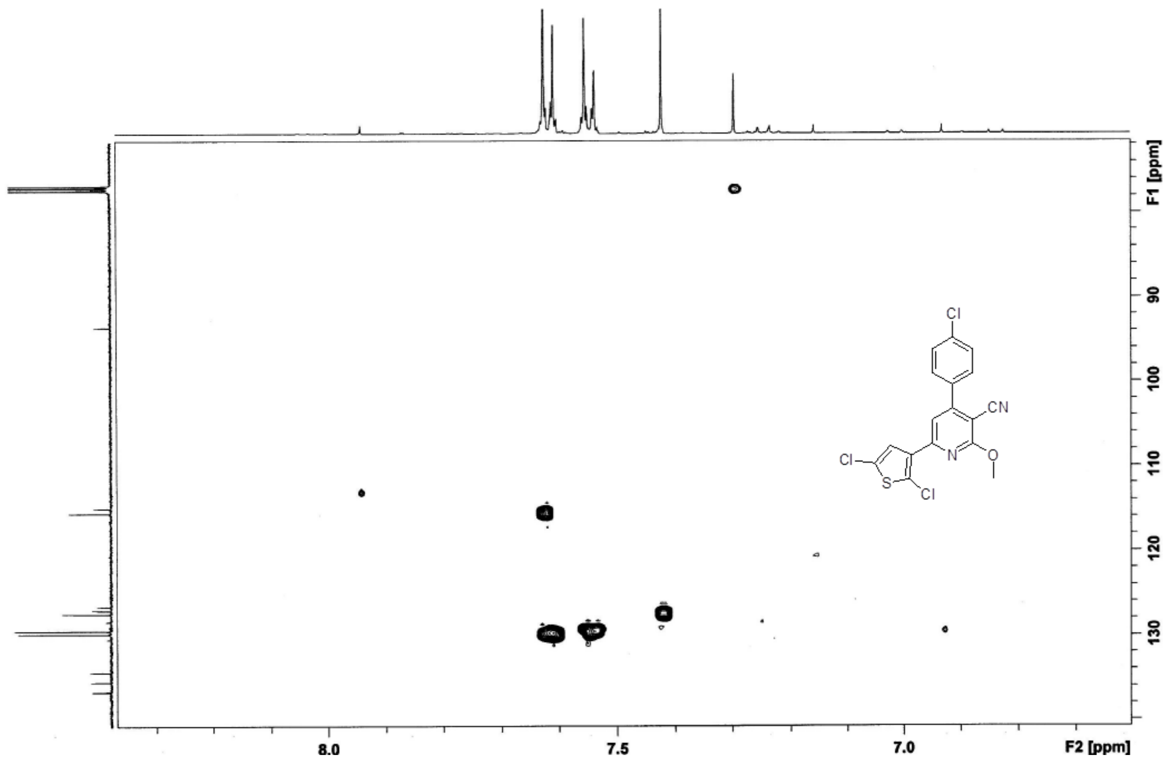

S 24: HSQC spectrum ($CDCl_3$, 500 MHz) of 4-(4-chlorophenyl)-6-(2,5-dichlorothiophen-3-yl)-2-methoxypyridine-3-carbonitrile (**5c**).

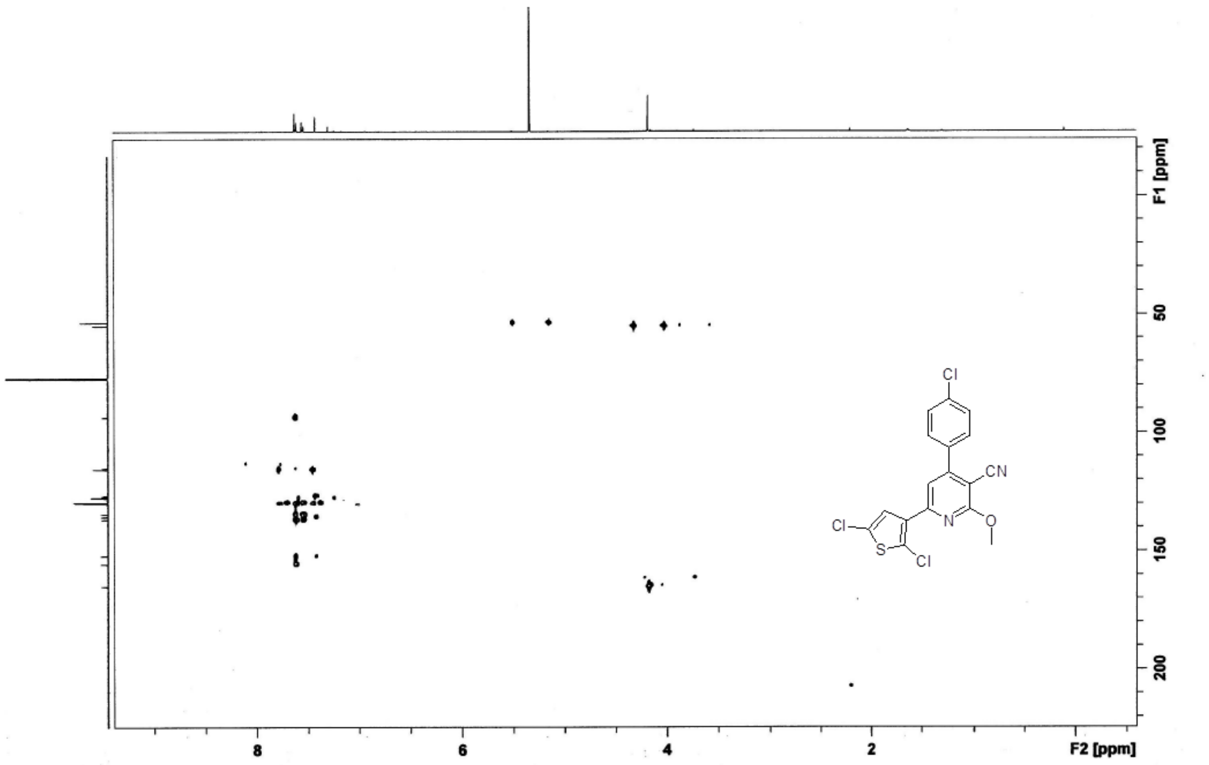

S 25: HMBC spectrum ($CDCl_3$, 500 MHz) of 4-(4-chlorophenyl)-6-(2,5-dichlorothiophen-3-yl)-2-methoxypyridine-3-carbonitrile (**5c**).

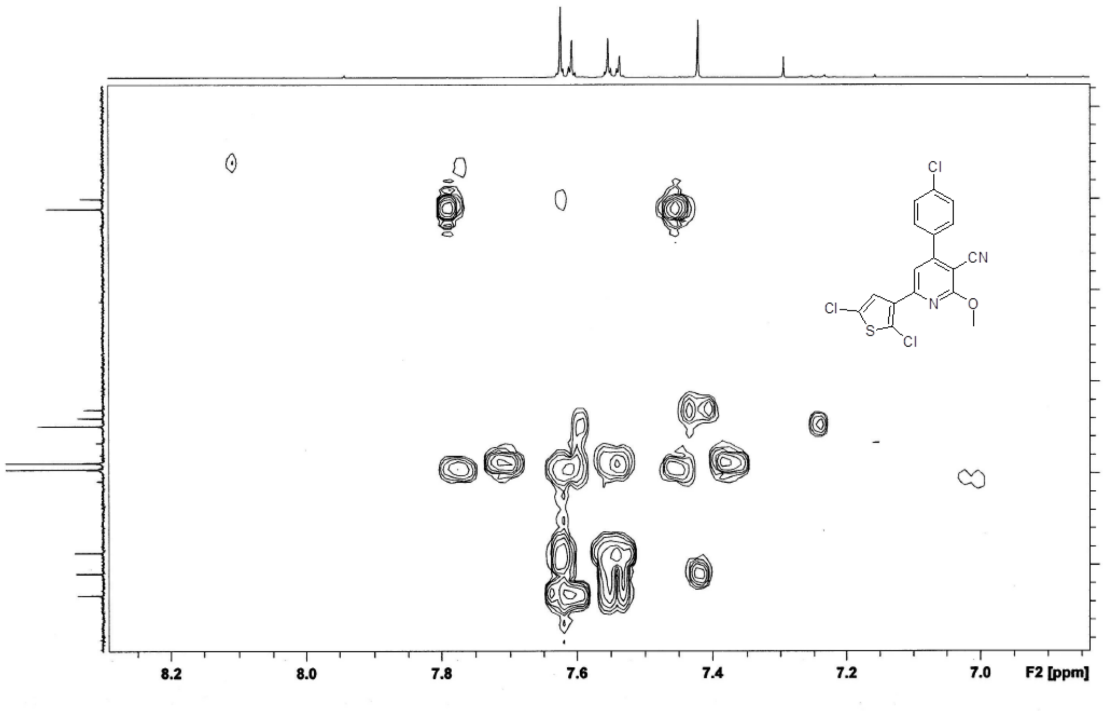

S 26: HMBC spectrum ($CDCl_3$, 500 MHz) of 4-(4-chlorophenyl)-6-(2,5-dichlorothiophen-3-yl)-2-methoxypyridine-3-carbonitrile (**5c**).

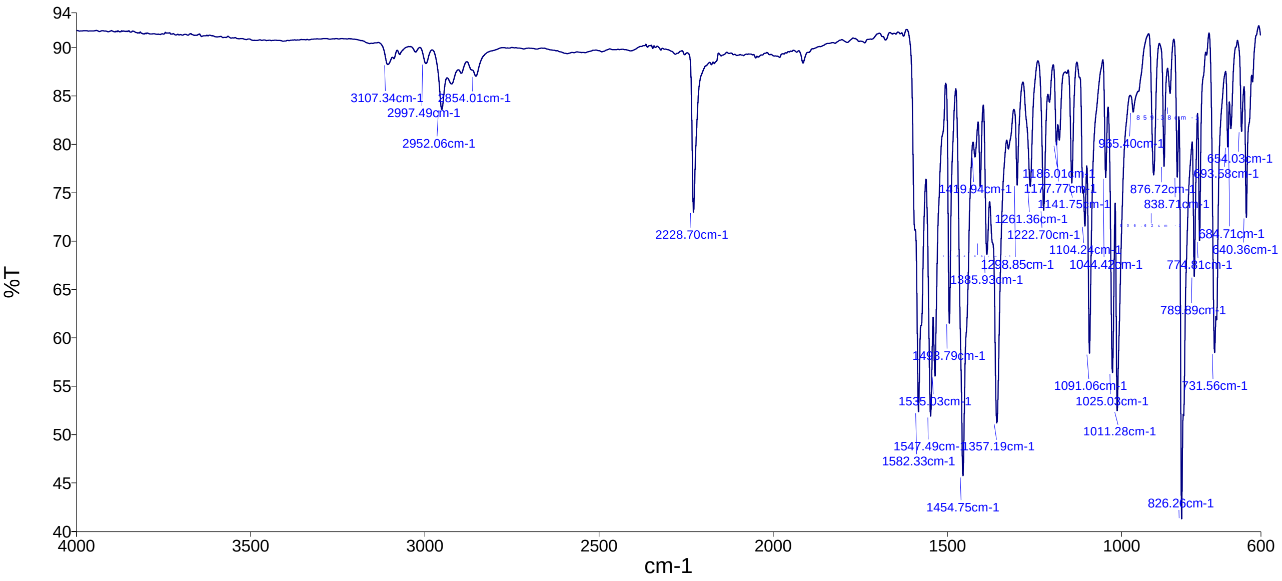

S 27: IR spectrum of 4-(4-chlorophenyl)-6-(2,5-dichlorothiophen-3-yl)-2-methoxypyridine-3-carbonitrile (**5c**).

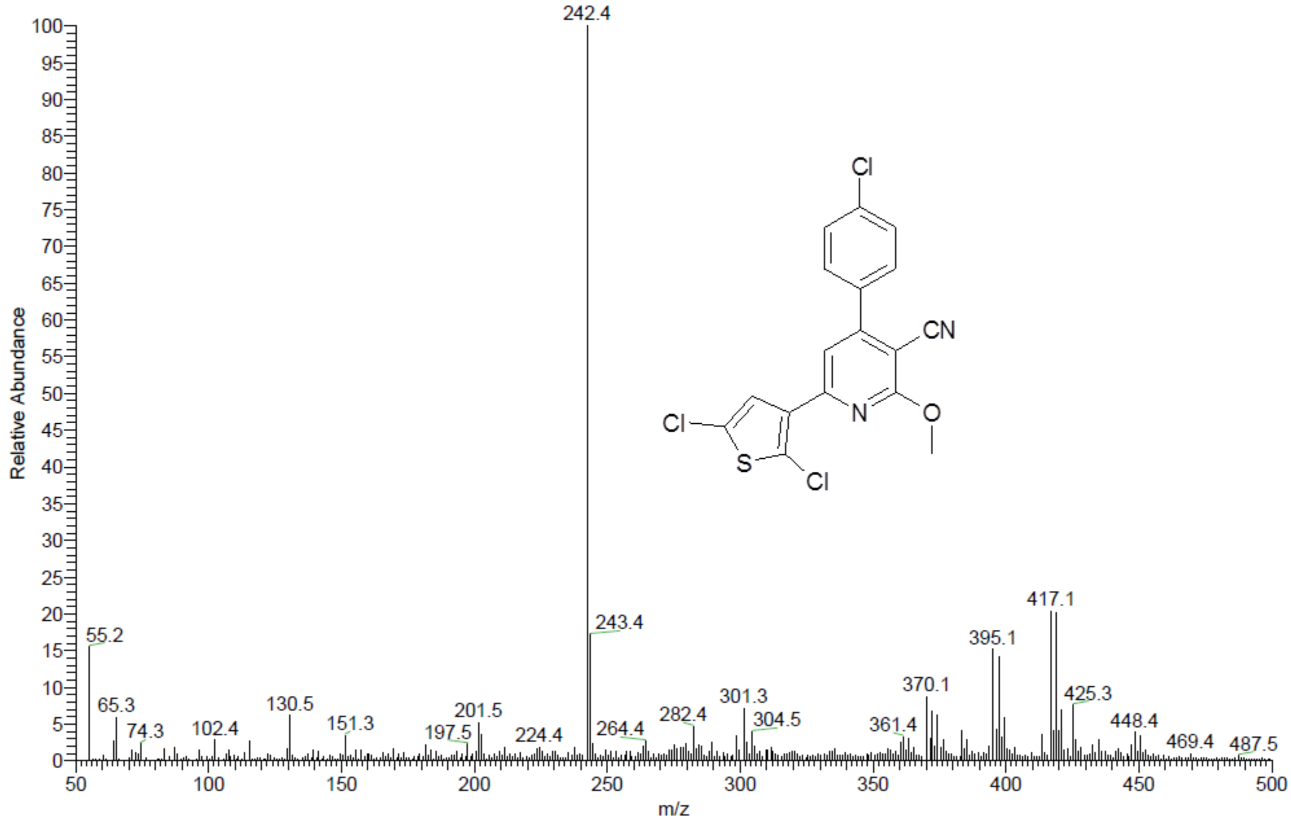

S 28: ESI mass spectrum of 4-(4-chlorophenyl)-6-(2,5-dichlorothiophen-3-yl)-2-methoxypyridine-3-carbonitrile (**5c**).

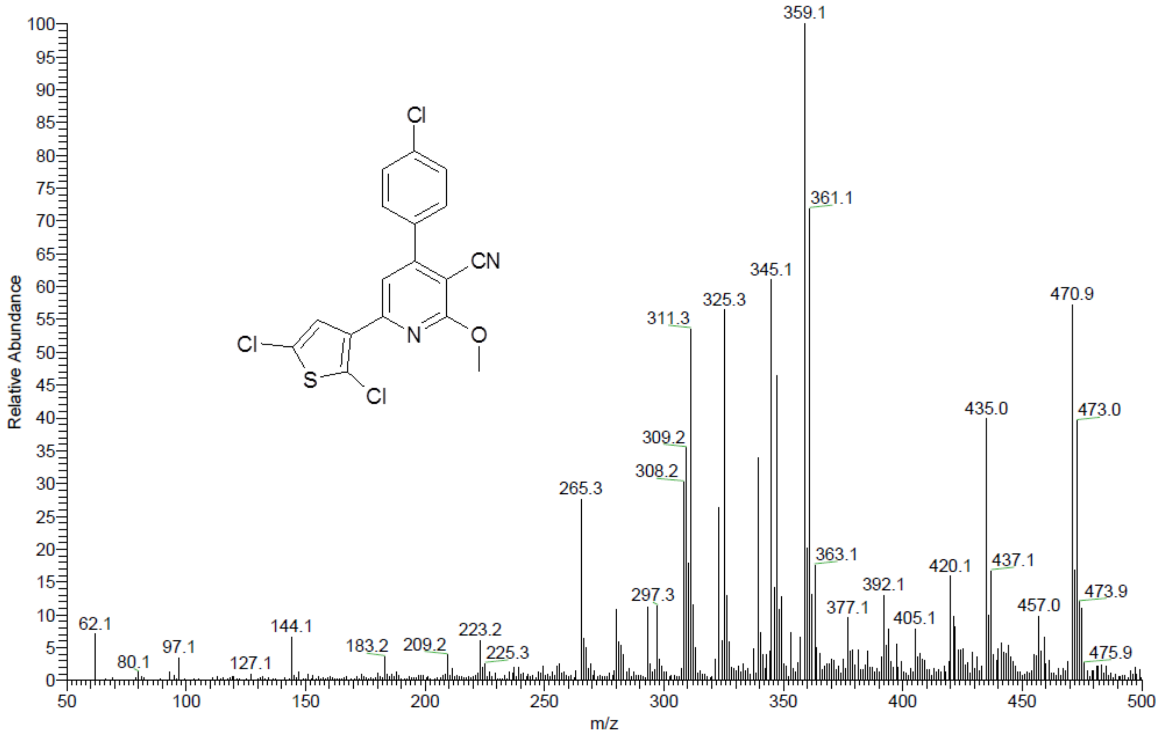

S 29: ESI mass spectrum of 4-(4-chlorophenyl)-6-(2,5-dichlorothiophen-3-yl)-2-methoxypyridine-3-carbonitrile (**5c**).

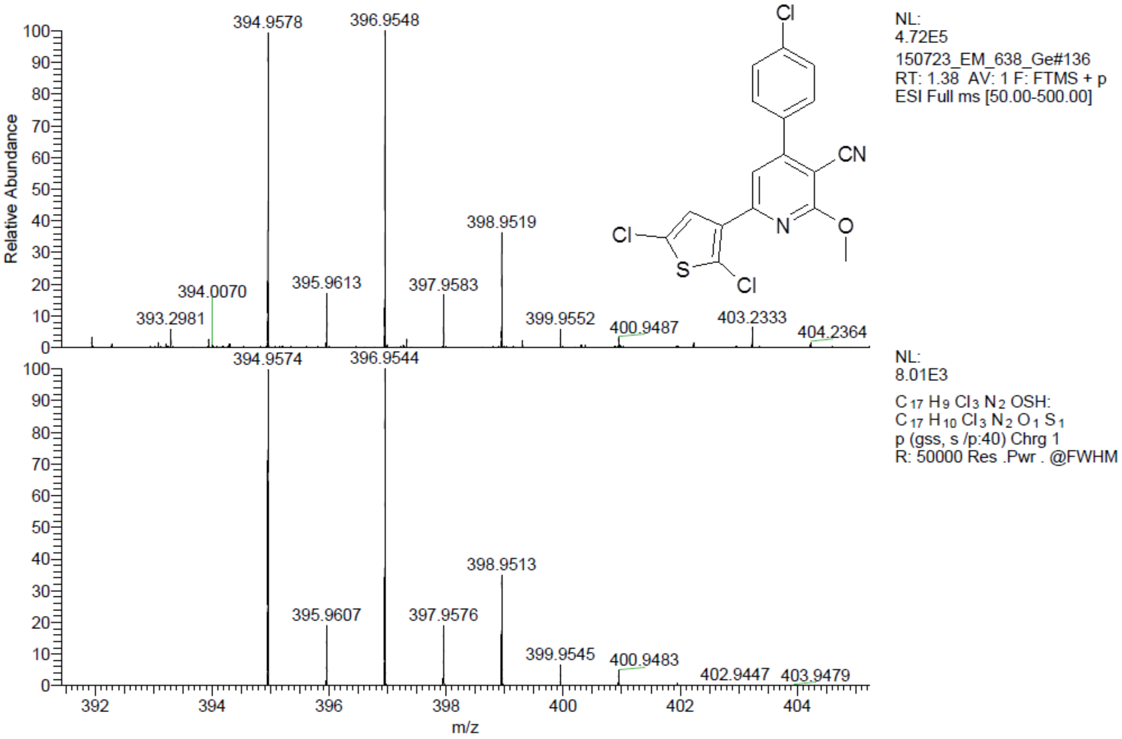

S 30: HRESI mass spectrum of 4-(4-chlorophenyl)-6-(2,5-dichlorothiophen-3-yl)-2-methoxypyridine-3-carbonitrile (**5c**).

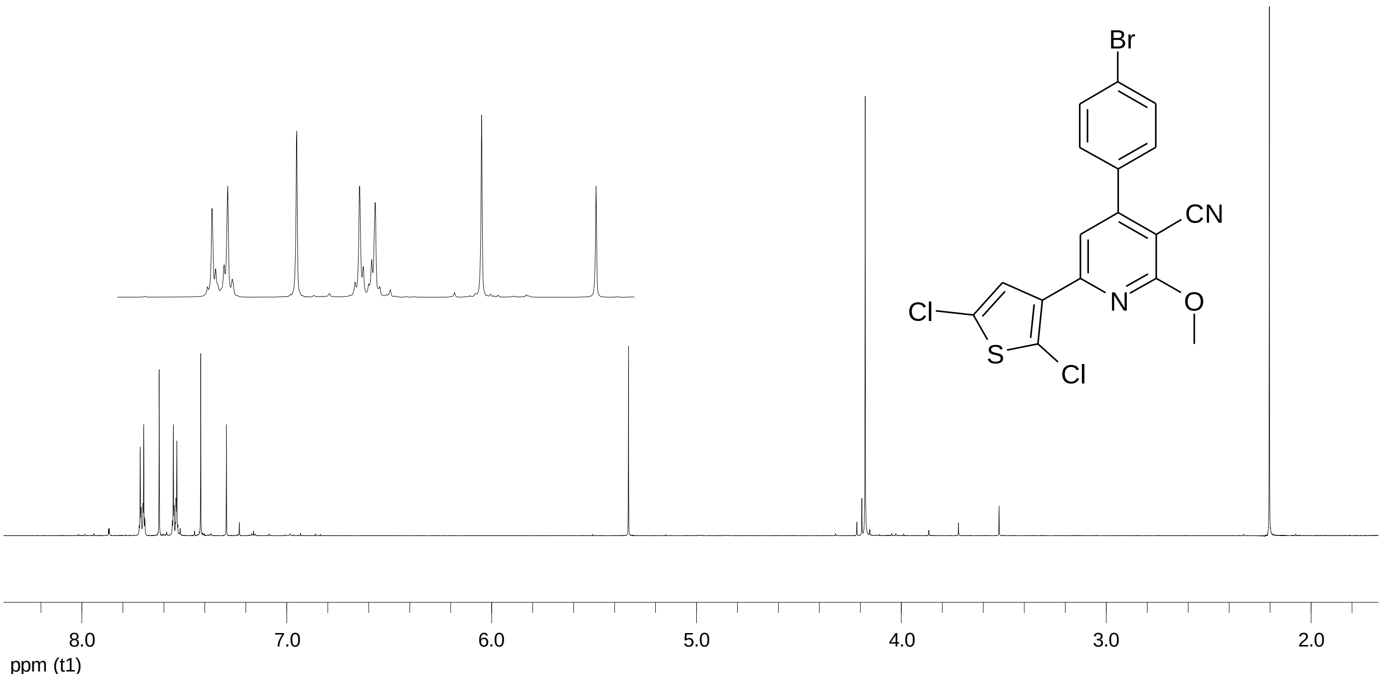

S 31: ^{1}H NMR spectrum ($CDCl_3$, 500 MHz) of 4-(4-bromophenyl)-6-(2,5-dichlorothiophen-3-yl)-2-methoxypyridine-3-carbonitrile (**5d**).

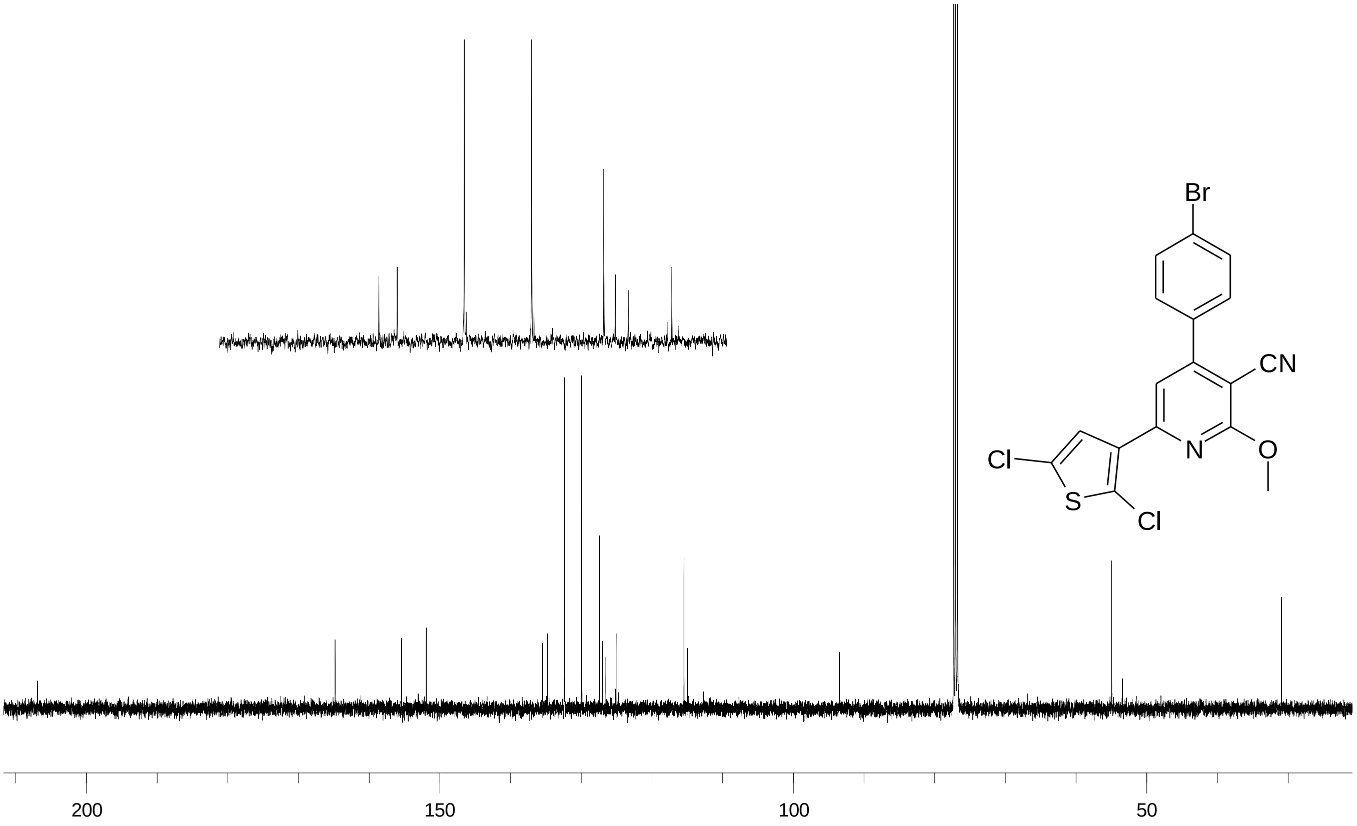

S 32: ^{13}C NMR spectrum ($CDCl_3$, 125MHz) of 4-(4-bromophenyl)-6-(2,5-dichlorothiophen-3-yl)-2-methoxypyridine-3-carbonitrile (**5d**).

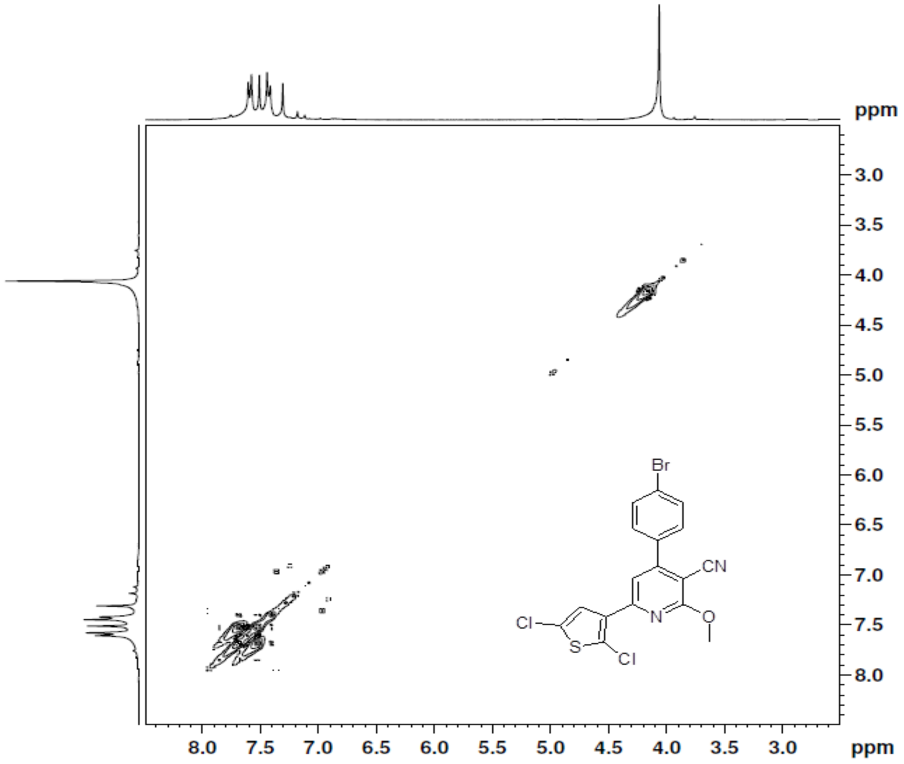

S 33: H,H COSY spectrum ($CDCl_3$, 300 MHz) of 4-(4-bromophenyl)-6-(2,5-dichlorothiophen-3-yl)-2-methoxypyridine-3-carbonitrile (**5d**).

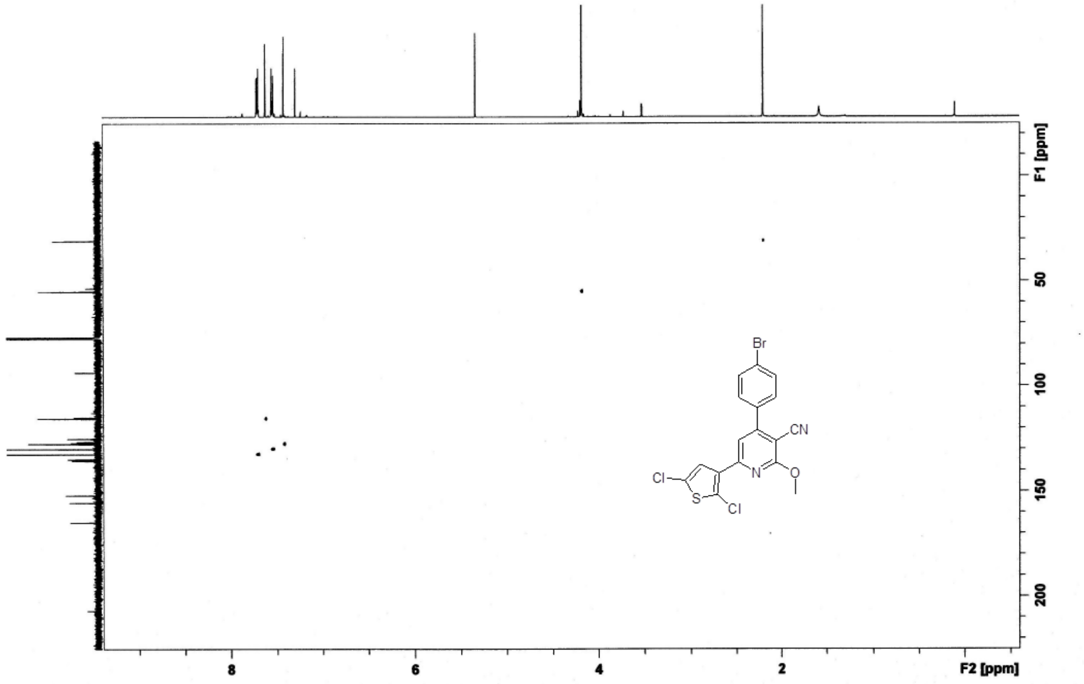

S 34: HSQC spectrum ($CDCl_3$, 500 MHz) of 4-(4-bromophenyl)-6-(2,5-dichlorothiophen-3-yl)-2-methoxypyridine-3-carbonitrile (**5d**).

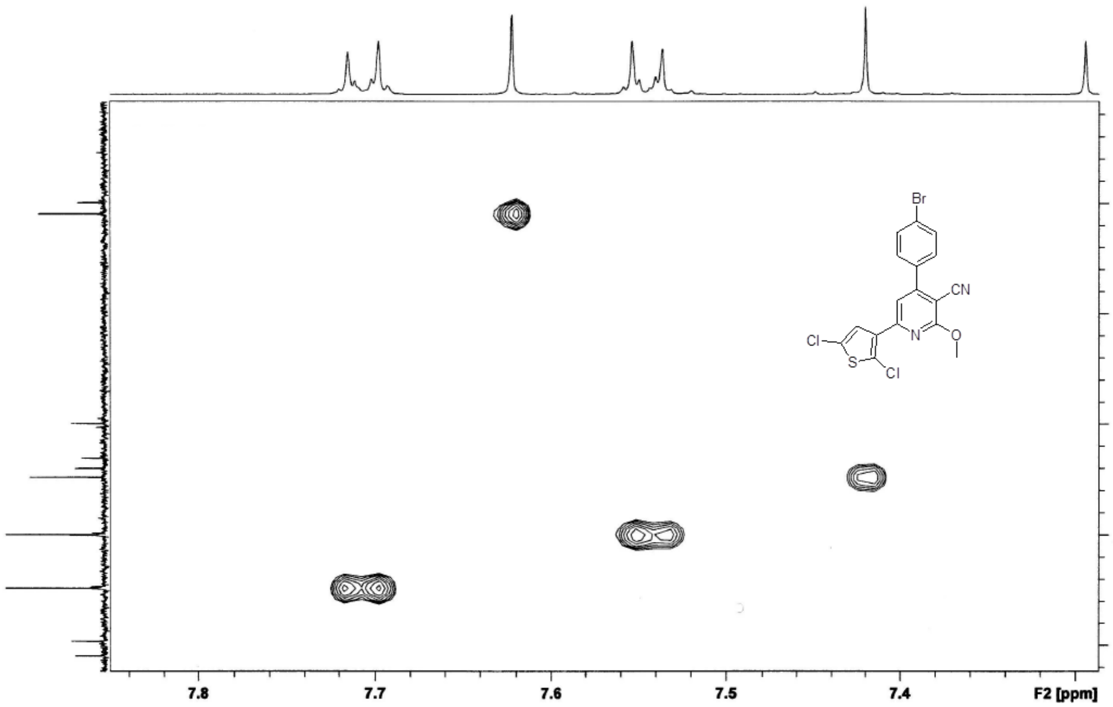

S 35: HSQC spectrum ($CDCl_3$, 500 MHz) of 4-(4-bromophenyl)-6-(2,5-dichlorothiophen-3-yl)-2-methoxypyridine-3-carbonitrile (**5d**).

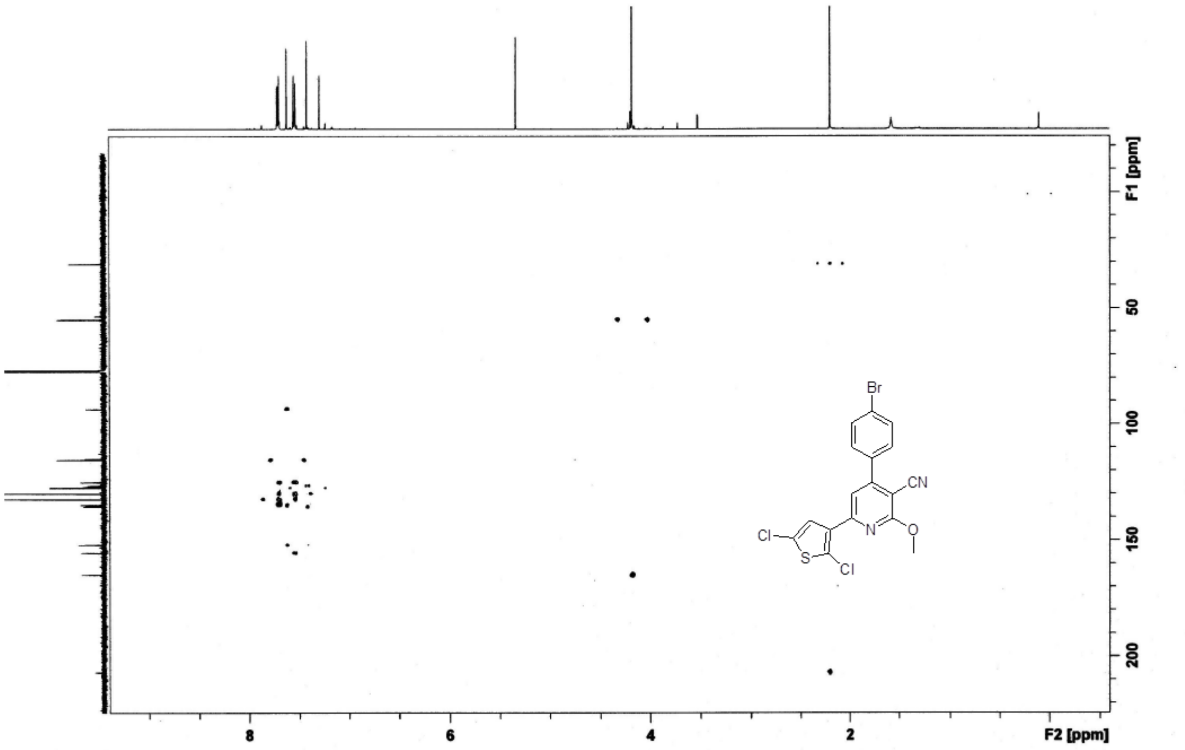

S 36: HMBC spectrum ($CDCl_3$, 500 MHz) of 4-(4-bromophenyl)-6-(2,5-dichlorothiophen-3-yl)-2-methoxypyridine-3-carbonitrile (**5d**).

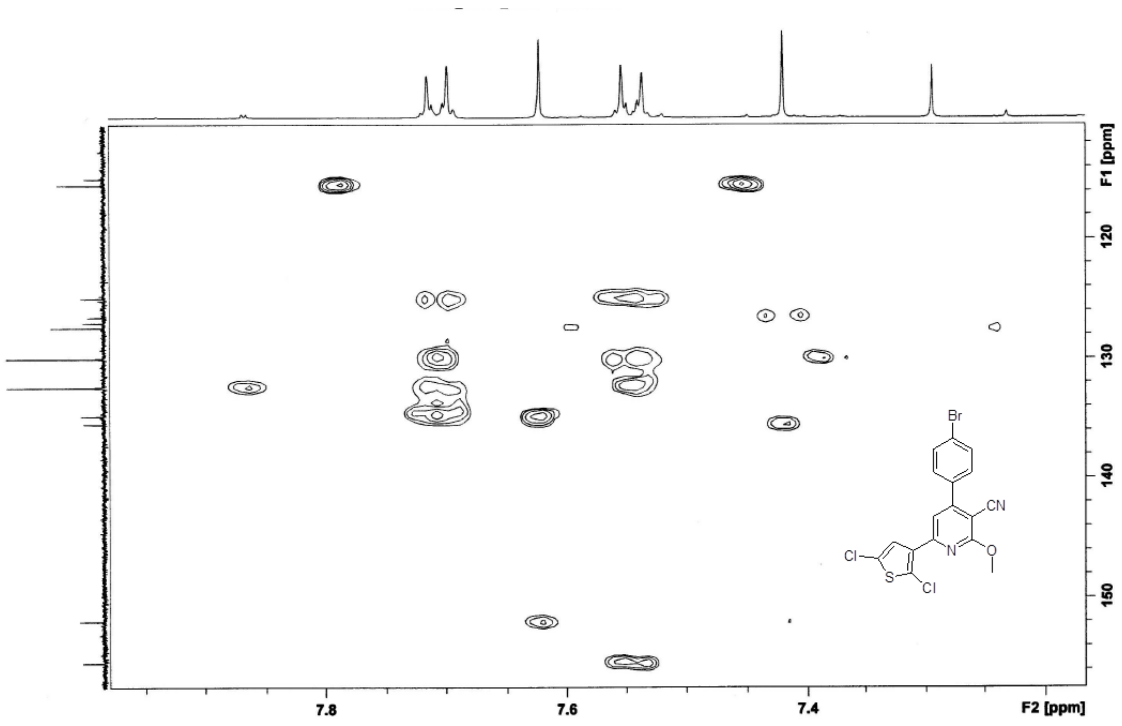

S 37: HMBC spectrum ($CDCl_3$, 500 MHz) of 4-(4-bromophenyl)-6-(2,5-dichlorothiophen-3-yl)-2-methoxypyridine-3-carbonitrile (**5d**).

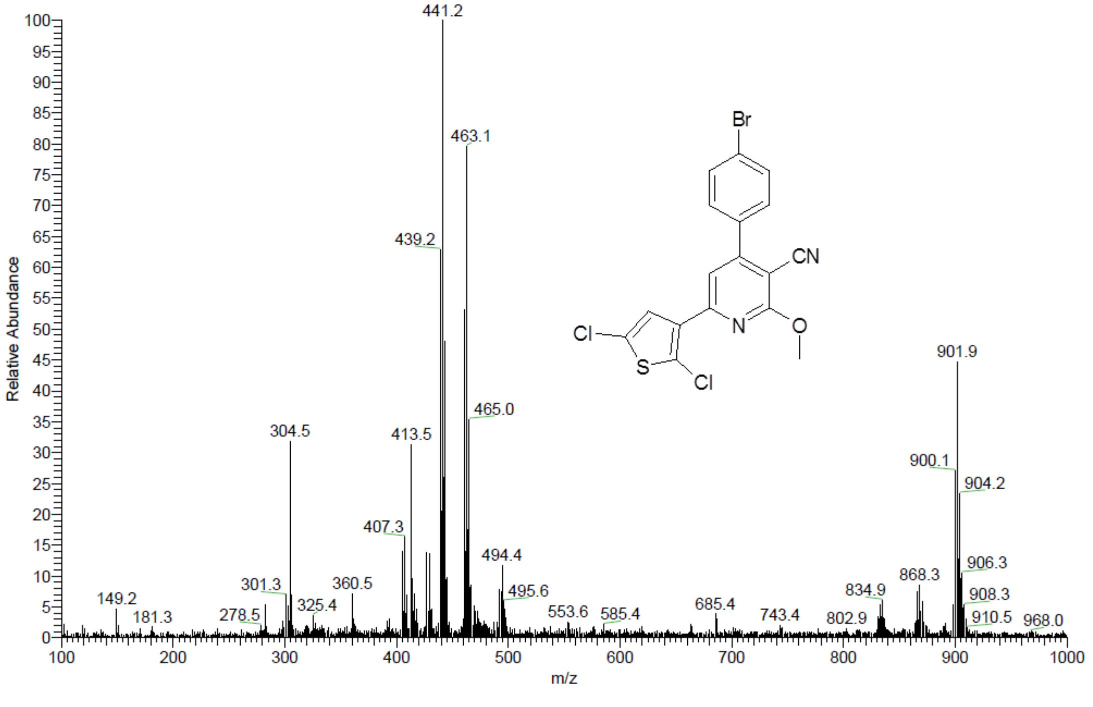

S 38: ESI mass spectrum of 4-(4-bromophenyl)-6-(2,5-dichlorothiophen-3-yl)-2-methoxypyridine-3-carbonitrile (**5d**).

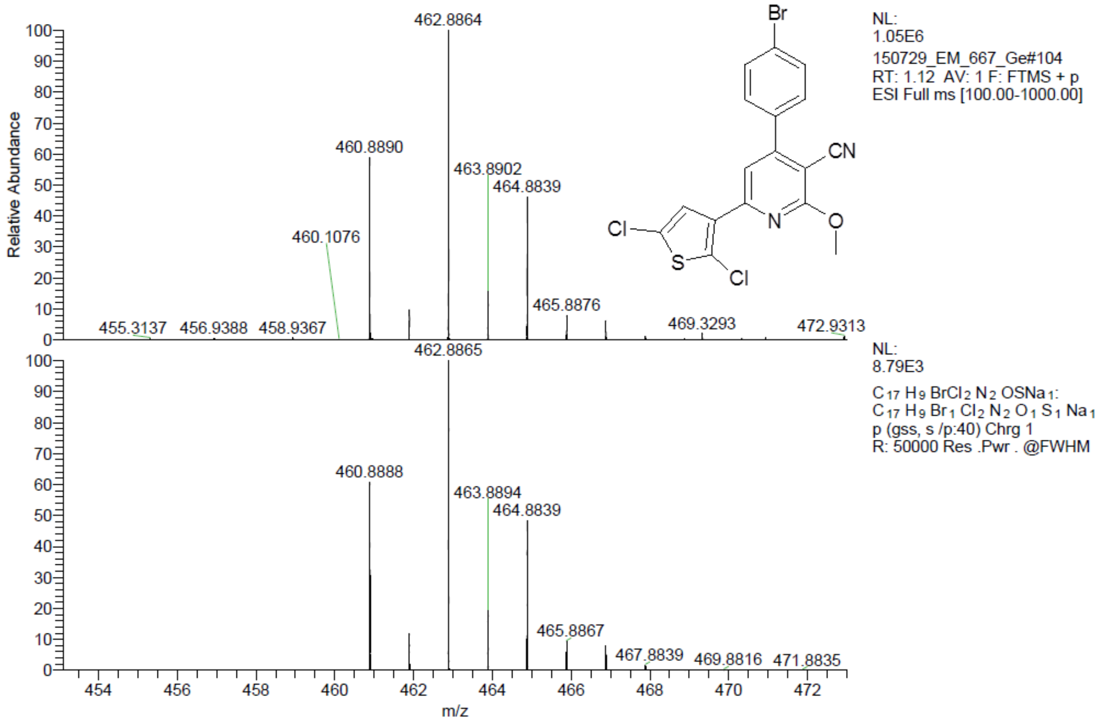

S 39: HRESI mass spectrum of 4-(4-bromophenyl)-6-(2,5-dichlorothiophen-3-yl)-2-methoxypyridine-3-carbonitrile (**5d**).

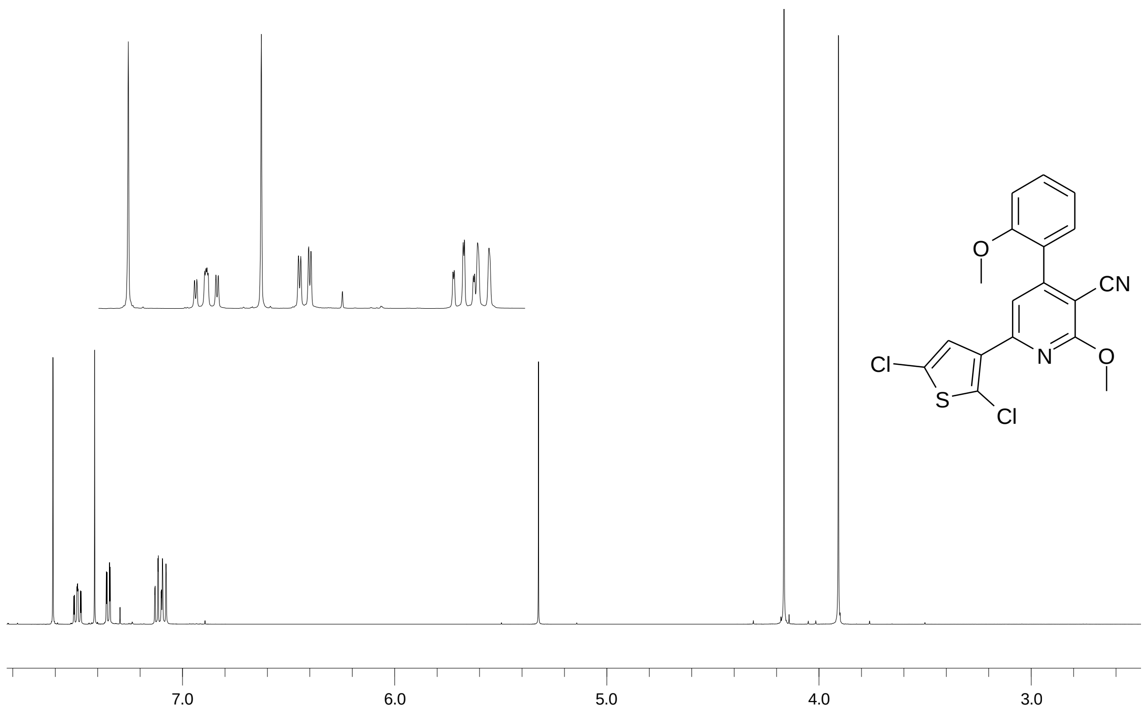

S 40: ^{1}H NMR spectrum ($CDCl_3$, 500 MHz) of 6-(2,5-dichlorothiophen-3-yl)-2-methoxy-4-(2-methoxyphenyl)pyridine-3-carbonitrile (**5e**).

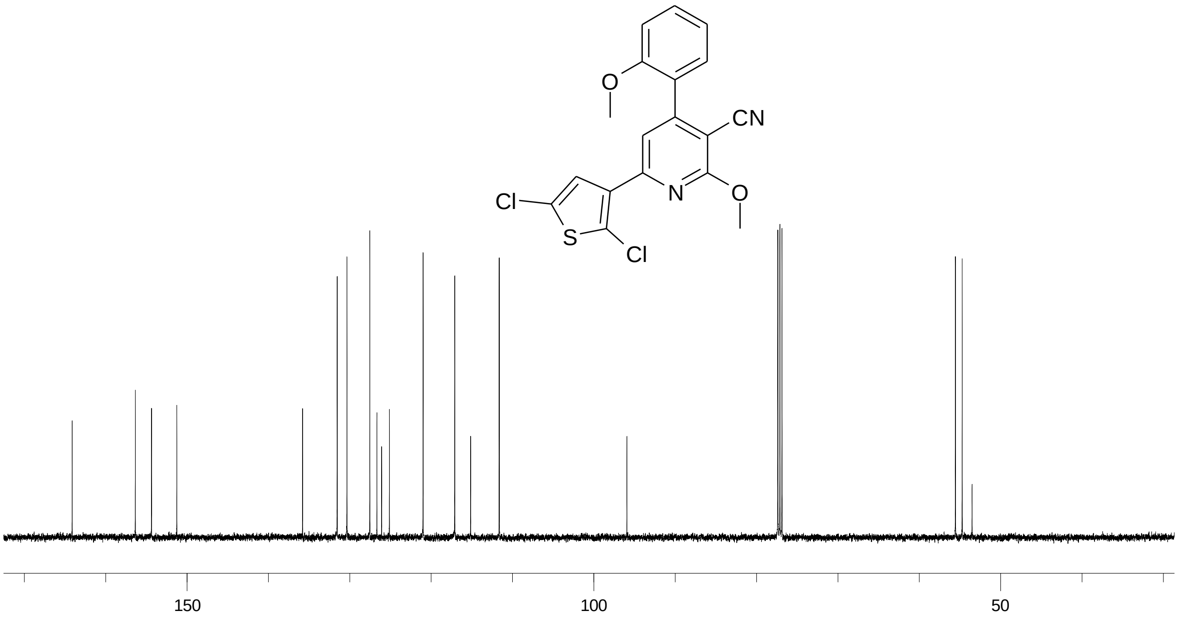

S 41: ^{13}C NMR spectrum ($CDCl_3$, 125MHz) of 6-(2,5-dichlorothiophen-3-yl)-2-methoxy-4-(2-methoxyphenyl)pyridine-3-carbonitrile (**5e**).

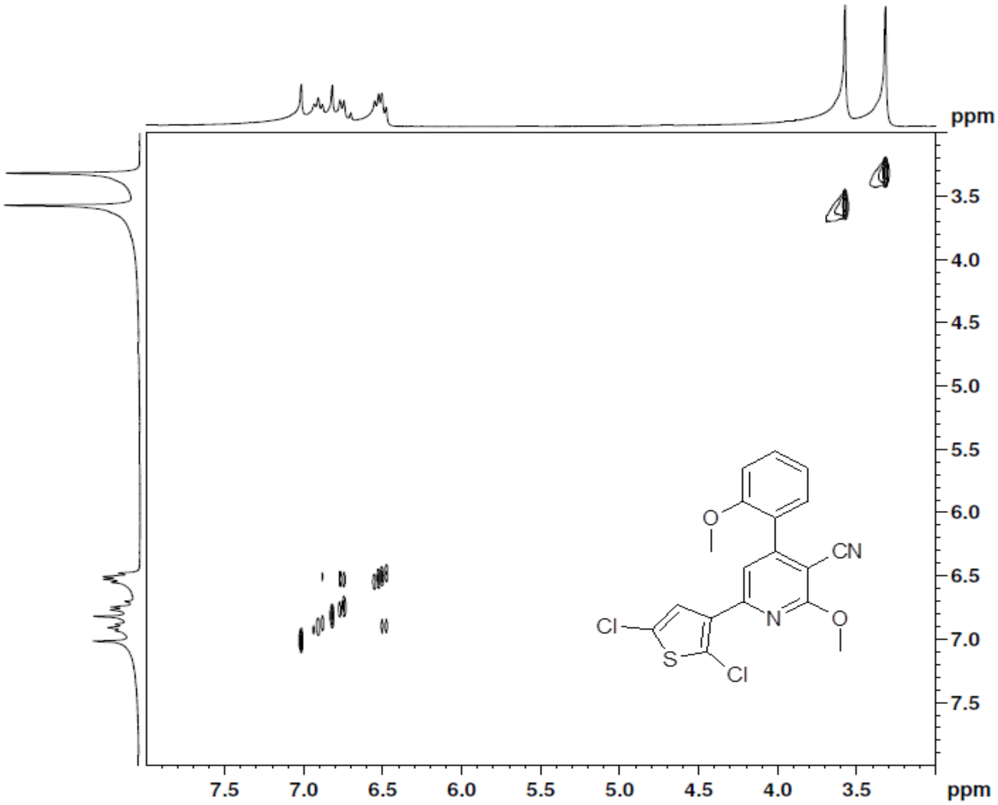

S 42: H,H COSY spectrum ($CDCl_3$, 300 MHz) of 6-(2,5-dichlorothiophen-3-yl)-2-methoxy-4-(2-methoxyphenyl)pyridine-3-carbonitrile (**5e**).

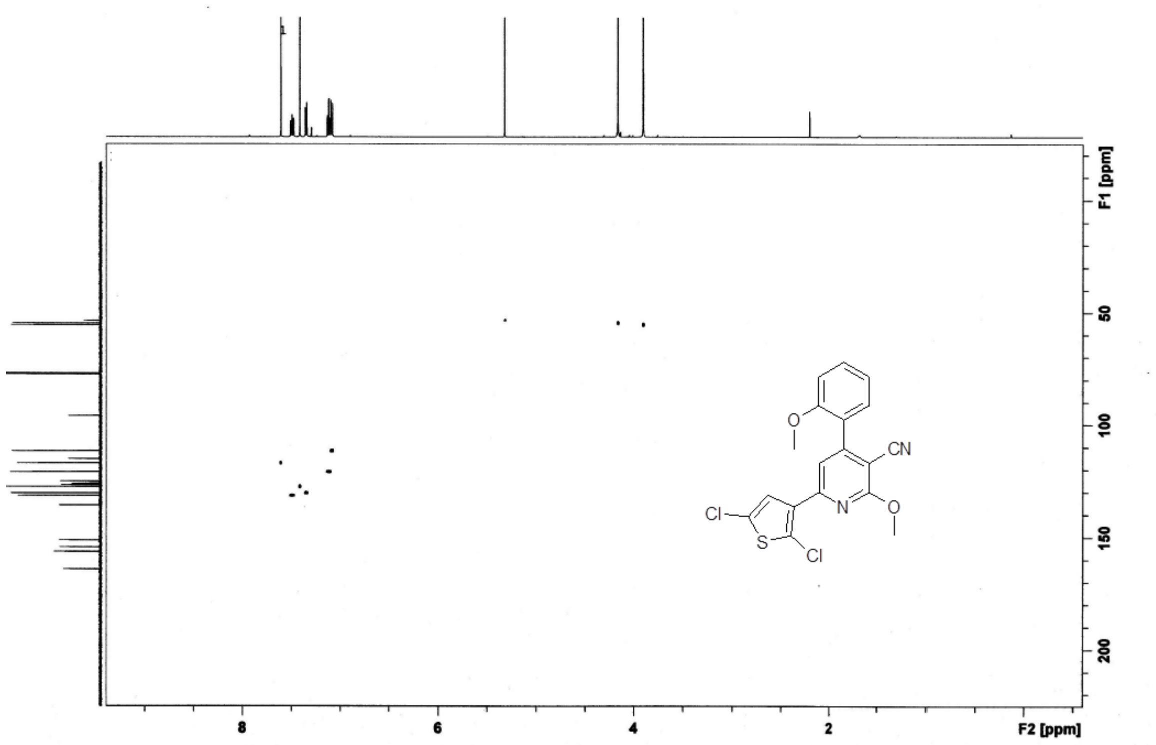

S 43: HSQC spectrum ($CDCl_3$, 500 MHz) of 6-(2,5-dichlorothiophen-3-yl)-2-methoxy-4-(2-methoxyphenyl)pyridine-3-carbonitrile (**5e**).

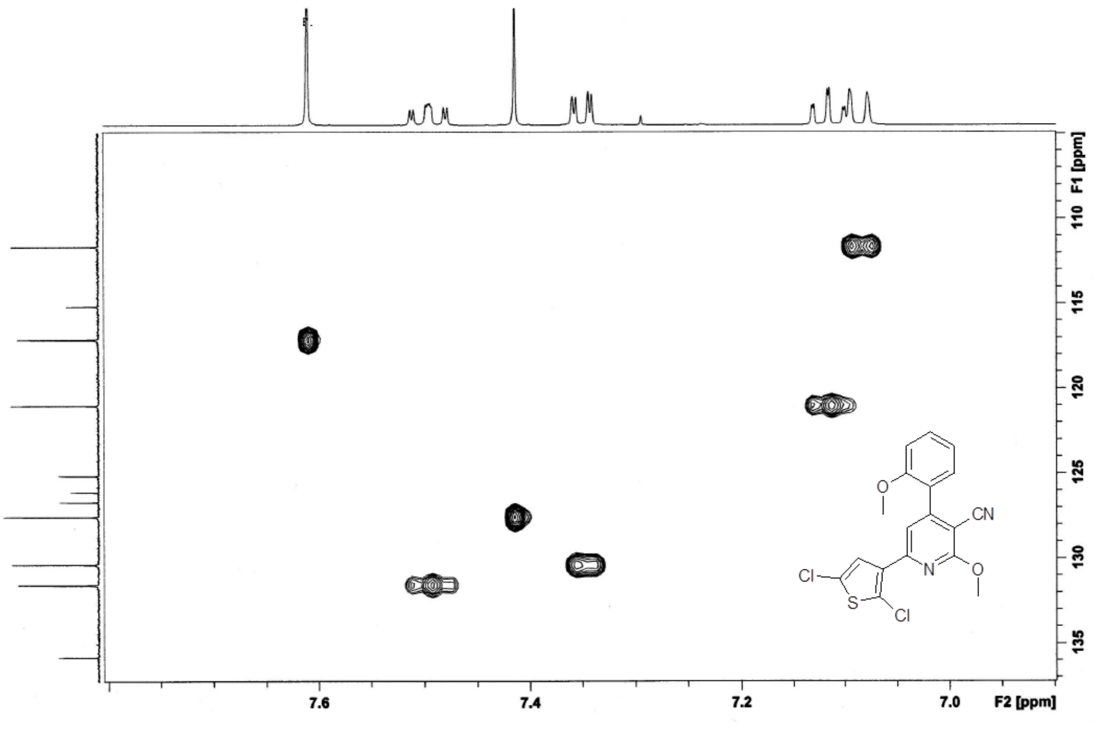

S 44: HSQC spectrum ($CDCl_3$, 500 MHz) of 6-(2,5-dichlorothiophen-3-yl)-2-methoxy-4-(2-methoxyphenyl)pyridine-3-carbonitrile (**5e**).

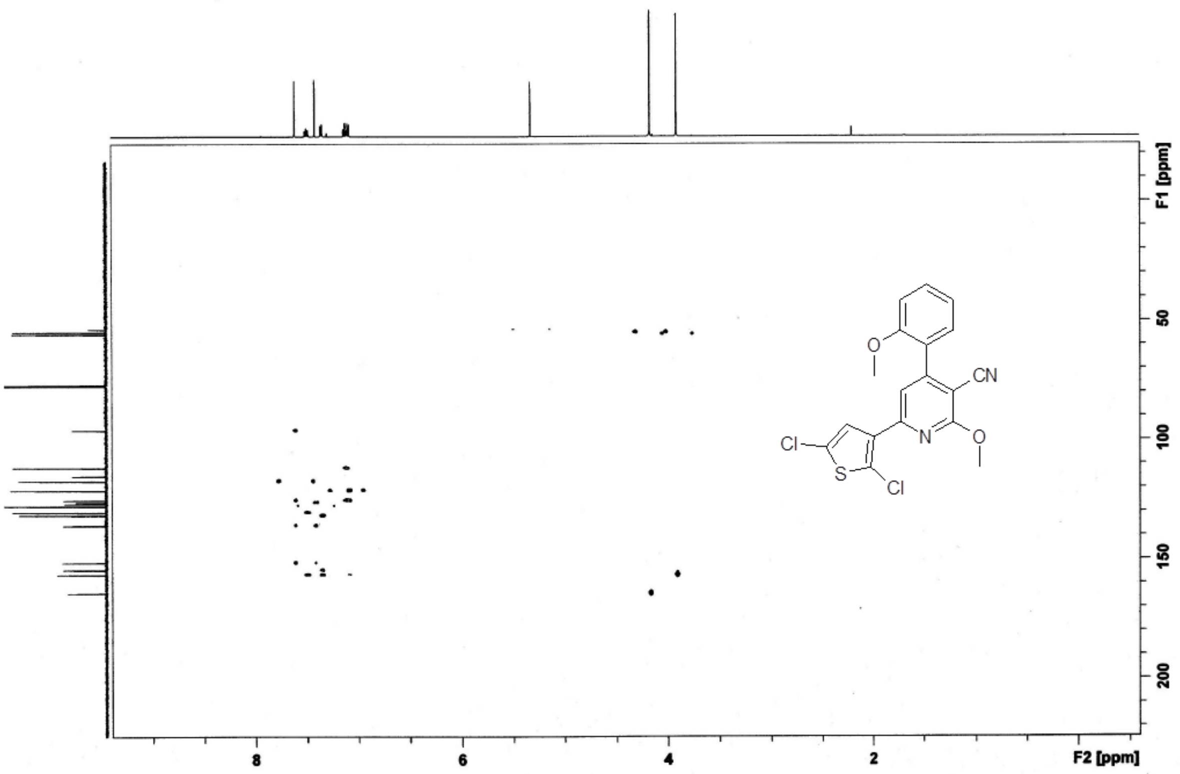

S 45: HMBC spectrum ($CDCl_3$, 500 MHz) of 6-(2,5-dichlorothiophen-3-yl)-2-methoxy-4-(2-methoxyphenyl)pyridine-3-carbonitrile (**5e**).

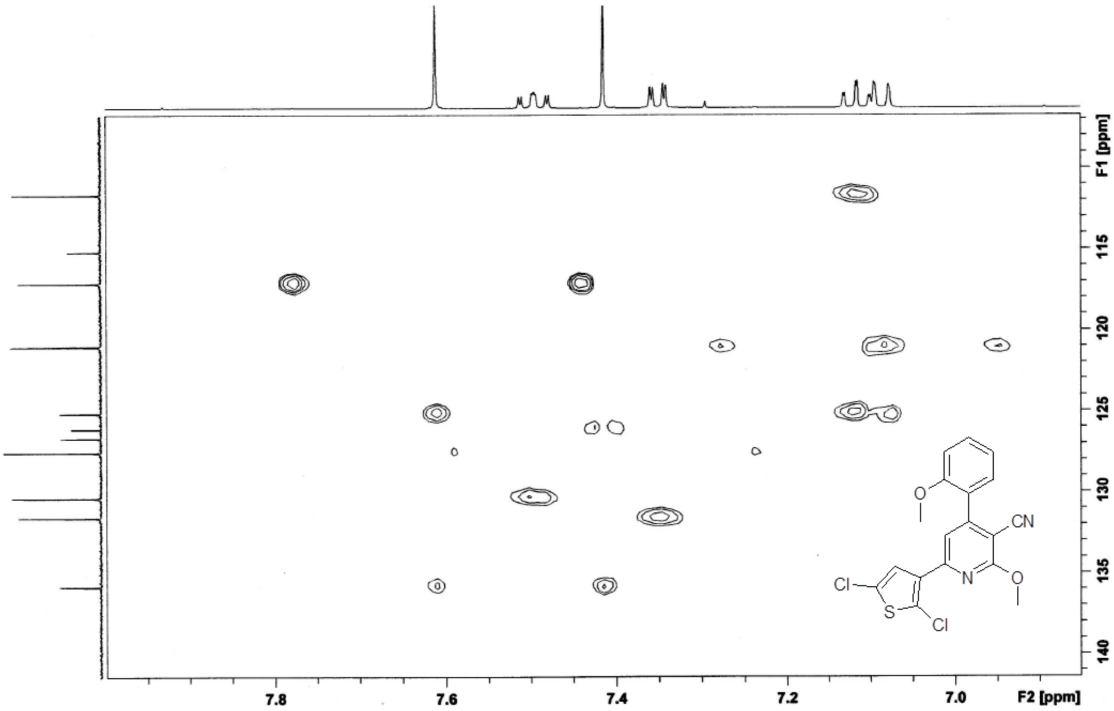

S 46: HMBC spectrum ($CDCl_3$, 500 MHz) of 6-(2,5-dichlorothiophen-3-yl)-2-methoxy-4-(2-methoxyphenyl)pyridine-3-carbonitrile (**5e**).

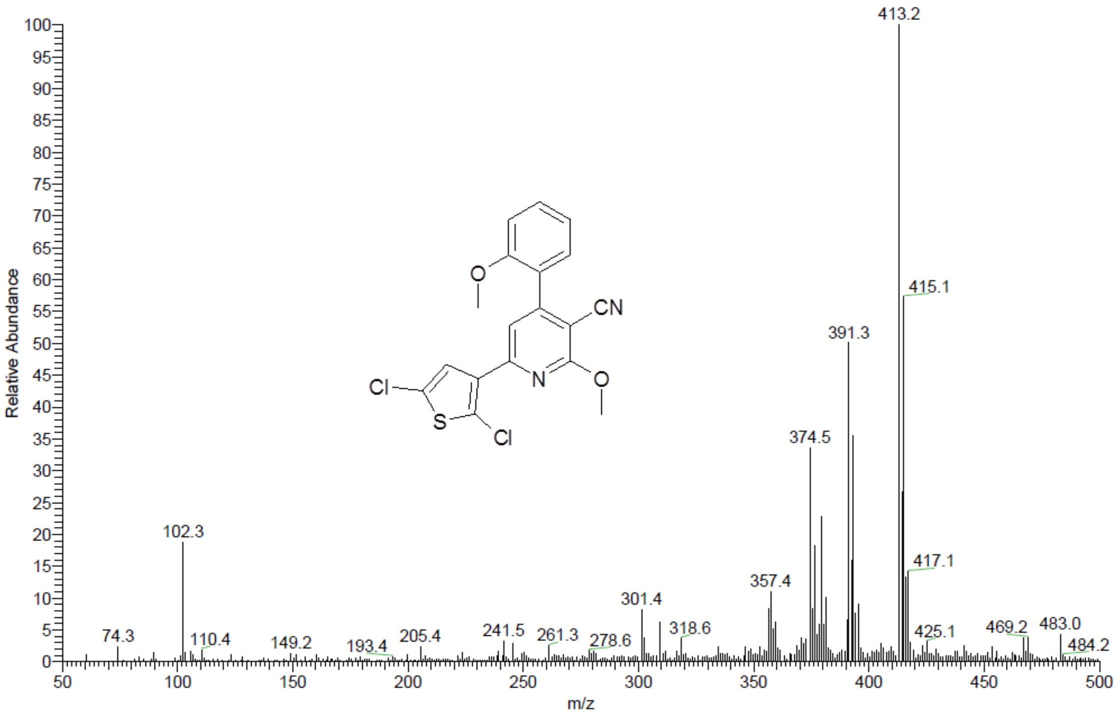

S 47: ESI mass spectrum of 6-(2,5-dichlorothiophen-3-yl)-2-methoxy-4-(2-methoxyphenyl)pyridine-3-carbonitrile (**5e**).

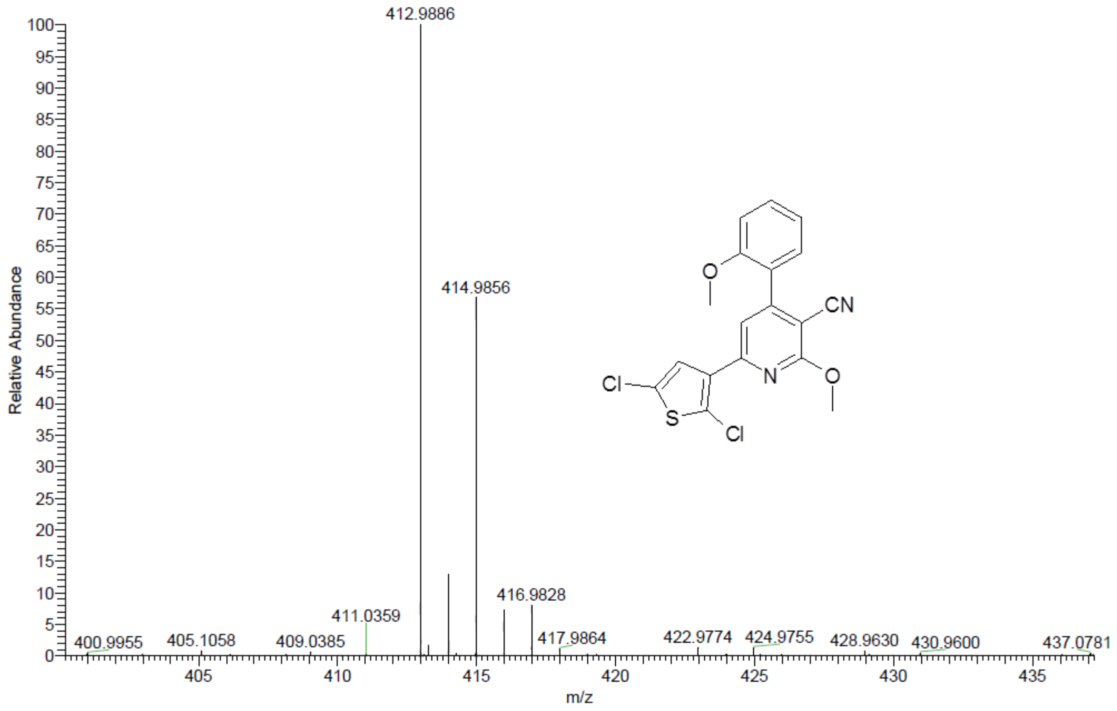

S 48: ESI mass spectrum of 6-(2,5-dichlorothiophen-3-yl)-2-methoxy-4-(2-methoxyphenyl)pyridine-3-carbonitrile (**5e**).

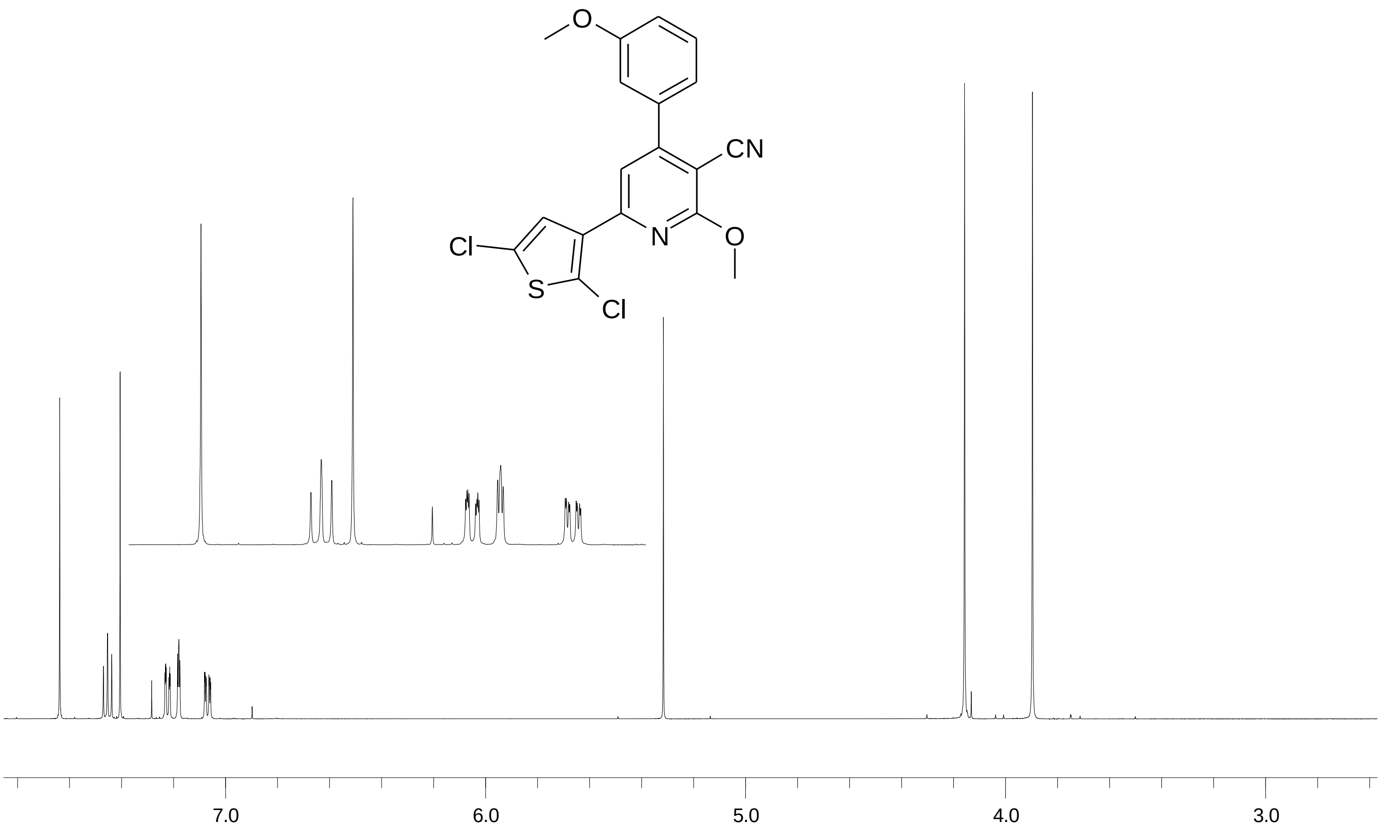

S 49: ^{1}H NMR spectrum ($CDCl_3$, 500 MHz) of 6-(2,5-dichlorothiophen-3-yl)-2-methoxy-4-(3-methoxyphenyl)pyridine-3-carbonitrile (**5f**).

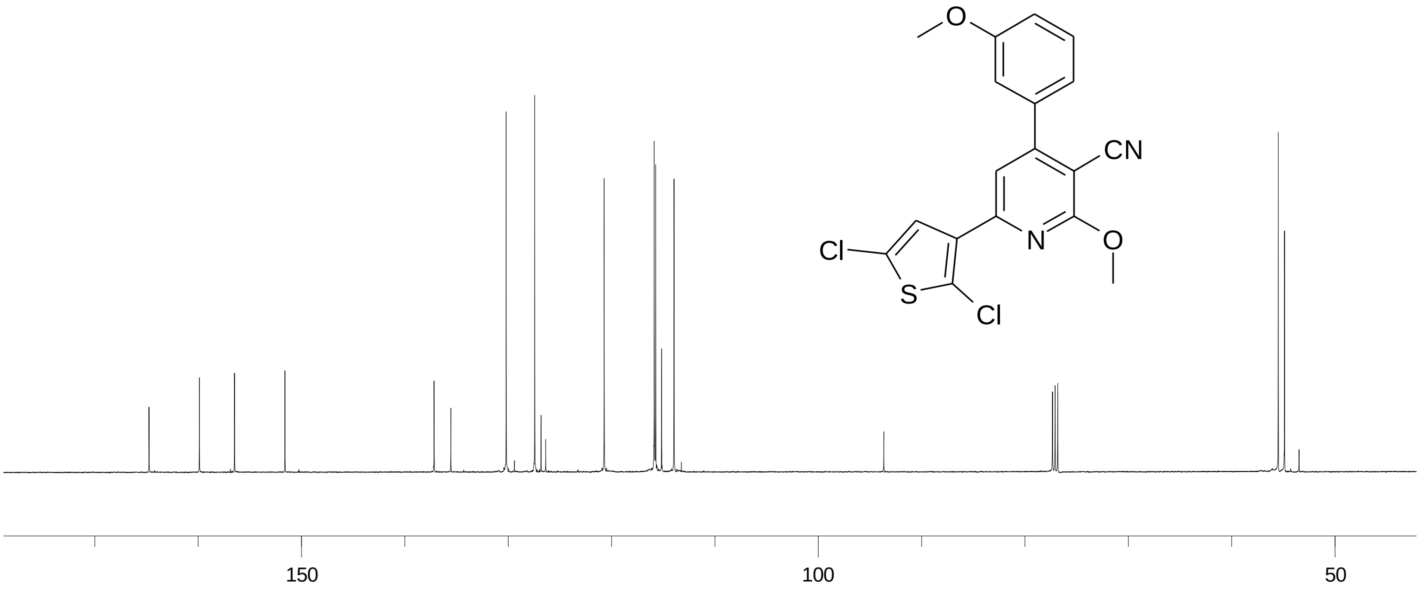

S 50: ^{13}C NMR spectrum ($CDCl_3$, 125MHz) of 6-(2,5-dichlorothiophen-3-yl)-2-methoxy-4-(3-methoxyphenyl)pyridine-3-carbonitrile (**5f**).

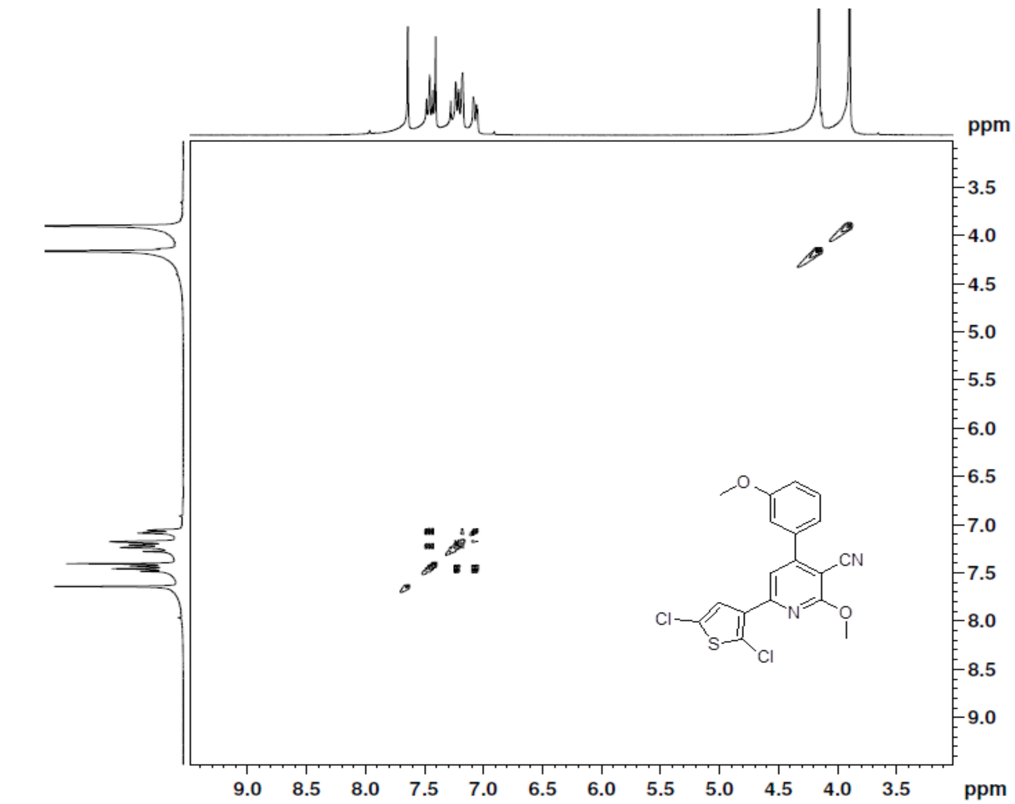

S 51: H,H COSY spectrum ($CDCl_3$, 300 MHz) of 6-(2,5-dichlorothiophen-3-yl)-2-methoxy-4-(3-methoxyphenyl)pyridine-3-carbonitrile (**5f**).

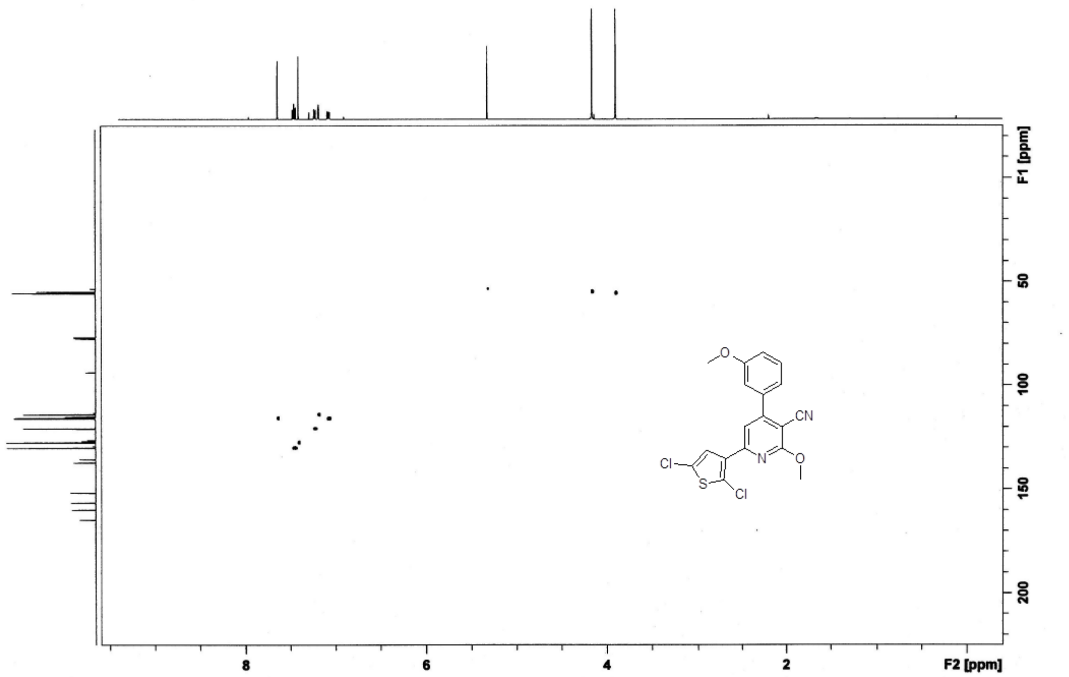

S 52: HSQC spectrum ($CDCl_3$, 500 MHz) of 6-(2,5-dichlorothiophen-3-yl)-2-methoxy-4-(3-methoxyphenyl)pyridine-3-carbonitrile (**5f**).

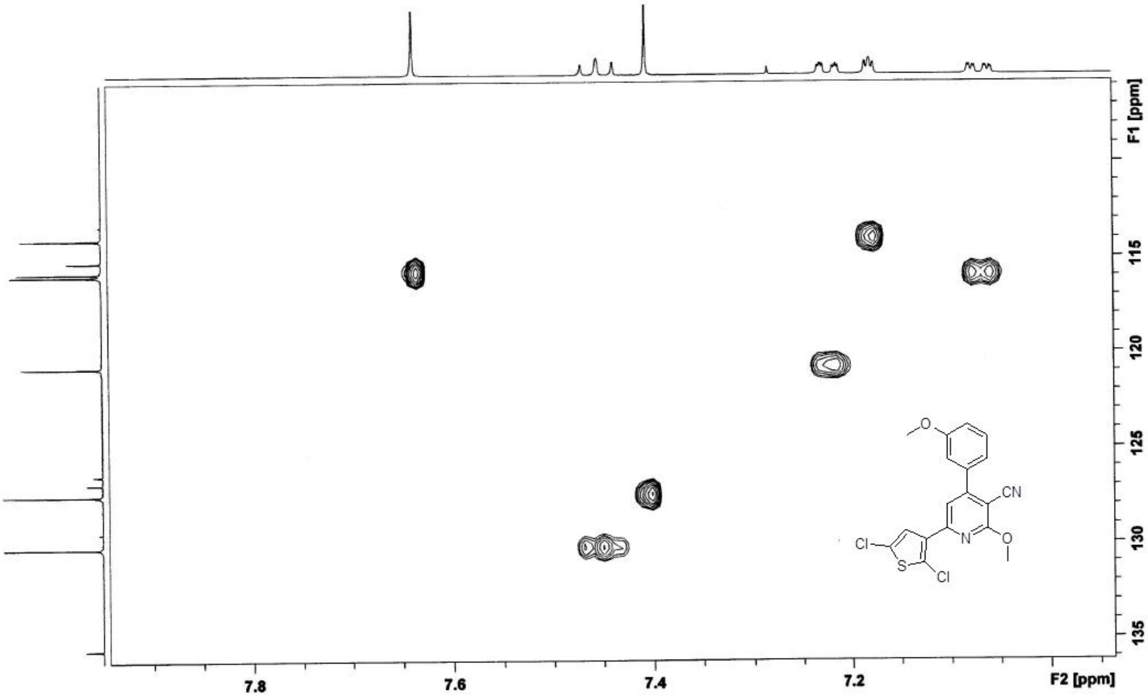

S 53: HSQC spectrum ($CDCl_3$, 500 MHz) of 6-(2,5-dichlorothiophen-3-yl)-2-methoxy-4-(3-methoxyphenyl)pyridine-3-carbonitrile (**5f**).

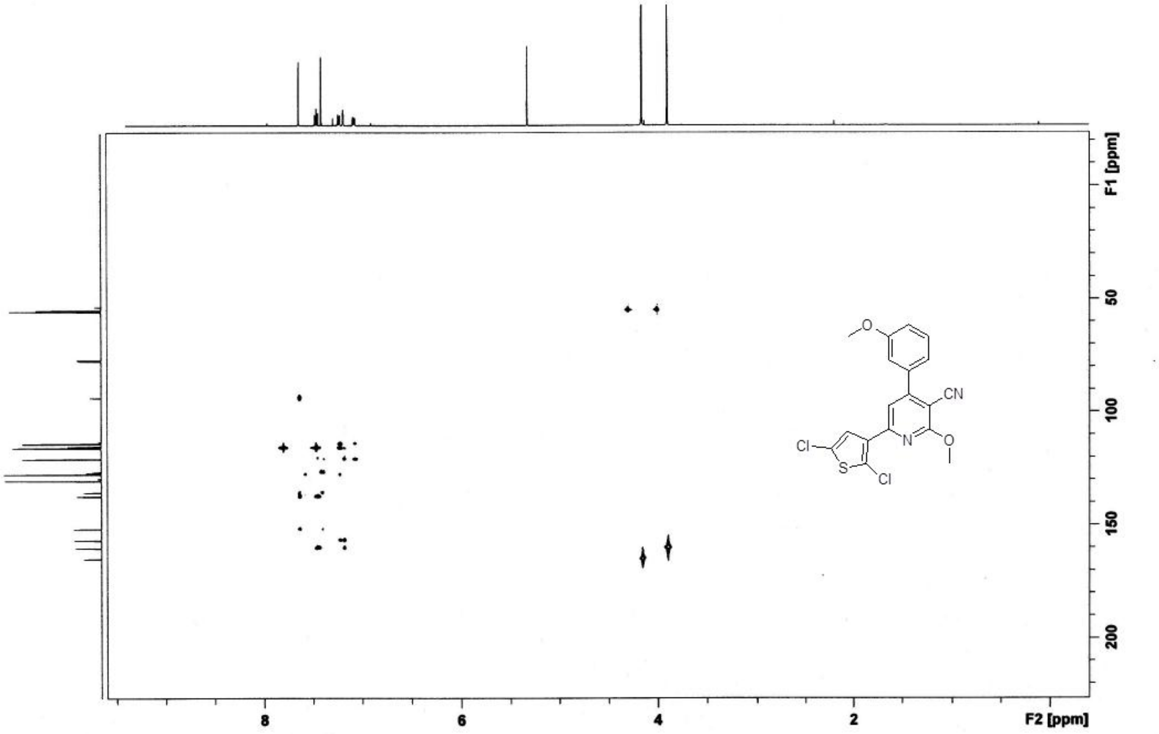

S 54: HMBC spectrum ($CDCl_3$, 500 MHz) of 6-(2,5-dichlorothiophen-3-yl)-2-methoxy-4-(3-methoxyphenyl)pyridine-3-carbonitrile (**5f**).

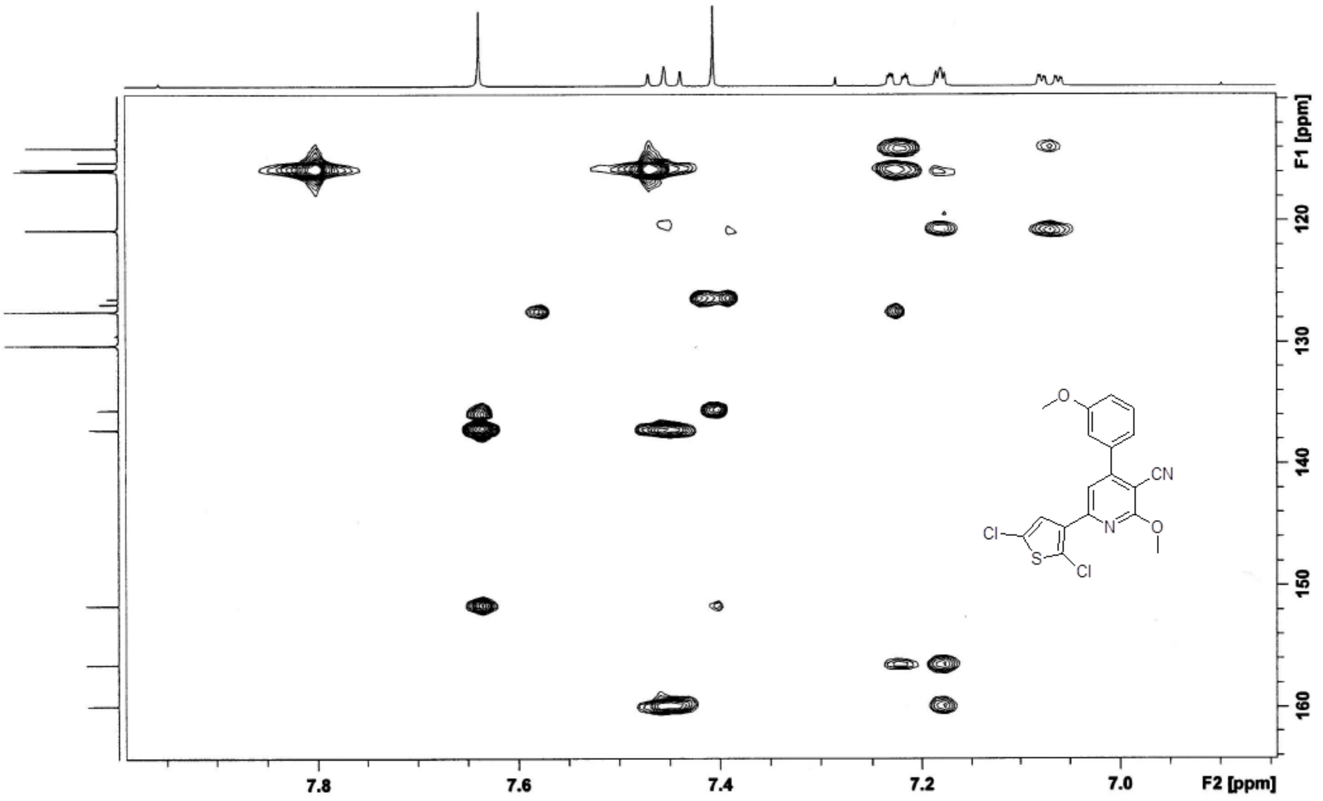

S 55: HMBC spectrum ($CDCl_3$, 500 MHz) of 6-(2,5-dichlorothiophen-3-yl)-2-methoxy-4-(3-methoxyphenyl)pyridine-3-carbonitrile (**5f**).

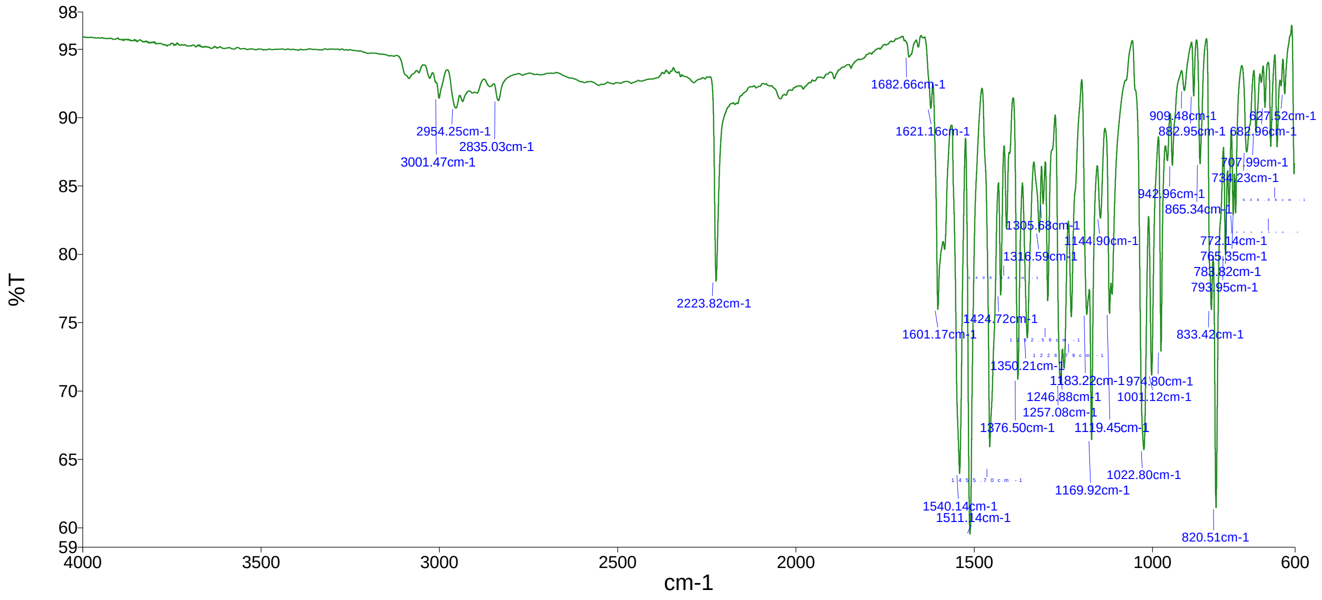

S 56: IR spectrum of 6-(2,5-dichlorothiophen-3-yl)-2-methoxy-4-(3-methoxyphenyl)pyridine-3-carbonitrile (**5f**).

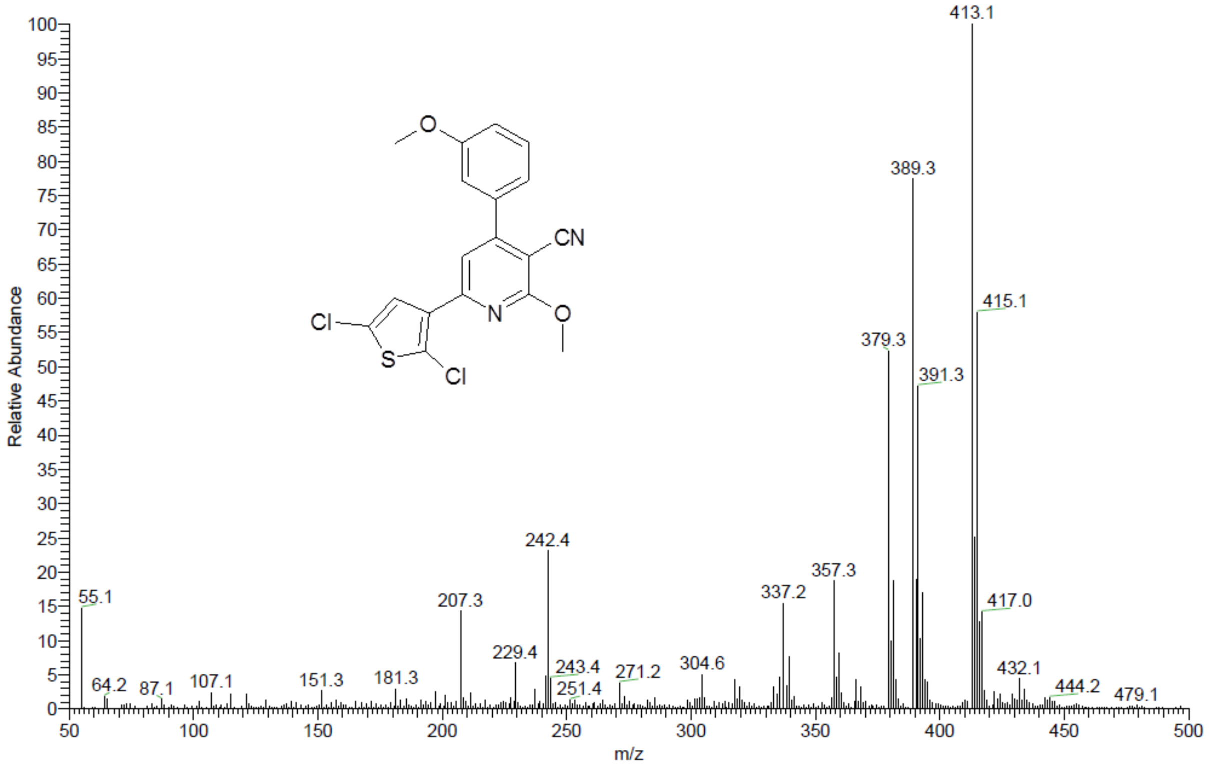

S 57: ESI mass spectrum of 6-(2,5-dichlorothiophen-3-yl)-2-methoxy-4-(3-methoxyphenyl)pyridine-3-carbonitrile (**5f**).

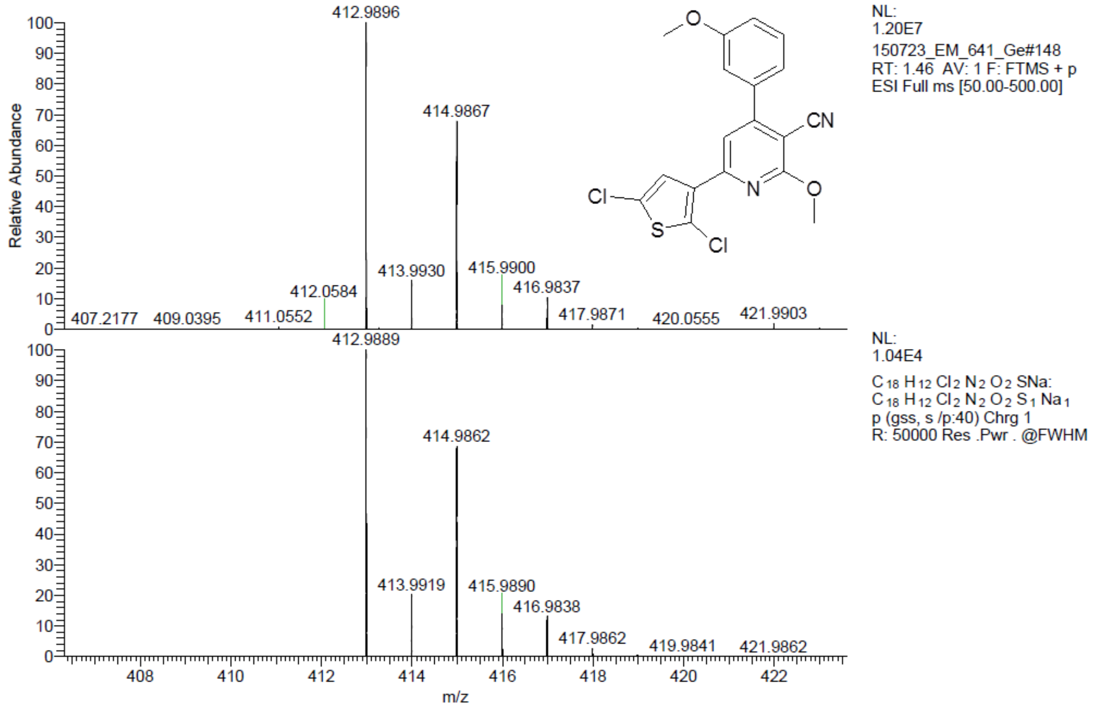

S 58: HRESI mass spectrum of 6-(2,5-dichlorothiophen-3-yl)-2-methoxy-4-(3-methoxyphenyl)pyridine-3-carbonitrile (**5f**).

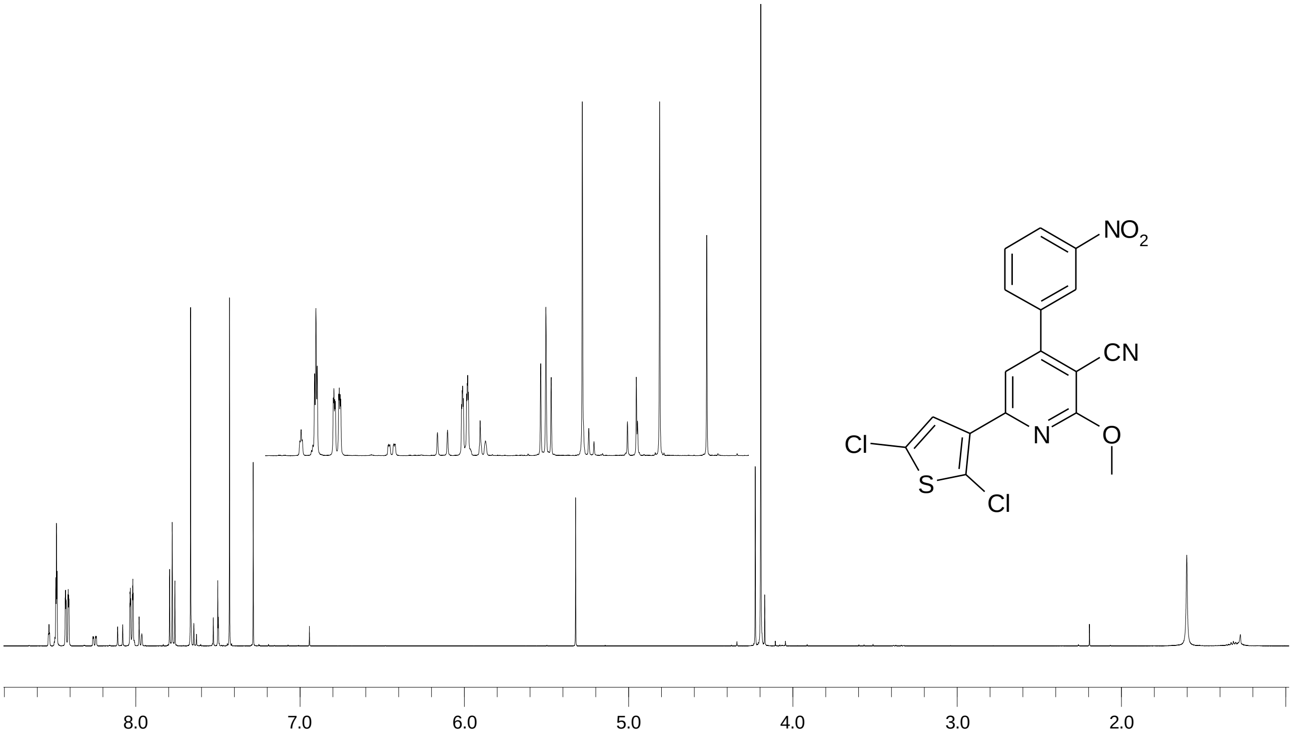

S 59: ^{1}H NMR spectrum ($CDCl_3$, 500 MHz) of 6-(2,5-dichlorothiophen-3-yl)-2-methoxy-4-(3-nitrophenyl)pyridine-3-carbonitrile (**5g**).

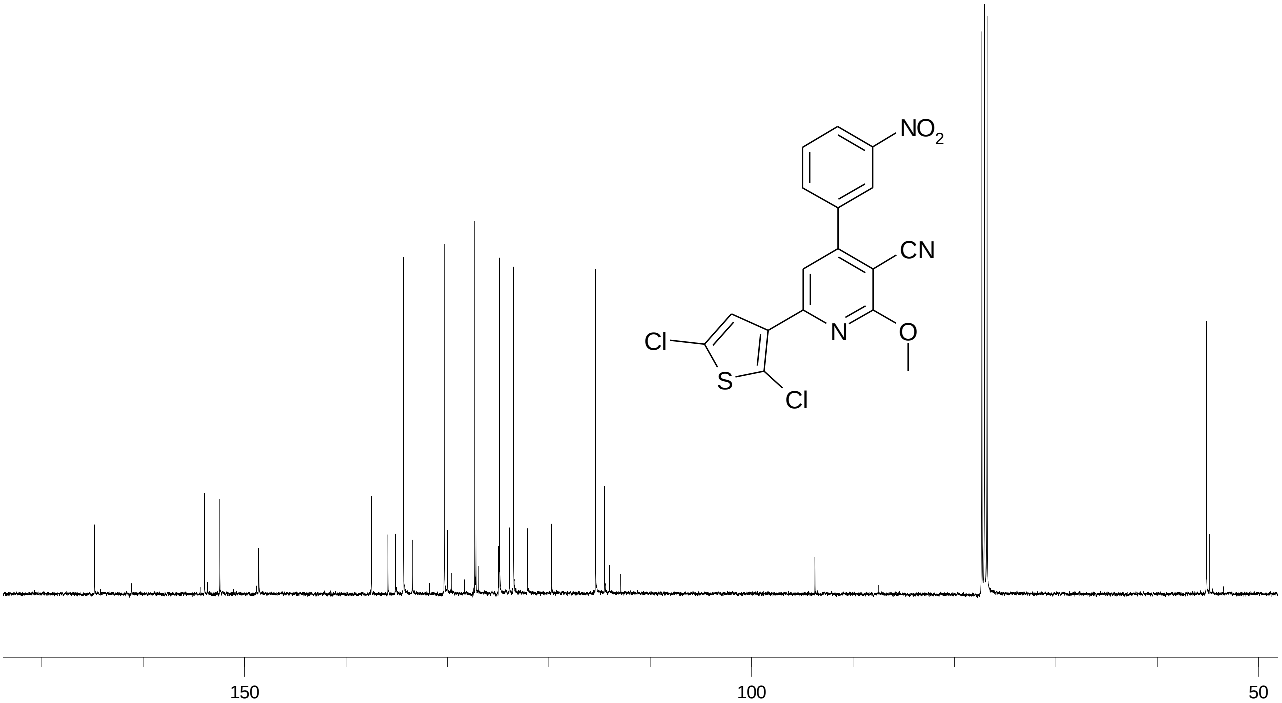

S 60: ^{13}C NMR spectrum ($CDCl_3$, 125MHz) of 6-(2,5-dichlorothiophen-3-yl)-2-methoxy-4-(3-nitrophenyl)pyridine-3-carbonitrile (**5g**).

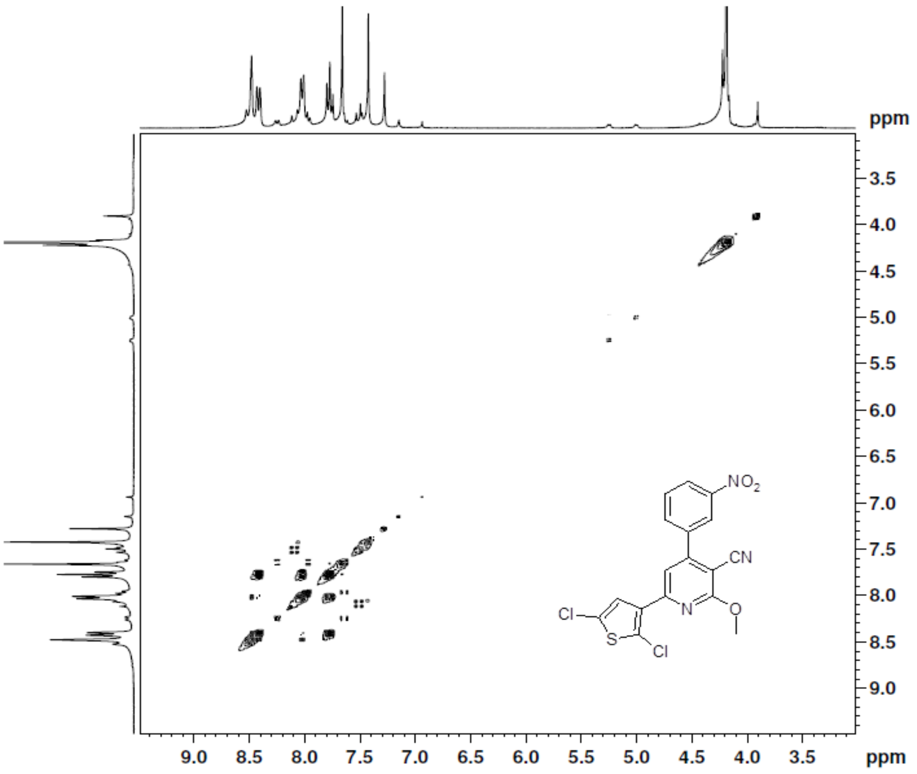

S 61: H,H COSY spectrum ($CDCl_3$, 300 MHz) of 6-(2,5-dichlorothiophen-3-yl)-2-methoxy-4-(3-methoxyphenyl)pyridine-3-carbonitrile (**5g**).

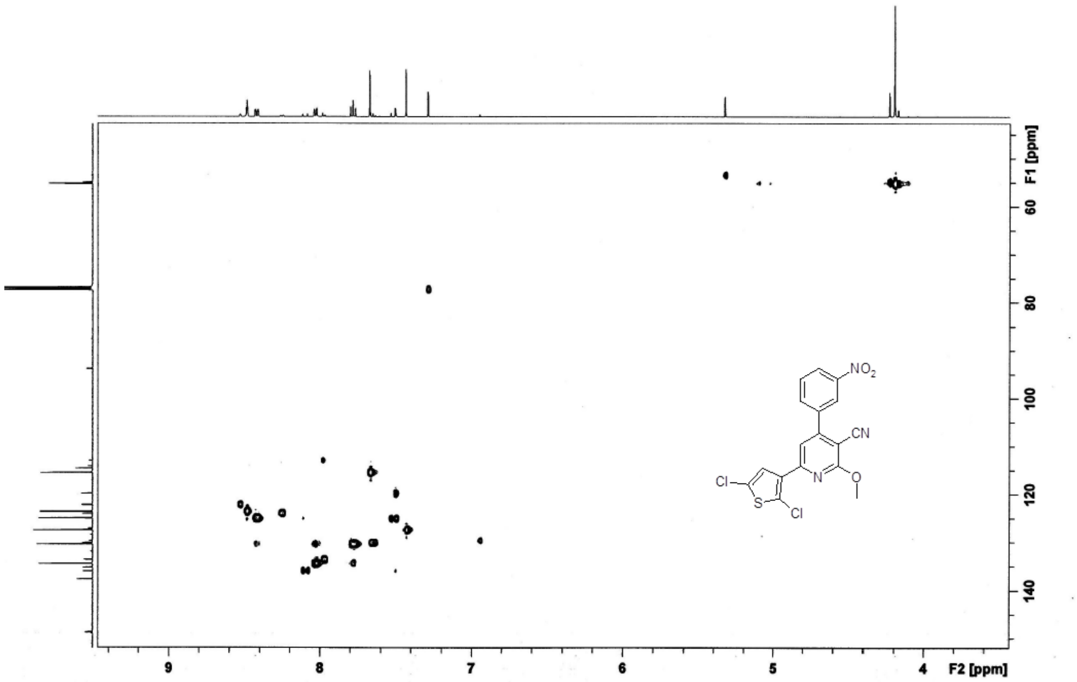

S 62: HSQC: spectrum ($CDCl_3$, 500 MHz) of 6-(2,5-dichlorothiophen-3-yl)-2-methoxy-4-(3-nitrophenyl)pyridine-3-carbonitrile (**5g**).

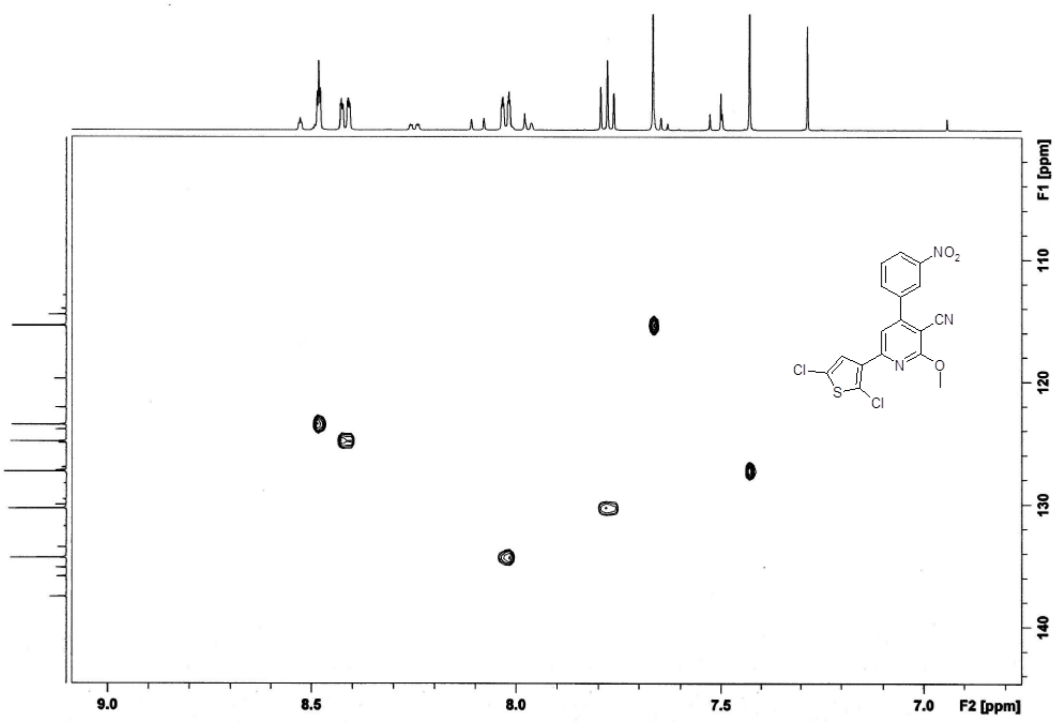

S 63: HSQC spectrum ($CDCl_3$, 500 MHz) of 6-(2,5-dichlorothiophen-3-yl)-2-methoxy-4-(3-nitrophenyl)pyridine-3-carbonitrile (**5g**).

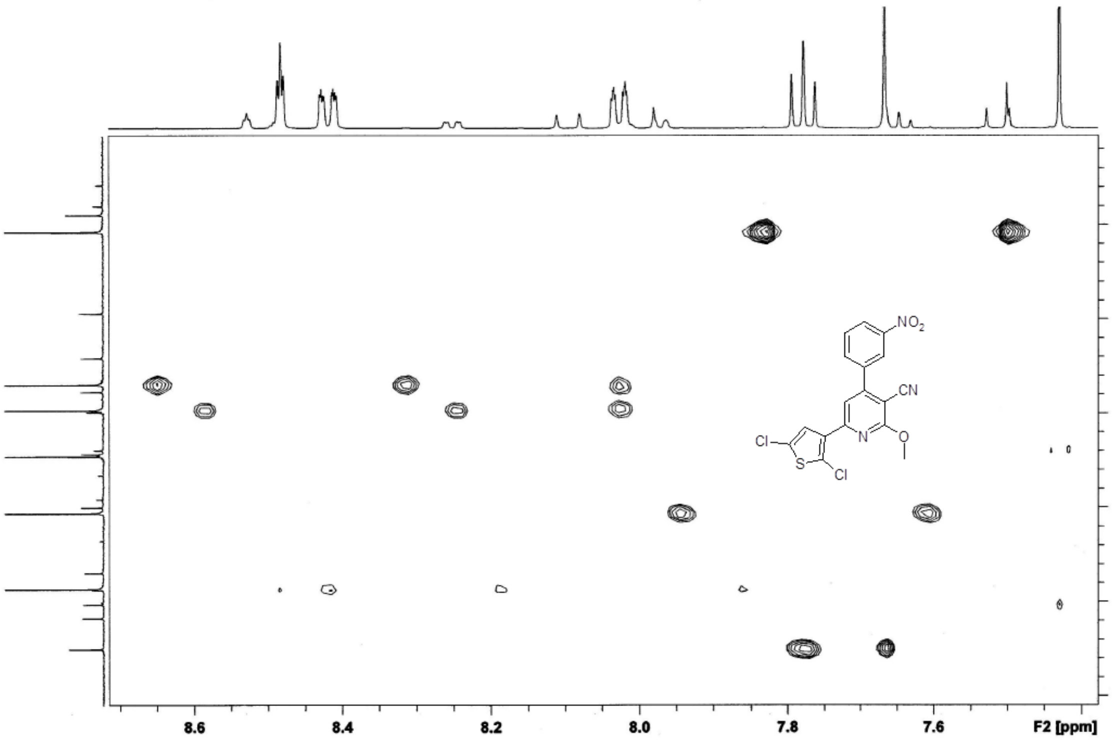

S 64: HMBC spectrum ($CDCl_3$, 500 MHz) of 6-(2,5-dichlorothiophen-3-yl)-2-methoxy-4-(3-nitrophenyl)pyridine-3-carbonitrile (**5g**).

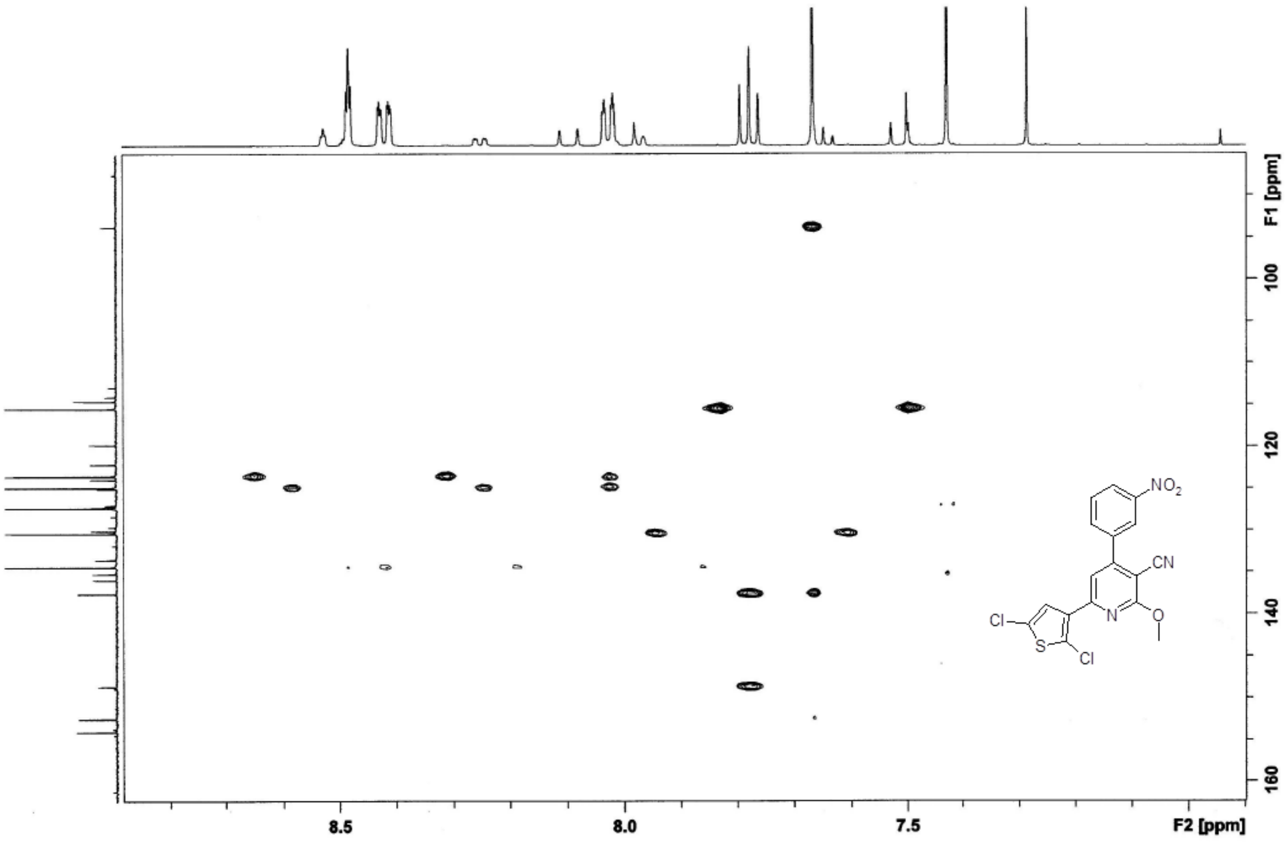

S 65: HMBC spectrum ($CDCl_3$, 500 MHz) of 6-(2,5-dichlorothiophen-3-yl)-2-methoxy-4-(3-nitrophenyl)pyridine-3-carbonitrile (**5g**).

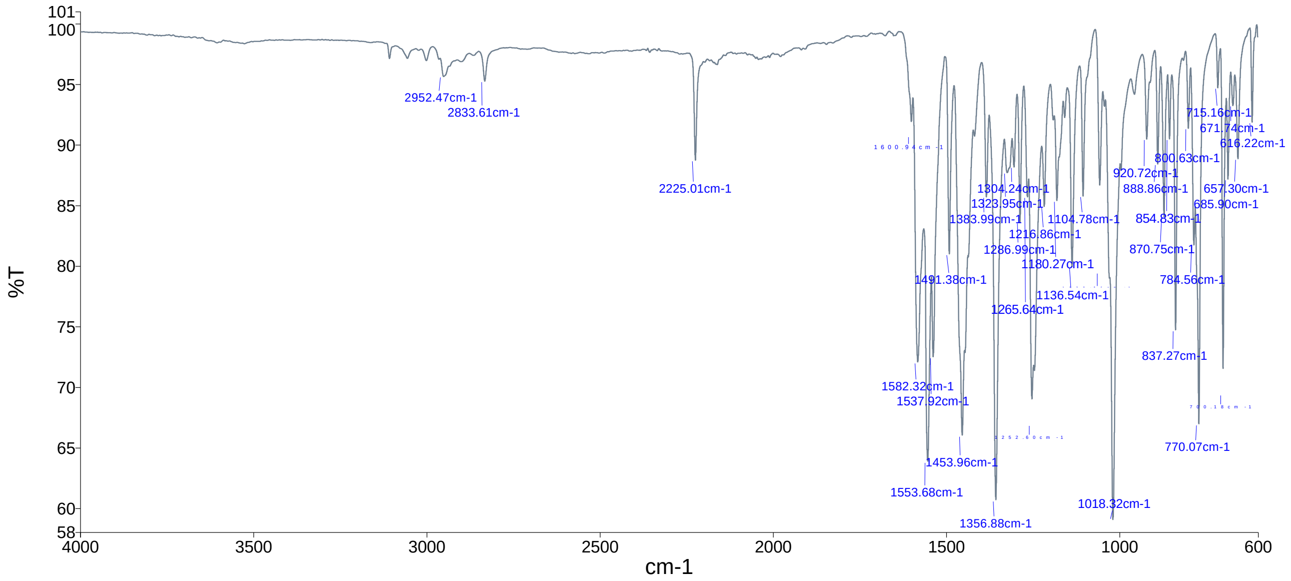

S 66: IR spectrum of 6-(2,5-dichlorothiophen-3-yl)-2-methoxy-4-(3-nitrophenyl)pyridine-3-carbonitrile (**5g**).

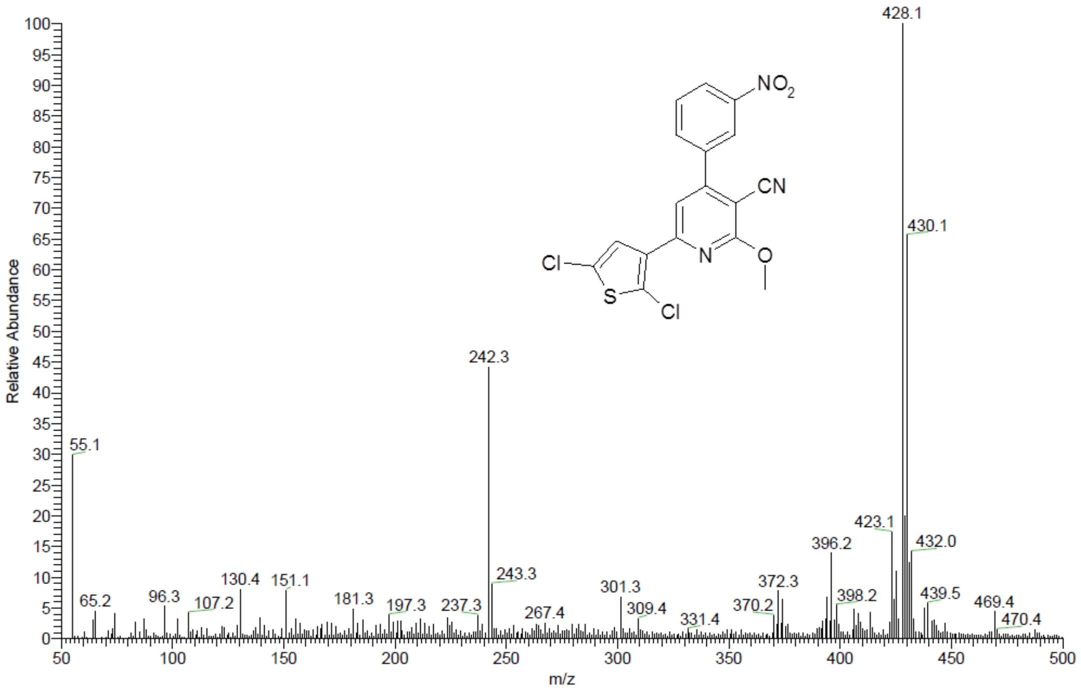

S 67: ESI mass spectrum of 6-(2,5-dichlorothiophen-3-yl)-2-methoxy-4-(3-nitrophenyl)pyridine-3-carbonitrile (**5g**).

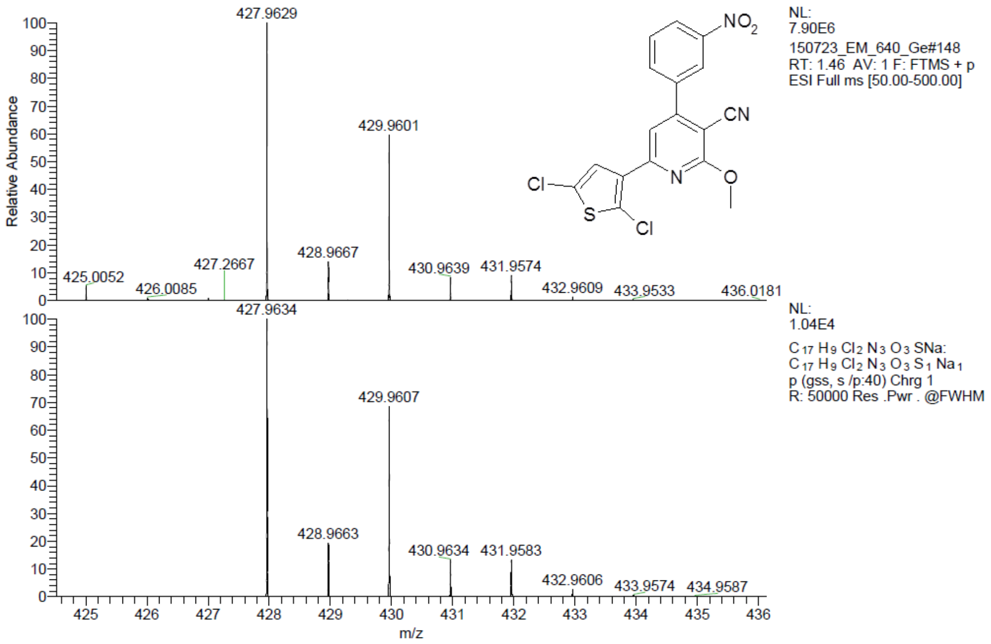

S 68: HRESI mass spectrum of 6-(2,5-dichlorothiophen-3-yl)-2-methoxy-4-(3-nitrophenyl)pyridine-3-carbonitrile (**5g**).

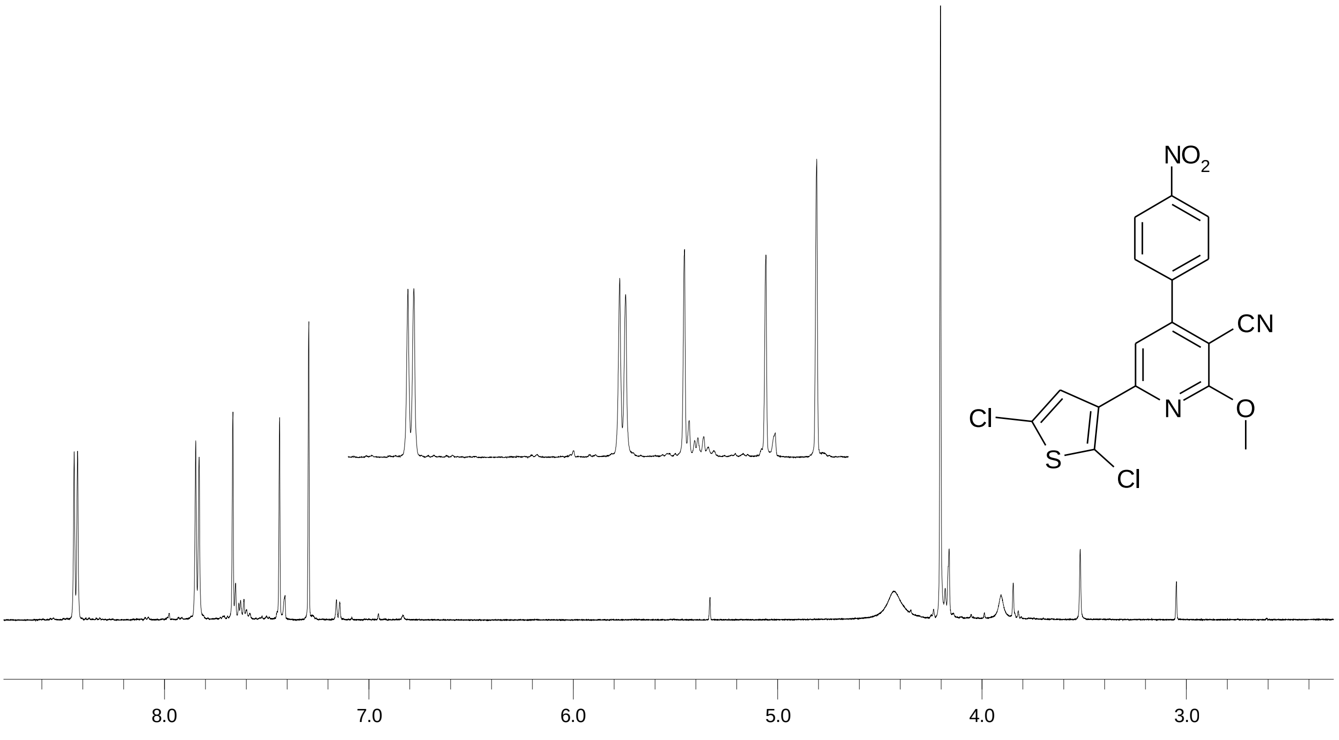

S 69: ^{1}H NMR spectrum ($CDCl_3$, 500 MHz) of 6-(2,5-dichlorothiophen-3-yl)-2-methoxy-4-(4-nitrophenyl)pyridine-3-carbonitrile (**5h**).

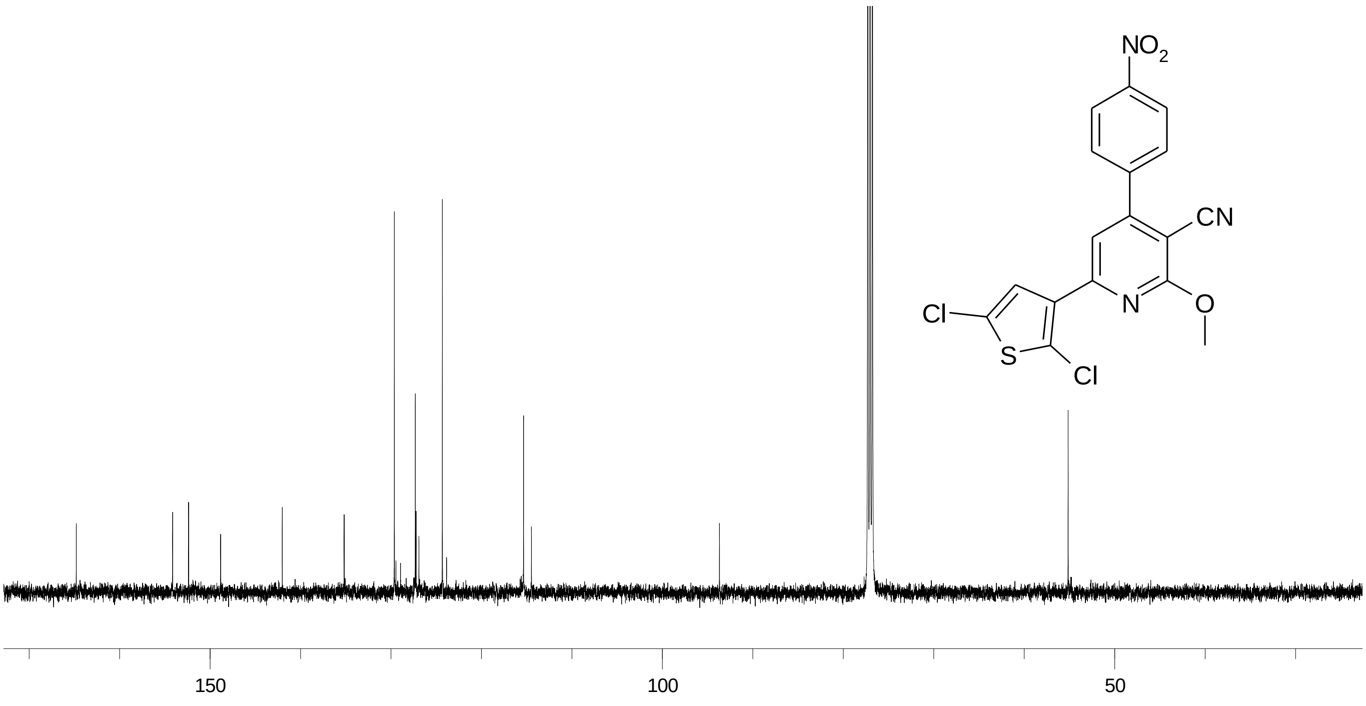

S 70: ^{13}C NMR spectrum ($CDCl_3$, 125MHz) of 6-(2,5-dichlorothiophen-3-yl)-2-methoxy-4-(4-nitrophenyl)pyridine-3-carbonitrile (**5h**).

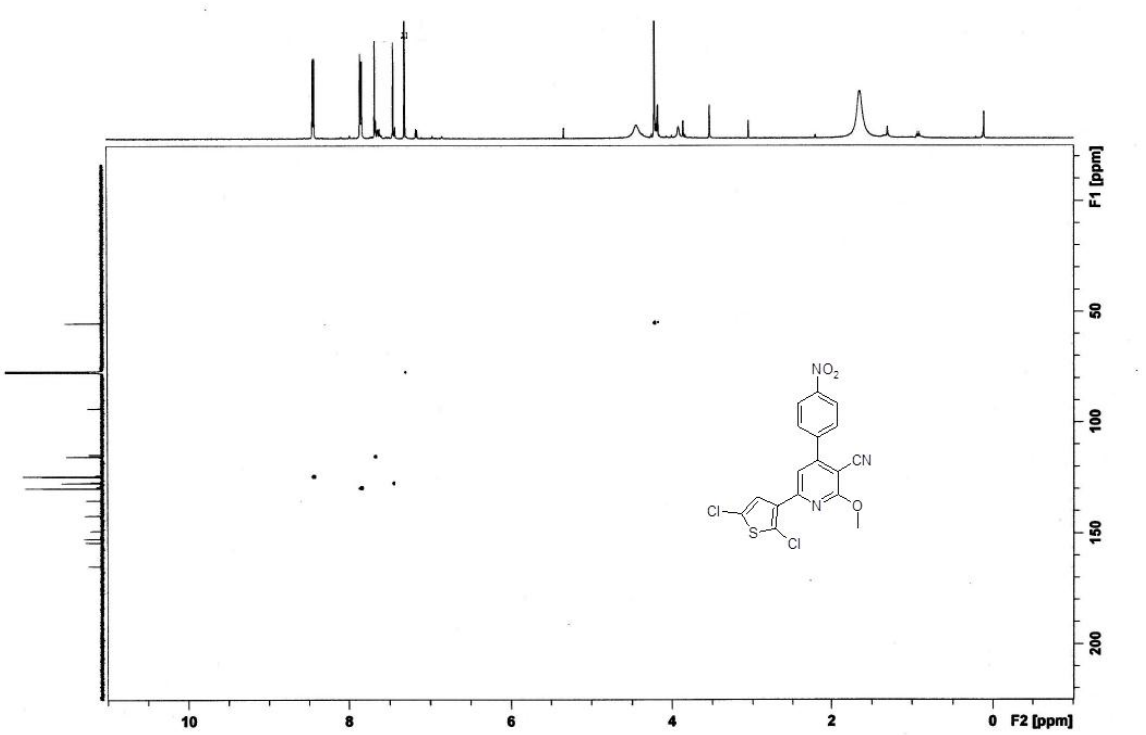

S 71: HSQC spectrum ($CDCl_3$, 500 MHz) of 6-(2,5-dichlorothiophen-3-yl)-2-methoxy-4-(4-nitrophenyl)pyridine-3-carbonitrile (**5h**).

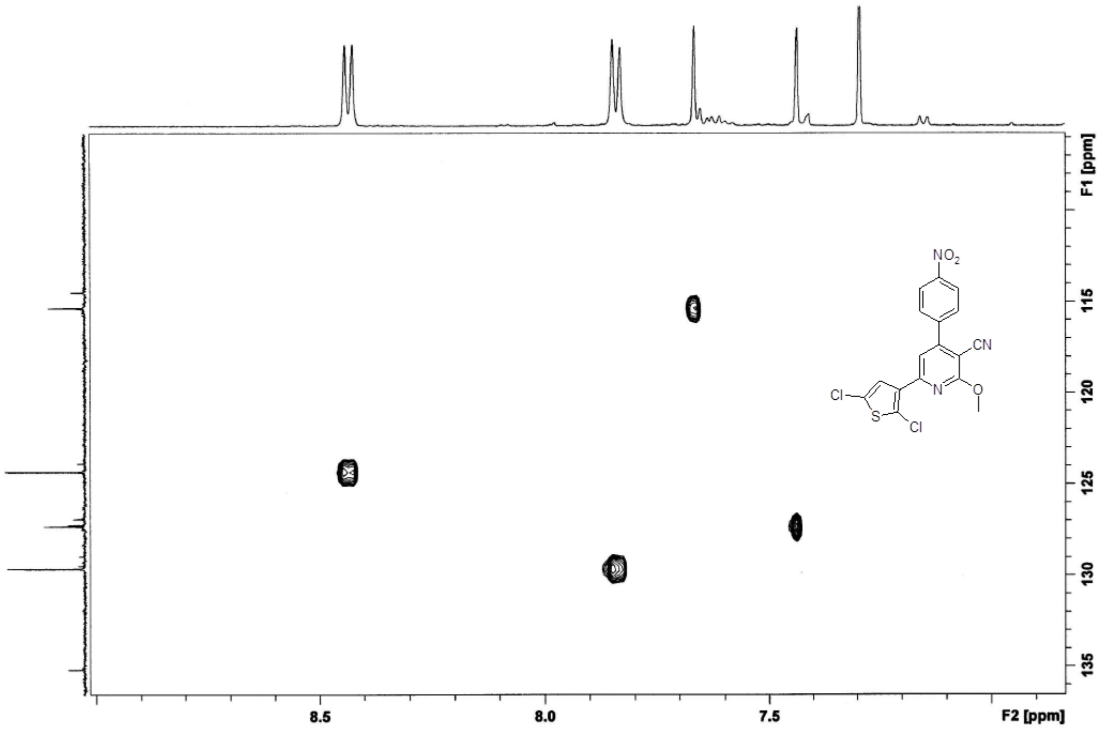

S 72: HSQC spectrum ($CDCl_3$, 500 MHz) of 6-(2,5-dichlorothiophen-3-yl)-2-methoxy-4-(4-nitrophenyl)pyridine-3-carbonitrile (**5h**).

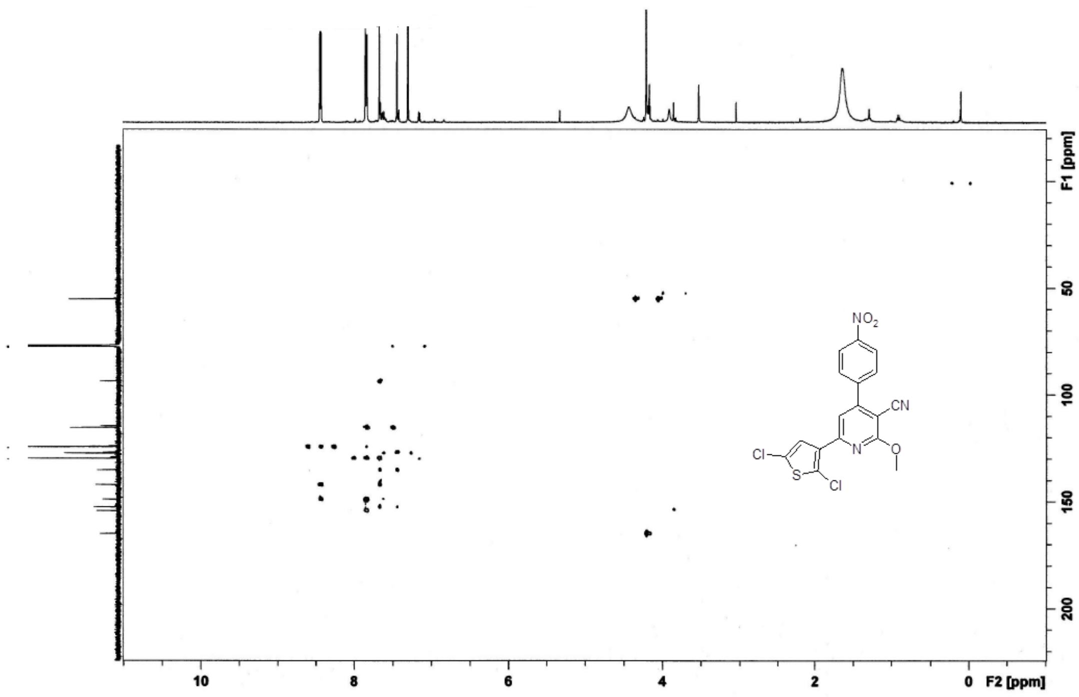

S 73: HMBC spectrum ($CDCl_3$, 500 MHz) of 6-(2,5-dichlorothiophen-3-yl)-2-methoxy-4-(4-nitrophenyl)pyridine-3-carbonitrile (**5h**).

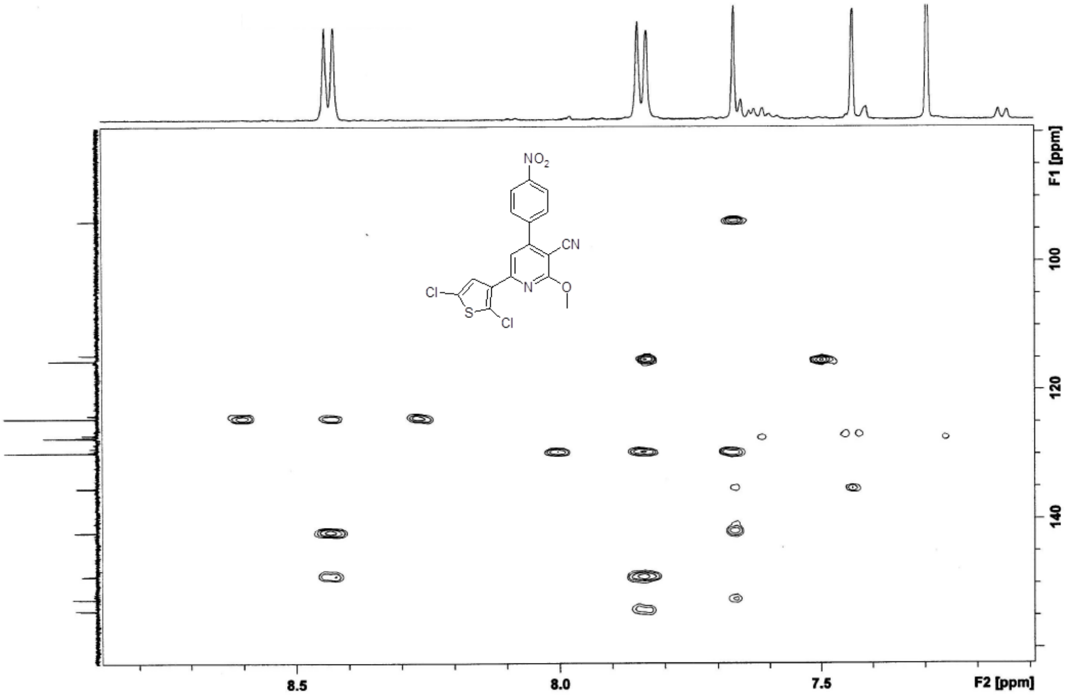

S 74: HMBC spectrum ($CDCl_3$, 500 MHz) of 6-(2,5-dichlorothiophen-3-yl)-2-methoxy-4-(4-nitrophenyl)pyridine-3-carbonitrile (**5h**).

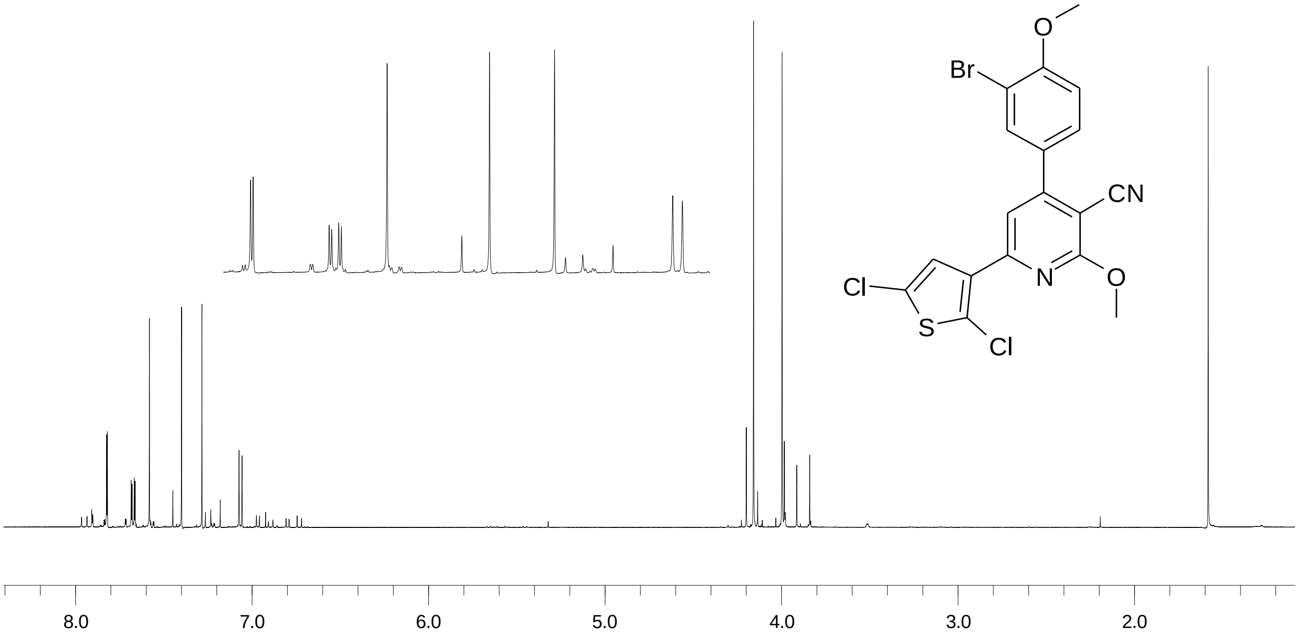

S 75: ^{1}H NMR spectrum ($CDCl_3$, 500 MHz) of 4-(3-bromo-4-methoxyphenyl)-6-(2,5-dichlorothiophen-3-yl)-2-methoxypyridine-3-carbonitrile (**5i**).

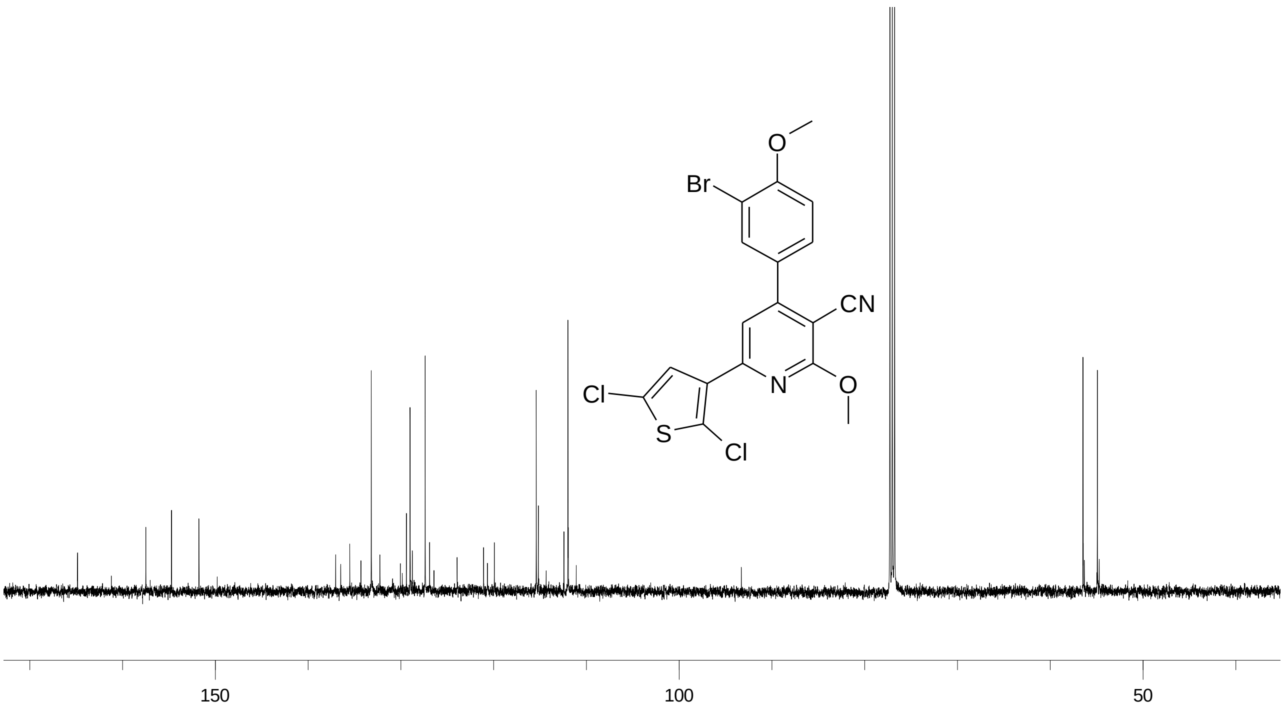

S 76: ^{13}C NMR spectrum ($CDCl_3$, 125MHz) of 4-(3-bromo-4-methoxyphenyl)-6-(2,5-dichlorothiophen-3-yl)-2-methoxypyridine-3-carbonitrile (**5i**).

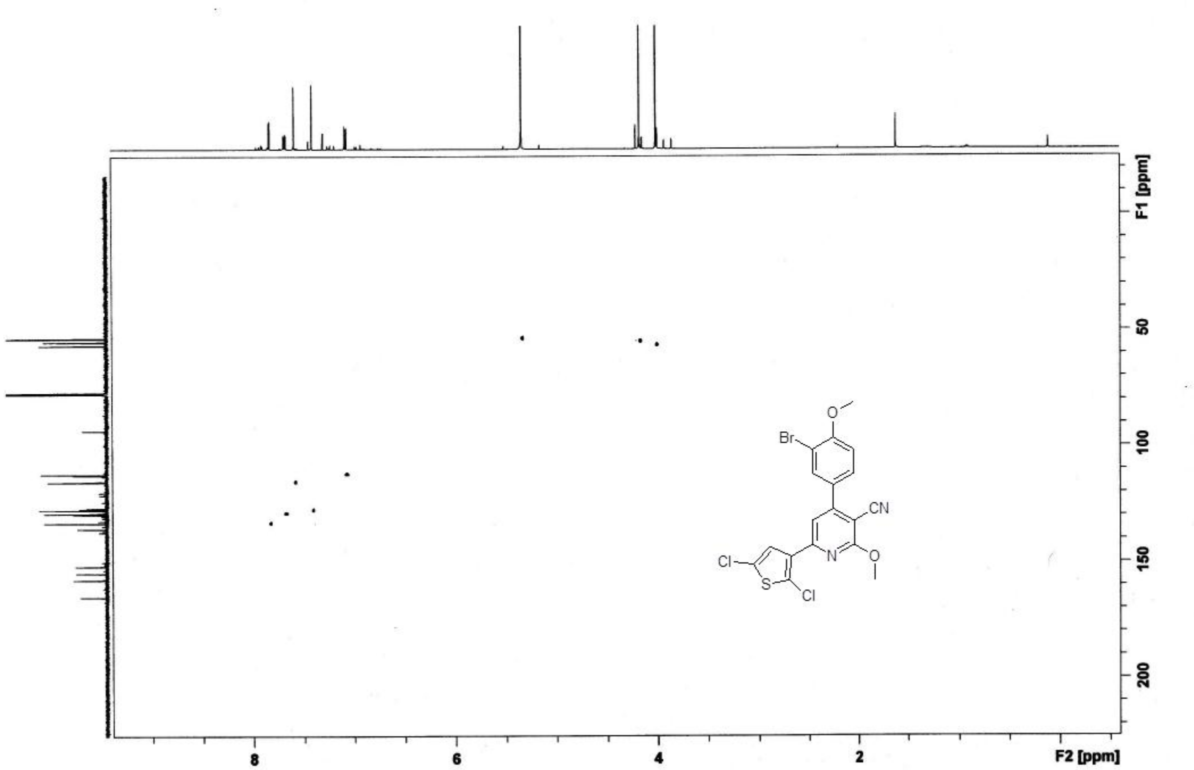

S 77: HSQC spectrum (CDCl3, 500 MHz) of 4-(3-bromo-4-methoxyphenyl)-6-(2,5-dichlorothiophen-3-yl)-2-methoxypyridine-3-carbonitrile (**5i**).

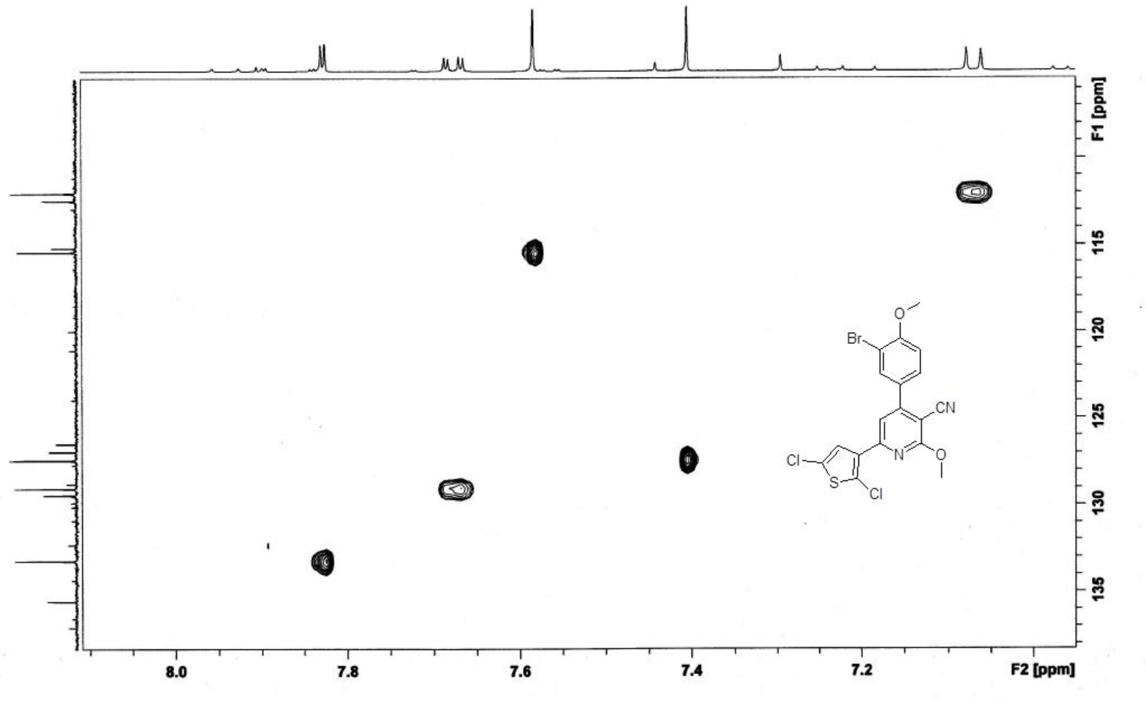

S 78: HSQC spectrum (CDCl3, 500 MHz) of 4-(3-bromo-4-methoxyphenyl)-6-(2,5-dichlorothiophen-3-yl)-2-methoxypyridine-3-carbonitrile (**5i**).

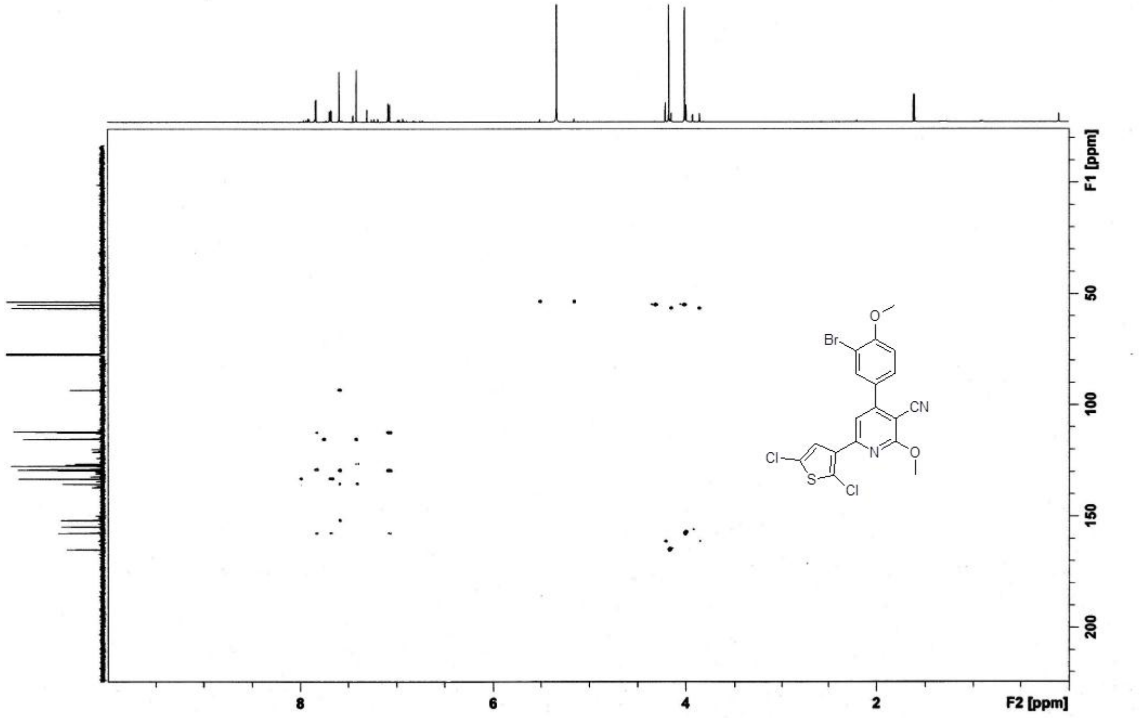

S 79: HMBC spectrum ($CDCl_3$, 500 MHz) of 4-(3-bromo-4-methoxyphenyl)-6-(2,5-dichlorothiophen-3-yl)-2-methoxypyridine-3-carbonitrile (**5i**).

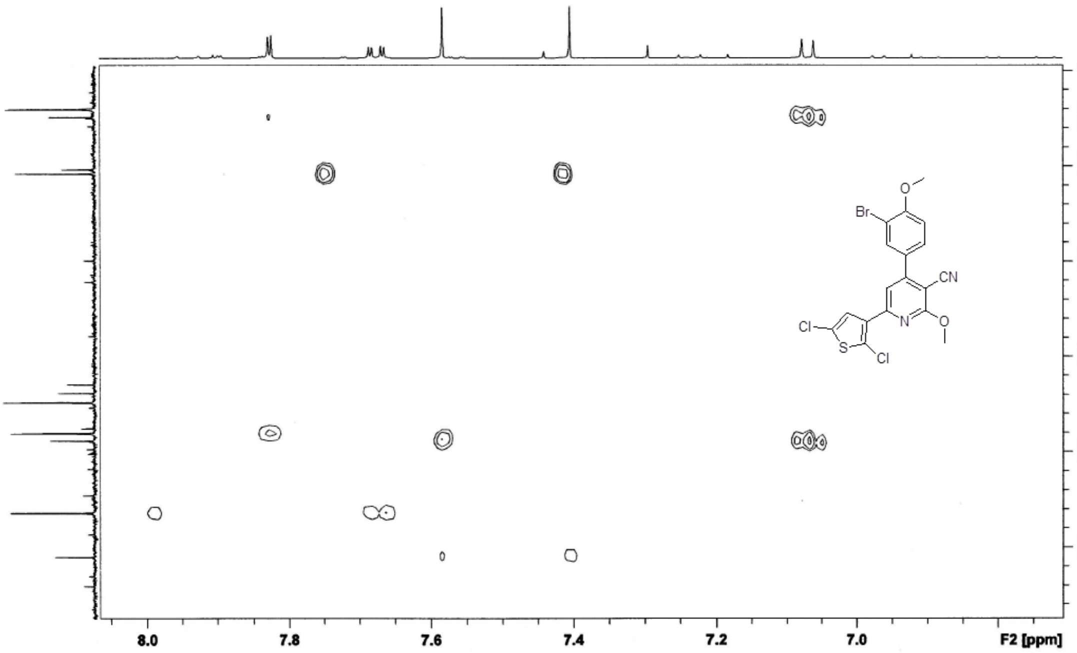

S 80: HMBC spectrum ($CDCl_3$, 500 MHz) of 4-(3-bromo-4-methoxyphenyl)-6-(2,5-dichlorothiophen-3-yl)-2-methoxypyridine-3-carbonitrile (**5i**).

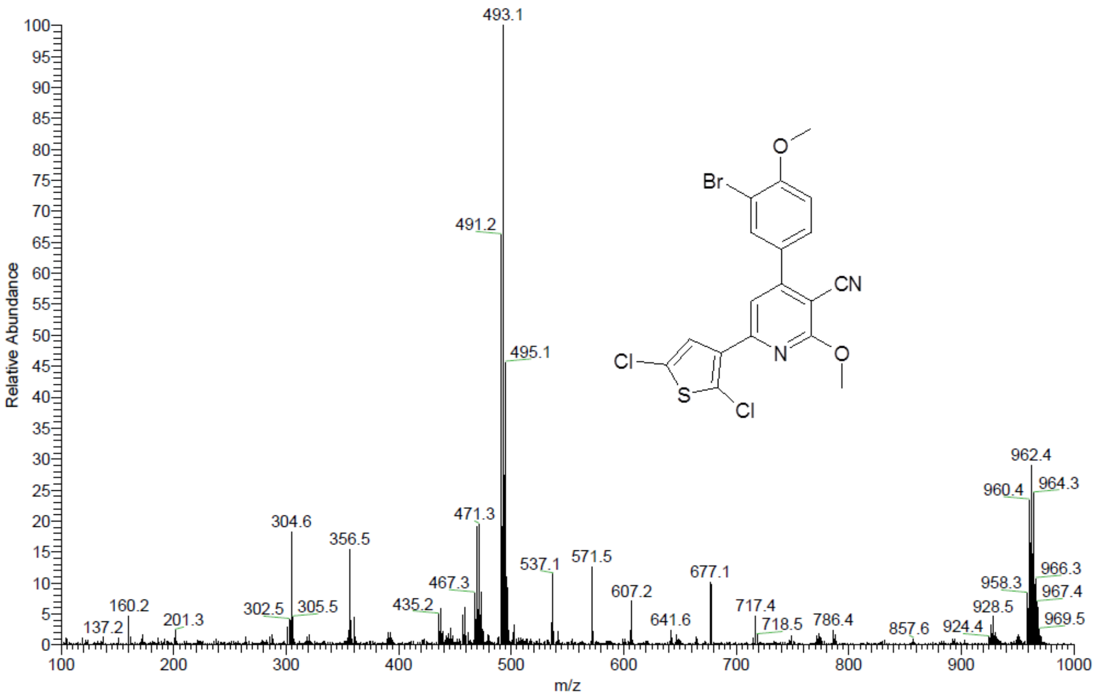

S 81: ESI mass spectrum of 4-(3-bromo-4-methoxyphenyl)-6-(2,5-dichlorothiophen-3-yl)-2-methoxypyridine-3-carbonitrile (**5i**).

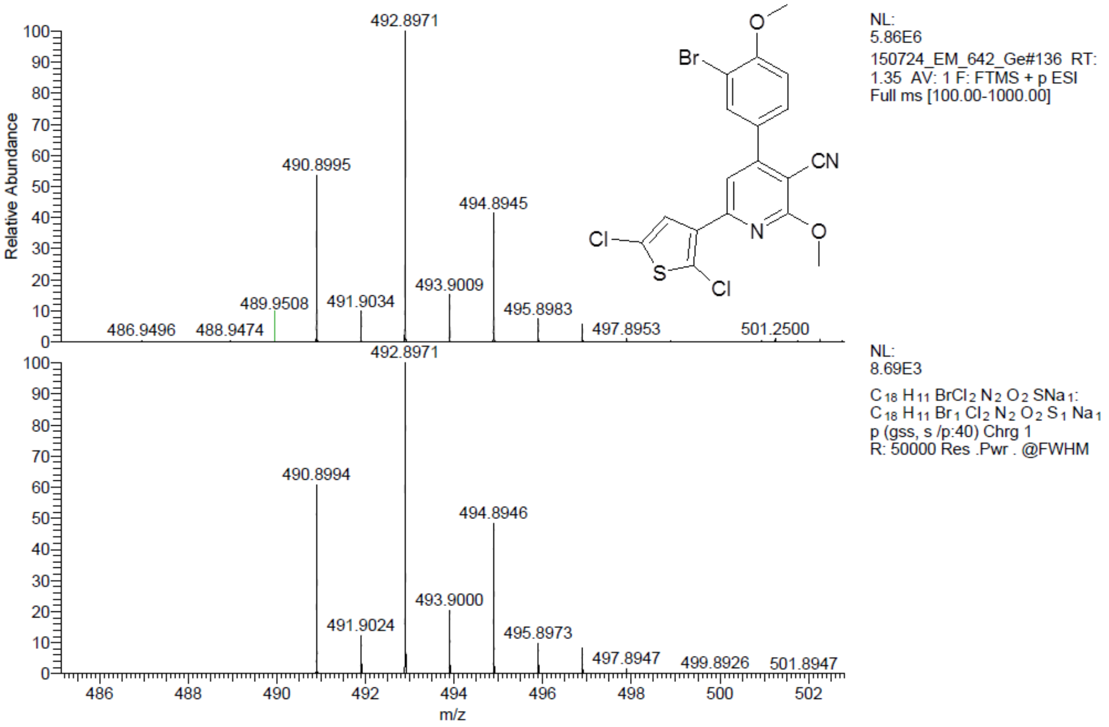

S 82: HRESI mass spectrum of 4-(3-bromo-4-methoxyphenyl)-6-(2,5-dichlorothiophen-3-yl)-2-methoxypyridine-3-carbonitrile (**5i**).

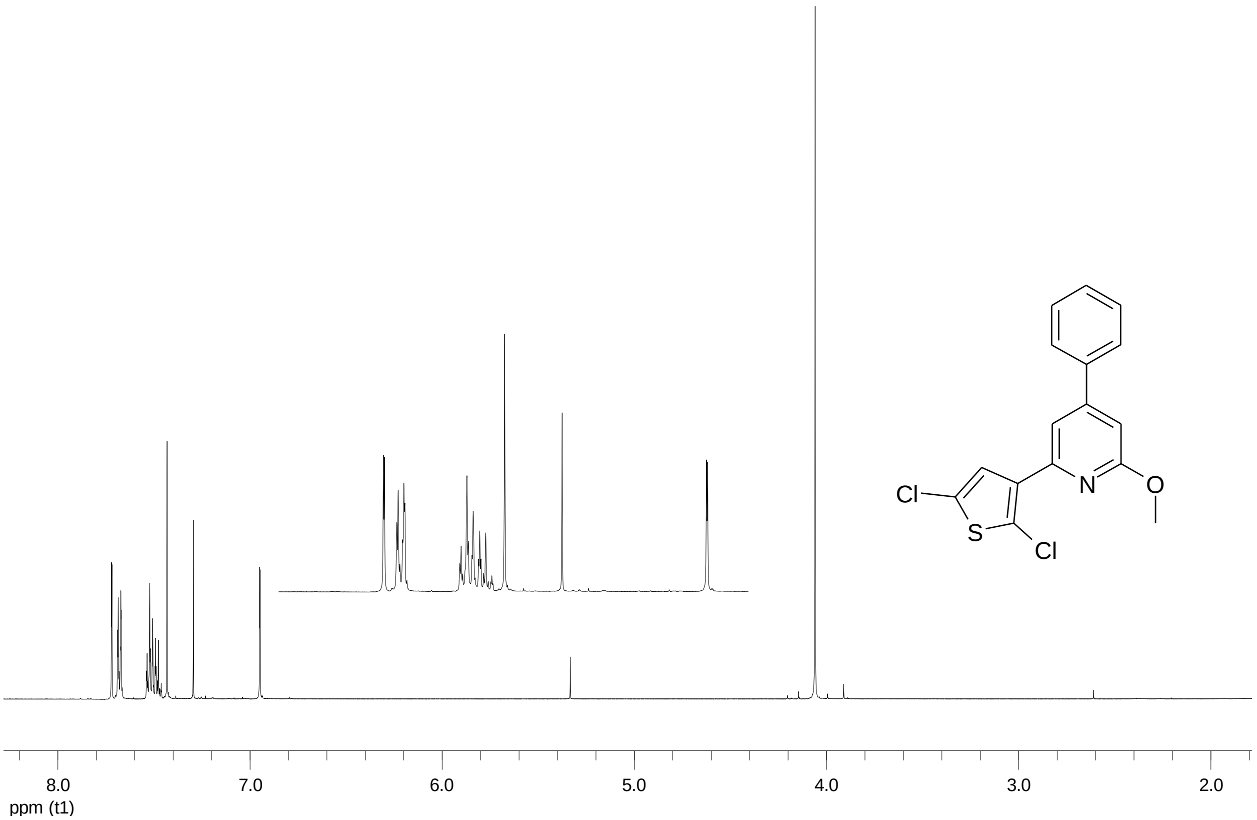

S 83: ^{1}H NMR spectrum ($CDCl_3$, 500 MHz) of 2-(2,5-dichlorothiophen-3-yl)-6-methoxy-4-phenylpyridine (**6a**).

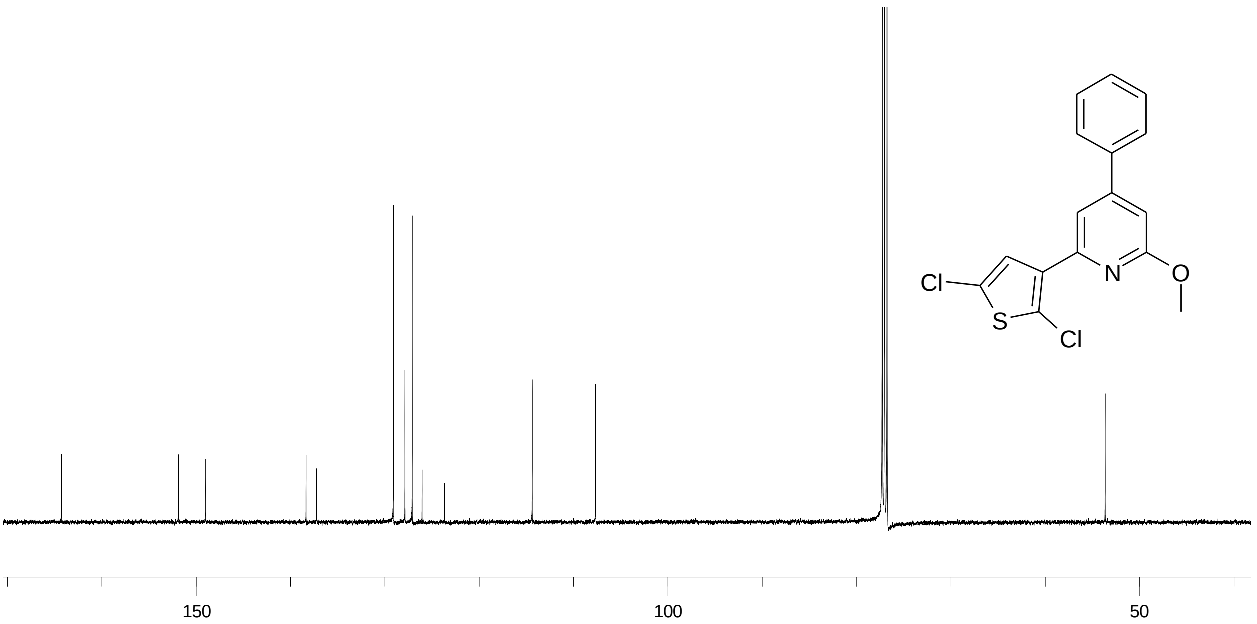

S 84: ^{13}C NMR spectrum ($CDCl_3$, 125MHz) of 2-(2,5-dichlorothiophen-3-yl)-6-methoxy-4-phenylpyridine (**6a**).

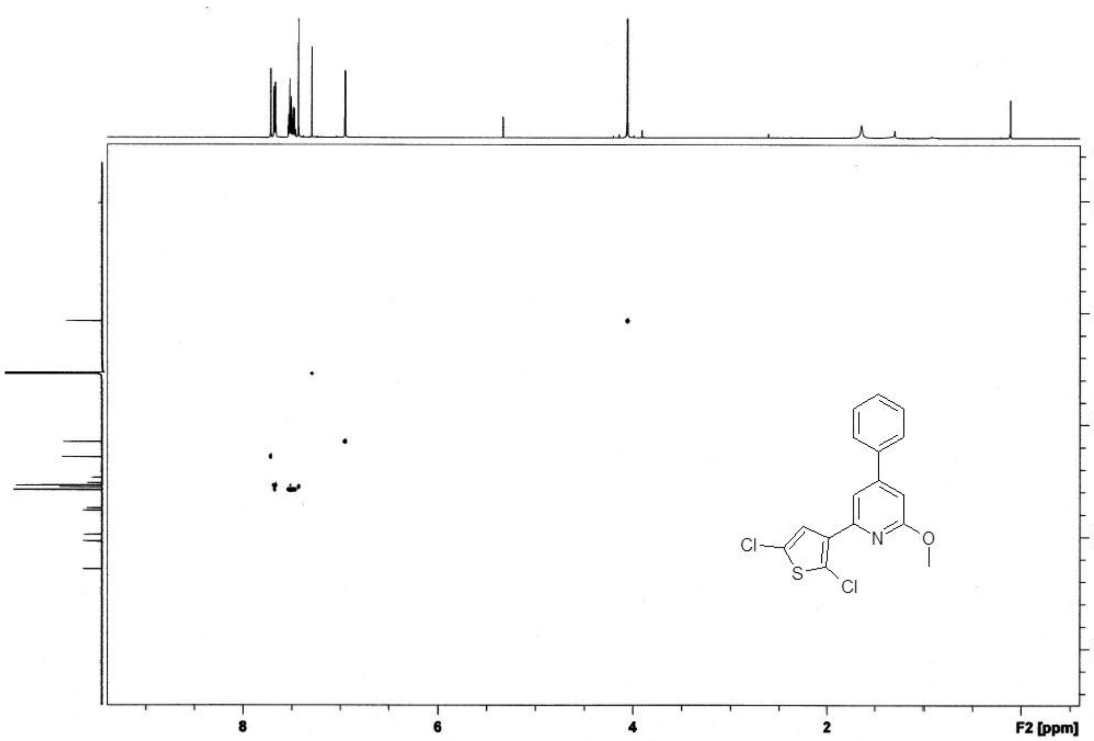

S 85: HSQC spectrum ($CDCl_3$, 500 MHz) of 2-(2,5-dichlorothiophen-3-yl)-6-methoxy-4-phenylpyridine (**6a**).

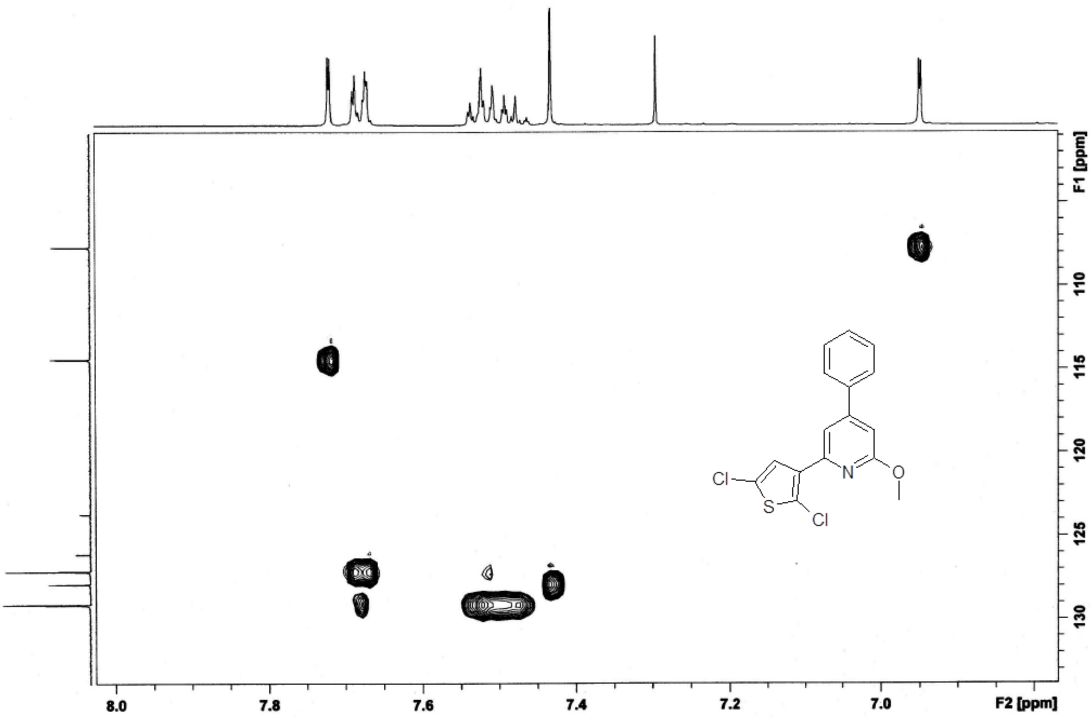

S 86: HSQC spectrum ($CDCl_3$, 500 MHz) of 2-(2,5-dichlorothiophen-3-yl)-6-methoxy-4-phenylpyridine (**6a**).

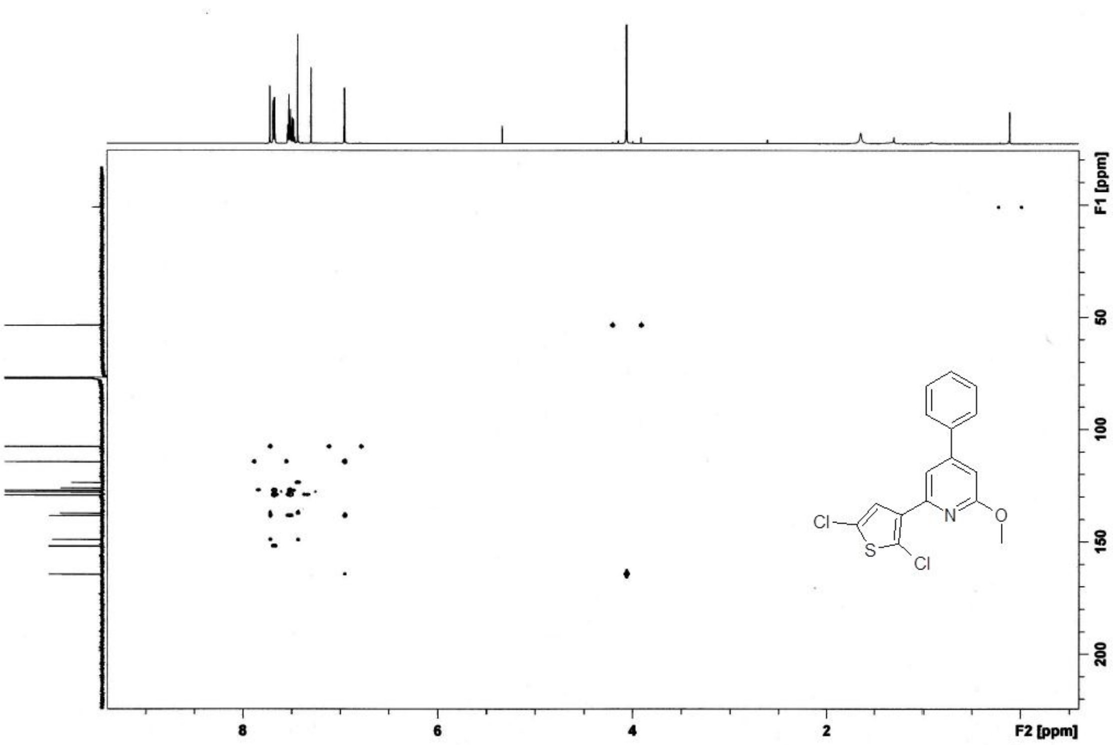

S 87: HMBC spectrum ($CDCl_3$, 500 MHz) of 2-(2,5-dichlorothiophen-3-yl)-6-methoxy-4-phenylpyridine (**6a**).

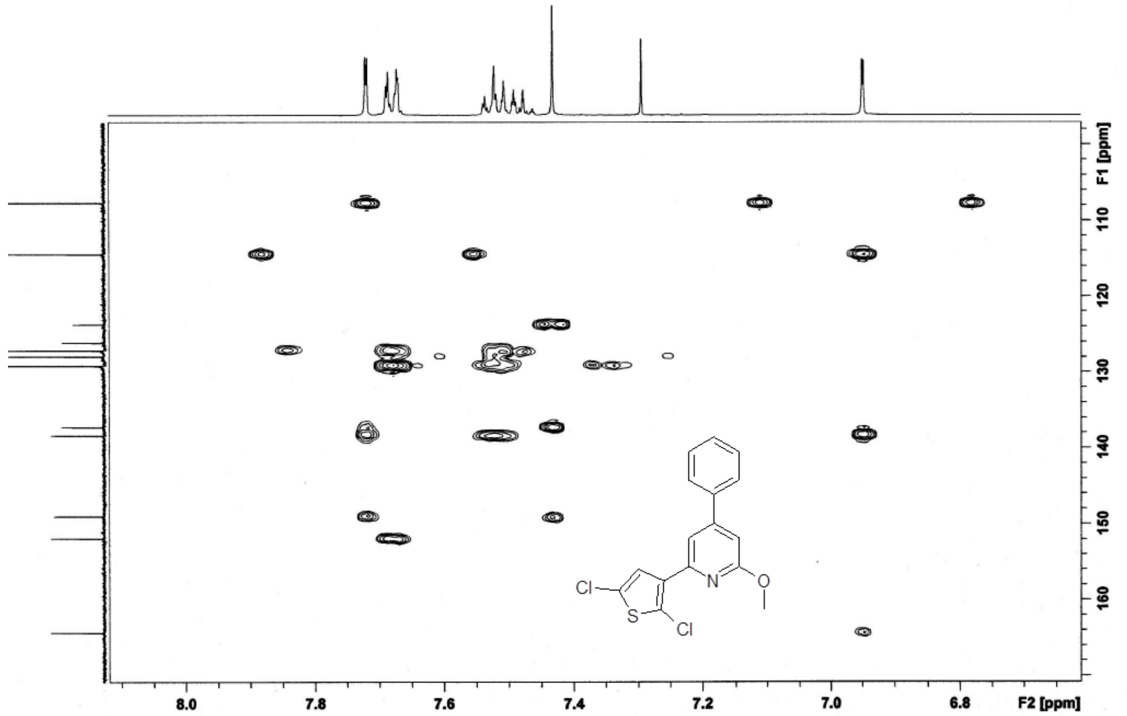

S 88: HMBC spectrum ($CDCl_3$, 500 MHz) of 2-(2,5-dichlorothiophen-3-yl)-6-methoxy-4-phenylpyridine (**6a**).

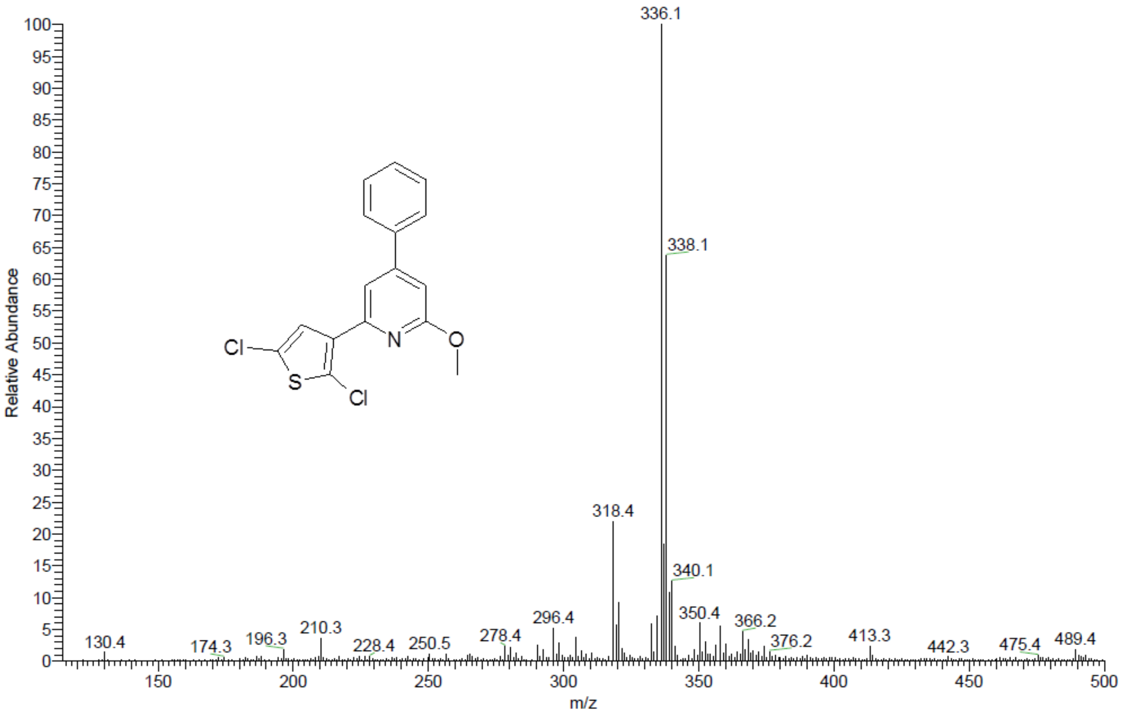

S 89: ESI mass spectrum of 2-(2,5-dichlorothiophen-3-yl)-6-methoxy-4-phenylpyridine (**6a**).

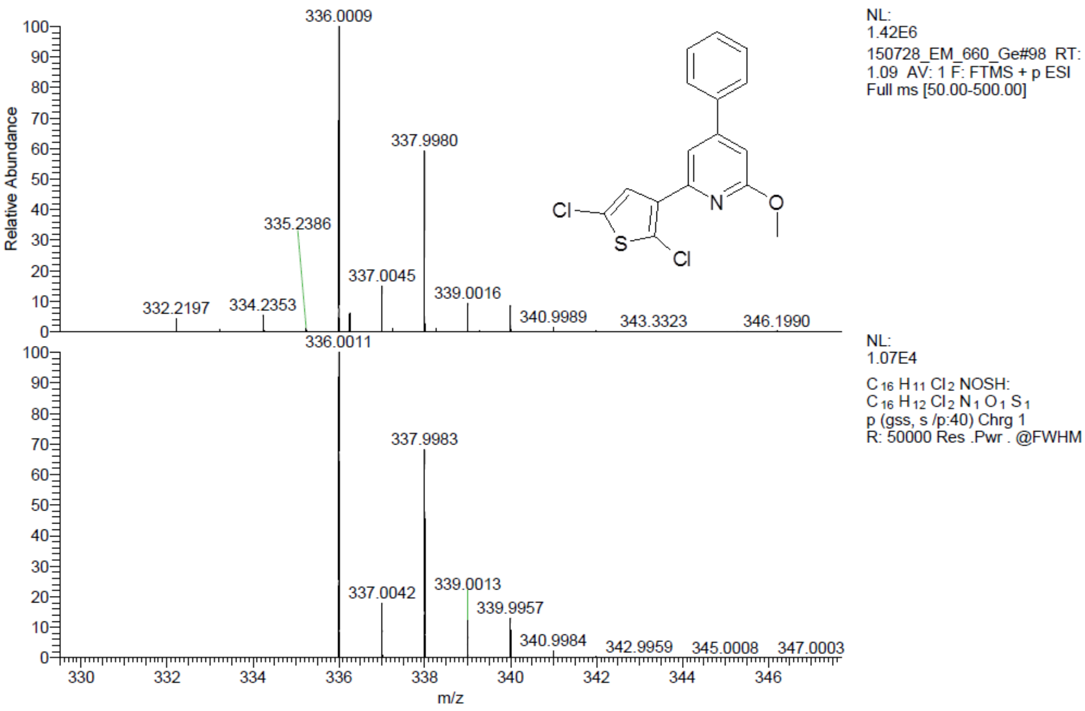

S 90: HRESI mass spectrum of 2-(2,5-dichlorothiophen-3-yl)-6-methoxy-4-phenylpyridine (**6a**).

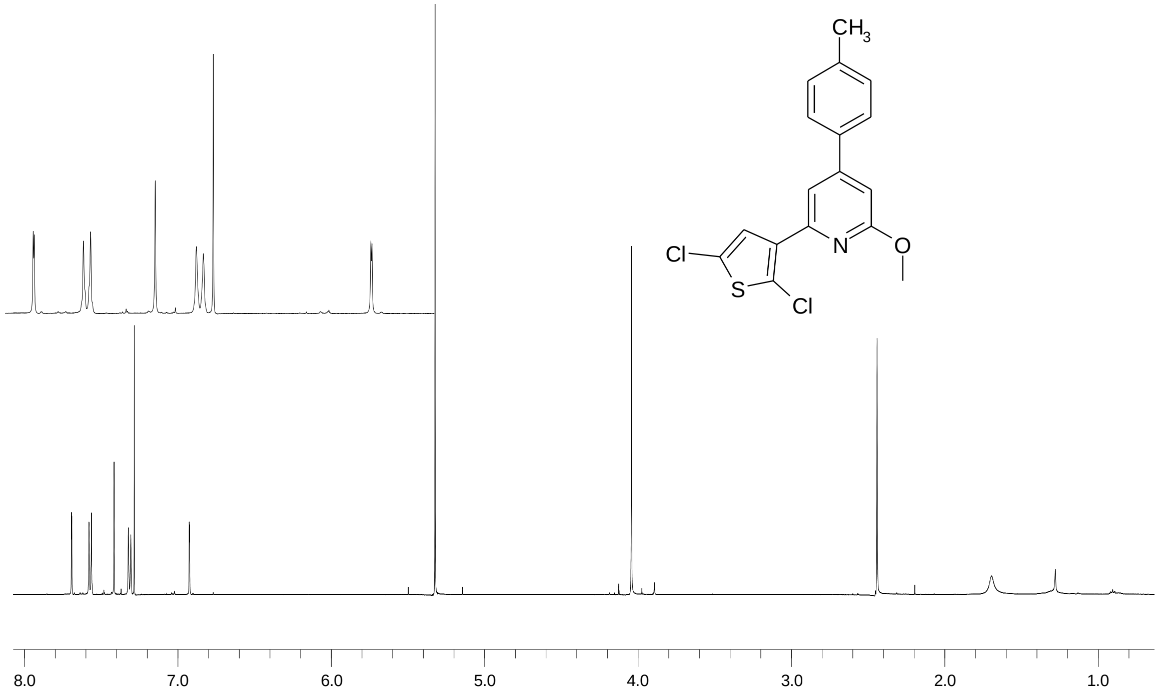

S 91: ^{1}H NMR spectrum ($CDCl_3$, 500 MHz) of 2-(2,5-dichlorothiophen-3-yl)-6-methoxy-4-p-tolylpyridine (**6b**).

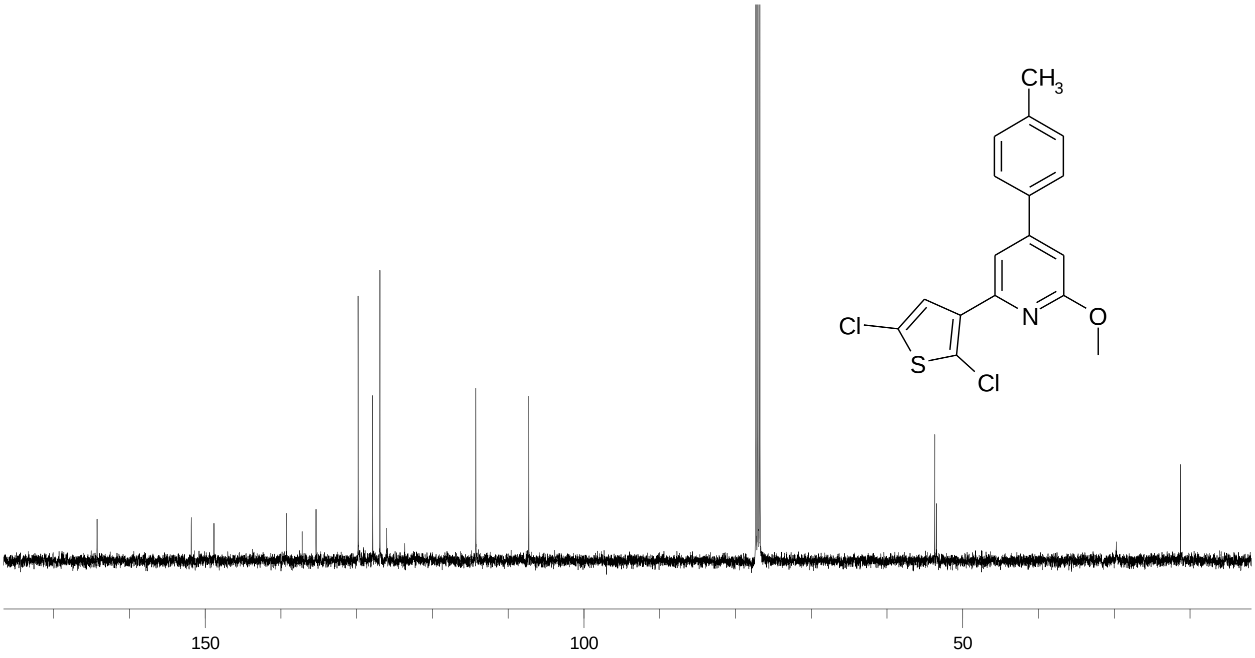

S 92: ^{13}C NMR spectrum ($CDCl_3$, 125MHz) of 2-(2,5-dichlorothiophen-3-yl)-6-methoxy-4-p-tolylpyridine (**6b**).

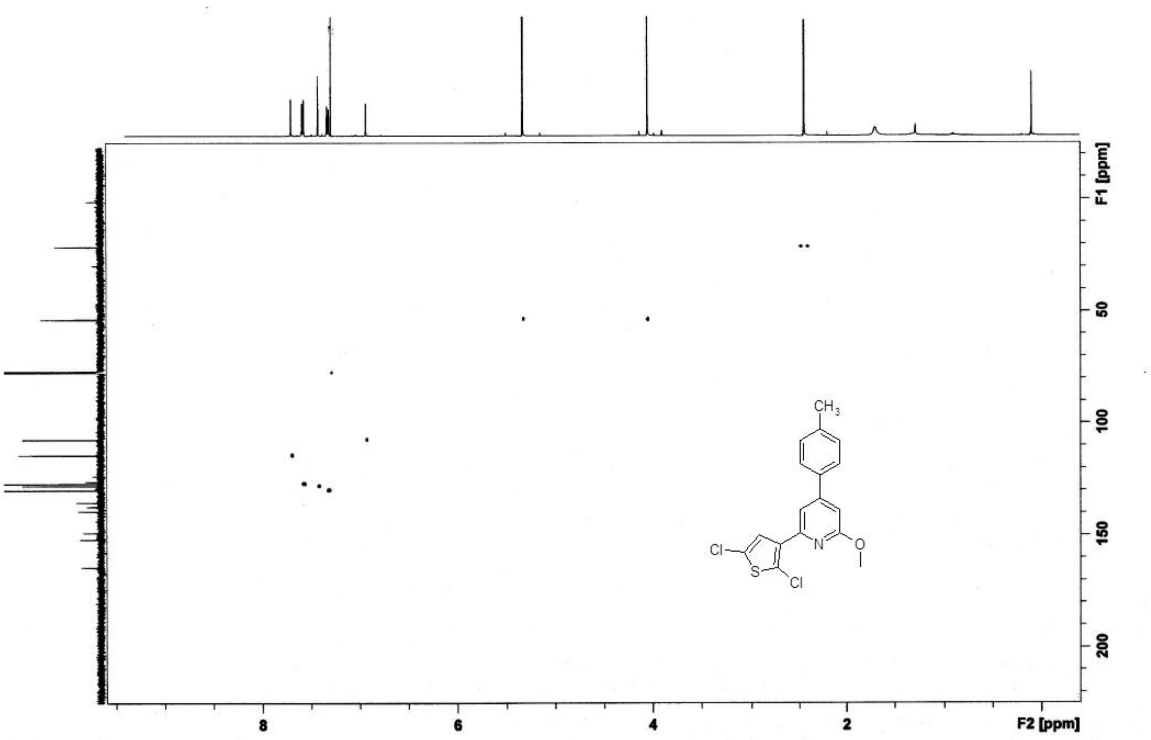

S 93: HSQC spectrum ($CDCl_3$, 500 MHz) of 2-(2,5-dichlorothiophen-3-yl)-6-methoxy-4-p-tolylpyridine (**6b**).

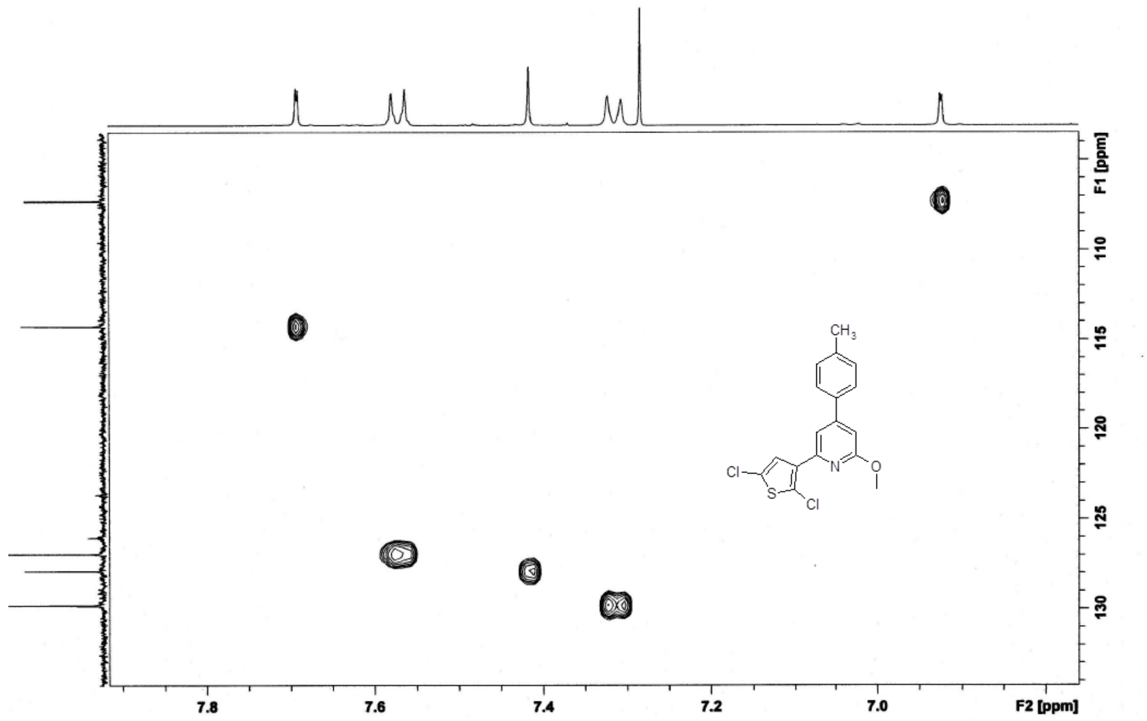

S 94: HSQC spectrum ($CDCl_3$, 500 MHz) of 2-(2,5-dichlorothiophen-3-yl)-6-methoxy-4-p-tolylpyridine (**6b**).

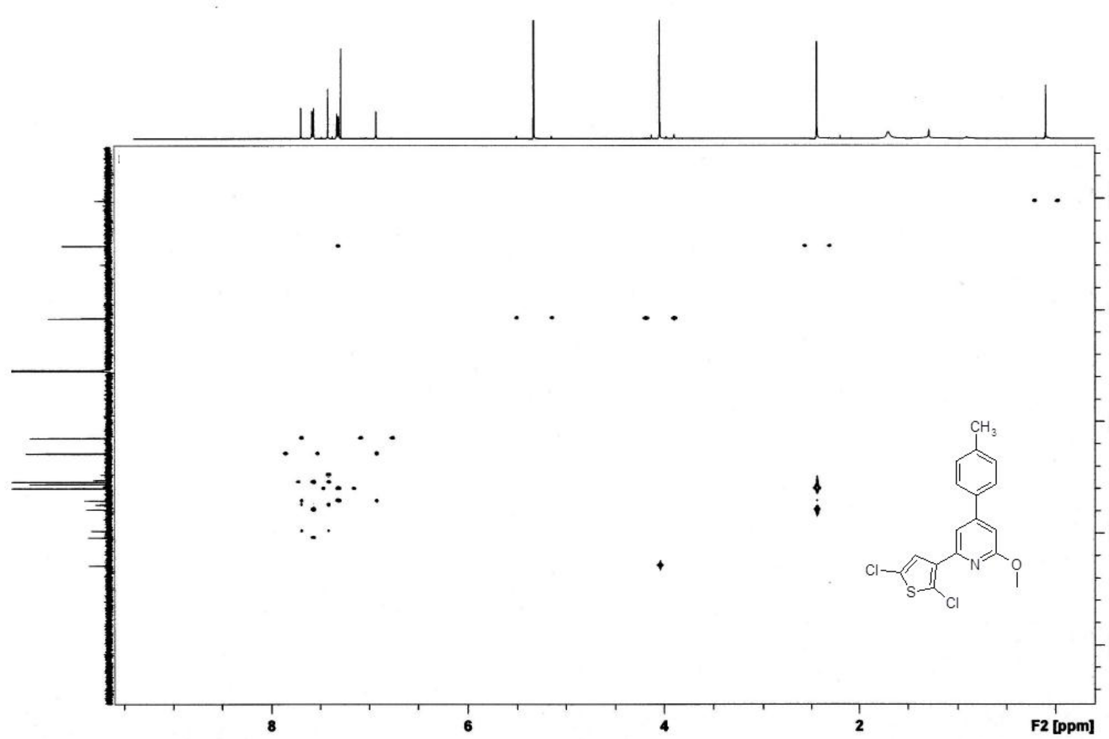

S 95: HMBC spectrum ($CDCl_3$, 500 MHz) of 2-(2,5-dichlorothiophen-3-yl)-6-methoxy-4-p-tolylpyridine (**6b**).

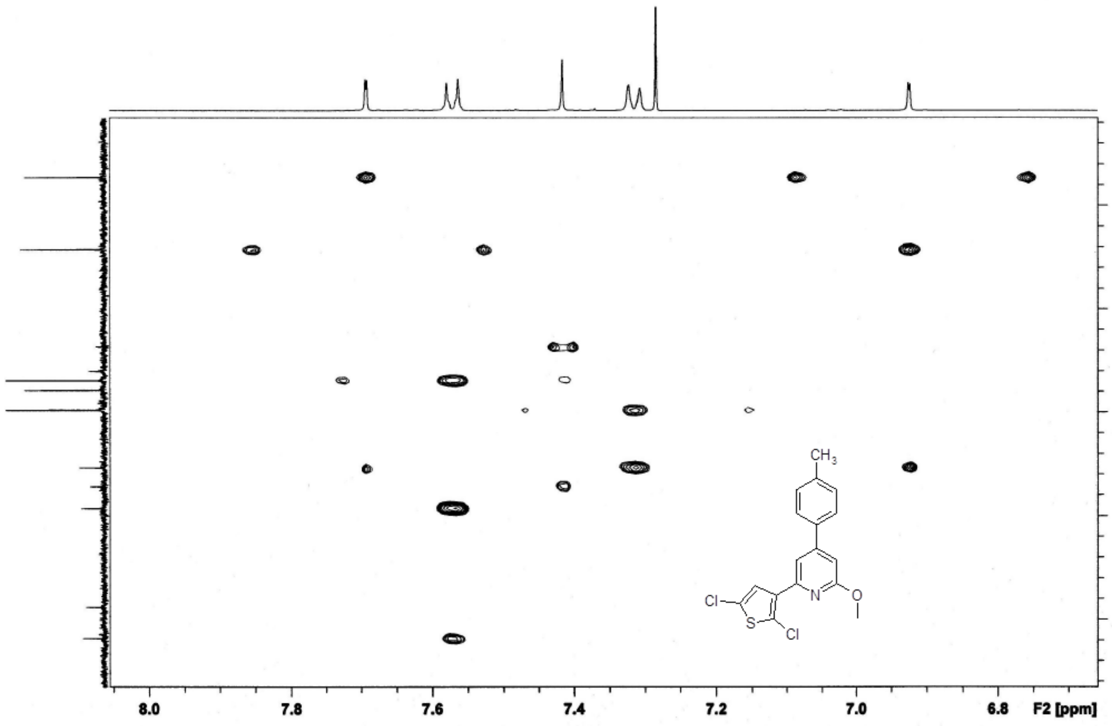

S 96: HMBC spectrum ($CDCl_3$, 500 MHz) of 2-(2,5-dichlorothiophen-3-yl)-6-methoxy-4-p-tolylpyridine (**6b**).

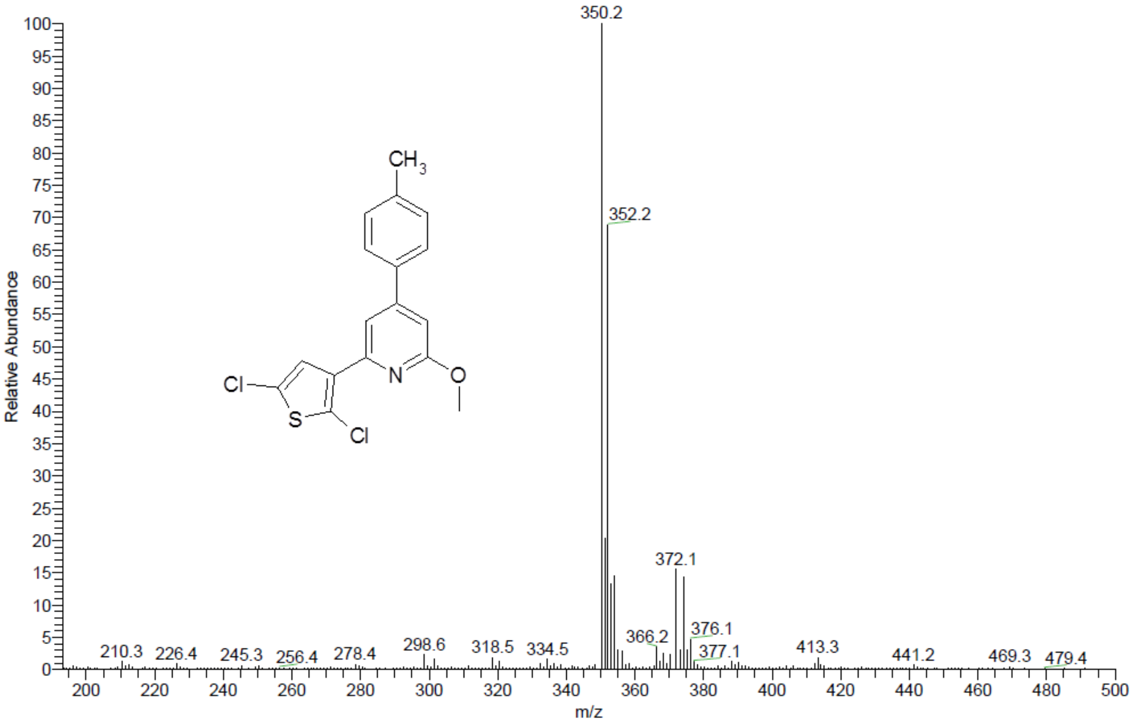

S 97: ESI mass spectrum of 2-(2,5-dichlorothiophen-3-yl)-6-methoxy-4-p-tolylpyridine (**6b**).

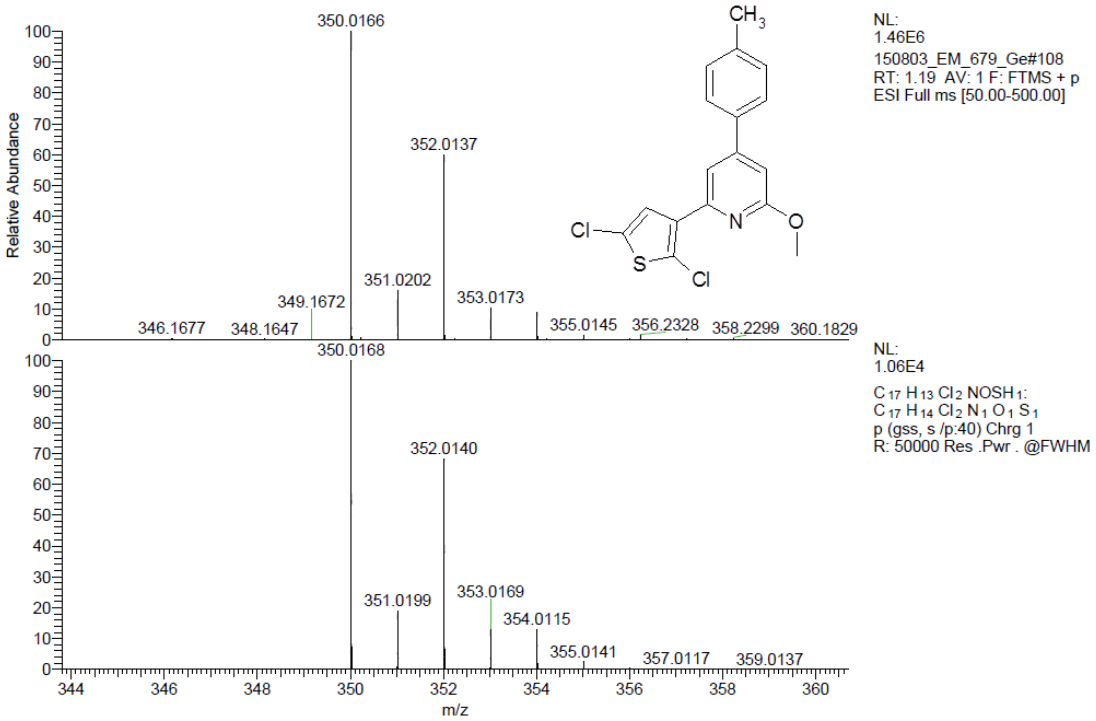

S 98: HRESI mass spectrum of 2-(2,5-dichlorothiophen-3-yl)-6-methoxy-4-p-tolylpyridine (**6b**).

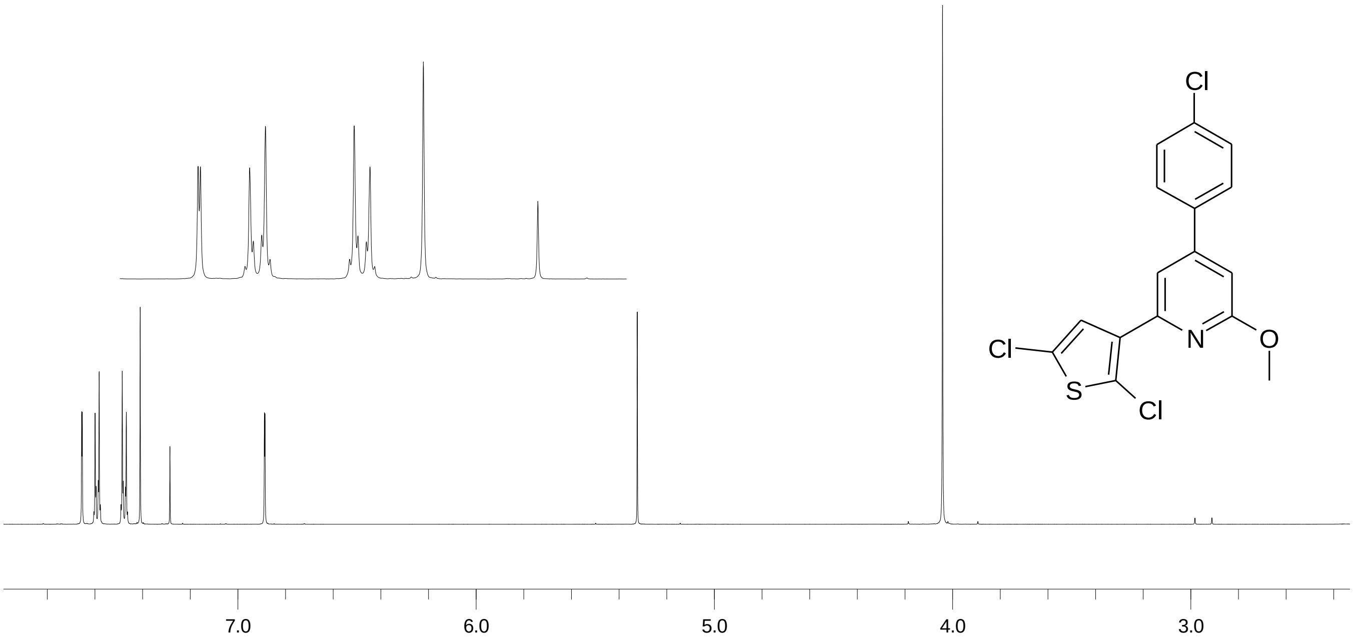

S 99: ^{1}H NMR spectrum ($CDCl_{3,}$ 500 MHz) of 4-(4-chlorophenyl)-2-(2,5-dichlorothiophen-3-yl)-6-methoxypyridine (**6c**).

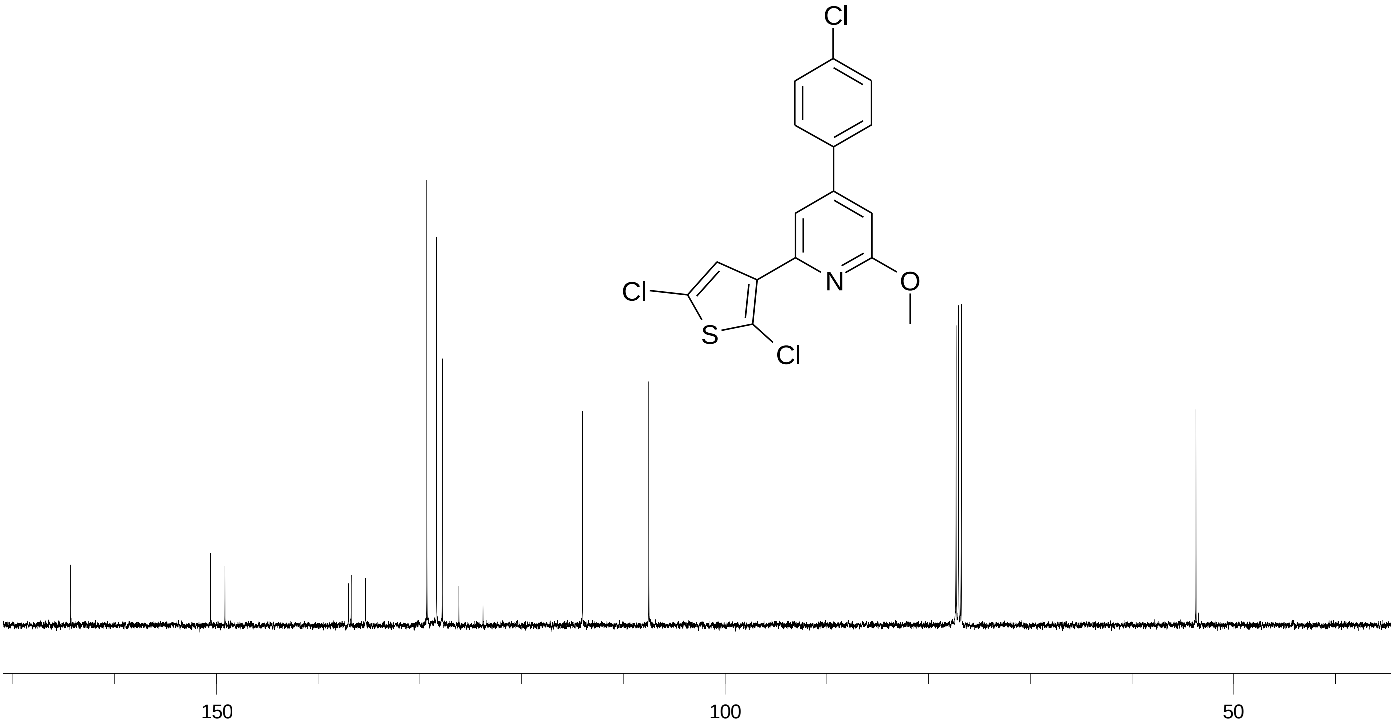

S 100: ^{13}C NMR spectrum ($CDCl_{3,}$ 125MHz) of 4-(4-chlorophenyl)-2-(2,5-dichlorothiophen-3-yl)-6-methoxypyridine (**6c**).

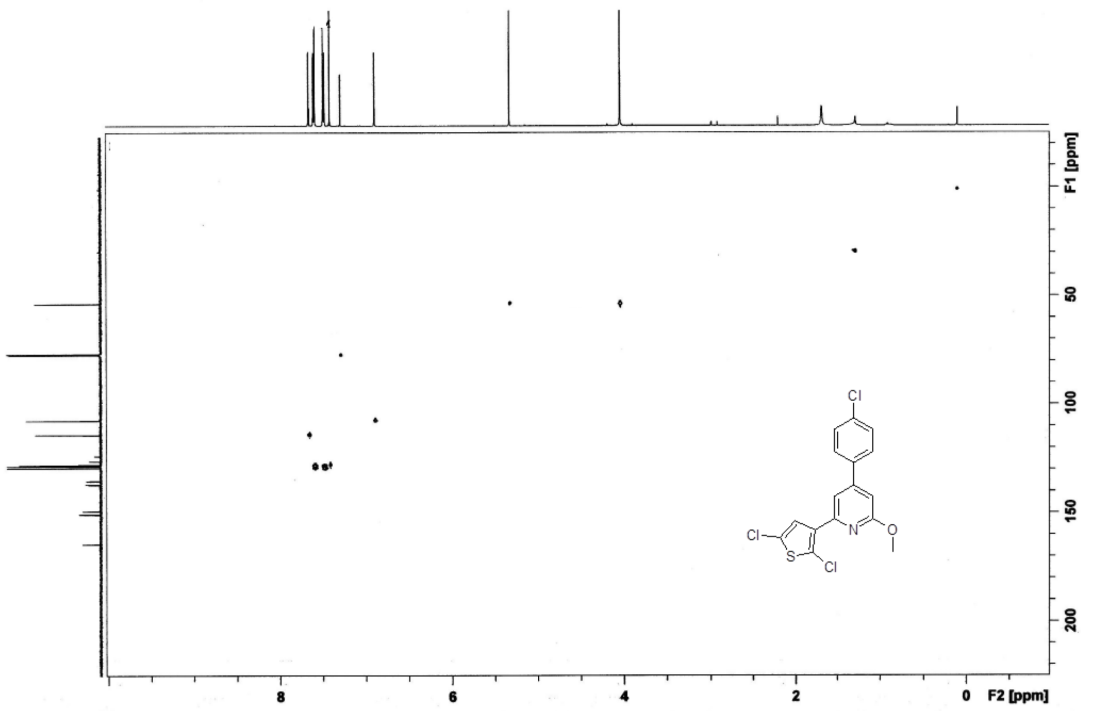

S 101: HSQC spectrum ($CDCl_3$, 500 MHz) of 4-(4-chlorophenyl)-2-(2,5-dichlorothiophen-3-yl)-6-methoxypyridine (**6c**).

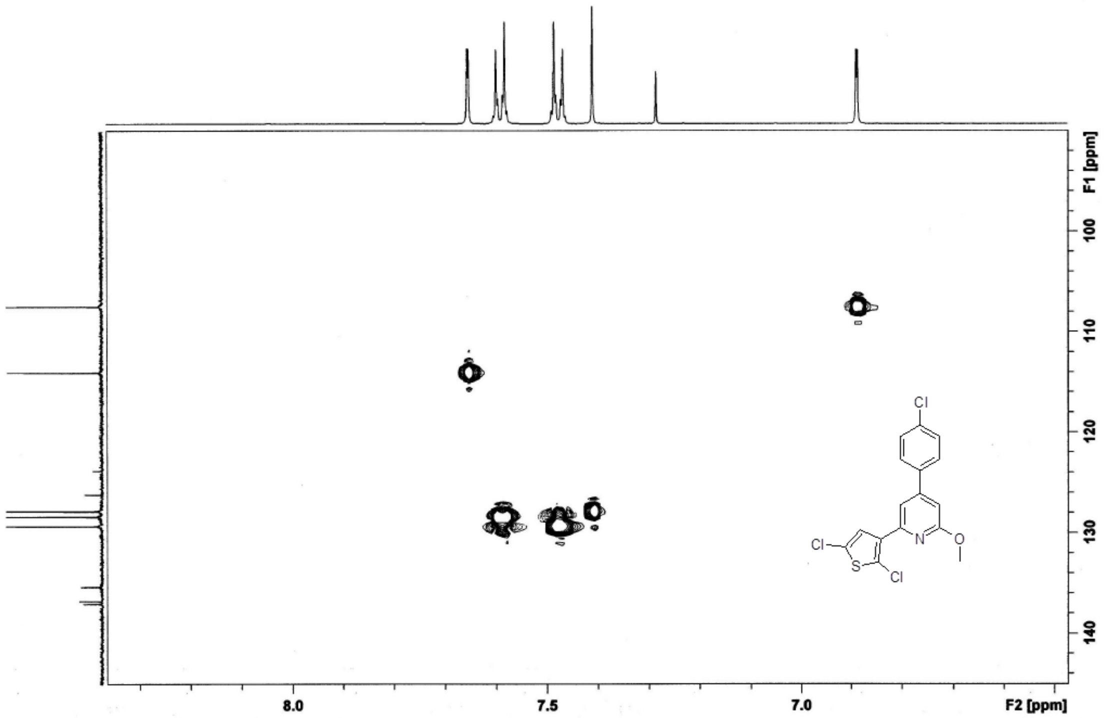

S 102: HSQC spectrum ($CDCl_3$, 500 MHz) of 4-(4-chlorophenyl)-2-(2,5-dichlorothiophen-3-yl)-6-methoxypyridine (**6c**).

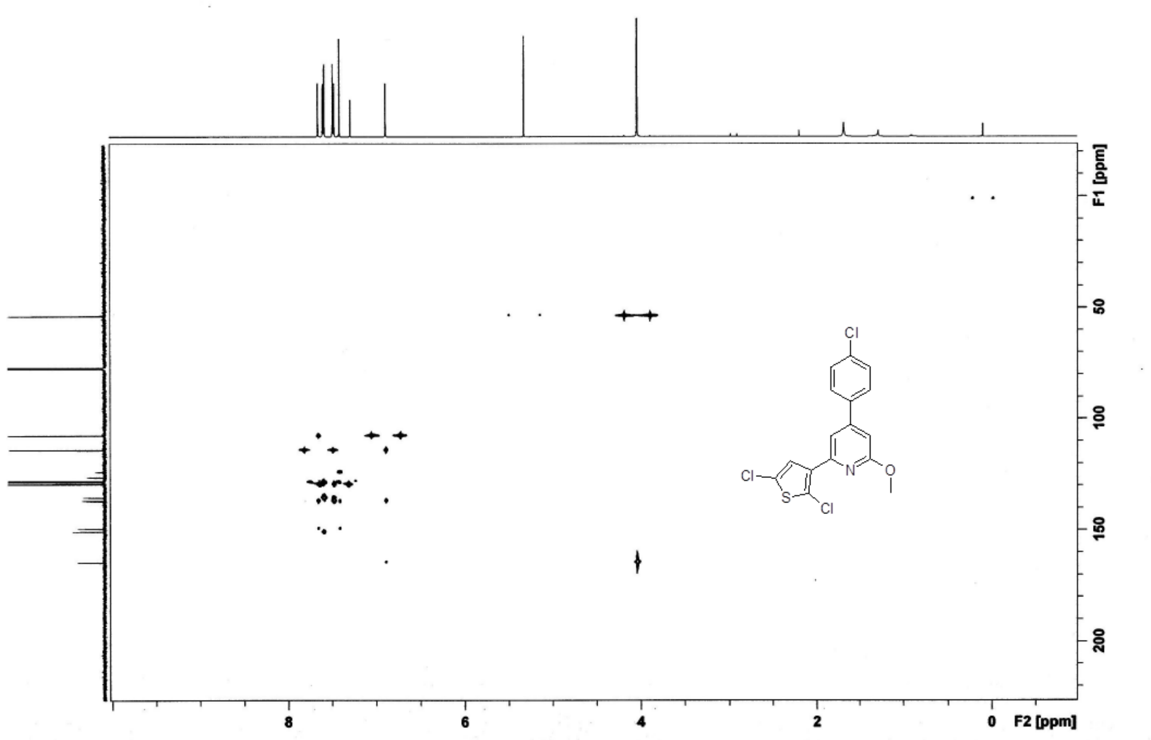

S 103: HMBC spectrum ($CDCl_3$, 500 MHz) of 4-(4-chlorophenyl)-2-(2,5-dichlorothiophen-3-yl)-6-methoxypyridine (**6c**).

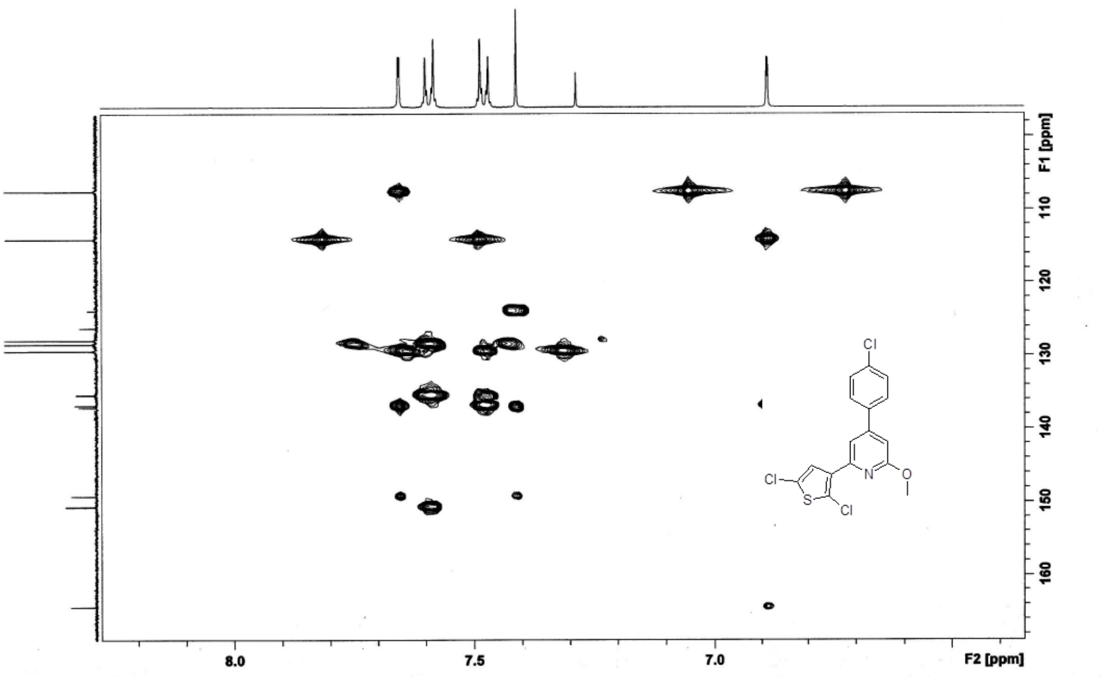

S 104: HMBC spectrum ($CDCl_3$, 500 MHz) of 4-(4-chlorophenyl)-2-(2,5-dichlorothiophen-3-yl)-6-methoxypyridine (**6c**).

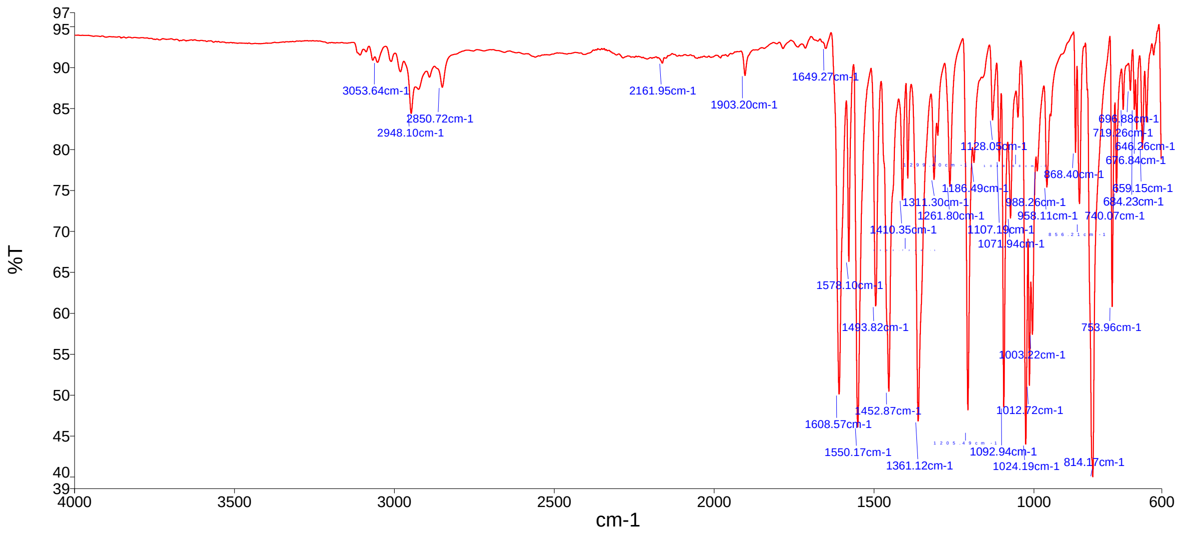

S 105: IR spectrum of 4-(4-chlorophenyl)-2-(2,5-dichlorothiophen-3-yl)-6-methoxypyridine (**6c**).

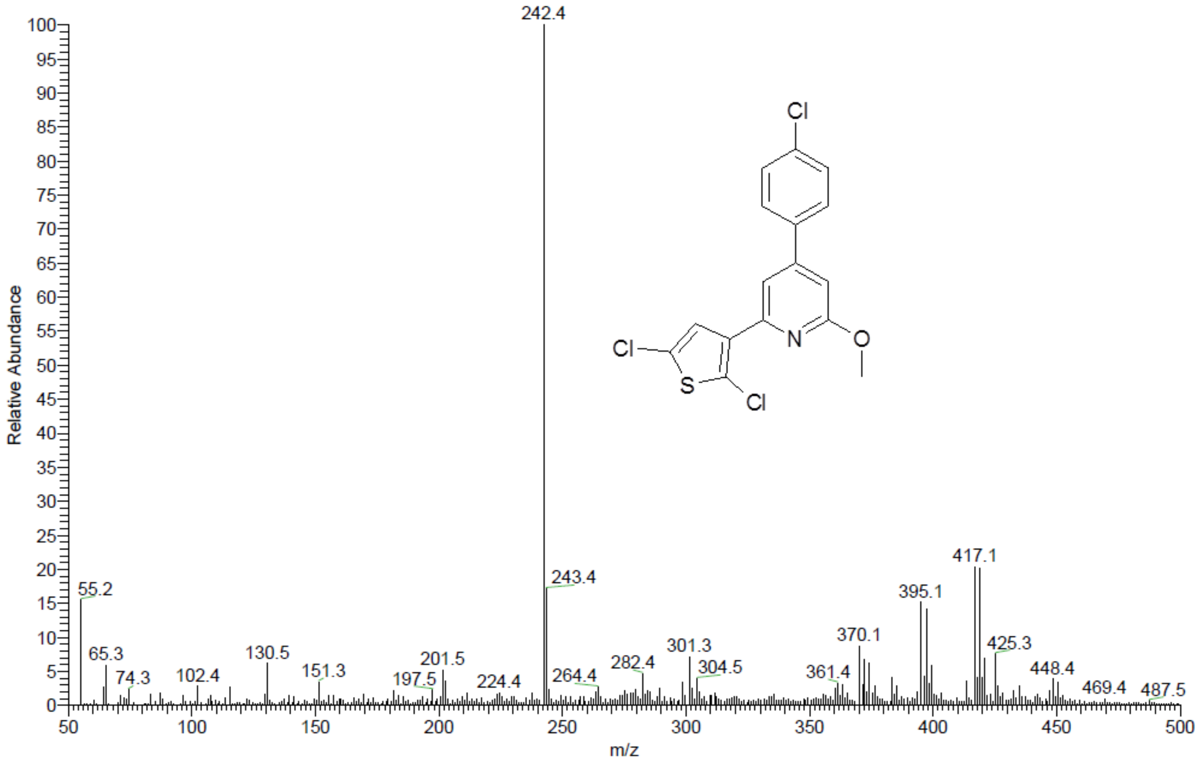

S 106: ESI mass spectrum of 4-(4-chlorophenyl)-2-(2,5-dichlorothiophen-3-yl)-6-methoxypyridine (**6c**).

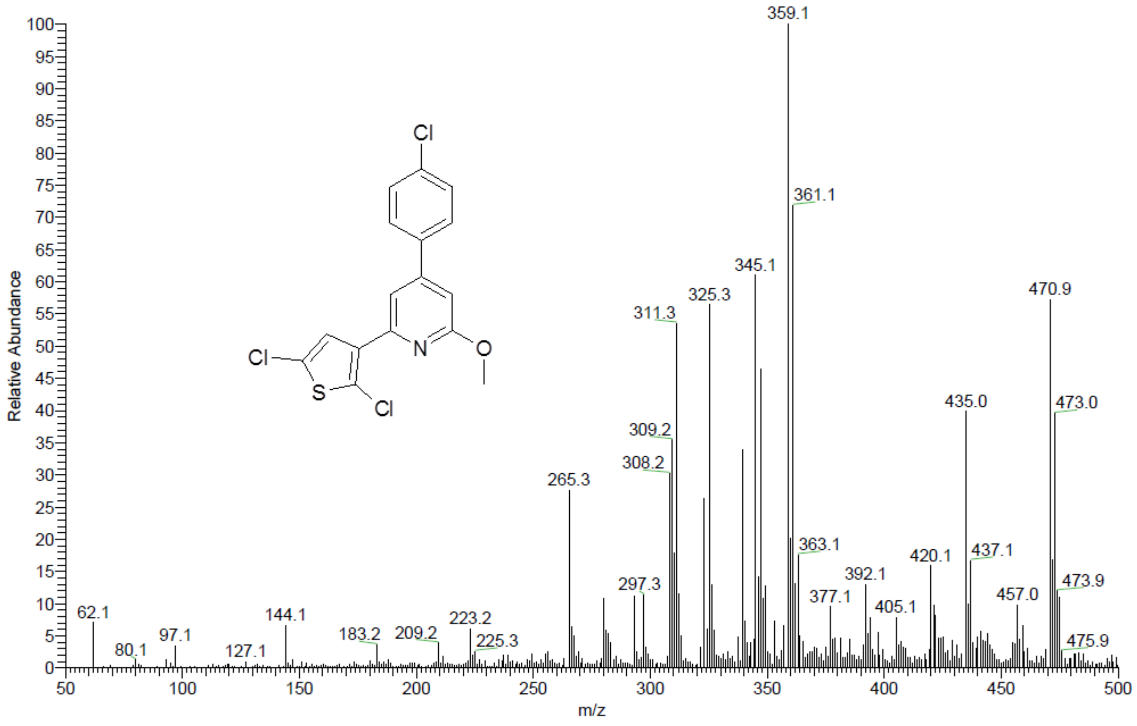

S 107: ESI mass spectrum of 4-(4-chlorophenyl)-2-(2,5-dichlorothiophen-3-yl)-6-methoxypyridine (**6c**).

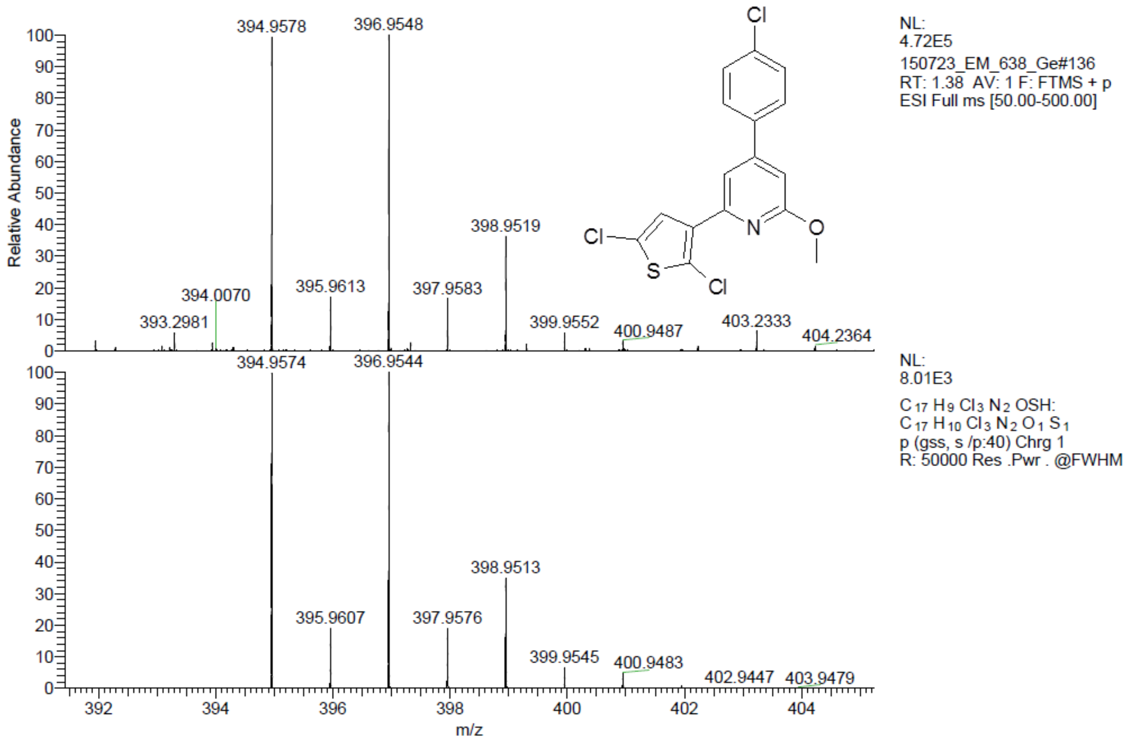

S 108: HRESI mass spectrum of 4-(4-chlorophenyl)-2-(2,5-dichlorothiophen-3-yl)-6-methoxypyridine (**6c**).

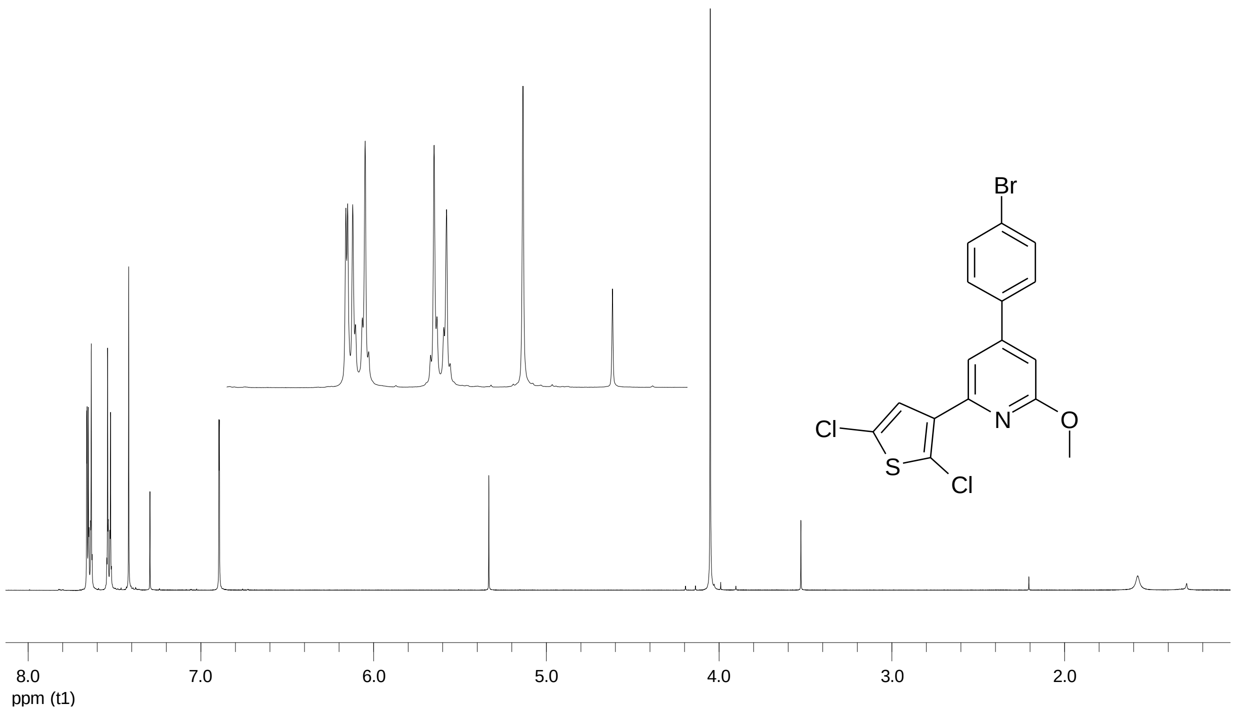

S 109: ^{1}H NMR spectrum ($CDCl_3$, 500 MHz) of 4-(4-bromophenyl)-2-(2,5-dichlorothiophen-3-yl)-6-methoxypyridine (**6d**).

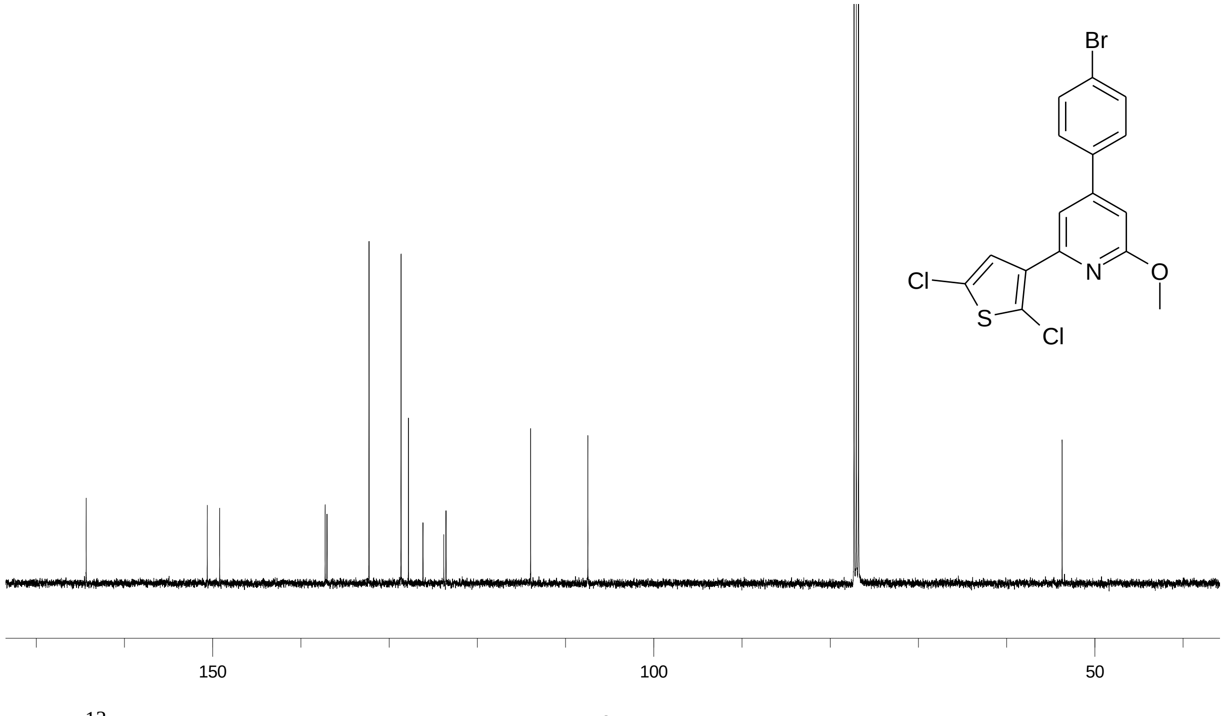

S 110: ^{13}C NMR spectrum ($CDCl_3$, 125MHz) of 4-(4-bromophenyl)-2-(2,5-dichlorothiophen-3-yl)-6-methoxypyridine (**6d**).

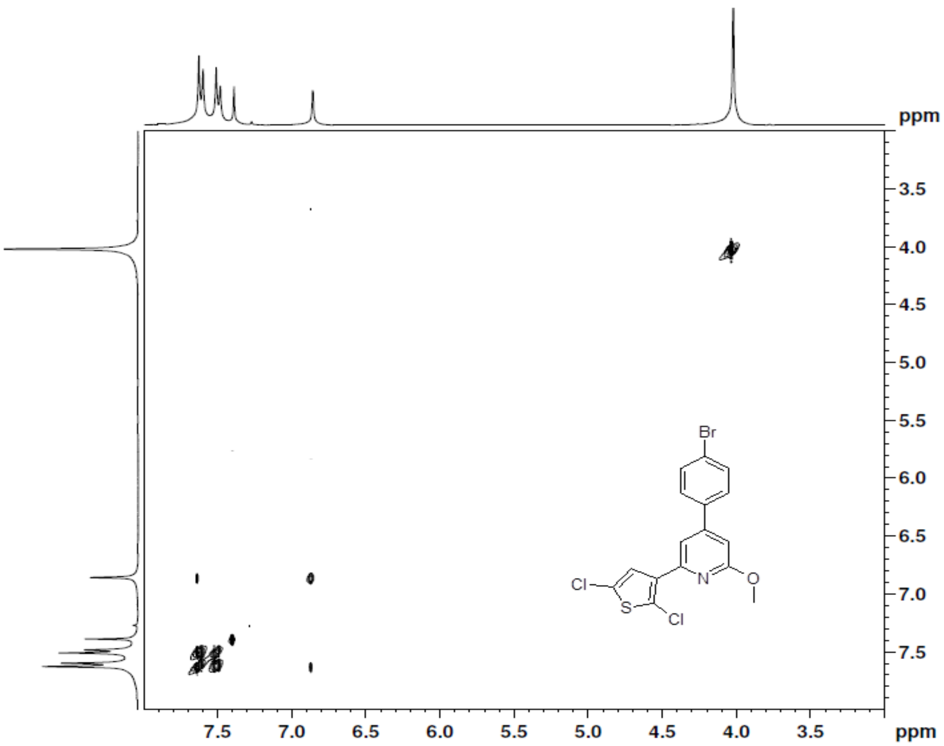

S 111: H,H COSY spectrum ($CDCl_3$, 300 MHz) of 4-(4-bromophenyl)-2-(2,5-dichlorothiophen-3-yl)-6-methoxypyridine (**6d**).

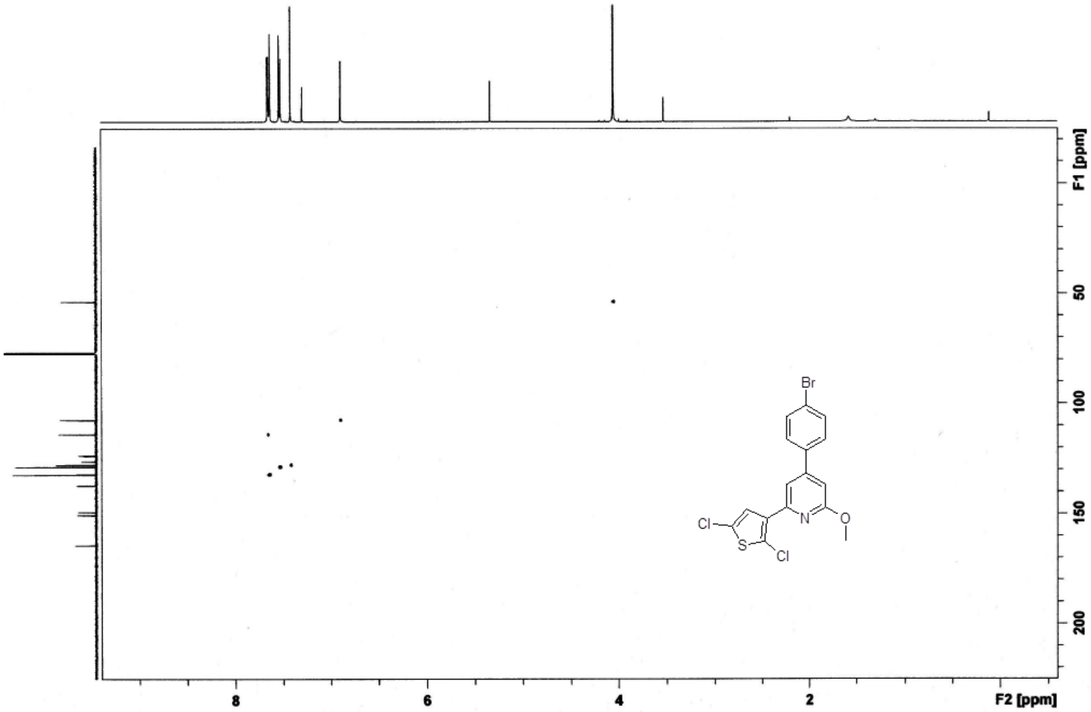

S 112: HSQC spectrum ($CDCl_3$, 500 MHz) of 4-(4-bromophenyl)-2-(2,5-dichlorothiophen-3-yl)-6-methoxypyridine (**6d**).

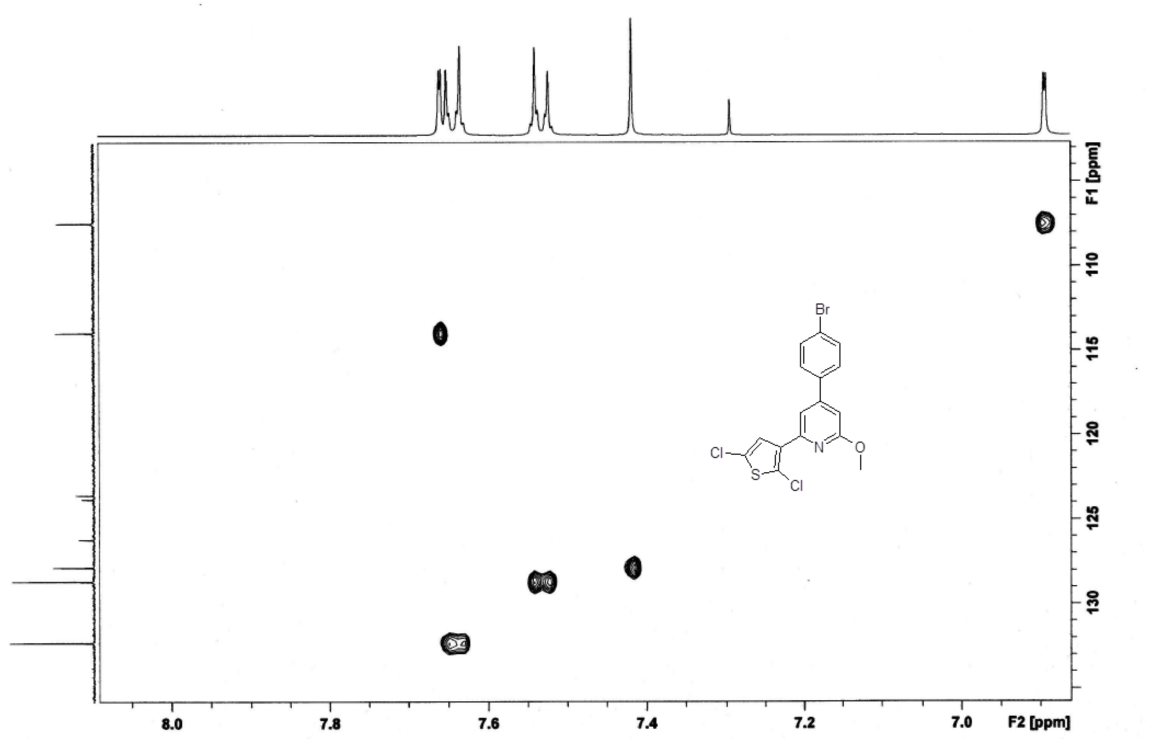

S 113: HSQC spectrum ($CDCl_3$, 500 MHz) of 4-(4-bromophenyl)-2-(2,5-dichlorothiophen-3-yl)-6-methoxypyridine (**6d**).

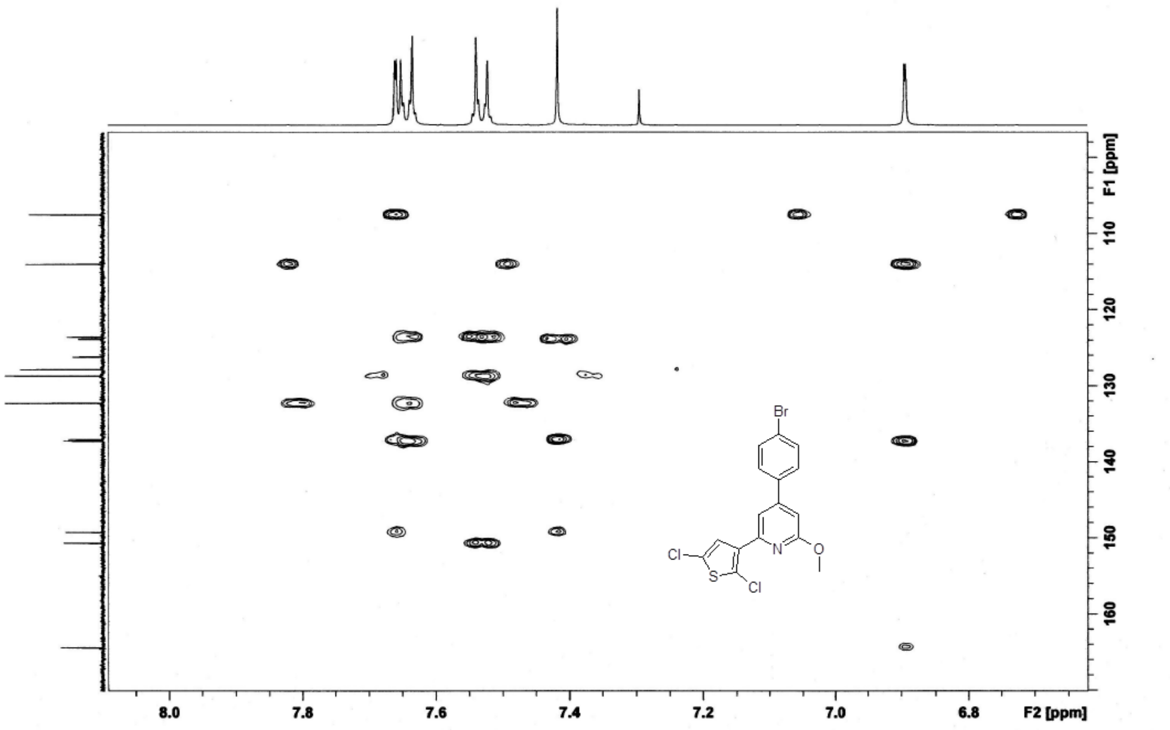

S 114: HMBC spectrum ($CDCl_3$, 500 MHz) of 4-(4-bromophenyl)-2-(2,5-dichlorothiophen-3-yl)-6-methoxypyridine (**6d**).

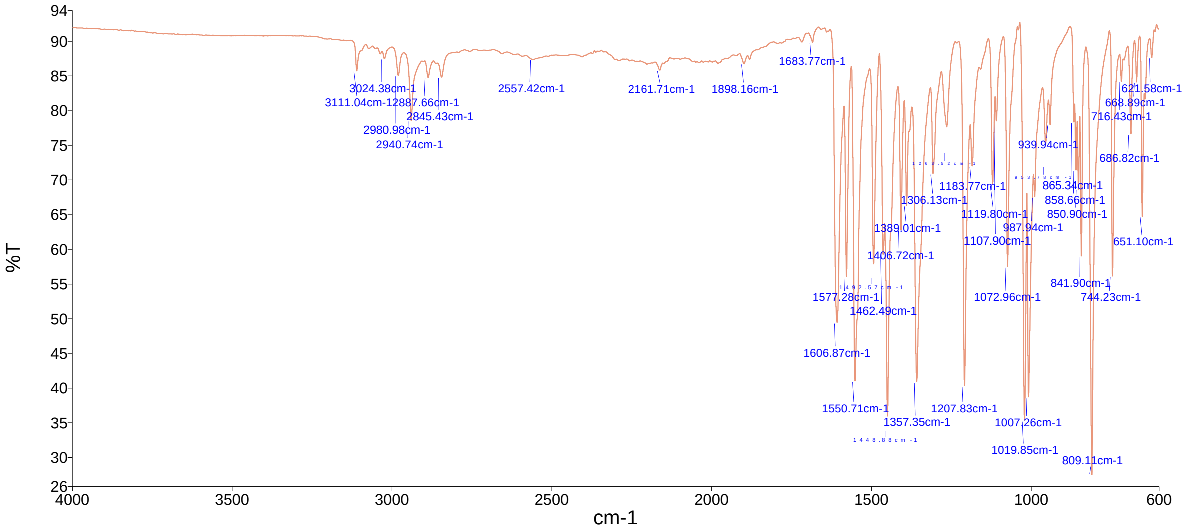

S 115: IR spectrum of 4-(4-bromophenyl)-2-(2,5-dichlorothiophen-3-yl)-6-methoxypyridine (**6d**).

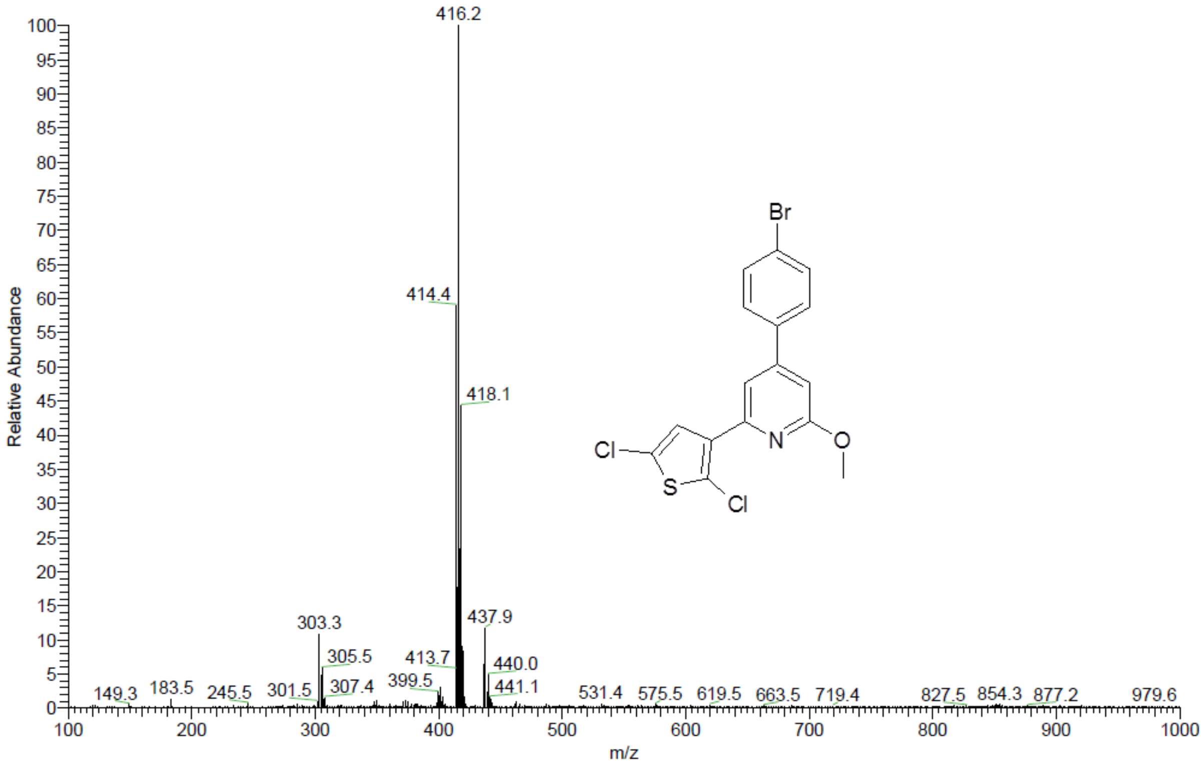

S 116: ESI mass spectrum of 4-(4-bromophenyl)-2-(2,5-dichlorothiophen-3-yl)-6-methoxypyridine (**6d**).

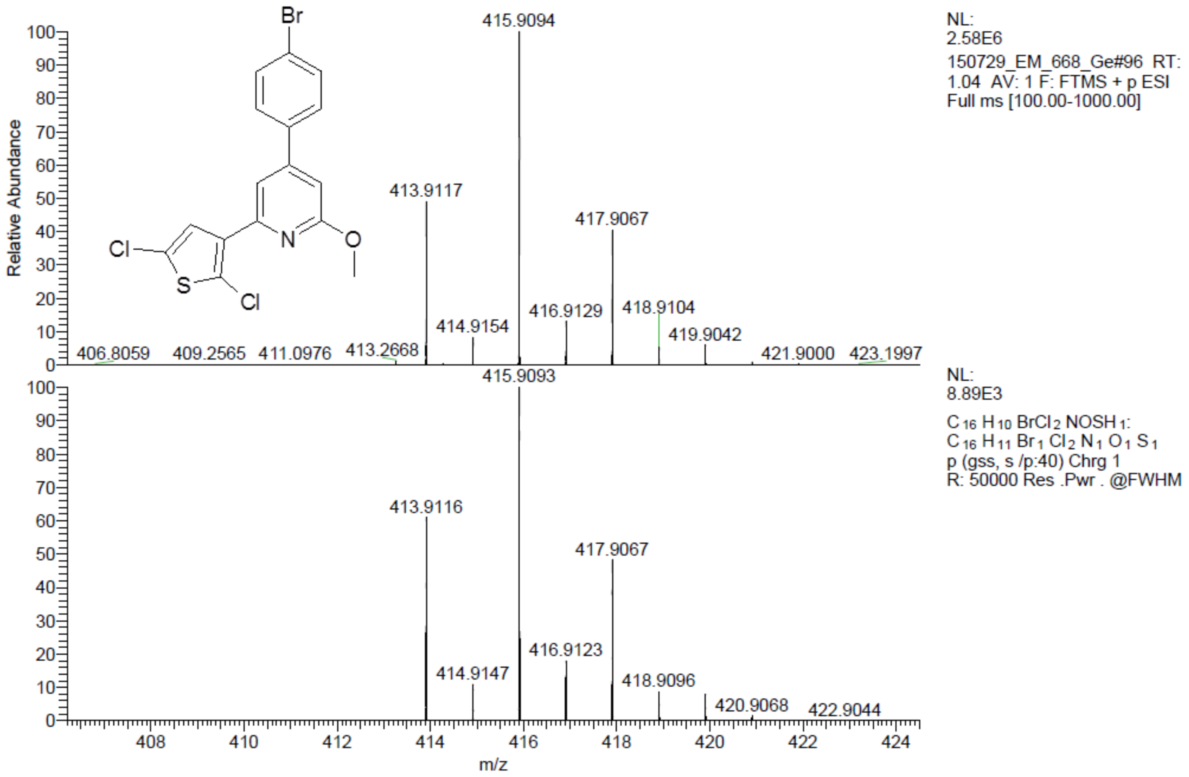

S 117: HRESI mass spectrum of 4-(4-bromophenyl)-2-(2,5-dichlorothiophen-3-yl)-6-methoxypyridine (**6d**).

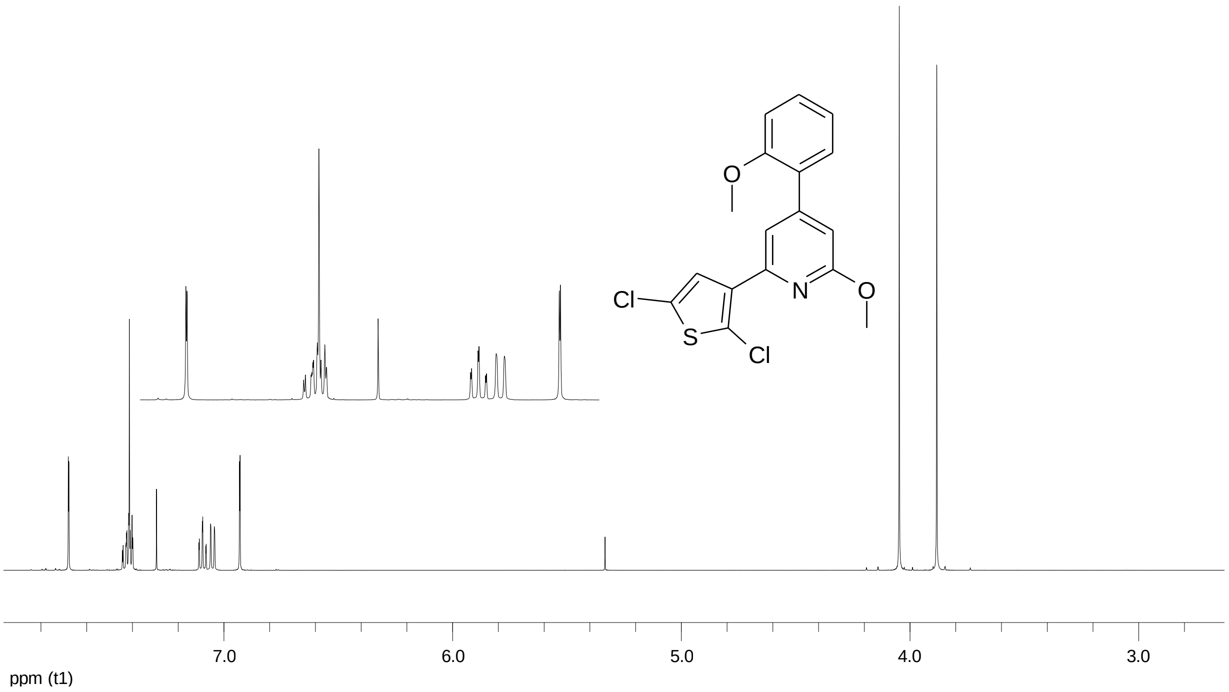

S 118: ^{1}H NMR spectrum ($CDCl_3$, 500 MHz) of 2-(2,5-dichlorothiophen-3-yl)-6-methoxy-4-(2-methoxyphenyl)pyridine (**6e**).

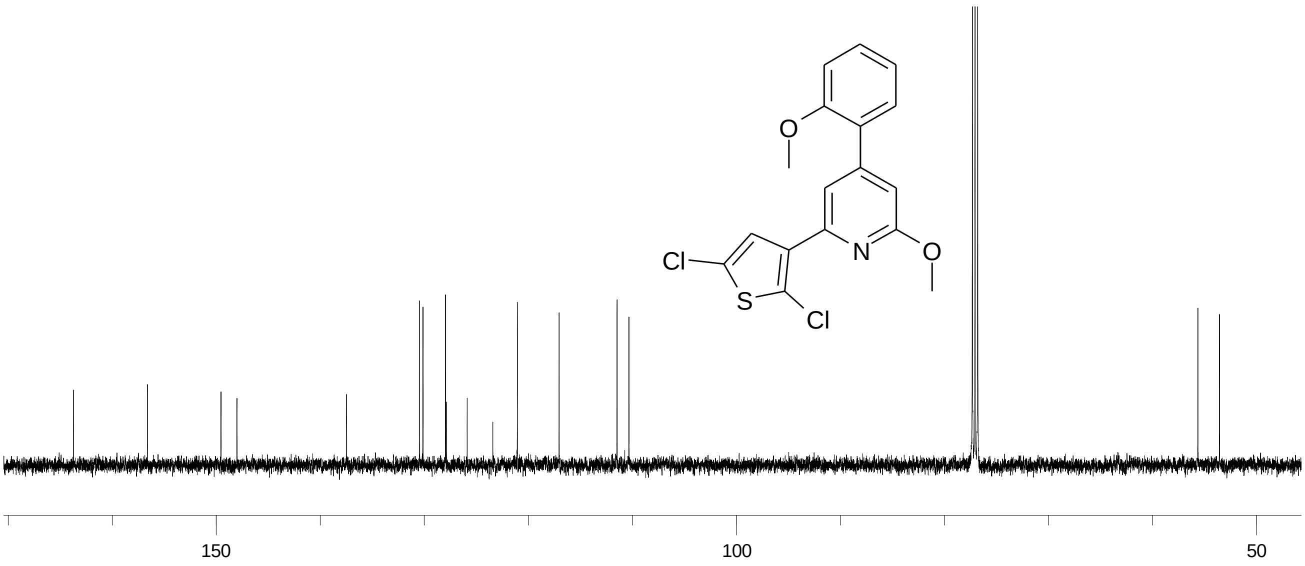

S 119: ^{13}C NMR spectrum ($CDCl_3$, 125MHz) of 2-(2,5-dichlorothiophen-3-yl)-6-methoxy-4-(2-methoxyphenyl)pyridine (**6e**).

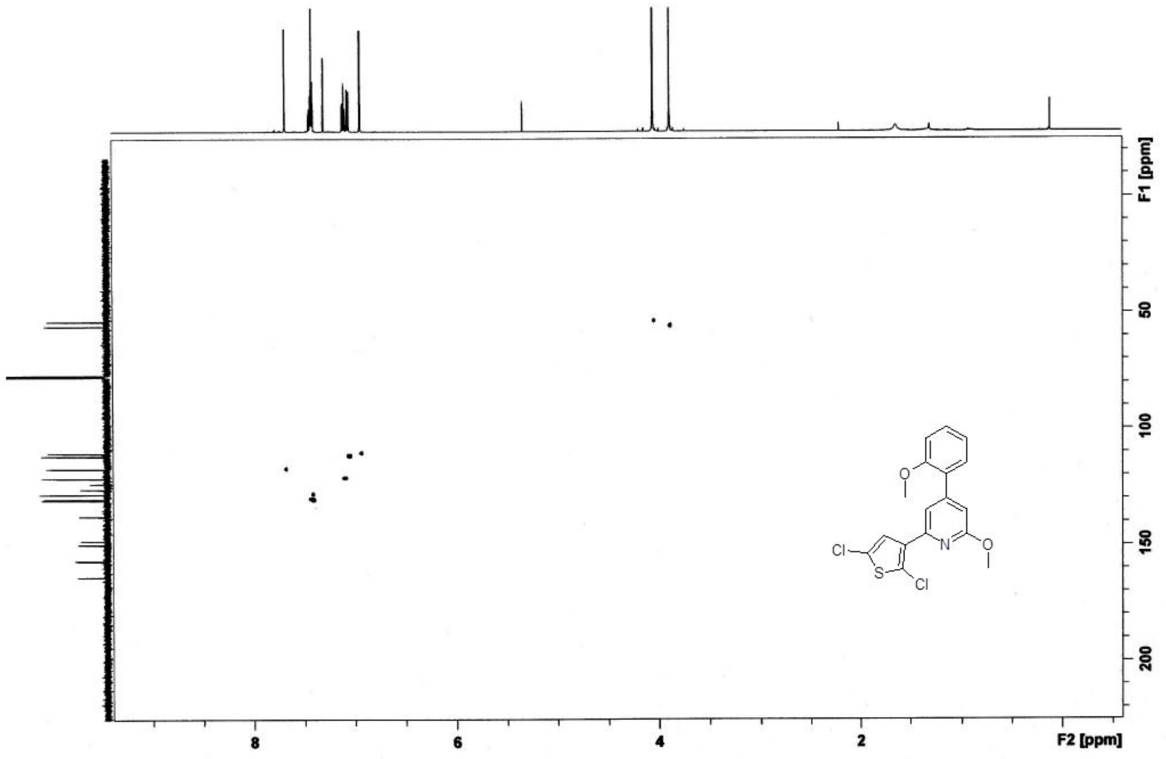

S 120: HSQC spectrum ($CDCl_3$, 500 MHz) of 2-(2,5-dichlorothiophen-3-yl)-6-methoxy-4-(2-methoxyphenyl)pyridine (**6e**).

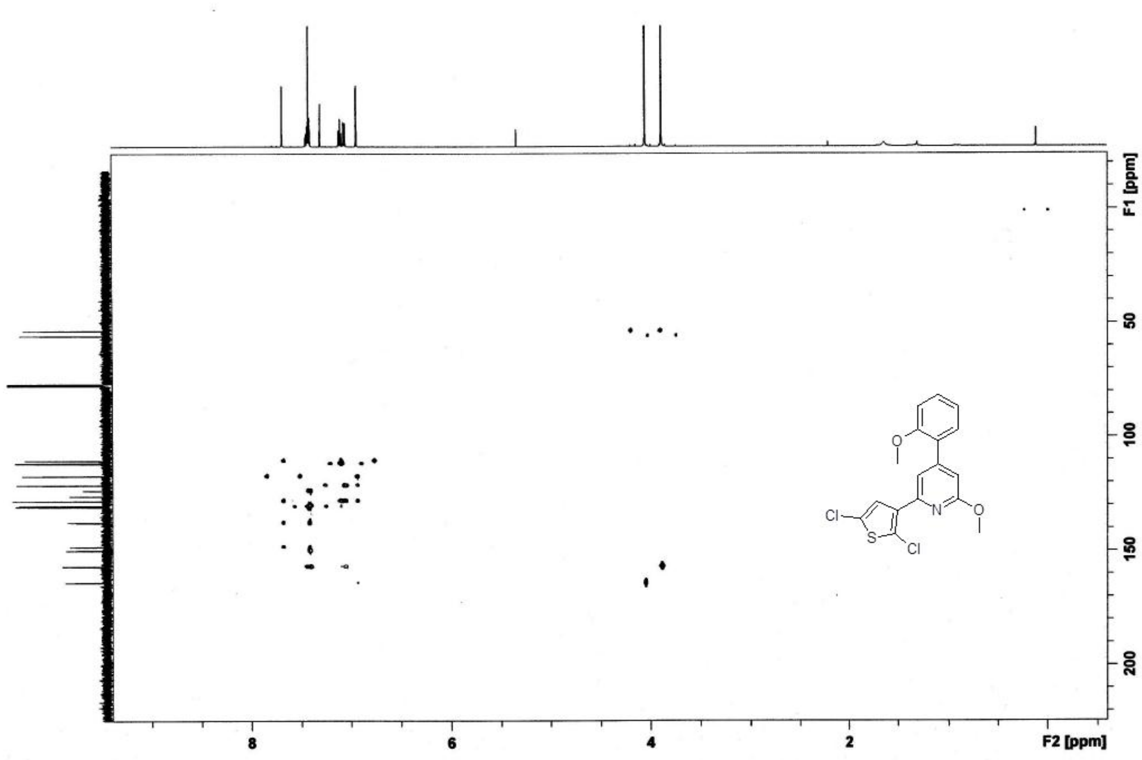

S 121: HMBC spectrum ($CDCl_3$, 500 MHz) of 2-(2,5-dichlorothiophen-3-yl)-6-methoxy-4-(2-methoxyphenyl)pyridine (**6e**).

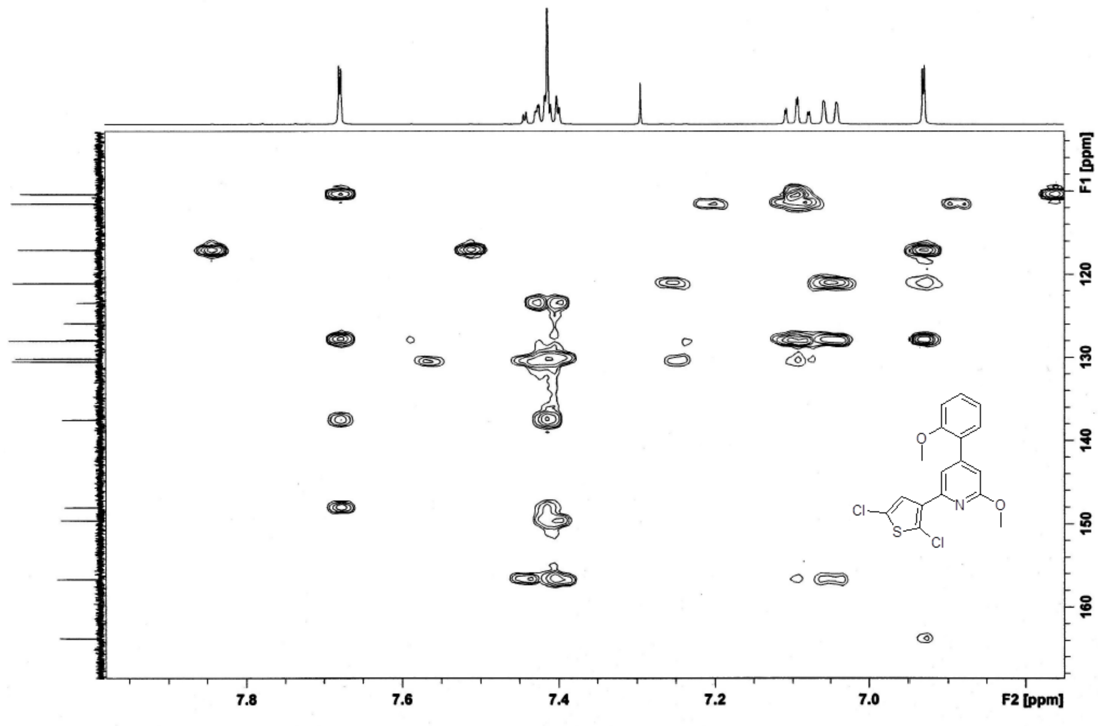

S 122: HMBC spectrum ($CDCl_3$, 500 MHz) of 2-(2,5-dichlorothiophen-3-yl)-6-methoxy-4-(2-methoxyphenyl)pyridine (**6e**).

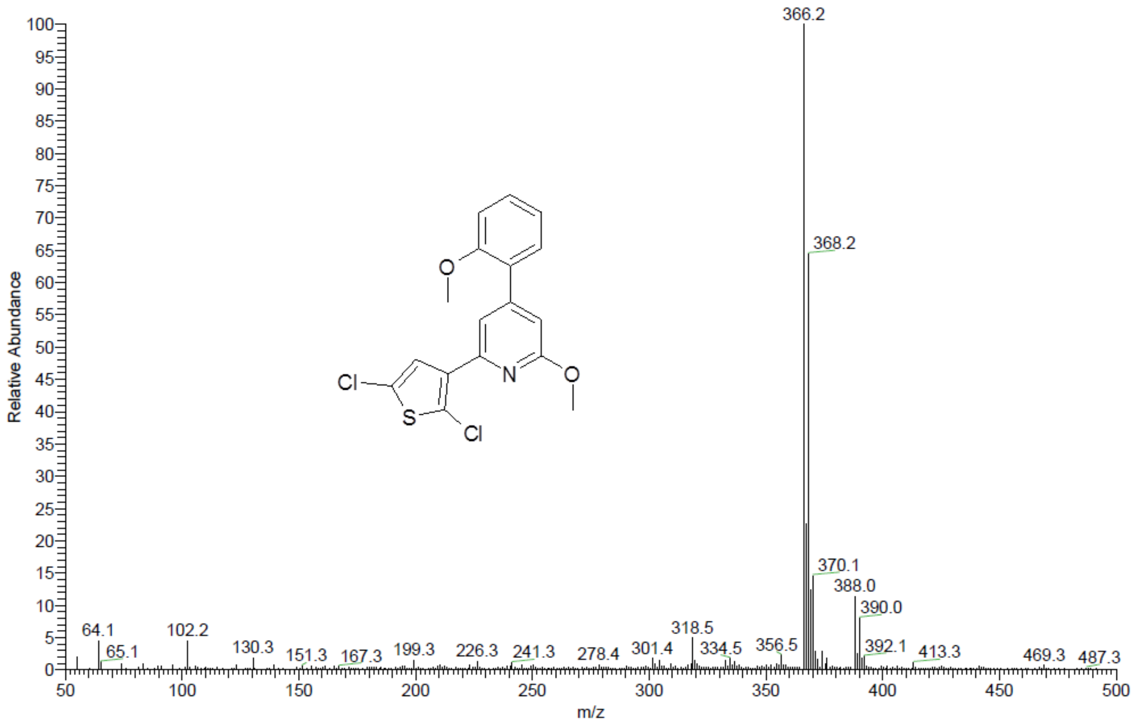

S 123: ESI mass spectrum of 2-(2,5-dichlorothiophen-3-yl)-6-methoxy-4-(2-methoxyphenyl)pyridine (**6e**).

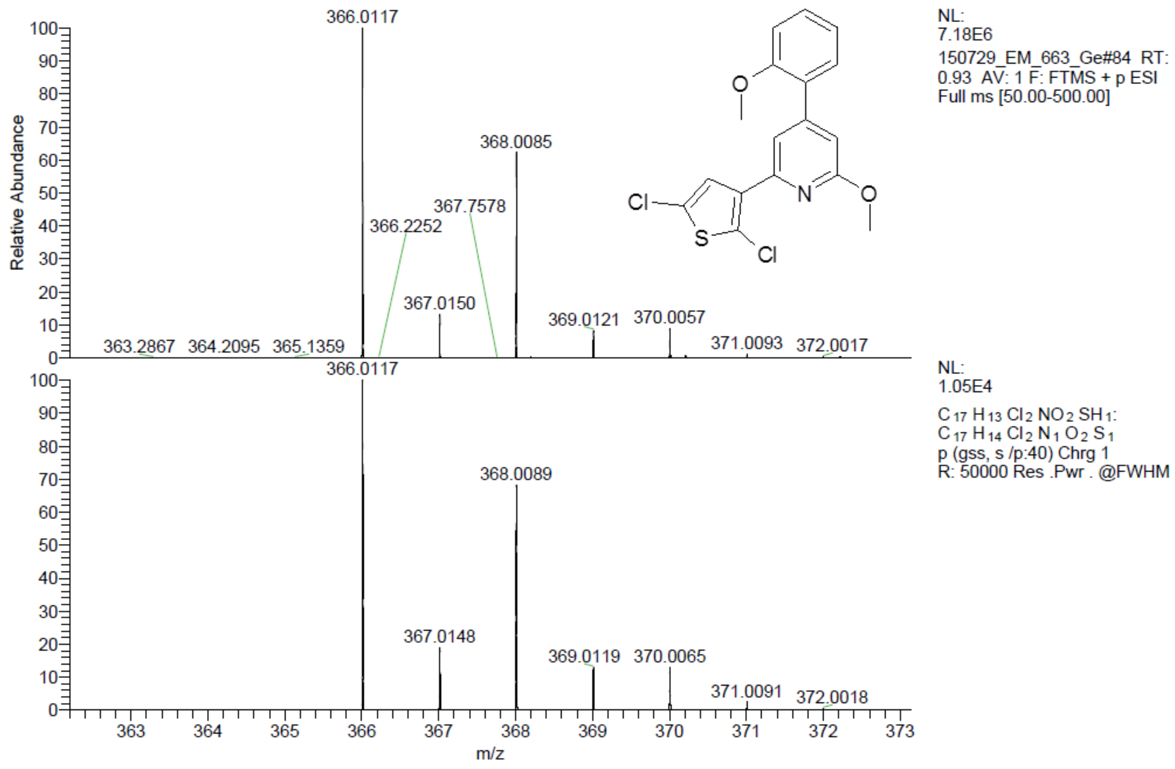

S 124: HRESI mass spectrum of 2-(2,5-dichlorothiophen-3-yl)-6-methoxy-4-(2-methoxyphenyl)pyridine (**6e**).

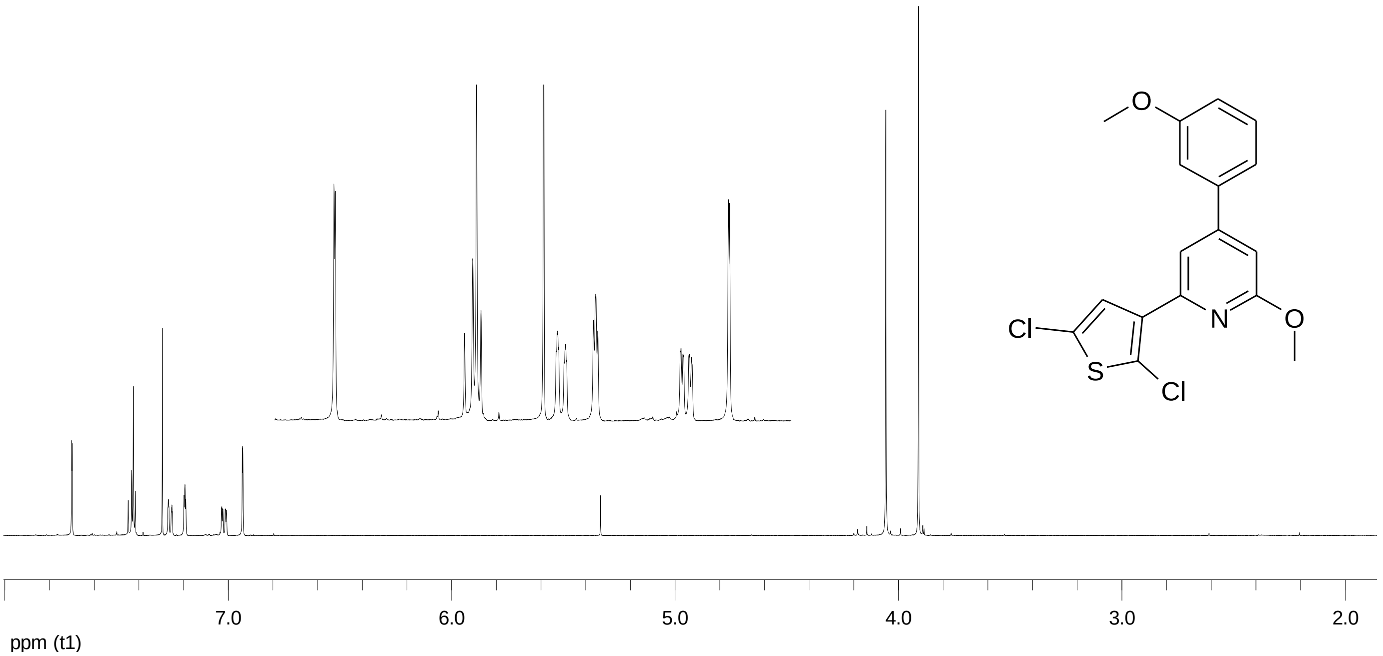

S 125: ^{1}H NMR spectrum ($CDCl_3$, 500 MHz) of 2-(2,5-dichlorothiophen-3-yl)-6-methoxy-4-(3-methoxyphenyl)pyridine (**6f**).

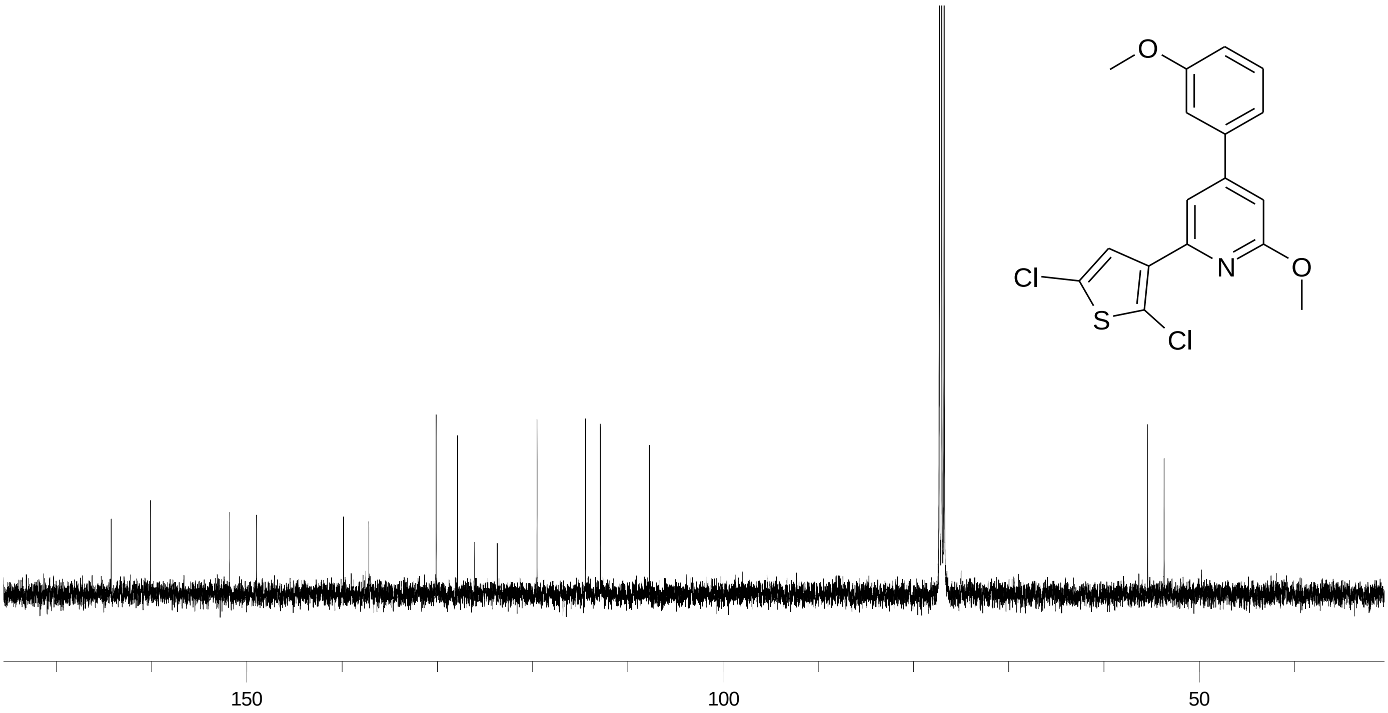

S 126: ^{13}C NMR spectrum ($CDCl_3$, 125MHz) of 2-(2,5-dichlorothiophen-3-yl)-6-methoxy-4-(3-methoxyphenyl)pyridine (**6f**).

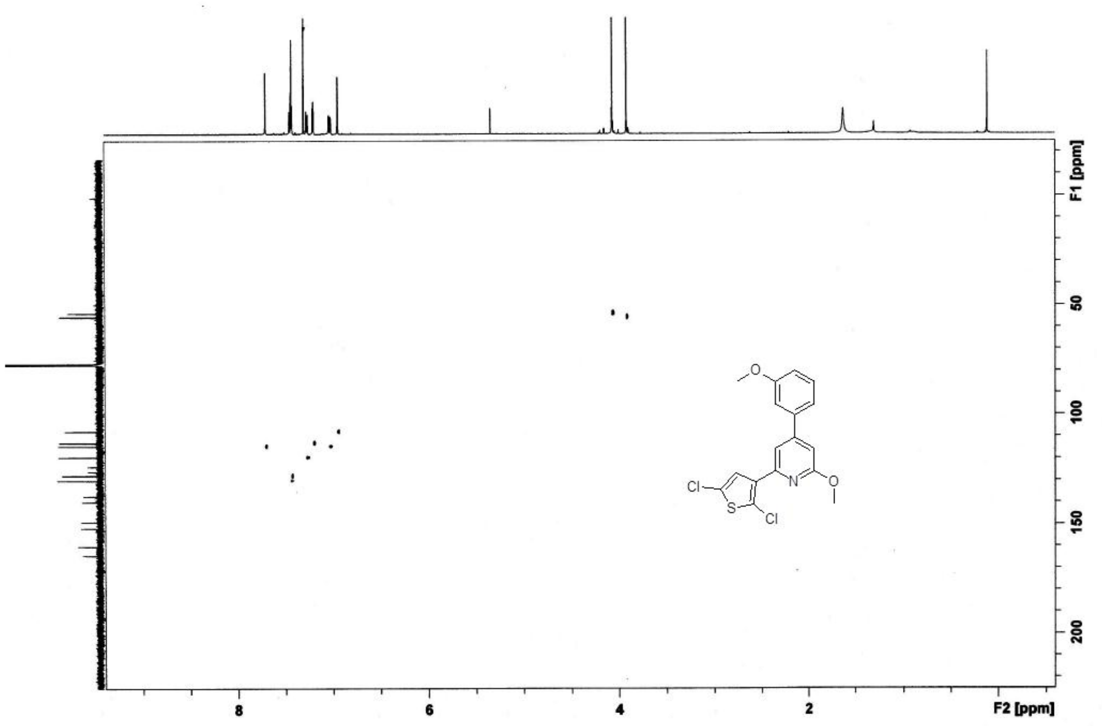

S 127: HSQC spectrum ($CDCl_3$, 500 MHz) of 2-(2,5-dichlorothiophen-3-yl)-6-methoxy-4-(3-methoxyphenyl)pyridine (**6f**).

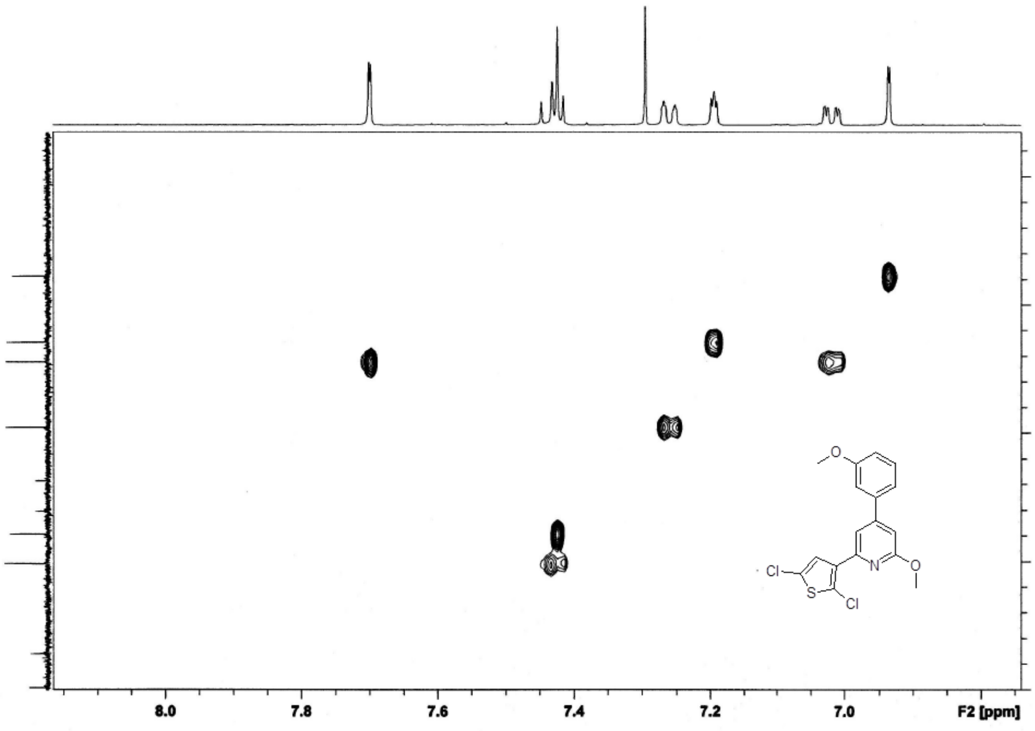

S 128: HSQC spectrum ($CDCl_3$, 500 MHz) of 2-(2,5-dichlorothiophen-3-yl)-6-methoxy-4-(3-methoxyphenyl)pyridine (**6f**).

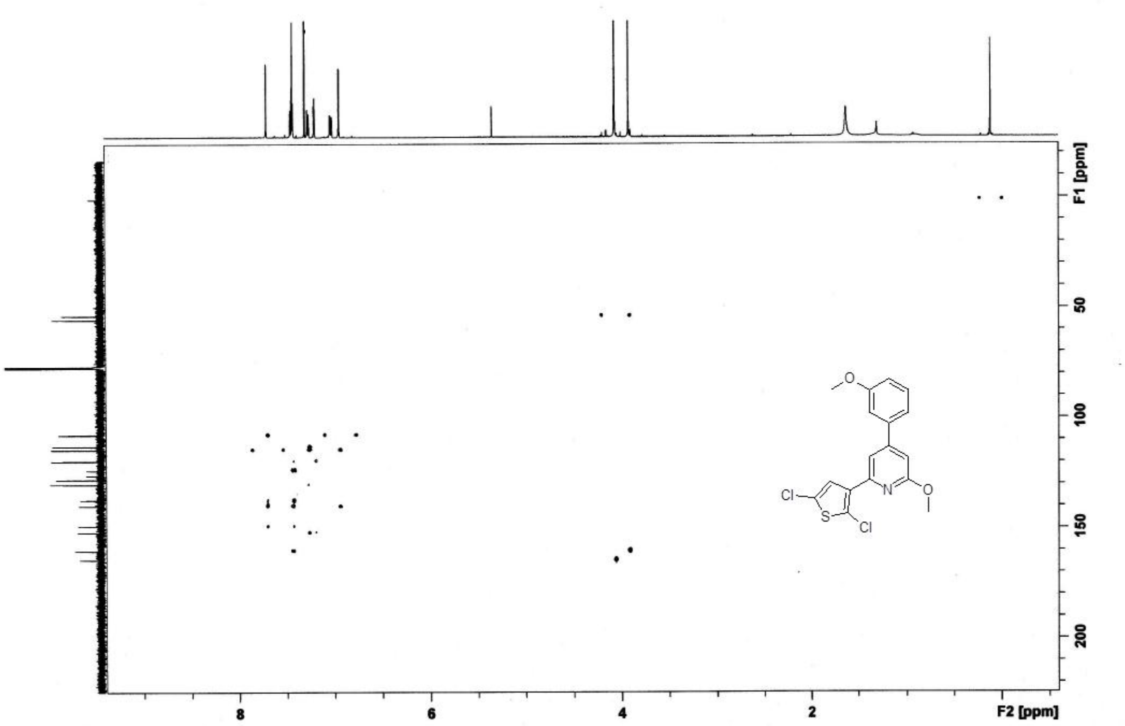

S 129: HMBC spectrum ($CDCl_3$, 500 MHz) of 2-(2,5-dichlorothiophen-3-yl)-6-methoxy-4-(3-methoxyphenyl)pyridine (**6f**).

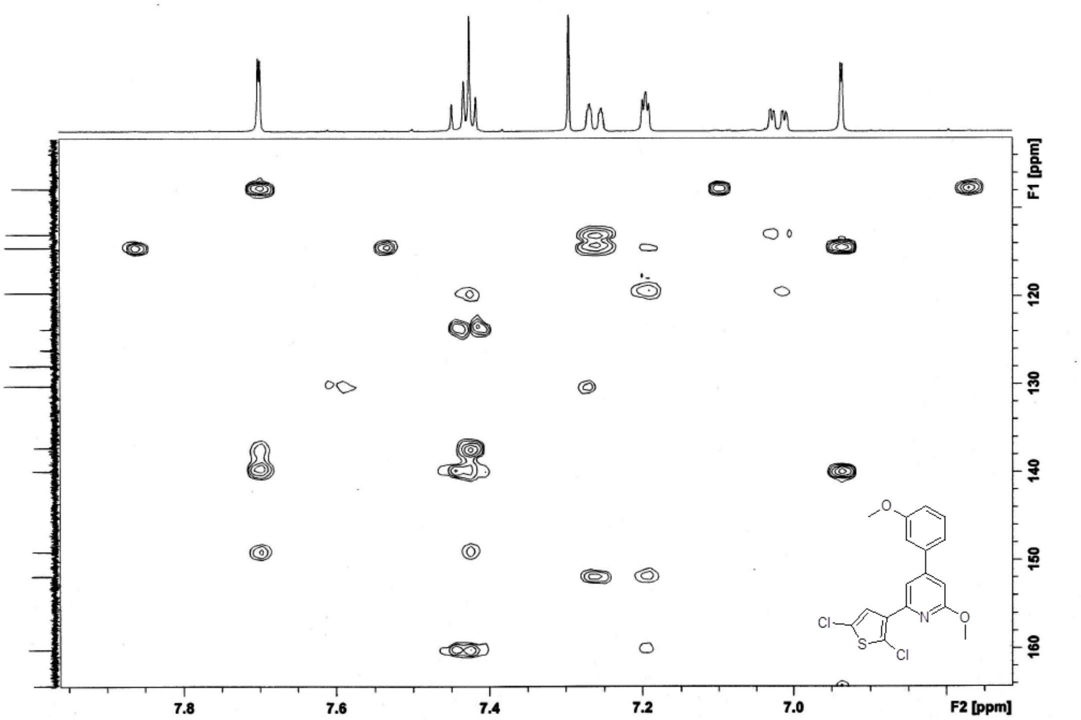

S 130: HMBC spectrum ($CDCl_3$, 500 MHz) of 2-(2,5-dichlorothiophen-3-yl)-6-methoxy-4-(3-methoxyphenyl)pyridine (**6f**).

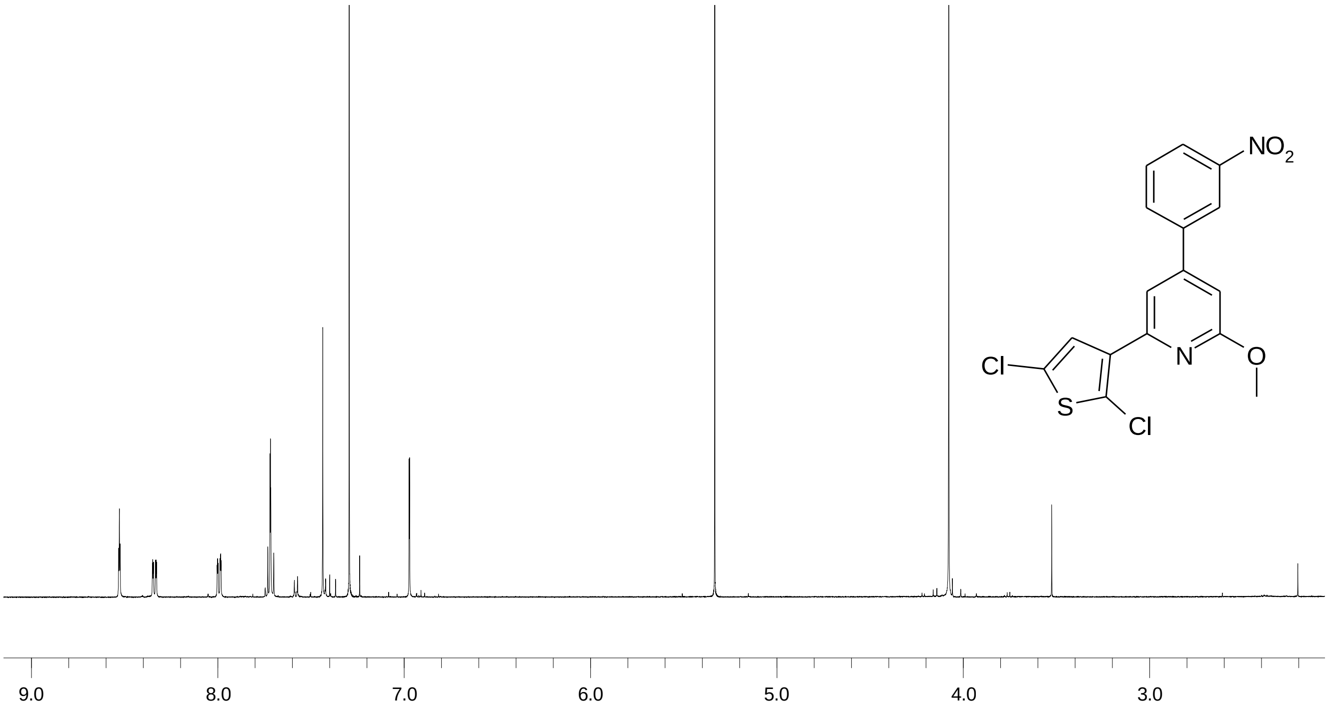

S 131: ^{1}H NMR spectrum (CDCl3, 500 MHz) of 2-(2,5-dichlorothiophen-3-yl)-6-methoxy-4-(3-nitrophenyl)pyridine (**6g**).

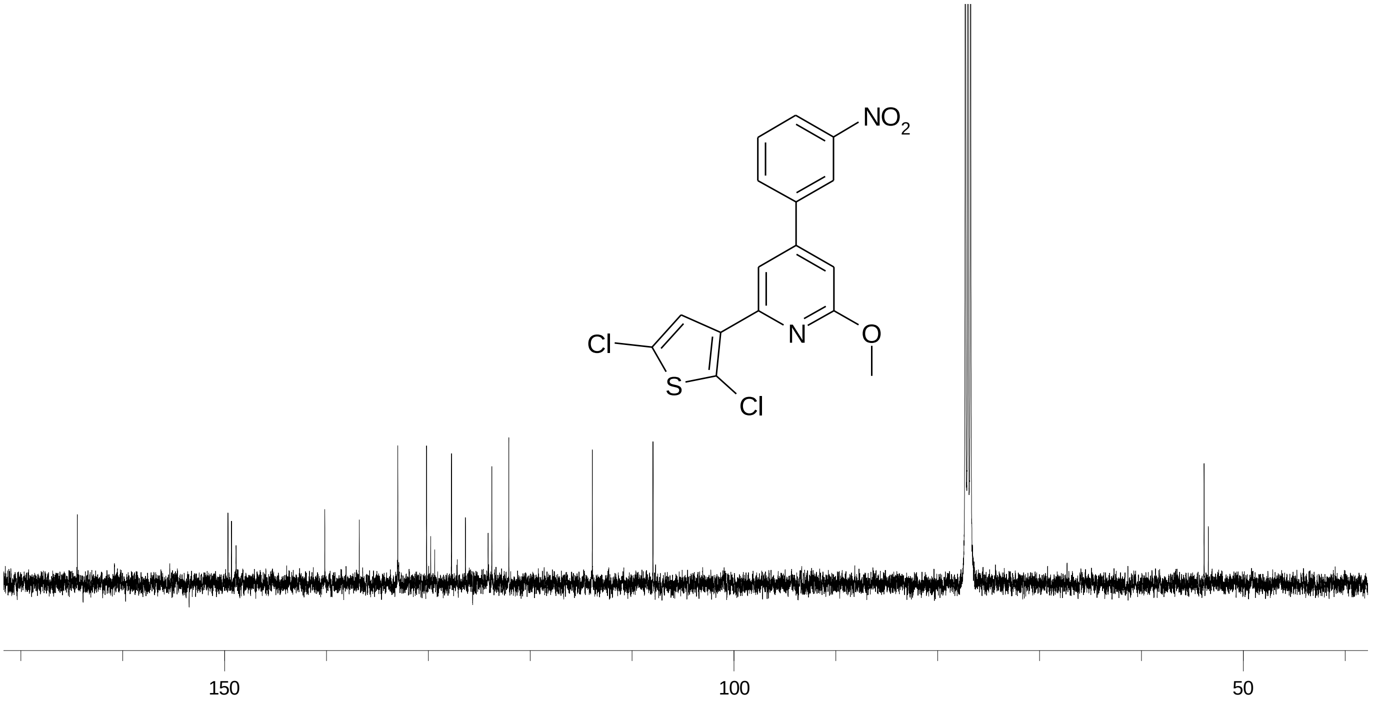

S 132: ^{13}C NMR spectrum (CDCl3, 125MHz) of 2-(2,5-dichlorothiophen-3-yl)-6-methoxy-4-(3-nitrophenyl)pyridine (**6g**).

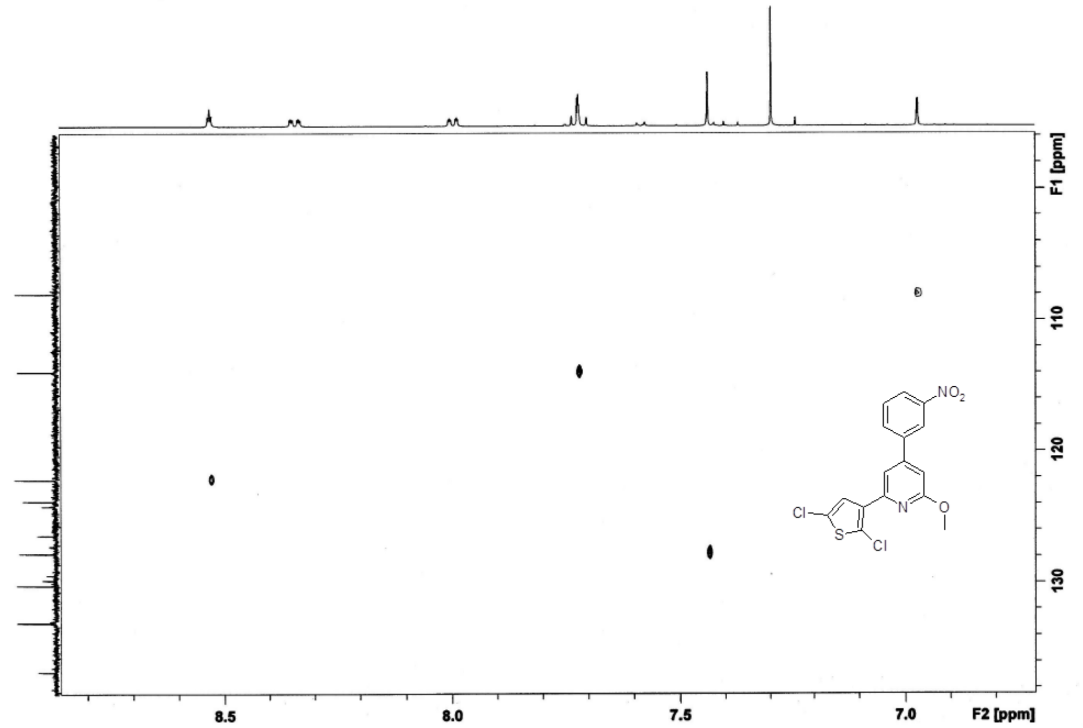

S 133: HSQC spectrum ($CDCl_3$, 500 MHz) of 2-(2,5-dichlorothiophen-3-yl)-6-methoxy-4-(3-nitrophenyl)pyridine (**6g**).

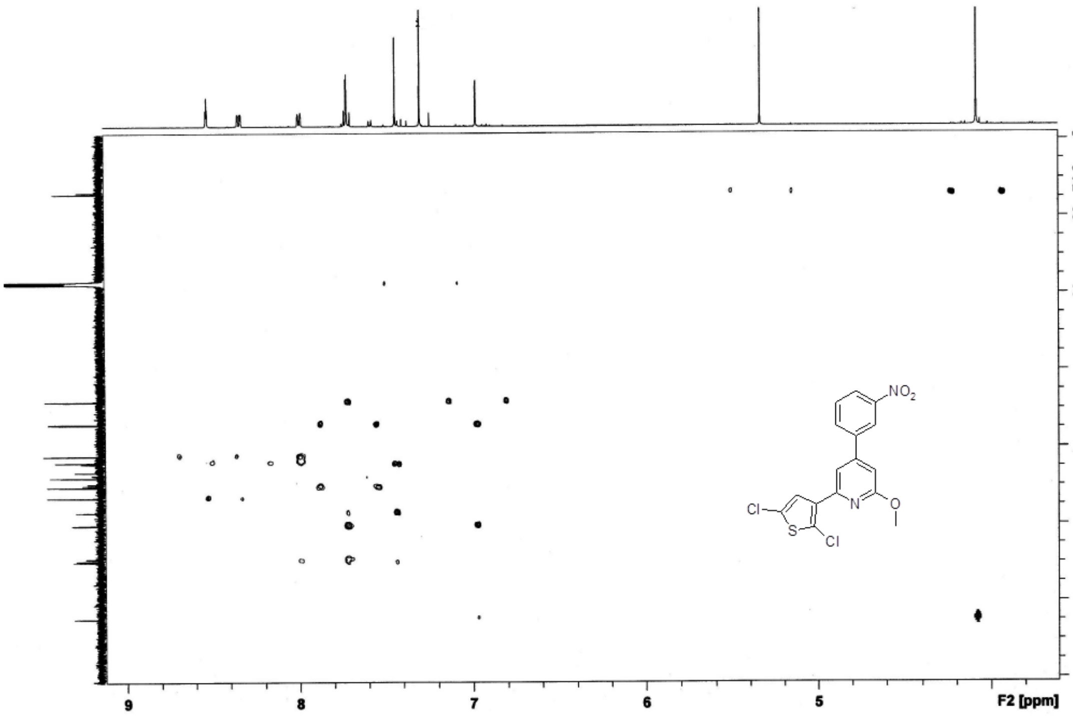

S 134: HMBC spectrum ($CDCl_3$, 500 MHz) of 2-(2,5-dichlorothiophen-3-yl)-6-methoxy-4-(3-nitrophenyl)pyridine (**6g**).

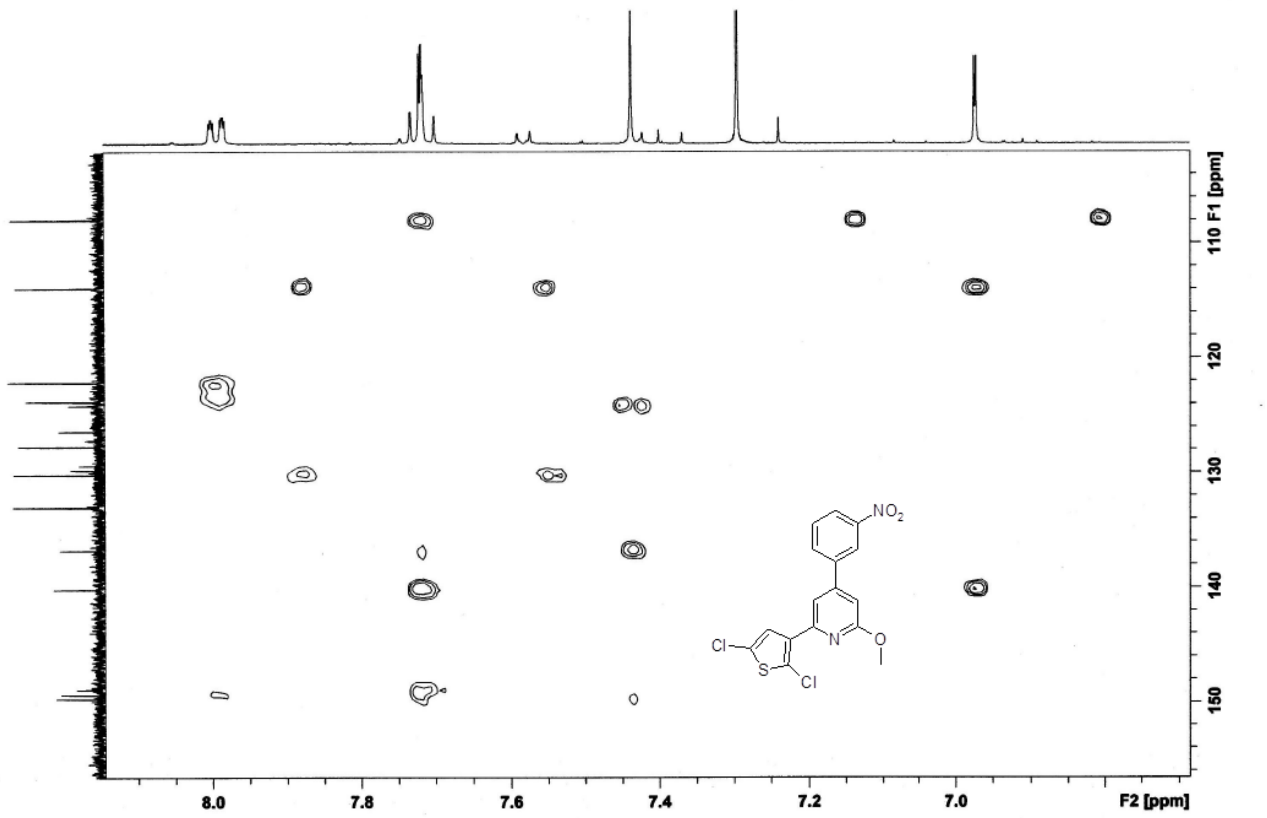

S 135: HMBC spectrum ($CDCl_3$, 500 MHz) of 2-(2,5-dichlorothiophen-3-yl)-6-methoxy-4-(3-nitrophenyl)pyridine (**6g**).

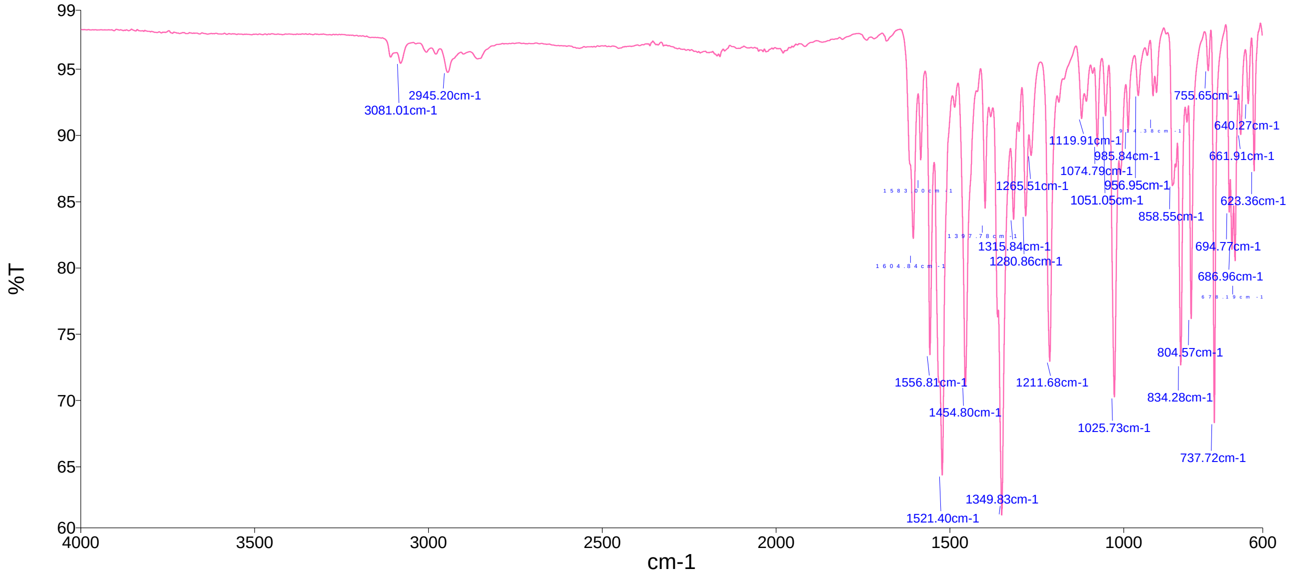

S 136: IR spectrum of 2-(2,5-dichlorothiophen-3-yl)-6-methoxy-4-(3-nitrophenyl)pyridine (**6g**).

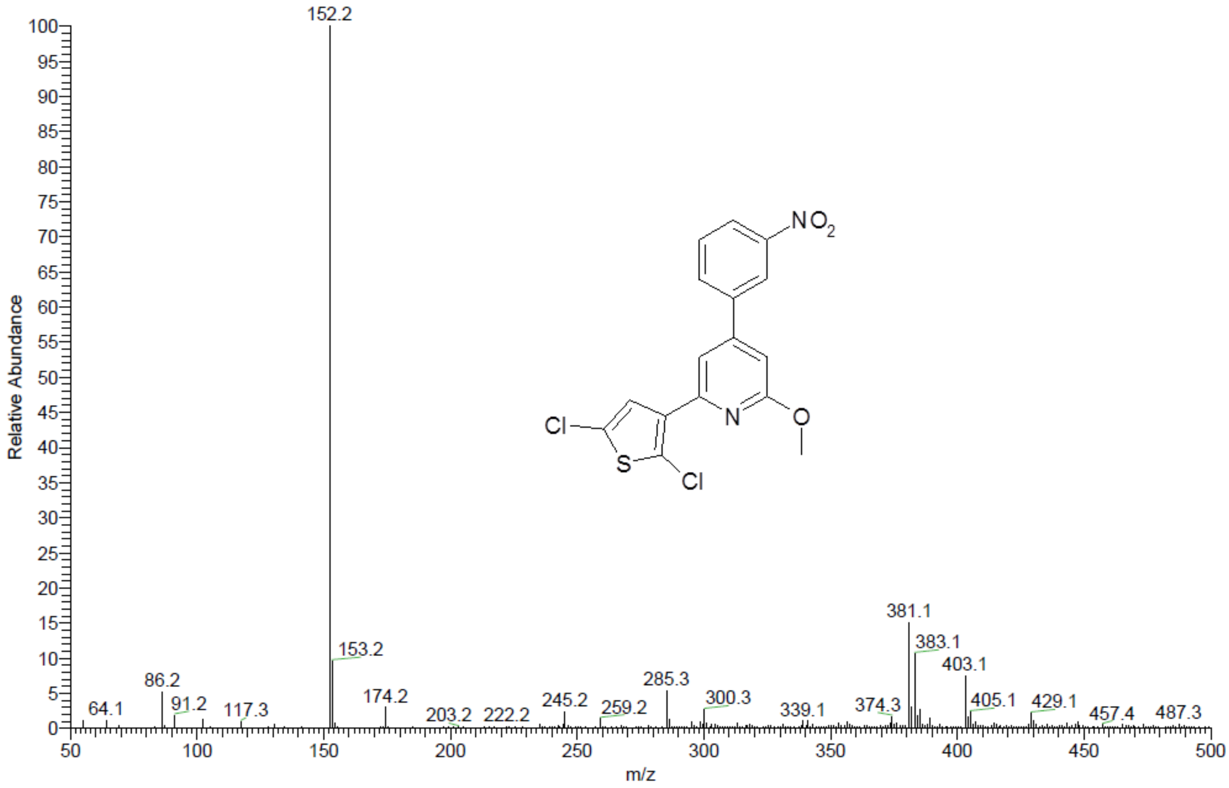

S 137: ESI mass spectrum of 2-(2,5-dichlorothiophen-3-yl)-6-methoxy-4-(3-nitrophenyl)pyridine (**6g**).

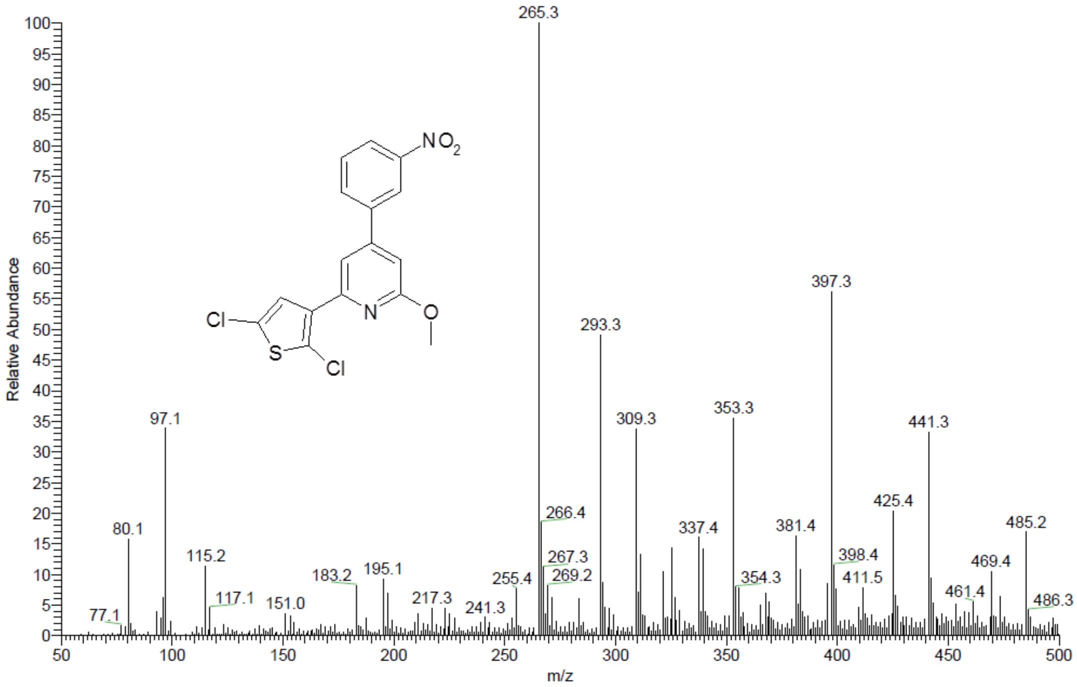

S 138: ESI mass spectrum of 2-(2,5-dichlorothiophen-3-yl)-6-methoxy-4-(3-nitrophenyl)pyridine (**6g**).

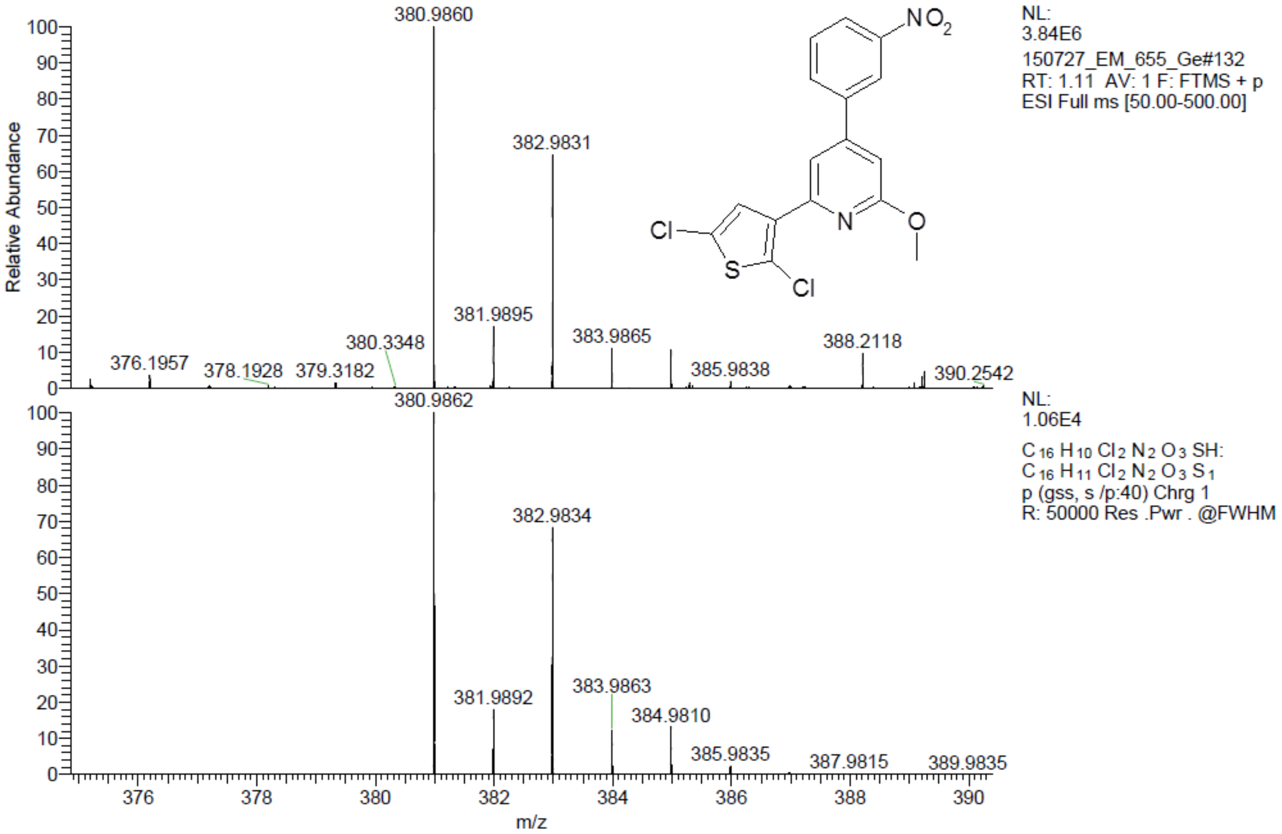

S 139: HRESI mass spectrum of 2-(2,5-dichlorothiophen-3-yl)-6-methoxy-4-(3-nitrophenyl)pyridine (**6g**).
